# Spatial transcriptomics revealed the regulatory role of CCN1 to microglia distribution through region-specific cellular interactions

**DOI:** 10.1016/j.gendis.2025.101969

**Published:** 2025-12-09

**Authors:** Zhile Bai, Zhanhe Chang, Shuguang Wang, Jiangli Zheng, Huan Wu, Hanyu Liu, Yalin Zhang, Jiayang Xie, Qingran Bai, Jiqing Yin, You Wu, Qin Shen, Shaorong Gao, Yawei Gao

**Affiliations:** a State Key Laboratory of Cardiology and Medical Innovation Center, Department of Reproductive Medicine Center, Shanghai East Hospital, Frontier Science Center for Stem Cell Research, School of Life Sciences and Technology, Tongji University, Shanghai 200092, China; b Clinical and Translation Research Center of Shanghai First Maternity & Infant Hospital, Frontier Science Center for Stem Cell Research, School of Life Sciences and Technology, Tongji University, Shanghai 200092, China; c Shanghai Institute of Stem Cell Research and Clinical Translation, Shanghai 200120, China; d School of Medicine, Tsinghua University, Beijing 100084, China; e Sycamore Research Institute of Life Sciences, Shanghai 201203, China

**Keywords:** *Ccn1*, Cellular regional interactions, Microglia, Neural stem cells, Spatial transcriptomics

## Abstract

During brain development, neural stem progenitor cells (NSPCs) and microglia interact within precise spatial niches; however, decoding mechanism is full of challenges using traditional analytical techniques. Here, we investigated the role of CCN1, a secreted protein enriched in NSPCs, using CNS-specific *Ccn1* knockout mice. Through single cell RNA-seq analysis, we found that microglia were significantly reduced in the ventricular zone (VZ) at E17.5 and P2, with elevated expression of autophagy- and activation-related genes in *Ccn1*-CKO mice. Spatial transcriptomics at P2 further showed that *Ccn1* deletion region-specific redistributes microglia and leads to increased microglial aggregation and activation in the rostral lateral septum (LSR), alongside reductions in the VZ. Mechanistically, *Ccn1* deletion in NSCs led to region-specific dysregulation of the key signaling ligands, *Csf1* and *Il2*. We found the elevated expression of *Csf1* and *Il2* specifically within the LSR region, which corresponded to the up-regulation of their respective receptors (*Csf1r*, *Cd53*) and downstream targets (*Spp1*, *Ctsb*) in LSR microglia, affecting microglial status. Our study reveals a region-specific NSC–microglia interaction regulated by *Ccn1*, offering a new paradigm for understanding multicellular dynamics in brain development.

## Introduction

During brain development, besides diverse types of cell differentiation, the establishment of specific ventricular and regional structures is extremely essential.^1^^,^^2^ Both events are coordinated by extensive interactions between different cellular populations, which collectively shape the brain microenvironment and are critical for both neurodevelopment and functional organization.^3^, ^4^, ^5^ However, deciphering these processes remains challenging due to the sheer complexity and diversity of brain cell types, as well as the spatial heterogeneity in gene expression profiles among the same cell types across distinct anatomical regions.^6^, ^7^, ^8^ Recently, advances in single-cell and spatial transcriptomics technologies have enabled high-resolution characterization of brain cell populations and their spatial organization.^9^^,^^10^ These technologies provide a powerful foundation for resolving the cellular and molecular architecture of the developing brain.

Microglia play a pivotal role in regulating the brain microenvironment. Derived from yolk sac progenitors, microglial precursors migrate into the brain around embryonic day 9.5 (E9.5), and eventually comprise approximately 5%–12% of the total brain cell population, depending on the specific brain region.^11^^,^^12^ Microglia exhibit diverse functional states and respond dynamically to external stimuli. Through state transitions, they perform a variety of roles—including phagocytosis and secretion of signaling molecules—that contribute to the fine-tuning of brain development and function.^13^^,^^14^ For instance, as the resident macrophages of the central nervous system (CNS), microglia regulate CNS neuroinflammation for the damage response,^15^ and are involved in numerous neurological diseases, such as Alzheimer's disease, Parkinson's disease, and amyotrophic lateral sclerosis.^16^, ^17^, ^18^ Meanwhile, microglia also control the synaptic plasticity, neuronal excitability, neurogenesis, phagocytosis of neuronal precursor cells, and the clearance of dead cells and debris, which ensures brain development and CNS homeostasis.^19^, ^20^, ^21^, ^22^, ^23^, ^24^ Recent single-cell and spatial transcriptomic studies have revealed that microglial transcriptomic states vary across developmental stages and brain regions. These differences may be associated with pathological conditions. For instance, genes highly expressed in activated response microglia (ARMs), such as Apolipoprotein E (*Apoe*), Cathepsin B (*Ctsb*), Lysozyme 2 (*Lyz2*), Secreted Phosphoprotein 1 (*Spp1*), and Cathepsin D (*Ctsd*), are also prominently up-regulated in disease-associated microglia (DAMs), particularly in the context of neurodegeneration and aging.^25^, ^26^, ^27^ However, the underlying causes of these diverse microglial states remain unclear. Notably, interactions between microglia and other neighboring cell types within the brain microenvironment are likely to play a critical role, yet the molecular mechanisms governing these interactions warrant further investigation. Recent studies have shown that microglia and NSCs are well-positioned to interact with each other: microglia could influence the proliferation and differentiation of NSCs in both adult and embryonic mice.^28^, ^29^, ^30^ Alternatively, NSCs could affect microglial proliferation, migration, and phagocytic activity.^31^, ^32^, ^33^ However, the cellular and molecular mechanisms by which NSCs impact microglia during brain development have not yet been properly established.

In our study, we focused on investigating the role of cellular communication network factor 1 (*Ccn1*) in brain development and the regulation of microglial function. *Ccn1,* originally named *Cyr61*, belongs to the *Ccn* gene family encoding secretory proteins. *Ccn1* is known to affect the growth and behavioral environment of target cells in various ways outside the cell. After secretion, CCN1 binds to cell membranes and extracellular matrix (ECM) and regulates cell adhesion, migration, proliferation, and apoptosis.^34^, ^35^, ^36^ The Allen Brain Atlas database showed that the mRNA of *Ccn* family genes was expressed in various regions of the central nervous system at multiple stages of development and in adulthood. Particularly, *Ccn1* was mainly expressed in the germinal region around the ventricle in the telencephalon in the fetal brain, and in the margin of the ventricle and neurons in the visual cortex after birth,^37^ suggesting its potential role in brain development. Our previous study demonstrated that in the adult mouse brain, *Ccn1*, which is especially expressed in ependymal cells, could negatively regulate niche capacity and NSC number in the ventricular–subventricular zone.^38^ However, whether *Ccn1* plays a regulatory role in the developing mouse brain has not been previously studied.

In this study, we integrated published single-cell transcriptomic datasets from embryonic mouse brains with immunofluorescence staining and RNA *in situ* hybridization to demonstrate that *Ccn1* is predominantly expressed in neural stem and progenitor cells (NSPCs). To investigate its function, we generated the *Nestin*-Cre/*Ccn1*^flox/flox^ mice^39^ to achieve central nervous system-specific deletion of *Ccn1*. Through single-cell RNA sequencing, immunostaining, and fluorescence-activated cell sorting (FACS) analysis, we found that *Ccn1* loss not only impaired NSC differentiation but also led to a significant reduction in microglial cell numbers and altered the expression of genes associated with autophagy and immune activation. Spatial transcriptomics further showed that *Ccn1* deficiency disrupted the spatial and temporal expression patterns of NSCs. Notably, the expression of *Csf1* and *Il2*—critical for microglial recruitment and activation—was decreased in ventricular zone (VZ) NSCs but increased in those of the lateral subventricular region (LSR), thereby reshaping microglial distribution and transcriptional states via *Csf1*–*Csf1r* and *Il2*–*Cd53* signaling.

Taken together, these findings uncover a region-specific, *Ccn1*-mediated mechanism of NSC–microglia crosstalk and highlight the utility of spatiotemporal transcriptomic approaches for decoding cellular interactions within the developing brain microenvironment.

## Materials and methods

### Mice

**Figure 1:**
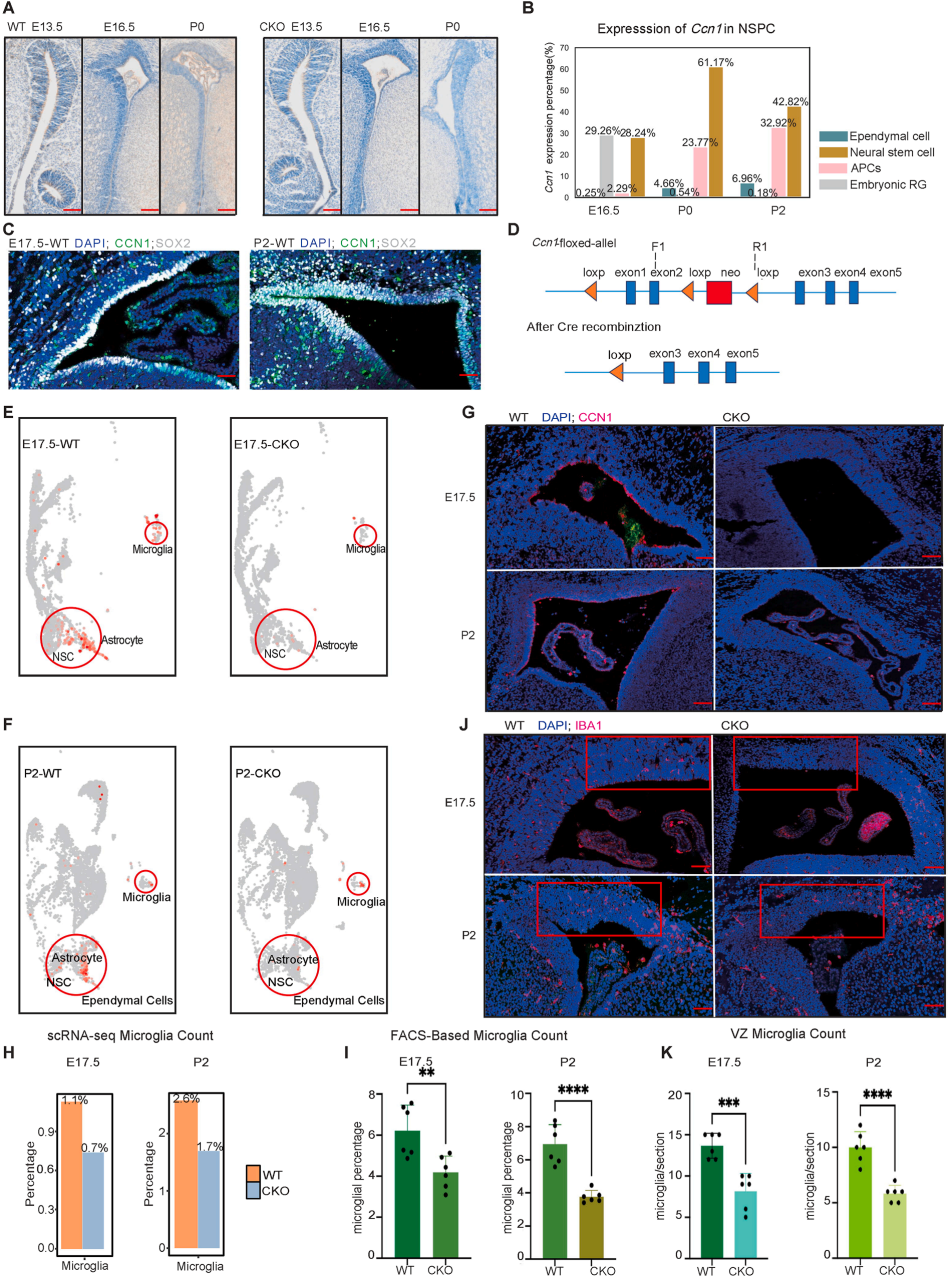
*Ccn1* deletion in neural stem progenitor cells (NSPCs) leads to a reduction of microglia in the brain at E17.5 and P2. **(A)** Immunohistochemistry analysis of CCN1 in wild type (WT) and conditional knockout (CKO) brains at E13.5, E16.5, and P0. Scale bar: 50 μm. **(B)** Expression of *Ccn1* in NSPCs of the mouse brain at E16.5, P0, and P2 from published data. **(C)** CCN1 is highly expressed in NSCs at E17.5 and P2, as confirmed by co-immunostaining of CCN1 (green) and SOX2 (silver). Scale bar: 50 μm. **(D)** Schematic diagram of the generation of *Ccn1*-CKO mice using Nestin-Cre. **(E, F)** Confirmed the expression specificity and CKO efficiency of *Ccn1* in WT and CKO mouse brains at E17.5 (E) and P2 (F), as validated by scRNA-seq.**(G)** CCN1 expression is nearly absent in E17.5 and P2 CKO mouse brains, as shown by co-immunostaining of DAPI (blue) and CCN1 (red). Scale bar: 50 μm. **(H)** The percentage of microglia was significantly reduced at E17.5 and P2 according to scRNA-seq analysis. **(I)** The proportion of microglia in the brain was significantly decreased upon *Ccn1* depletion in NSPCs at E17.5 and P2, as determined by FACS analysis using ∗tmem119-tdTomato; Nestin-cre; Ccn1flox/flox∗ mice (*n* = 6 brains per group; E17.5, *p* = 0.0071; P2, *p* < 0.0001). **(J, K)** Microglia were markedly reduced in the ventricular zone (VZ), as shown by immunofluorescence of the microglial marker IBA1 (J) and statistical analysis of IBA1^+^ nuclei in the VZ (K) (*n* = 6 mice per group; E17.5, *p* < 0.001; P2, *p* < 0.0001). Scale bar: 50 μm. Data information: In (I) and (K), data are presented as mean ± SEM. Unpaired Student's *t*-test (two-tailed) was used: ∗∗*p* < 0.01, ∗∗∗*p* < 0.001, ∗∗∗∗*p* < 0.0001.

The *Nestin-Cre* transgenic mouse strain (JAX stock #003771), originally developed by Tronche et al,^40^ was generated using a transgenic construct in which the Cre recombinase gene is controlled by the promoter and enhancer elements located in the second intron of the rat *Nestin* gene to drive Cre expression in neuronal and glial precursor cells, enabling genomic recombination specifically within the central nervous system^41^ (CNS). *Ccn1*^*flox/flox*^ (generously provided by Lau, L. F.),^39^ Figure 1D schematic diagrams showing the targeted *Ccn1* genomic locus with the *Ccn1*flox-neo allele, following Cre-mediated excision deleted *Ccn1* by recombination performed with flox sites 2 and 3. NSC-specific *Ccn1* knockout mice were generated by crossing *Nestin-Cre* with *Ccn1*^*flox/flox*^. T*mem119-*tdTomato mice constitute a reporter mouse model targeting a microglia-specific marker, T*mem119*, for studying microglia. NSC-specific *Ccn1* knockout microglia-labeled mice (*Nestin-Cre; Ccn1*^*flox/flox*^; T*mem119-*tdTomato) were generated by crossing *Nestin-Cre* with *Ccn1*^*flox/flox*^ and *Tmem119-*tdTomato.

The genotypes of the transgenic mice were detected by agarose gel electrophoresis of the PCR amplification products. The positive band size of *Nestin*-WT is 246 bp, the positive band size of *Nestin*-Cre is 150 bp, the positive band size of *Ccn1*-flox wild-type is approximately 500–600 bp, and the positive band size of Ccn1-flox is approximately 600–700 bp. The primers used were as follows: (*Ccn1*-flox) F1: TTGCTAAAGCGCTACATAGGA; (*Ccn1*-flox) R1: GCCTTATTGTGGAAGGACTG; (*Nestin*-wt) F3: CCTTCCTGAAGCAGTAG AGCA; (*Nestin*-cre) F2: CTGCTGTAAGGTCTGCGCTA; (Cre) R2:GGAAGCTT TCCCCGTTTTGG.

All the mice were housed in a specific pathogen-free (SPF) animal facility at Tongji University, Shanghai, China. All animal care and experimental protocols have been approved by the Laboratory Animal Welfare and Ethics Committee of Tongji University (TJAB08924102). Both males and females aged 8–10 weeks were used. All animal experiments and breeding procedures were performed in accordance with the experimental animal use guide of Tongji University.

### Tissue processing, immunostaining, and imaging

For coronal section preparation, the mice were deeply anesthetized with 1% pentobarbital sodium (i.p.) and transcardially perfused with saline, followed by 4% PFA. The brains were post-fixed overnight at 4 °C in 4% PFA for 18 h and cryoprotected in 30% sucrose for 2 days at 4 °C. Fixed tissues were embedded in optimal cutting temperature (OCT) compound and cut into 15-um-thick coronal sections. The sections were blocked with 5% BSA containing 0.3% Triton X-100 and stained with primary antibodies overnight at 4 °C. The primary antibodies used in this study were as follows: IBA1 (Santa Cruz, sc-393770), CTSB (CST, 31718T), LC3 (CST, 4108). DAPI (Sigma–Aldrich D9542) was used to stain nuclei for 15–20 min at room temperature, followed by incubation with secondary antibodies for 2 h at room temperature. The samples were washed with DPBS 3 times before imaging. All the prepared samples were observed and photographed with a Zeiss LSM 880 confocal microscope. Simple image processing was performed using Zeiss processing software.

### RNA fluorescence *in situ* hybridization (FISH) assays

For the FISH assay data, we used SEERNA® ISH RNA fluorescence *in situ* detection kit (Dynamic Biosystems). The overall steps were as follows: cortex sections in 10-μm thickness were first fixed in 4% (w/v) paraformaldehyde (Sigma) for 5 min, and washed twice with diethyl pyrocarbonate (DEPC)-treated PBS, followed by dehydration in an ethanol series. After three washes with wash buffer (Dynamic Biosystems), the sections were permeabilized with 0.1 M HCl at 37 °C for 5 min.

The sections were then incubated in order with the following solutions: target probes in hybridization mix at 37 °C for 4 h, ligation mix at 37 °C for 30 min, splint primers in the circularization mix at 37 °C for 30 min, and amplification mix for rolling circle amplification (RCA) at 30 °C overnight. The slides were washed with wash buffer (Dynamic Biosystems) three times at RT after each step. After that, the sections were incubated in hybridization buffer containing the label probes at RT for 30 min. Finally, the slides were mounted with SlowFade Gold Antifade Mountant (Thermo Fisher Scientific) medium containing 0.5 μg/mL DAPI (Sigma), which were then ready for image acquisition.

### *In situ* sequencing (ISS)

Dynamic Biosystems (Suzhou, China) for carrying out the RNA improved *in situ* sequencing (ISS) assay and its data analysis. ISS introduces paired-end sequencing and a 2-base combinatorial encoding strategy for barcode interrogation, which can improve not only signal intensity but also specificity for ISS. The label sequencing interrogation probe was conjugated to Cy3, Texas Red, Cy5 or Alexa Fluor 750 (Life Technologies, Thermo Fisher Scientific, Shanghai, China).

Tissue sections in 10-μm thickness were first fixed in 4% (w/v) paraformaldehyde (Sigma, Shanghai, China) for 5 min, and washed twice with diethyl pyrocarbonate (DEPC)-treated PBS, followed by dehydration in an ethanol series. After three washes with wash buffer (Dynamic Biosystems, Suzhou, China), the sections were permeabilized with 0.1M HCl at 37 °C for 5 min.

The sections were then incubated in order with the following solutions: target probes tagged with a specific barcode for each RNA molecule in hybridization mix at 37 °C for 4 h, ligation mix at 37 °C for 30 min, splint primers in the circularization mix at 37 °C for 30 min, and amplification mix for rolling circle amplification (RCA) at 30 °C overnight. The slides were washed with wash buffer (Dynamic Biosystems, Suzhou, China) three times at RT after each step. For sequencing, the sections were incubated in the ligation buffer of anchor primers, label sequencing interrogation probes and ligase at 30 °C for 45 min, followed by mounting with SlowFade Gold Antifade Mountant (Thermo Fisher Scientific, Shanghai, China) medium containing 0.5 μg/mL DAPI (Sigma, Shanghai, China). The images were acquired by using the 3D-Hitech Pannoramic®MIDI II equipped with a pco.edge 4.2bi camera using a 20× objective lens. A round of DAPI images is selected as the reference image, the SIFI algorithm is used to obtain the features of the DAPI images of different rounds, the feature points of the DAPI images of different rounds are matched, and the matching feature points obtained are used for affine transformation to register the images of different rounds. Then, the TopHat of OpenCV is used for background filtering. By recognizing the real signal and decoding the signal, the spatial localization and quantification of RNA are finally confirmed.

### FACS analysis

The cortex was dissected, minced, and digested in 0.05% trypsin EDTA at 37 °C for 10 min. Digested cortex pieces were centrifugated and washed three times in 1% BSA in PBS. The cell suspension was filtered through a 40-um mesh to remove large tissue pieces. The cells were washed and resuspended in FACS buffer. All analyses were performed on a MoFlo XDP cell sorter (Beckman Coulter).

### Western blotting

Protein samples were separated on SDS–PAGE and transferred onto PVDF membranes. After blocking, the membranes were incubated with primary antibodies overnight at 4 °C followed by secondary antibodies for 1 h at room temperature. The primary antibodies used in this study were as follows: LC3 (CST, 4108), GAPDH (Proteintech, 60004-1-IG). The signal was detected with the corresponding HRP-conjugated secondary antibodies and an enhanced chemiluminescence (ECL) immunoblotting detection system (Sigma–Aldrich).

### Single cell RNA library preparation

For single cell RNA data, we used the Chromium platform (10x Genomics) to generate scRNA libraries. The overall steps were as follows: the cortex was dissociated with pestle and lysed on ice for 10 min, after which the dissociated tissue was transferred to a nucleus isolation column. After a series of centrifugation procedures, the single nucleus suspension was obtained.

The isolated nuclei were then washed, resuspended and loaded directly into compatible 10x Genomics singlecell assays. A large number of nuclei were transposed, and then individual nuclei were captured in GEMs, where the mRNA 3′ ends were labeled with barcodes. scRNA libraries were generated from each sample.

### Spatial transcriptomics library preparation

Spatial transcriptomics libraries were prepared with STOmics Stereo-seq kit from BGI (STOmics Tech; 101KL114). The steps include tissue collection, tissue processing and imaging, tissue permeabilization, cDNA synthesis, Stereo-seq library preparation, and library construction and sequencing. Overall, the fresh mouse brain was embedded in molds with OCT and flash frozen using liquid nitrogen, then stored at −80 °C. Before the tissue patch, the sample was placed in a Leica CM1950 cryostat for 30 min to equalize the temperature, and then the embedded tissue was cut to a thickness of 10 μm. The cryosections were adhered to the surface of the Stereo-seq capture chip and incubated at 37 °C for 5 min. The chip with the section was subjected to the steps of fixation, staining and imaging, permeabilization, and reverse transcription. Finally, the chip was digested with tissue removal buffer and then treated with cDNA release mix for 6 h at 55 °C to collect cDNA for the next step of library construction.

After purification, 20 ng of cDNA products were fragmented, and the fragmentation products were then amplified using the Stereo-seq library kit. Finally, the PCR products were purified using KAPA DNA Clean Beads ( × 0.55). The library was sequenced (50 bp for read 1 and 100 bp for read 2) using an MGISEQ-2000RS from BGI.

### Data analysis

The dataset information was summarized in Supplementary Table 6.

### Single cell RNA-seq analysis

Single-cell RNA sequencing was performed on E17.5 and P2 mouse brain tissues. The raw reads were assessed with FASTQC, trimmed using Trim_galore (v0.6.4), and aligned to the GRCm38 reference genome via CellRanger v2. The cells were retained if they had 500–13,000 detected genes, 500–25,000 total RNA counts, and < 10% mitochondrial content. For the E17.5 and P2 datasets (wild type (WT) and conditional knockout (CKO)), we applied the standard Seurat integration workflow.^42^ Briefly, highly variable features were identified, cross-dataset anchors were computed using canonical correlation analysis (CCA) or reciprocal principal component analysis (PCA), and batch effects were corrected via *IntegrateData*. The integrated dataset was then normalized, reduced via PCA and uniform manifold approximation and projection (UMAP), and clustered using the shared nearest neighbor (SNN) modularity optimization algorithm. Differentially expressed genes between WT and CKO were identified with the FindMarkers function, using thresholds of min.pct ≥ 0.15, log_2_ fold change (FC) ≥ 0.15, and adjusted *p* < 0.05.

### Differential expression analysis in single cell

Differentially expressed genes between WT and CKO were identified using the Seurat FindMarkers function. To increase robustness, we first restricted the analysis to genes expressed in at least 15% of the cells in either group (parameter min.pct ≥ 0.15), which helps filter out very lowly expressed genes. Genes with log_2_ FC ≥ 0.15 were initially considered as up- or down-regulated. To define significantly differentially expressed genes, we applied a more stringent cutoff of log_2_ FC ≥ 0.58 (approximately 1.5-FC) and *p* value < 0.05.

### Calculation of the module score

To define activated and quiescent microglia modules, we used *AddModuleScore* with marker genes from the relevant literature. Cell apoptosis and autophagy-related genes were defined using all the genes associated with Gene Ontology (GO) terms related to negative cell proliferation and autophagy that were up-regulated in E17.5. Additionally, we included well-known markers such as *Ctsd* and *Ctsl* (which are related to autophagy), *Bex2* and *Bad* (which are related to cell apoptosis).

### Spatial transcriptomics analysis

Mouse spatial transcriptome data were sequenced using Stereo-seq technology. Preprocessing of the raw sequencing data followed the instructions of the Stereo-seq Analysis Workflow (SAW v6.0.0, https://github.com/BGIResearch/SAW). Spatial barcodes were first filtered against a whitelist to remove low-quality barcodes. cDNA reads were then aligned to the mouse reference genome (mm10) using STAR (bcSTAR v1.0.2), and a gene-to-spot expression matrix was generated with the corresponding genome annotation.

For downstream analysis, we applied STAGATE,^43^ which constructs a spatial neighbor network (SNN) by integrating spatial adjacency with gene expression similarity. The SNN is pruned using pre-clustered expression information and modeled with a graph attention auto-encoder to jointly capture spatial and transcriptional features. To integrate the P2 WT and CKO samples, we combined the STAGATE and Harmony algorithms^44^ to remove batch effects. The Harmony-corrected embeddings were then used in Scanpy for UMAP visualization and Louvain clustering. Given that microglia are predominantly localized in the VZ and LSR regions, we extracted these specific regions for further spatial analysis. In the spatial plots, expression values were calculated by normalizing UMI counts by the total counts per cell, followed by log-transformation.

We used the differential NicheNet pipeline (https://github.com/saeyslab/nichenetr) to model spatial cell–cell communication between neural stem cells (senders) and microglia (receivers) across two niches (WT and CKO). This approach integrates ligand–receptor networks, intracellular signaling cascades, and transcription factor–target relationships to prioritize ligand–target links. We defined niches of interest, including the LSR, in WT and CKO conditions compared to the VZ. To summarize ligand specificity across niches, we used the minimum log fold change (minLFC) metric, which prioritizes ligands that are consistently up-regulated relative to the other niche. Spatial information was incorporated to prioritize LSR ligands differentially expressed between the LSR and VZ regions. Ligand activities were then predicted and averaged (scaled) for each receiver cell type. A final prioritization score for ligand–receptor–target interactions was calculated as a weighted sum of the scaled metrics.

We identified the top 50 ligands per niche and visualized the top 8 ligand–receptor pairs, with ligands ordered by their average z-score across conditions. The corresponding receptors are shown in the ligand–receptor heatmap, with scores representing interaction weights in NicheNet's integrated signaling network. In the ligand–target heatmap, we displayed regulatory potential scores for interactions between top-ranked ligands and target genes, including *Ccnd2, Spp1, Ctsb, Apoe, Hmox1, Hsp90ab1, Csf1r, Gpnmb, Grn, Hexb, Lpl, and Col4a1*. The expression of ligands and target genes was visualized using DotPlots in Seurat.

### Gene Ontology enrichment analysis

ClusterProfiler35 (version 4.8.3) R package was used for GO term analysis with default parameters.

### Gene set enrichment analysis

We performed gene set enrichment analysis using the fgsea package (v1.28.0) in R, focusing on differentially expressed genes for each cell type, and then used all genes ranked by log_2_ FC after removing low expression levels. The fgseaMultilevel function was employed with the parameter ‘nPermSimple = 10000’ to calculate enrichment scores for each cell type. The *p* value is estimated by randomly assigning phenotype labels to samples, recomputing the enrichment scores for 1000 permutations, and comparing the observed ES with the distribution of these permuted scores. NES, normalized enrichment score.

### Quantification and statistical analysis

Statistical analysis was performed using two-tailed Student's *t*-test for two-group comparison. The results were analyzed using GraphPad Prism version 10 (GraphPad Software, Inc.) R and python software. The data are presented as mean ± SEM. Significance is defined as N.S. *p* > 0.05, ∗*p* ≤ 0.05, ∗∗*p* < 0.01, ∗∗∗*p* < 0.001, ∗∗∗∗*p* < 0.0001.

## Results

### *Ccn1* deletion in NSPCs results in a significant decrease in microglia in E17.5 and P2

Our prior study revealed that *Ccn1* is specifically expressed in ependymal cells, which differentiate from NSCs after birth and become multiciliated in adult mice, and regulates the proliferation and differentiation of NSCs in adult mice.^38^ Using the Allen Brain Atlas database, we found that *Ccn1* is also specifically expressed in the germinal region around the ventricle in the telencephalon in the fetal brain,^37^ and our recent study revealed that *Ccn1* deletion in NSCs impairs NSC maintenance and neuronal differentiation (unpublished data). The evidence suggests that CCN1 may play an important role in brain development before and around birth, which aroused our research interest.

We first investigated the expression pattern of *Ccn1* during embryonic brain development using immunohistochemistry at embryonic (E) stages 13.5, 16.5, and postnatal day 0 (P0). We found that *Ccn1* was highly expressed in the ventricular zone (VZ), where neural progenitors reside (Fig. 1A, left). We then analyzed a published single-cell RNA-seq dataset (GSE200202) to provide a developmental reference for defining the expression pattern of *Ccn1* across brain cell populations.^15^ We found that *Ccn1* is predominantly expressed in neural stem and progenitor cells (NSPCs), including radial glia (RG), neural stem cells (NSCs), and astrocyte precursor cells (APCs), which exhibit self-renewal capacity or the potential to differentiate into neural lineages (Fig. S1A–S1C), which is consistent with the immunohistochemistry results. Quantitative analysis of *Ccn1*-expressing cells showed that *Ccn1* expression was primarily observed in NSCs and embryonic radial glia (embryonic RGs) at E16.5 (Fig. 1B). As development progressed toward late embryonic and early postnatal stages (E17.5–P0), embryonic RG cells were no longer detectable, but NSCs and APCs accounted for the vast majority of *Ccn1*-expressing populations during this stage (Fig. S1D). For ependymal cells, although the RNA expression of *Ccn1* was considerable, the percentage of the cell population was much lower than that of NSCs and APCs from E16.5 to P2 (Fig. S1D). These data revealed that among all stem/progenitor-like populations, NSCs consistently represented the major source of *Ccn1* expression, with the highest expression levels and relative abundance throughout this developmental stage. We then performed *Ccn1* mRNA *in situ* hybridization, combined with immunostaining of the marker for NSPCs (*Id1*) and ependymal cells (*Foxj1*) to validate the expression specificity of *Ccn1* in the brains of E17.5 and P2 mice (see Methods). Consistent with our single-cell analysis results, we found that *Ccn1* is highly expressed around *Id1* in the lateral walls of the lateral ventricles where NSCs reside (Fig. S1F). Meanwhile, the expression of *Foxj1* was rarely detected due to the low proportion of ependymal cells at these two stages (Fig. S1F). The immunostaining of CCN1 and SOX2 also proved that CCN1 was expressed in SOX2^+^ cells with a large apical surface on forebrain coronal sections, indicating that CCN1 is highly expressed in NSCs (Fig. 1C).

Due to its early and broad expression in neural stem cells and embryonic lethality in systemic *Ccn1* knockout mice,^45^ we selected *Nestin*-Cre mice for conditional knockout. We generated Nestin-Cre/*Ccn1*^flox/flox^ mice by crossing *Nestin*-Cre mice with *Ccn1*^flox/flox^ mice (Fig. 1D), and the deletion of *Ccn1* was confirmed via PCR using mouse tail genomic DNA and primers (see Methods), CKO mice showed a DNA band of approximately 700 bp DNA band (flox-cko) using primers F1 and R1, and a 150-bp DNA band (cre-cko) using primers F2 and R2 (Fig. S1E). We performed immunohistochemistry and immunostaining of CCN1 in forebrain sections from WT and *Ccn1* CKO mice at E13.5, 16.5, 17.5, and postnatal days 0, 2 (P0, P2), and confirmed that *Ccn1* expression was nearly abolished in the CKO mouse brain (Fig. 1A, right; Fig. 1G).

To systematically assess this effect, we performed single-cell RNA sequencing on forebrains from both WT and CKO mice at E17.5 and P2. We first analyzed our scRNA-seq dataset in E17.5, which included 3276 cells from WT and 3380 from CKO forebrains after QC. Clustering analysis identified 13 distinct cell populations (Fig. S1G and S1I; Table S5). As to the scRNA-seq dataset in P2, 8211 cells were defined from WT and 9386 from CKO forebrains in P2 after QC, which were clustered into 19 clusters (Fig. S1H and S1J). Consistent with previous reports, due to the temporal dynamics of cortico-neurogenesis, neurons at E17.5 remain immature, whereas by P2 neuronal migration is largely complete and neurons have acquired robust subtype-specific identities that enable clear clustering.^46^, ^47^, ^48^ The expression data of *Ccn1* in single-cell revealed that *Ccn1* expression was markedly reduced in NSCs and astrocytes, which represent NSPCs, upon knockout, while its expression remained minimal in other cell types, confirming the specificity of deletion (Fig. 1E and F). Next, we examined the cellular composition at E17.5 and found that the proportion of NSCs and certain stem/progenitor-like cell subtypes was altered in the CKO brain, indicating that *Ccn1* plays a role in regulating both progenitor maintenance and neuronal differentiation (Fig. S2A and S2B). A similar trend in cell composition changes was also observed at P2 (Fig. S2C and S2D).

Interestingly, we noted that the proportion of microglia was significantly reduced at both E17.5 and P2 (Fig. 1H). Since microglia originate from yolk sac-derived progenitors and migrate into the brain during early embryonic development (around E9.5–E10.5), the expression of *Ccn1* is not impacted in microglia in CKO mice. Therefore, the altered microglia may originate from changes in the microenvironment, which we are particularly interested in and wish to investigate further.

To confirm the decrease of microglia in CKO mice, we crossed Tmem119-tdTomato mice with *Nestin*-Cre; *Ccn1*^flox/flox^ mice to generate tdTomato-labeled microglia in WT and CKO mice (tdTomato-WT and tdTomato-CKO). We digested the cell suspension from the brains of the tdTomato-WT and CKO mice at E17.5, and performed FACS to quantify the proportion of microglia (Fig. S2E). Consistent with the sequencing data, we observed a significant reduction in the microglial proportion (Fig. 1I). We then examined microglial spatial distribution through immunostaining experiments and found that the decrease in microglia was spatially restricted, predominantly occurring in the VZ region in CKO mice at E17.5 (Fig. 1J and K). We further confirmed the reduced proportion of microglia and spatial reduction in the VZ region at P2 (Fig. 1I–K; Fig. S2F).

Taken together, these results showed that *Ccn1* is specifically expressed in NSPCs during brain development. *Nestin*-Cre-mediated knockout of *Ccn1* reduced the number of microglia, particularly in the VZ region. Interestingly, the microglial changes appeared to result from the altered CNS microenvironment following *Ccn1* deletion in NSCs and astrocytes, and these changes were sustained postnatally, which is likely to further impact the microglial immune function in the brain.

### Deletion of *Ccn1* in NSPCs induced transcriptional alterations in microglia

**Figure 2:**
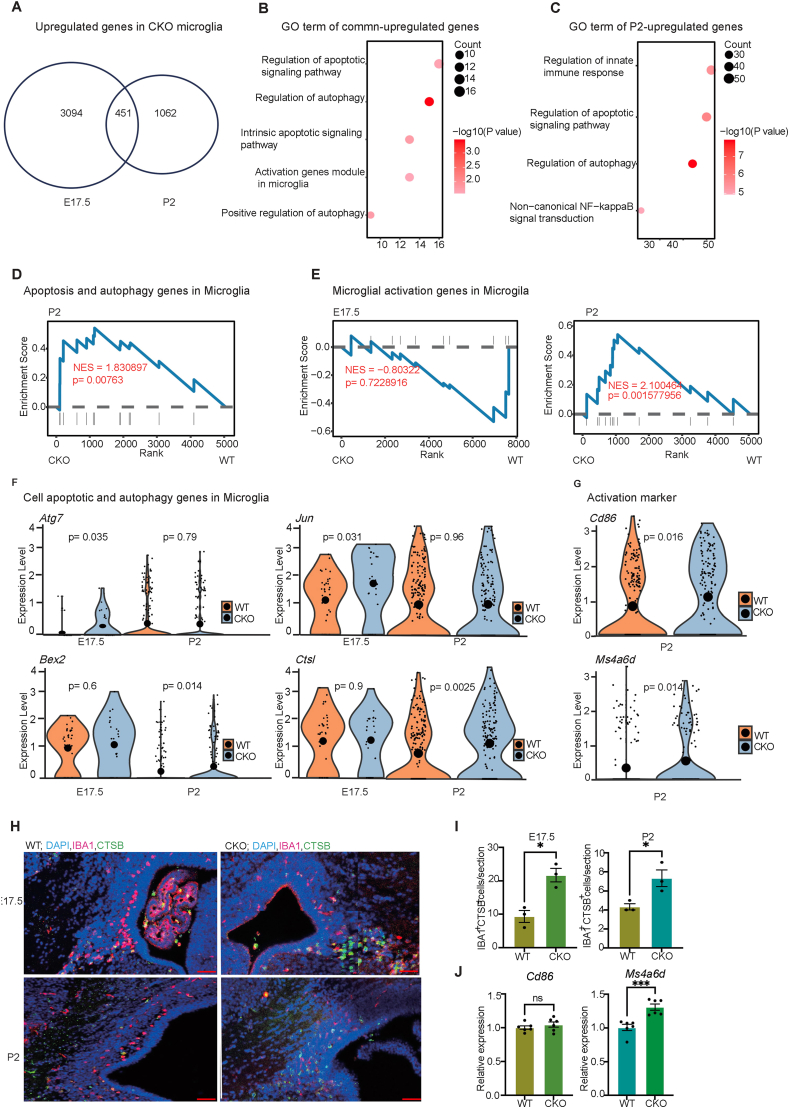
Deletion of *Ccn1* in neural stem progenitor cells (NSPCs) induces transcriptional alterations in microglia. **(A)** Identification of commonly upregulated genes in microglia upon *Ccn1* deficiency in NSPCs at E17.5 and P2. **(B)** Gene Ontology (GO) term analysis of commonly upregulated genes in microglia upon *Ccn1* deficiency in NSPCs. **(C)** GO term analysis of upregulated genes specifically in P2 microglia upon *Ccn1* deficiency in NSPCs. **(D)** Gene set enrichment analysis (GSEA) shows upregulation of apoptosis and autophagy-related genes in microglia of *Ccn1* conditional knockout (CKO) mice at E17.5 and P2. **(E)** GSEA analysis indicates downregulation of gene modules regulating microglial activation at E17.5, but upregulation at P2 in *Ccn1* CKO mice. **(F)** Expression levels of apoptotic genes (Jun, Bex2) and autophagy genes (Atg7, Ctsl) in E17.5 and P2 CKO microglia compared to WT. **(G)** Expression of microglial activation markers (Cd86, Ms4a6d) in P2 CKO microglia compared to WT. **(H, I)** Autophagy-related protein CTSB is highly expressed in microglia of E17.5 and P2 CKO mice, as shown by co-immunostaining of IBA1 (red) and CTSB (green) (H), with statistical analysis of IBA1^+^/CTSB^+^ nuclei (I) (*n* = 3; E17.5, *p* = 0.0101; P2, *p* = 0.0335). Scale bar: 50 μm. **(J)** qRT-PCR analysis of Cd86 and Ms4a6d expression in microglia of P2 WT and CKO mice (*n* = 3; Cd86, *p* = 0.3799; Ms4a6d, *p* = 0.0009). Data information: In (I) and (J), data are presented as mean ± SEM. Unpaired Student's *t*-test (two-tailed) was used: ∗*p* < 0.05, ∗∗∗*p* < 0.001; *ns*, not significant.

To elucidate the detailed molecular state transitions of microglia after *Ccn1* knockout, we further analyzed the transcriptomic profiles of microglia in our single-cell transcriptome datasets. We identified 154 significantly up-regulated and 189 significantly down-regulated genes (Tables S3 and S4) in CKO mouse microglia at E17.5 (log_2_ FC ≥ 0.58; adjusted *p* < 0.05; see Methods for details) (Fig. S3A). GO analysis of all the up-regulated genes revealed strong enrichment of autophagy, apoptosis, and negative regulation of the cell cycle (Fig. S3C). At P2, 177 genes were significantly up-regulated and 136 were significantly down-regulated (Fig. S3B), with GO analysis further highlighting neuroimmunity-related pathways in addition to the enrichments observed at E17.5 (Fig. 2C). These data indicate that *Ccn1* deletion in NSCs induces substantial transcriptional changes in microglia, with stage-specific differences across developmental time points. We then took the intersection of up-regulated genes in microglia from CKO mice at E17.5 and P2 and found 451 transitionally active genes (Fig. 2A). GO analysis indicated that the functions of these genes were mainly related to apoptosis, autophagy, and immune response (Fig. 2B). We selected apoptosis- and autophagy-related genes that were up-regulated at E17.5 (cell apoptosis and autophagy genes) (Fig. S3D) and found that these genes were up-regulated overall at P2 by gene set enrichment analysis (GSEA) (Fig. 2D), although they were more significantly up-regulated at E17.5 (Fig. S3D). Among these genes, the autophagy gene *Atg7* was significantly up-regulated in the microglia of CKO mice at E17.5. Besides, *Ctsl,* which is involved in microglia-mediated neuroinflammation, promoted autophagy in microglia and was significantly up-regulated in the microglia of CKO mice at P2 (Fig. 2F). We performed co-immunostaining for IBA1 and the autophagy-related protein CTSB, and observed significant signal increase in the CKO mouse brains at E17.5 and P2 (Fig. 2H and I). However, when we assessed the expression of LC3, a marker of mature autophagosomes, and evaluated the conversion of LC3I to LC3II by Western blotting to measure autophagic activity, the results showed no significant differences in the LC3II/LC3I ratio in CKO mouse brains at E17.5 and P2 (Fig. S4E and S4F). To assess whether apoptosis was also affected, we performed TUNEL (TdT-mediated dUTP Nick-End Labeling) assays, and the degree of apoptosis appeared comparable between WT and CKO brains at both stages (Fig. S4A and S4B). To further evaluate the proportion of microglia at different stages of apoptosis, we also performed Annexin-V-FITC assay followed by flow cytometry (Fig. S4C). The results revealed a higher percentage of early apoptotic microglia in E17.5 CKO mouse, and a higher percentage of late apoptotic cells in P2 CKO mouse (Fig. S4D). These results indicate that *Ccn1* deletion has a modest effect on microglial autophagy and apoptosis.

Together, these findings suggest that *Ccn1* deletion leads to the up-regulation of genes associated with autophagy and apoptosis, potentially contributing to altered microglial states.

Since many up-regulated genes were enriched in immunity-related terms based on GO analysis, we defined the microglial homeostatic and activated gene sets according to the literature (Tables S1 and S2) and analyzed the overall expression of the respective gene modules in the microglia of the WT and CKO mice.^49^, ^50^, ^51^, ^52^ The GSEA results revealed that the quiescent microglial gene module was significantly down-regulated (Fig. S3F), and that the activated gene module was markedly up-regulated (Fig. 2E) in CKO mouse brains at P2, which suggests enhanced microglial activation at this stage. In contrast, at E17.5, neither the up-regulation of the quiescent module nor the down-regulation of the activated module reached statistical significance (Fig. S3F; Fig. 2E), which may be attributed to the fact that microglial activation primarily occurs after birth. We take *Ms4a6d* and *Cd86*, two well-known microglial activation genes,^52^ as examples. In the single-cell data, we can observe that *Cd86 and Ms4a6d* were significantly elevated in CKO microglia (Fig. 2G). We further performed RT-qPCR to examine the expression levels of *Cd86* and *Ms4a6d* in microglia isolated from the WT and CKO mouse forebrains at P2. The up-regulation of *Cd8*6 was modest; the significant up-regulation of *Ms4a6d* was observed in CKO microglia at P2 (Fig. 2J).

These results suggest that *Ccn1* deletion in NSCs and astrocytes not only alters microglial abundance but also induces significant transcriptional changes in microglia. Notably, gene modules related to autophagy and apoptosis were up-regulated across both the embryonic and postnatal stages, and signs of abnormal microglial activation were particularly evident in the postnatal brain. Although the activation of autophagy- and apoptosis-related genes appears to be correlated with a mild reduction in the overall proportion of microglia, we did not observe a corresponding increase in apoptotic signals in the VZ region, where the number of microglia was mostly reduced. These findings suggest that the mechanisms underlying the altered spatiotemporal distribution of microglia remain unclear. Here, the spatial transcriptomic analysis may offer a promising approach for further exploration.

### Spatial transcriptomics reveals that specific knockout of *Ccn1* in NSPCs in the mouse brain alters microglial spatial distribution

**Figure 3:**
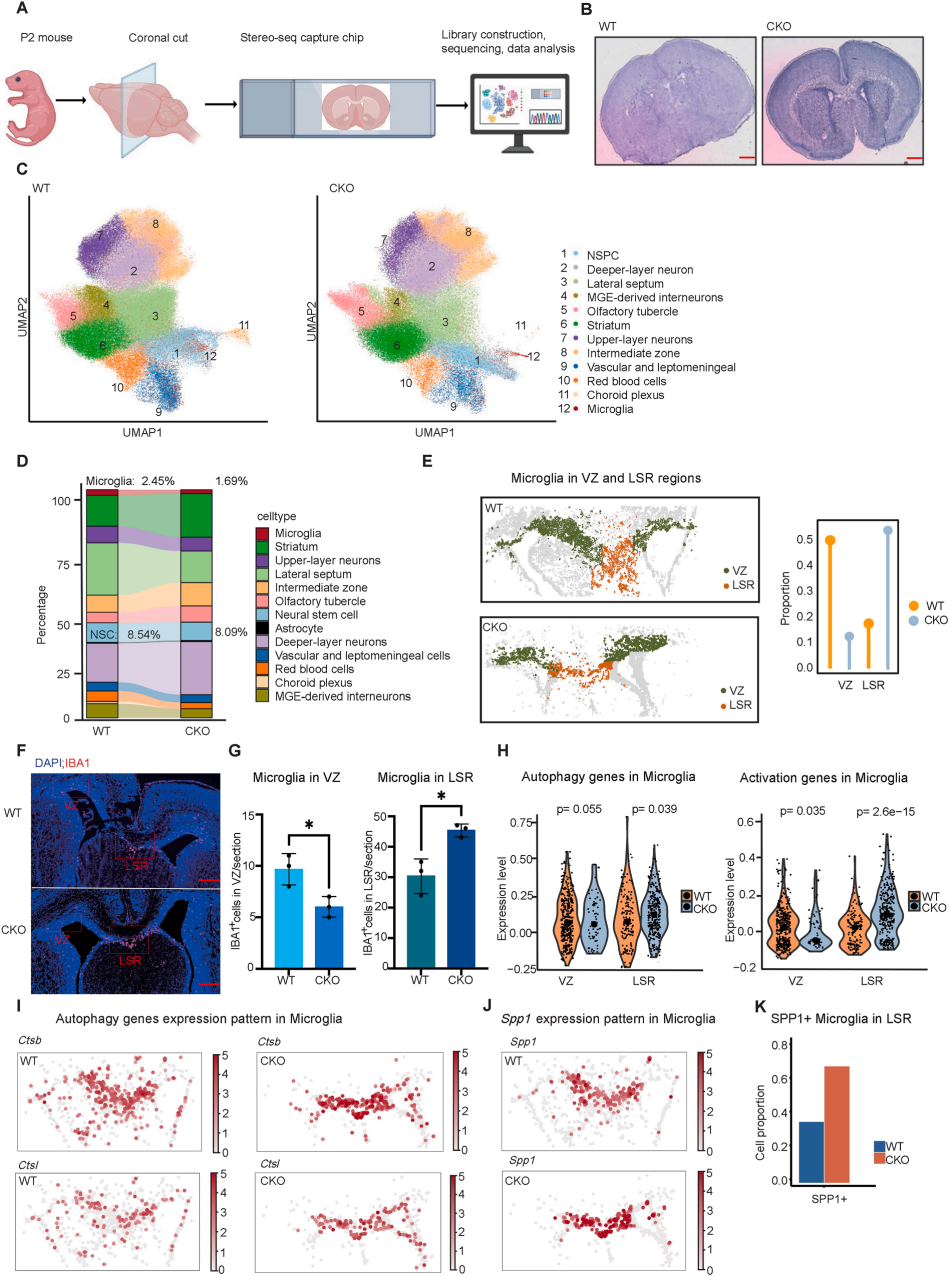
Spatial transcriptomics reveals altered microglial spatial distribution in *Ccn1* conditional knockout (CKO) mice. **(A)** Schematic diagram illustrating the generation of spatial transcriptomic data. **(B)** Hematoxylin and eosin staining of brain sections from P2 WT and CKO mice in Stereo-seq. Scale bar: 50 μm. **(C)** UMAP visualization of spatial transcriptomics profiles from P2 wild type (WT) and CKO brains, colored by annotated cell types. **(D)** Sankey plot comparing cell type composition between P2 WT and CKO brains, showing a reduced microglial ratio in CKO brains based on spatial transcriptomic data. **(E)** Spatial transcriptomic analysis showing microglial distribution primarily in the ventricular zone (VZ) and lateral sub-region (LSR), with a reduced ratio in VZ but increased ratio in LSR in CKO brains. **(F, G)** IBA1 immunofluorescence (IF) staining of microglia (F) and quantitative analysis of IBA1^+^ nuclei in VZ and LSR regions (G), confirming increased microglial numbers in LSR but decreased numbers in VZ of P2 CKO mice (*n* = 3 mice per group; VZ: *p* = 0.0254; LSR: *p* = 0.0127). Scale bar: 50 μm. **(H)** Violin plot showing regional expression changes: downregulation in VZ and upregulation in LSR of apoptotic, autophagy, and activation gene modules in CKO microglia versus WT, based on spatial transcriptomic analysis. **(I)** Spatial expression patterns of autophagy-related genes (Ctsb, Ctsl) in microglia from WT and CKO mouse brains, visualized by spatial transcriptomics. **(J, K)** Upregulation of microglial activation marker Spp1 in LSR microglia of P2 CKO mice, demonstrated by spatial expression pattern analysis (J) and quantitative analysis of its expression in microglia (K) from spatial transcriptomic data. Data information: In (G), data are presented as mean ± SEM. Unpaired Student's *t*-test (two-tailed) was used: ∗*p* < 0.05; *ns*, not significant.

To investigate the effect of specific knockout of *Ccn1* in NSCs on the CNS microenvironment, we generated spatial transcriptome (ST) libraries of two coronal sections of the P2 WT and CKO mouse brains (Fig. 3A and B; Fig. S5A). We used STAGATE,^43^ a graph attention auto-encoder framework, to precisely identify spatial domains by learning low-dimensional latent embeddings that integrate spatial data with gene expression profiles. Through integration, dimension reduction, and clustering analysis, a total of 12 clusters were obtained. Based on the expression of known marker genes, we identified them as NSPCs (*Sox2*, *Mki67*, *Eomes*), deeper-layer neurons (*Sox5*, *Tbr1*), lateral septum (*Tmem130*, *Fxyd6*), MGE-derived interneurons (*Sst*, *Npy, Lhx6*), olfactory tubercle (*Penk*, *Gng7*), striatum (*Ppp1r1b*, *Foxp1*, *Pccp4*), upper-layer neurons (*Cux1*, *Cux2*), intermediate zone (*Sox5*, *Dcx*), vascular and leptomeningeal cells (*Vtn*, *Lgals1*, *Col1a2*), red blood cells (*Hbb-bs*, *Hbb-bt*), choroid plexus (*Ttr*, *Col9a3*), and microglia (*Apoe*, *Ctss, C1qc*), which included the major cell types in the brain (Fig. 3C; Fig. S5B–S5D). We also described the unique spatial distribution of these cell clusters (Fig. S5D). Next, based on the astrocyte-specific marker genes *Aldh1l1* and *Aldoc*, we further divided the NSPC population into two subgroups: NSCs and astrocytes (Fig. S5E). We then quantified the proportion of each cell cluster in WT and CKO mouse brains and found that the microglial proportion in the brains of CKO mice was significantly reduced, and that the proportion of NSCs was mainly unchanged (Fig. 3D), which was also consistent with the analysis from the scRNA-seq results.

In the developing brain, microglia migrated from the periphery regions to the center of the brain via the bloodstream and preferentially localized in the VZ and subventricular zone (SVZ) region,^53^ where NSCs are enriched (Fig. S5F). Taking advantages of ST, we can distinguish differences in the spatial distribution of the same cell type. We therefore delineated the VZ and LSR regions according to their anatomical structures and analyzed microglial proportion in the two regions. Besides the significant decrease in the proportion of microglia in the VZ region of CKO mice (Fig. 3E), the microglial proportion was significantly increased in the LSR region of CKO mice (Fig. 3E). We then performed IBA1 immunostaining in brain sections of P2 mice, quantified the number of IBA1^+^ cells on the same area size in VZ and LSR regions, and confirmed the distribution disorder of microglia in the VZ and LSR regions between WT and CKO mice (Fig. 3F and G). We further examined the microglial transcriptomic profiles in the VZ and LSR regions of WT and CKO mice. Unlike single-cell sequencing, which can only analyze the overall changes in microglial populations and appears to show increased apoptosis, autophagy, and activation-related genes, ST data allow us to compare the expression differences of microglia in the LSR and VZ regions. We found that the up-regulation of cell apoptosis and autophagy genes occurred mainly in the microglial population in the LSR region rather than in the VZ region of the CKO mouse brain (Fig. 3H), in which the typical autophagy genes *Ctsb* and *Ctsl* both showed high aggregated expression in the LSR region (Fig. 3I). Meanwhile, the expression level of the activated gene module was also significantly elevated in the LSR region of the CKO mouse brain (Fig. 3H). The spatial expression pattern of the activation marker genes *Spp1*^52^ and *Lpl*^26^ also showed that their expression is apparently increased in the LSR region of CKO mouse brain (Fig. 3J and K; Fig. S5H and S5I).

Our results revealed that the deletion of *Ccn1* in NSPCs, mainly including NSCs and astrocytes, results in more microglia aggregating in the LSR region and fewer microglia localized in the VZ region with altered spatial expression patterns of apoptosis- and activation-associated genes. The molecular basis underlying the altered state and distribution of microglia was further investigated, which is believed to result from microenvironmental changes in the LSR region. We then performed a focused analysis to examine the interaction changes between neural progenitor cells and microglia in the LSR region upon depletion of *Ccn1*.

### *Ccn1* regulates NSC–microglia interaction by influencing the spatial expression pattern of *Csf1*/*Il2* in NSCs

The resolution of the microenvironment is mainly defined by changes in target genes, ligands, and receptors, which necessitates first identifying the key cell populations involved in its regulation. Previous single-cell RNA-seq data indicated that *Ccn1* is expressed in NSCs, astrocytes, and ependymal cells in the P2 mouse brain. In the CKO brain, *Ccn1* expression is lost in all three of these cell types, potentially impairing their capacity to regulate the local microenvironment. To define the principal cell population of interest, we first analyzed the spatial expression patterns of *Id1*, a marker highly expressed in NSCs and developing brain astrocytes, and *Foxj1*, a well-established marker of ependymal cells, in relation to the spatial distribution of the microglial marker *Ctss*. In WT mouse brains, spatial transcriptomic analysis revealed patterns consistent with prior immunostaining results: *Foxj1* expression was relatively sparse, and ependymal cells were primarily localized to the lateral ventricle, showing minimal spatial overlap with microglia (Fig. S6A). In contrast, *Id1* exhibited broader expression and was spatially adjacent to microglia, being present in both the VZ and LSR regions (Fig. S6A). These findings suggest that NSPCs, including both NSCs and astrocytes, are likely the primary contributors to the altered distribution and state of microglia observed in *Nestin*-Cre/*Ccn1*^flox/flox^ mouse brains.

**Figure 4:**
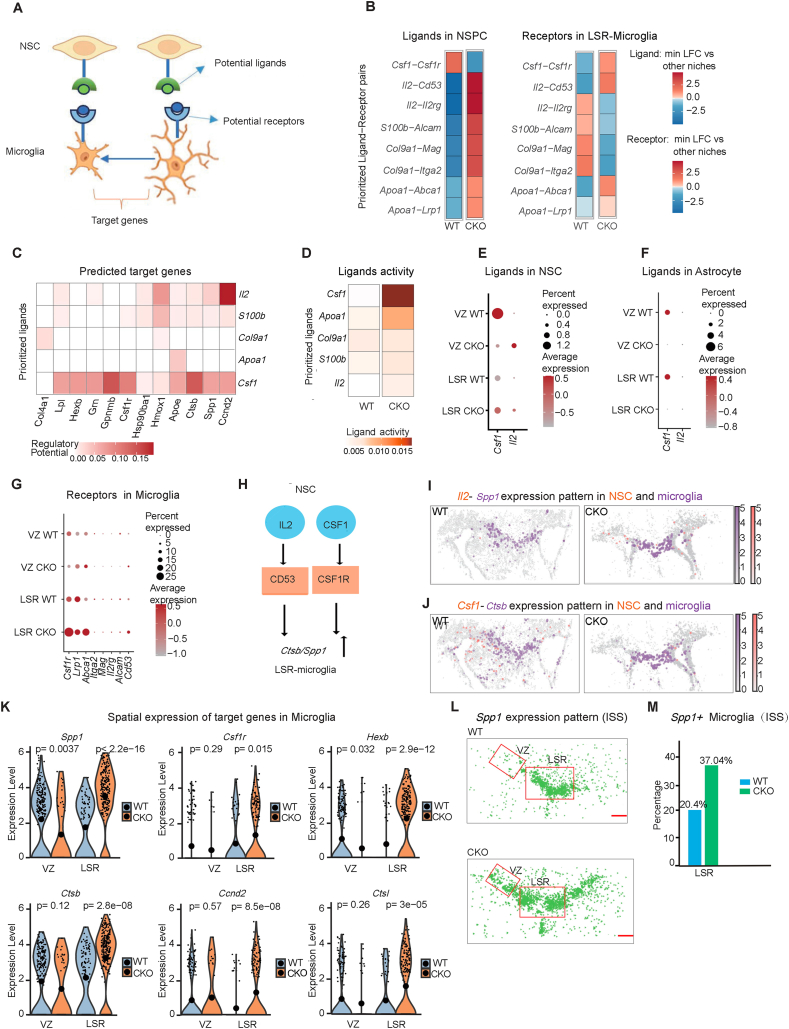
Ccn1 regulates the spatial expression of Csf1 and Il2 to influence NSC-microglia interactions. **(A)** Overview of the NicheNet analysis framework for investigating NSC-microglia interactions. **(B)** Prioritized ligand–receptor pairs from neural stem progenitor cells (NSPCs) to LSR microglia in wild type (WT) versus conditional knockout (CKO). Heatmaps display condition-specific min log_2_ FC of NSPC ligands (left) and lateral sub-region (LSR) microglia receptors (right) relative to the opposite niche. Red indicates higher specificity; blue indicates lower specificity (NicheNet analysis). **(C)** Heatmaps showing the regulatory potential of prioritized ligands in NSPCs and their predicted target genes in LSR microglia, based on spatial transcriptomic data (NicheNet analysis). **(D)** Ligand activity analysis of prioritized ligands in LSR microglia from spatial transcriptomic data (NicheNet analysis). **(E)** Normalized expression levels of Csf1 and Il2 in NSPCs located in ventricular zone (VZ) and LSR regions of WT and CKO mice, revealed by spatial transcriptomic analysis. **(F)** Normalized spatial expression of Csf1 and Il2 in astrocytes located in VZ and LSR regions of WT and CKO mice, based on spatial transcriptomic analysis. **(G)** Normalized spatial expression of corresponding receptors in microglia at VZ and LSR regions, analyzed from spatial transcriptomic data. **(H)** Proposed regulatory mechanisms involving the Il2-Cd25 and Csf1-Csf1r pathways and their target genes. Il2 positively regulates the downstream ligand Cd53, enhancing expression of target genes (e.g., *Ctsb*, *Spp1*). Csf1 positively regulates its receptor Csf1r, promoting expression of target genes (e.g., *Ctsb*, *Spp1*). **(I)** Spatial co-expression patterns of Il2 (ligand) and Spp1 (target) in NSPCs and microglia in WT and CKO brains (spatial transcriptomic analysis). **(J)** Spatial co-expression patterns of Csf1 (ligand) and Ctsb (target) in NSPCs and microglia in WT and CKO brains (spatial transcriptomic analysis). **(K)** Expression levels of representative target genes in microglia from VZ and LSR regions of WT versus CKO mice (spatial transcriptomic analysis). **(L, M)** Upregulation of Spp1 in LSR microglia of P2 CKO mice was confirmed by *in situ* sequencing (ISS) signal (L) and statistical analysis (M).

To investigate the impact of the microenvironment on microglia through *Ccn1*-deficient NSPCs, we used the NicheNet tool, an algorithm designed to predict ligand–receptor interactions that drive changes in gene expression, which integrates transcriptomic data from interacting cells with established information on signaling pathways and gene regulatory networks.^54^ We first applied NicheNet to predict which ligand–receptor interactions could potentially induce the differentially expressed genes identified in microglia with distinct spatial preferences in the LSR and VZ regions (Fig. 4A). We then inferred the ligand–receptor and target gene regulatory interaction networks between NSPCs and microglia identified in the ST data (Fig. 4B). Finally, we identified ligand–receptor pairs that may play important regulatory roles in the spatial distribution and activation of microglia (Fig. 4B–D; Fig. S6D and S6E), and further focused on two specific pairs, *Csf1*–*Csf1r* and *Il2*–*Cd53*, which exhibited positive and negative correlations, respectively, between NSPCs and microglia. Previous studies have demonstrated that the spatiotemporal localization pattern of microglia is associated with *Csf1* expression, while *Il2* has been implicated in modulating their activation state. Consistent with these reports, we found that many downstream target genes of the *Csf1*–*Csf1r* and *Il2*–*Cd53* axes were strongly associated with microglial migration and activation, and were up-regulated in LSR microglia from CKO brains (Fig. 4C; Fig. S6B). The activation of these downstream targets coincided with increased expression of the receptors *Csf1r* and *Cd53* in microglia, as well as elevated expression of the upstream ligand *Il2*. Interestingly, however, *Csf1* was not up-regulated in NSPCs. Instead, it was down-regulated (Fig. S6C), which is unexpected and suggests a more complex regulatory mechanism that warrants further investigation.

Given that the NSPC population comprises both NSCs and astrocytes, and that cells of the same type may exhibit region-specific expression patterns, we further analyzed the spatial expression of *Csf1* and *Il2*, separately examining their distribution in NSCs and astrocytes (Fig. 4E and F). In NSCs, *Csf1* expression was down-regulated in the VZ region, consistent with the overall reduction in expression. In contrast, it was significantly up-regulated in the LSR region, aligning with the regional activation of its receptor *Csf1r* in microglia: up-regulated in the LSR and down-regulated in the VZ (Fig. 4E–H; Fig. S6E). Similarly, *Il2* expression was markedly increased in both the VZ and LSR regions, corresponding with the activation of its downstream receptor *Cd53* in microglia across both regions (Fig. 4E–H; Fig. S6E). However, in astrocytes, *Csf1* expression was down-regulated in both regions of CKO brains, and *Il2* was virtually undetectable, suggesting that astrocytes are unlikely to be the primary source driving the altered ligand–receptor interactions described above.

We further confirmed that the target genes of these two pairs of ligand-receptors (e.g., *Spp1, Hexb, Csf1r, Ccnd2, Ctsb,* and *Ctsl*) were significantly up-regulated in LSR microglia in the CKO mouse brains (Fig. 4I–K). Among these genes, *Csf1r* has been previously implicated in regulating microglial migration^55^^,^^56^ *Ctsb* and *Ctsl*, as important lysosomal proteases, can regulate protein degradation to promote cell autophagy^57^; *Spp1* and *Hexb* are known to be microglial activation marker genes.^52^^,^^58^ These results are also consistent with our observation that microglia enhance autophagy and hyperactivation in the LSR region.

To further validate the altered expression patterns of target genes in the brains of CKO mice, we performed *in situ* sequencing (ISS) on coronal brain sections from WT and CKO mice at postnatal day 2 (P2) (see Methods). This technique enables direct detection of RNA transcripts within tissue sections while preserving spatial context. In this experiment, we focused on assessing the spatiotemporal expression patterns of *Csf1, Il2, Csf1r, Cd53,* and *Spp1* (Fig. S6F). Consistent with our sequencing results, the ISS data showed that *Csf1r* expression was increased in the LSR region in CKO brains (Figs. S6E, S6G, S6H). Additionally, the expression of *Spp1* and *Lpl*, markers of microglial activation, was significantly up-regulated in the LSR region (Fig. 4L and M), further supporting our previous findings (Fig. 3J and K).

In summary, our results suggest that *Ccn1* deficiency in NSPCs triggers widespread up-regulation of *Il2* in NSCs, along with regional activation of *Csf1* in the LSR and suppression in the VZ. The interactions between NSCs and microglia, mediated through the CSF1–CSF1R and IL2–CD53 axes, lead to transcriptional alterations and abnormal spatial distribution of microglia.

**Figure 5:**
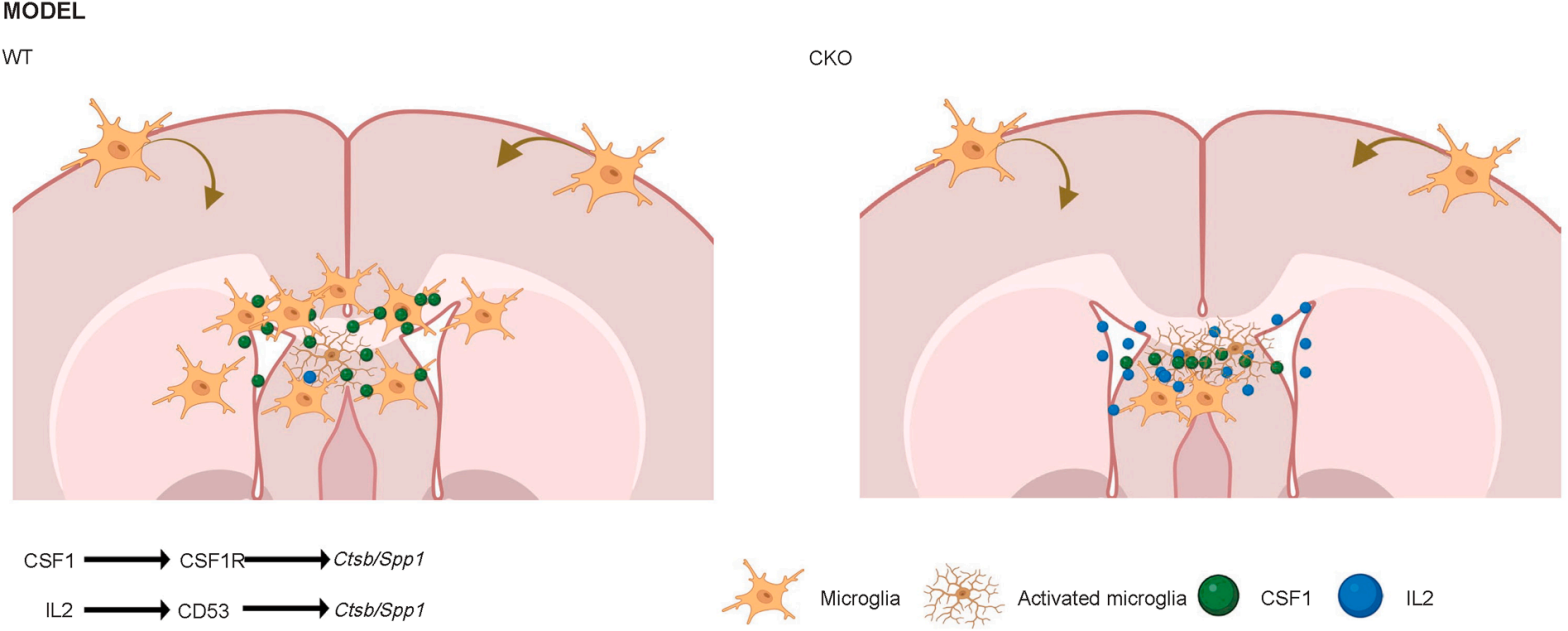
Ccn 1 plays as a “balance-keeper” for region-specific NSCs-microglia interaction.

Specifically, the reduced secretion of CSF1 by NSCs in the VZ and its accumulation in the LSR could be sensed by microglia via *Csf1r*, promoting their migration toward the LSR and resulting in a depletion of microglia in the VZ. Meanwhile, *Ccn1* knockout in NSCs led to increased *Il2* expression, particularly in the LSR, which in turn caused microglial overactivation and enhanced autophagy in this region (Fig. 5).

## Discussion

In this study, by applying spatial transcriptomics, we demonstrated the crucial role of *Ccn1* in maintaining the spatiotemporal expression pattern of key regulatory ligands, such as CSF1 and IL2, in NSCs. Specifically, the deletion of *Ccn1* caused a significant reduction of *Csf1* in NSCs in the VZ and a marked increase in the LSR region, altering microglial migration preferences through *Csf1r* and downstream target activation in microglia. Additionally, the IL2-CD53 pair showed an overall increase in NSCs and microglia. The activation of these ligand–receptor pairs triggered increased expression of target genes like *Spp1*, *Hexb*, *Csf1r*, *Ccnd2*, *Ctsb*, and *Ctsl* in microglial particularly within the LSR region, thereby possibly promoting microglial activation and autophagy. Given that *Ccn1* deficiency was also detected in astrocytes, we examined changes in the expression of *Csf1* and *Il2* in astrocytes. While *Csf1* was found to be down-regulated, *Il2* was barely expressed. Moreover, there was no significant difference in Il2 expression between WT and CKO astrocytes, suggesting that *Ccn1* deficiency in astrocytes is unlikely to contribute to the observed defects in microglial distribution and activation. Although *Csf1* expression was reduced in the astrocytes of CKO mice, this decrease lacked the region-specific features observed in NSCs. Together, our findings demonstrate that *Ccn1* modulates NSC–microglia interactions by regulating the spatiotemporal expression of key microenvironmental ligands within NSCs. These changes subsequently affect microglial migration, gene expression, and spatial distribution, thereby playing a crucial role in maintaining central nervous system microenvironmental homeostasis.

In this context, we utilized previously published single-cell RNA-seq data and immunostaining experiments to demonstrate that *Ccn1* is expressed in NSPCs, including NSCs, astrocytes, and ependymal cells, during embryonic and early postnatal development, and may play roles in regulating brain development. Here, we used *Nestin*-Cre mice, which is a widely used genetic tool for specifically targeting NSCs and their progeny in the CNS,^40^ to disrupt the expression of *Ccn1* in NSPCs. Although the *Nestin*-Cre system contains mixed background expression and is not entirely specific to NSCs, we proved that the main knockout effects are still limited to NSCs and its differentiation progeny astrocyte at E17.5 and P2 using the single-cell RNA-seq data, and further excluded the impact of astrocytes on spatial dysfunction of *Csf1* and *Il2* using the ST data, reinforcing that the observed phenotypes are primarily attributable to NSC-specific deletion of *Ccn1*. These findings also highlight the potential of emerging multi-omics approaches, such as spatial transcriptomics and single-cell sequencing, in uncovering the cellular mechanisms underlying developmental processes.

As a secreted protein, CCN1 binds tightly to the extracellular matrix and cell surface with non-covalent bonds and then regulates cellular biological activities by binding to different integrin isoforms.^34^^,^^59^^,^^60^ *Ccn1* has been reported to regulate the proliferation and differentiation of neural stem cells during the embryonic and adult stages and is correlated with the progression of glioblastoma through the EGFR signaling pathway.^38^^,^^60^ This function is likely induced by its intracellular rather than its extracellular role. Interestingly, our findings also suggest that *Ccn1* may not exert its effects on microglia primarily through direct extracellular signaling. This conclusion is supported by our analysis of NSPC–microglia interactions, which revealed that the most prominent changes occurred in the *Csf1*–*Csf1r* and *Il2*–*Cd53* signaling axes and their downstream target genes in microglia—corresponding with alterations in microglial distribution, number, and activation state. Also, we checked the expression of YAP downstream genes, which were reported to be regulated by *Ccn1*,^60^ and found few changes in microglia (Fig. S6I). Therefore, we propose that the primary role of *Ccn1* in regulating microglial recruitment and activation is to orchestrate the temporal and spatial expression patterns of key ligands in NSCs rather than acting directly on microglia. However, it remains unclear how *Ccn1* regulates key ligands in neural stem cells (NSCs). Previously, in tumor research, *Ccn1* was found to regulate integrins, growth factors, Wnt, NF-kappaB, or tyrosine kinase signaling pathways intracellularly.^61^^,^^62^ Whether the regulatory mechanism in NSCs is similar may require further exploration in the future. Meanwhile, our study does not exclude the possibility that the extracellular functions of CCN1 may also play important roles in brain development. In fact, both our single-cell and spatial transcriptomic data revealed abnormalities in neuronal differentiation and distribution. Thus, future studies exploring the extracellular regulatory roles of CCN1 during brain development will be essential to fully elucidate its multifaceted functions. Therefore, in the study of *Ccn1* during brain development, its extracellular regulatory functions remain an important direction for future investigations.

Another important aspect of our work is demonstrating the unique advantages of spatial transcriptomics in studying the regulation of microenvironment homeostasis in the brain. Unlike traditional research approaches, in our study, by spatially zoning NSCs and microglia in the VZ and LSR regions, we were able to effectively capture the spatial heterogeneity of the same cell type in different regions, an important aspect that has been less addressed in previous studies. Using high-resolution Stereo-seq technology,^63^ we were not only able to accurately identify the different transcriptomic profiles of the same cell population in specific regions of the brain, but also able to analyze in detail the cellular interactions between regions, which is important for an in-depth understanding of the cellular changes in the functional state of different spatial microenvironments.

Our data in this study suggest that *Ccn1* function as the “balance-keeper” between NSCs and microglia in the microenvironment, which effectively maintains the spatial localization of NSCs and microglia, enabling them to perform their respective roles and ensure the homeostatic balance of the CNS microenvironment. When *Ccn1* was knocked out in NSCs, this homeostatic “wall” was disrupted, resulting in excessive microglia migrating to the LSR region instead of the VZ region and over-activation of microglia, which enhanced microglial autophagy and immune function. This finding provides a new understanding of NSC–microglia interactions and highlights the important role of the spatial microenvironment in regulating cellular function. Meanwhile, this model may also apply to other cell interactions or serve as a reference for other multicellular systems, which warrants further exploration.

## CRediT authorship contribution statement

**Zhile Bai:** Writing – original draft, Project administration, Investigation. **Zhanhe Chang:** Writing – original draft, Project administration, Formal analysis. **Shuguang Wang:** Formal analysis. **Jiangli Zheng:** Project administration. **Huan Wu:** Project administration. **Hanyu Liu:** Project administration. **Yalin Zhang:** Project administration. **Jiayang Xie:** Project administration. **Qingran Bai:** Project administration. **Jiqing Yin:** Project administration. **You Wu:** Supervision. **Qin Shen:** Conceptualization. **Shaorong Gao:** Conceptualization. **Yawei Gao:** Writing – review & editing, Conceptualization.

## Data availability

The single-cell multi-omics datasets generated and analyzed in this study have been deposited in the OMIX database at the National Genomics Data Center (NGDC), National Bioinformation Center, China National Center for Bioinformation (CNCB), under accession number OMIX011972. The datasets are publicly accessible via https://ngdc.cncb.ac.cn/omix/release/OMIX011972.

## Funding

This work was primarily supported by the 10.13039/501100012166National Key R&D Program of China (No. 2024YFA1107000 to Y.G., 2021YFC2700301 and 2022YFC2702200 to S.G.), the 10.13039/501100001809National Natural Science Foundation of China (No. 32370869 to Y.G., 92168205 and 32330030 to S.G., 32030047 to Q. S.), the Shanghai Pilot Program for Basic Research (China) (to Y.G.), and the Peak Disciplines (type IV) of Institutions of Higher Learning in Shanghai (China) (to S.G.).

## Conflict of interests

The authors declare that they have no conflict of interests.
