# Supplementary material for: Spatial transcriptomics revealed the regulatory role of CCN1 to microglia distribution through region-specific cellular interactions
Source: Genes Dis. 2025 Dec 9;13(6):101969. doi: 10.1016/j.gendis.2025.101969 (PMC13486995; doi:10.1016/j.gendis.2025.101969)
Supplement: Supplementary file 1 [file mmc1.docx]

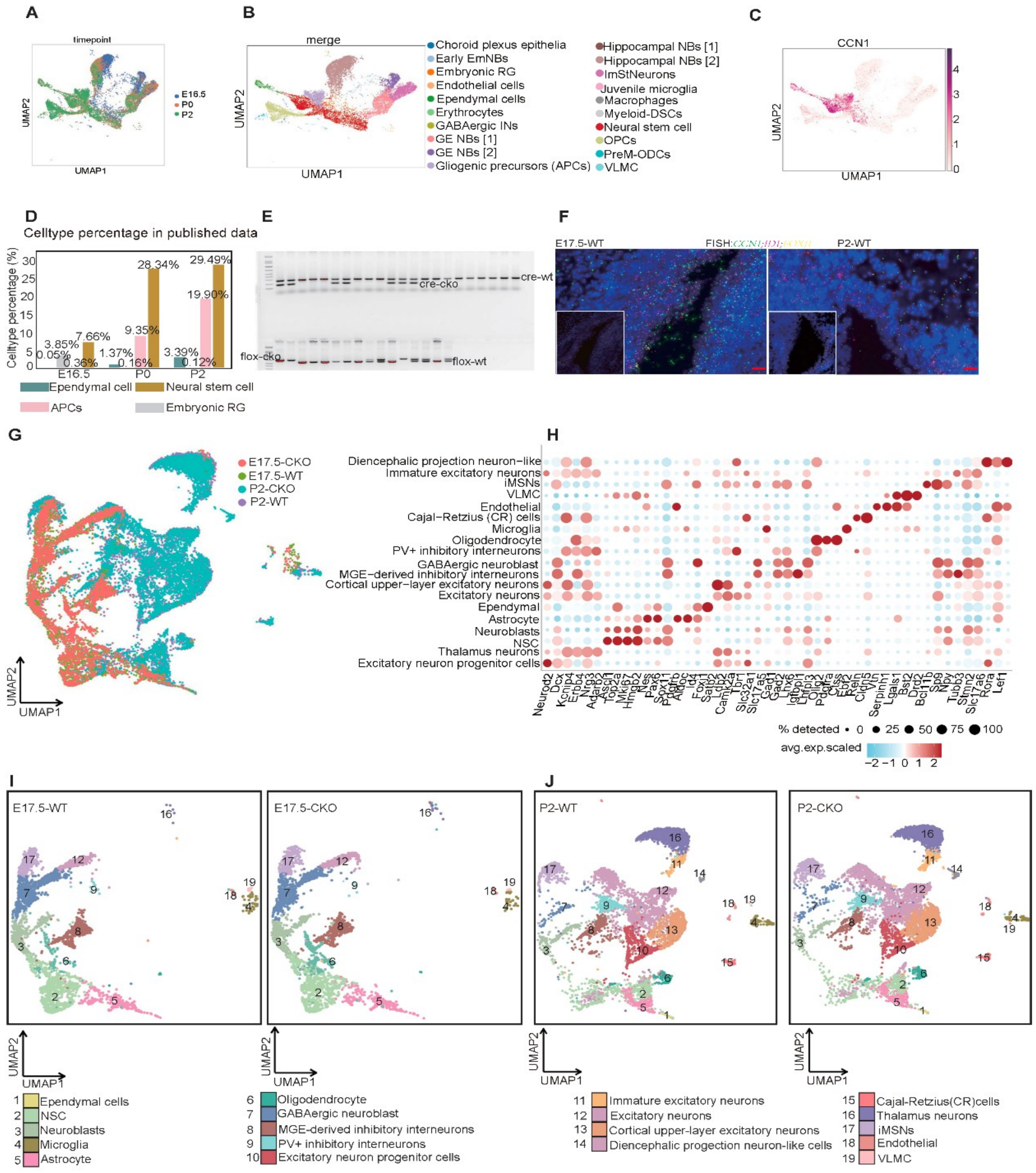
A
timepoint
E16.5
P0
P2
UMAP1
UMAP2
B
merge
Choroid plexus epithelia
Early EmNBs
Embryonic RG
Endothelial cells
Ependymal cells
Erythrocytes
GABAergic INs
GE NBs [1]
GE NBs [2]
Gliogenic precursors (APCs)
Hippocampal NBs [1]
Hippocampal NBs [2]
ImStNeurons
Juvenile microglia
Macrophages
Myeloid-DSCs
Neural stem cell
OPCs
PreM-ODCs
VLMC
C
CCN1
D
Celltype percentage in published data
Celltype percentage (%)
E16.5
P0
P2
Ependymal cell
Neural stem cell
APCs
Embryonic RG
E
cre-wt
cre-cko
flox-cko
flox-wt
F
E17.5-WT
P2-WT
G
E17.5-CKO
E17.5-WT
P2-CKO
P2-WT
H
Diencephalic projection neuron-like
Immature excitatory neurons
iMSNs
VLMC
Endothelial
Cajal-Retzius (CR) cells
Microglia
Oligodendrocyte
PV+ inhibitory interneurons
GABAergic neuroblast
MGE-derived inhibitory interneurons
Cortical upper-layer excitatory neurons
Excitatory neurons
Ependymal
Astrocyte
Neuroblasts
NSC
Thalamus neurons
Excitatory neuron progenitor cells
% detected
avg.exp.scaled
I
E17.5-WT
E17.5-CKO
J
P2-WT
P2-CKO
1 Ependymal cells
2 NSC
3 Neuroblasts
4 Microglia
5 Astrocyte
6 Oligodendrocyte
7 GABAergic neuroblast
8 MGE-derived inhibitory interneurons
9 PV+ inhibitory interneurons
10 Excitatory neuron progenitor cells
11 Immature excitatory neurons
12 Excitatory neurons
13 Cortical upper-layer excitatory neurons
14 Diencephalic progection neuron-like cells
15 Cajal-Retzius(CR)cells
16 Thalamus neurons
17 iMSNs
18 Endothelial
19 VLMC

Figure S1

Figure S1. The expression specifitity of *Ccn1* in NSPCs.

A. Integrative UMAP plot from published data. Each dot represents a single cell and colors correspond to the distinct timepoint from E16.5 to P2.

B. UMAP plot with cell clustering from published data. Each dot represents a single cell and colors correspond to the distinct cell types.

C. UMAP plot of the cells as in **(A)** but colored by *Ccn1* expression. Each dot represents a single cell and the colors correspond to the expression levels.

D. Cell percentage of Ependymal cell, Neural stem cell (NSC), APCs and Embryonic RG of the mice brain in E16.5, P0 and P2 in published data.

E. Genotype of *Nestin*-cre; *Ccn1*$^{flox/flox}$ mice

F. The mRNA in situ hybridization confirmed the expression specifitity of CCN1 in NSPCs in E17.5 and P2 mouse brain section.

Ccn1(green), *Id1*(red), and *Foxj1*(yellow) and Scale bar, 20 μm.

G. Integrative UMAP of scRNA-seq data of WT and CKO brains in E17.5 and P2.

H. Dot plot showing the cell type markers expression across scRNA-seq clusters in WT and CKO mice brains in E17.5 and P2.

I-J. UMAP of E17.5 **(I)** and P2 **(J)** WT and CKO scRNA-seq colored by clusters.

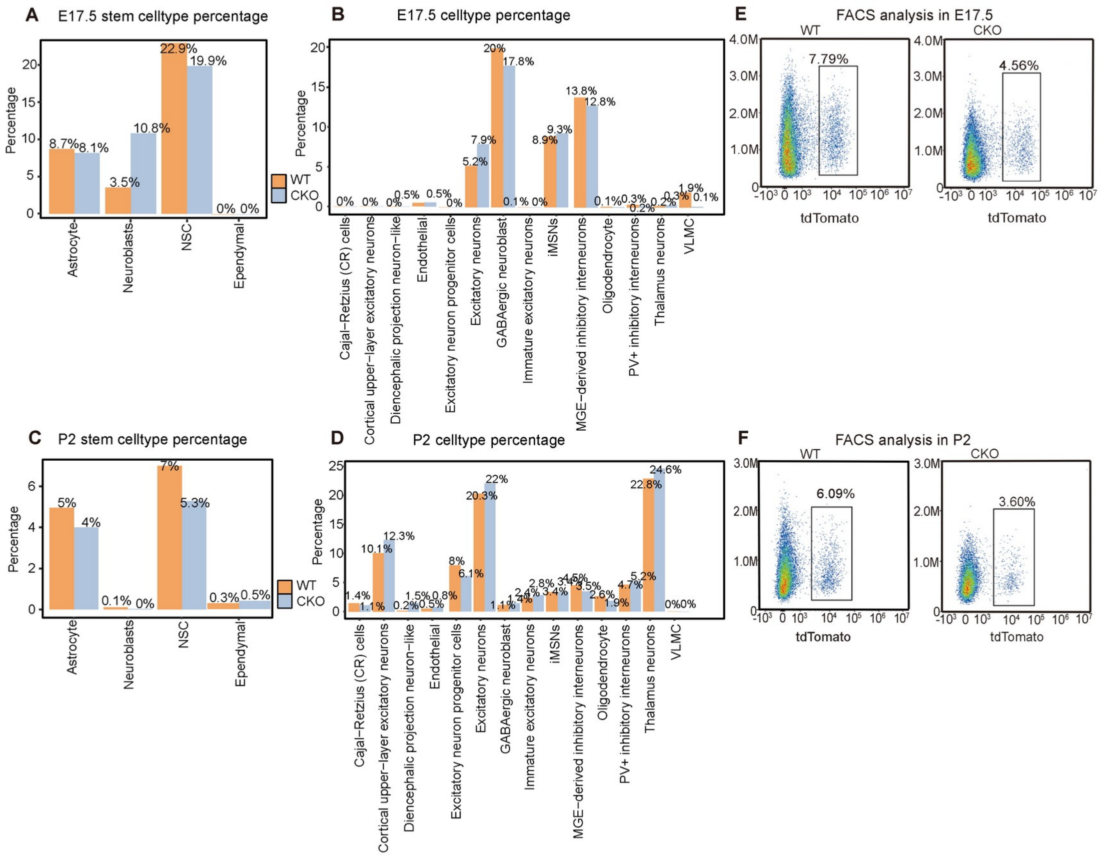

Figure S2. Deletion of *Ccn1* in NSPC induced alterations in celltype percentage in mouse brain.

A. Celltype percentage of Stem cells in WT and CKO mice brain in E17.5 defined by scRNA-seq cells.

B. Celltype percentage of other cells in WT and CKO mice brain in E17.5 defined by scRNA-seq.

C. Celltype percentage of Stem cells in WT and CKO mice brain in P2 defined by scRNA-seq cells.

D. Celltype percentage of other cells in WT and CKO mice brain in P2 defined by scRNA-seq.

E-F. FACS analysis of primary tdTomato-microglia isolated from the brains of E17.5 **(E)** /P2 **(F)** WT and CKO mice.

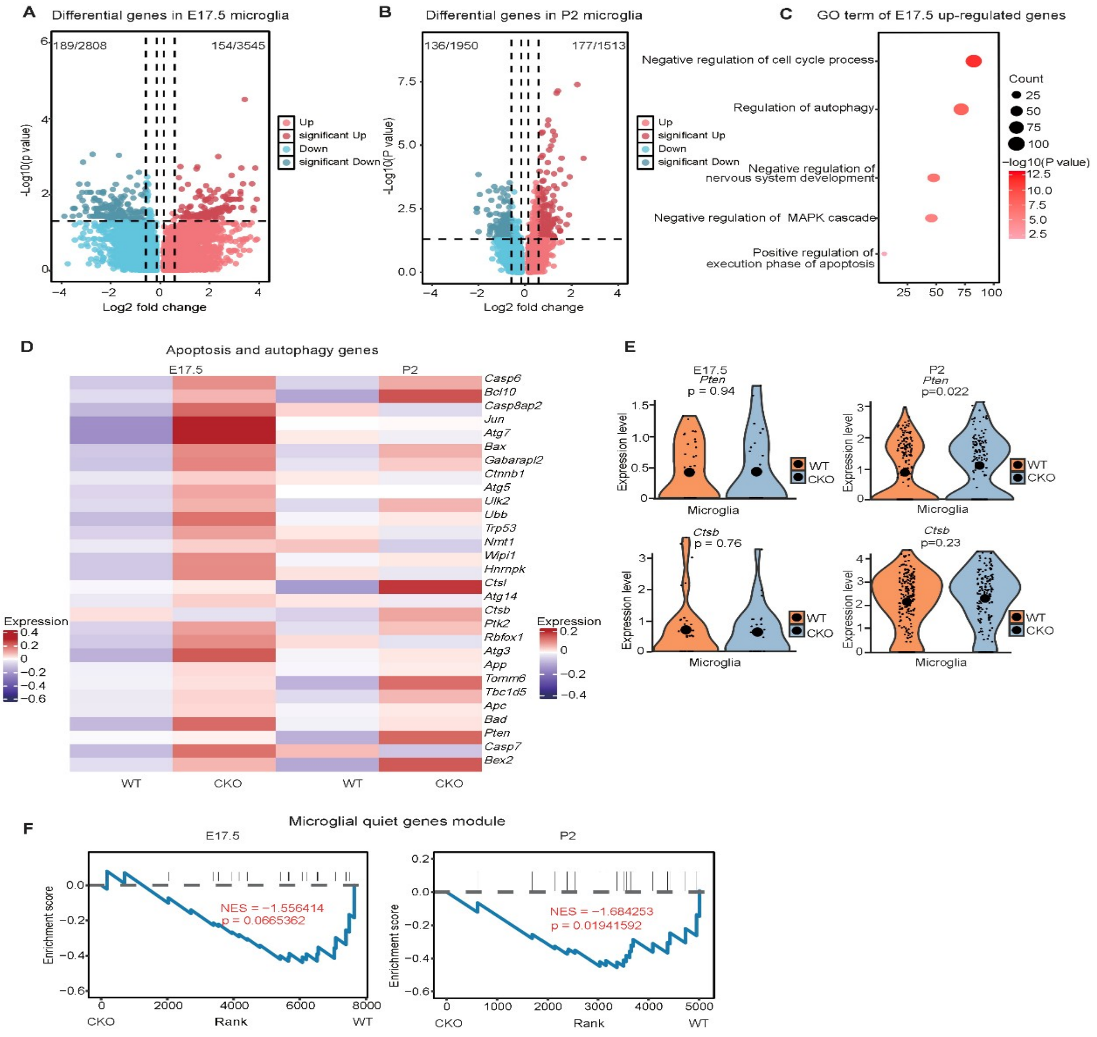

Figure S3

Figure S3. Deletion of *Ccn1* in NSPC induced transcriptional alterations in microglia.

A-B. Volcano plot showing differential expression genes in E17.5 microglia **(A)** and P2 microglia **(B)** cluster upon *Ccn1* CKO (min.pct = 0.15, logfc.threshold = 0.15). The genes number of WT and CKO in E17.5 microglia, differential up /all up genes: 154/3545, differential down /all down genes: 189/2808. The genes number of WT and CKO in P2 microglia, differential up /all up genes: 177/1513, differential down /all down genes: 136/1950. Differential cutoff of |log2FC| ≥ 0.58 (~1.5-fold change) and adjusted $p < 0.05$.

C. GO term analysis of microglial up-regulated genes in E17.5 CKO mice.

D. Heatmap of the cell apoptosis and autophagy genes scaled expression in E17.5 and P2.

E. Violin plot visualizing cell apoptotic gene *Pten* and autophagy gene *Ctsb* in WT vs CKO microglia in E17.5 and P2.

F. GSEA analysis demonstrates the global downregulation of microglial quiet genes (NES = -1.556; P-value =0.066) upon *Ccn1* CKO in E17.5 microglia single cell clusters; and global downregulation of quiet genes (NES = -1.68; P-value = 0.019) upon *Ccn1* CKO in P2 microglia single cell clusters.

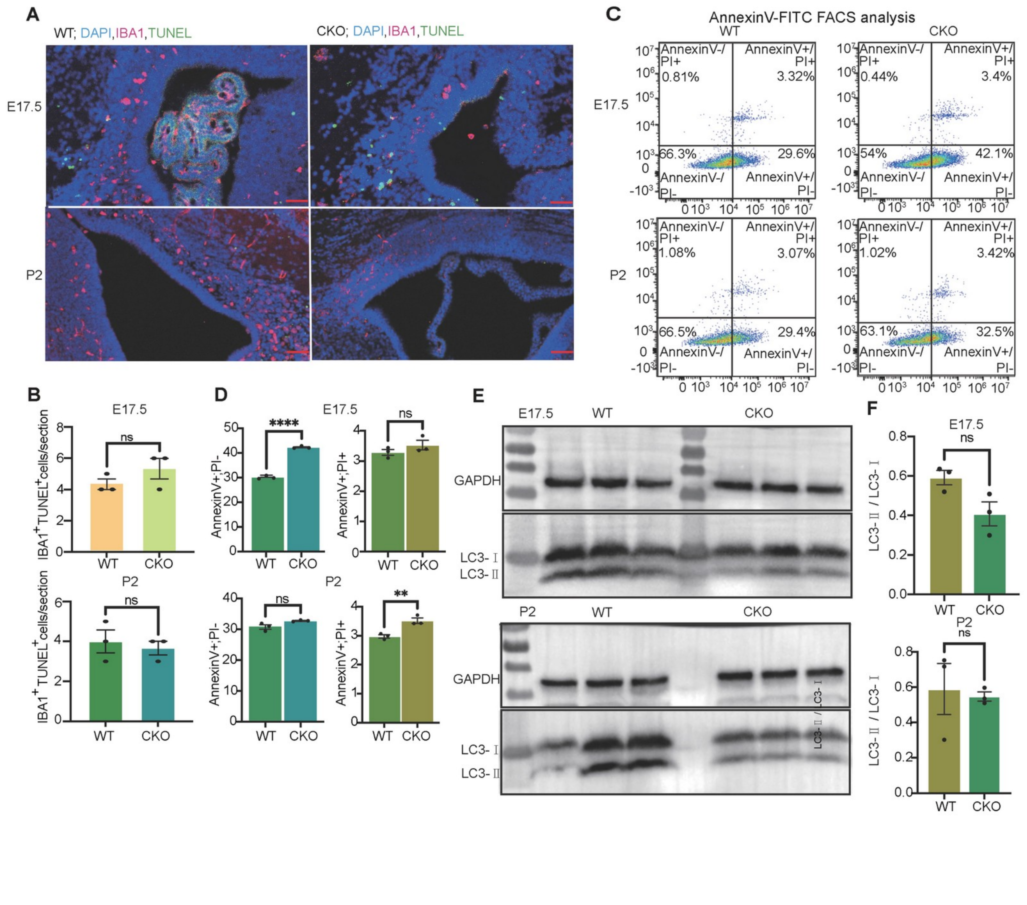

Figure S4. Deletion of *Ccn1* in NSPC induced a modest effect on microglial autophagy and apoptosis.

A-B. Microglial apoptosis detection (TUNEL Apoptosis Detection Kit (FITC)) combined with IBA1 immunostaining in E17.5/ P2 mouse brain sections **(A)** and statistical analysis for IBA1+/FITC+ nuclear **(B)** (N=3, E17.5, P=0.2508; P2, P=0.6433). (Scale bar, 50 μm.)

C. Cell apoptosis detected by flow cytometry. Cells were stained with IBA1 and Alexa Fluor 647, then Annexin V-FITC staining was performed. We analyzed the staining conditions of Annexin V-FITC and PI in microglia. Annexin V-FITC single positive cells were early apoptosis cells, Annexin V-FITC and PI double positive cells were late apoptosis cells, and PI single positive cells were naked nuclei.

D. Statistical analysis of Annexin V-FITC+; PI$^-$ and Annexin V-FITC+; PI+ cells in microglia in C (N=3), Annexin V-FITC+; PI-: E17.5, P<0.0001, P2=0.055; Annexin V-FITC+; PI+: E17.5, P=0.2583, P2=0.0084)

E-F. The expression of LC3, detected by western blotting **(E)**, and statistical analysis of LC3II/ LC3I **(F)** (N=3, E17.5, P=0.0602; P2, P=0.7836), protein derived from the brains of E17.5 and P2 WT and CKO mouse brains.

Data information: In **B, D, F,** data are presented as mean±SEM. Unpaired Student's t-test. Two-tailed t test: **P < 0.01; ***, P<0.0001, n.s., not significant.

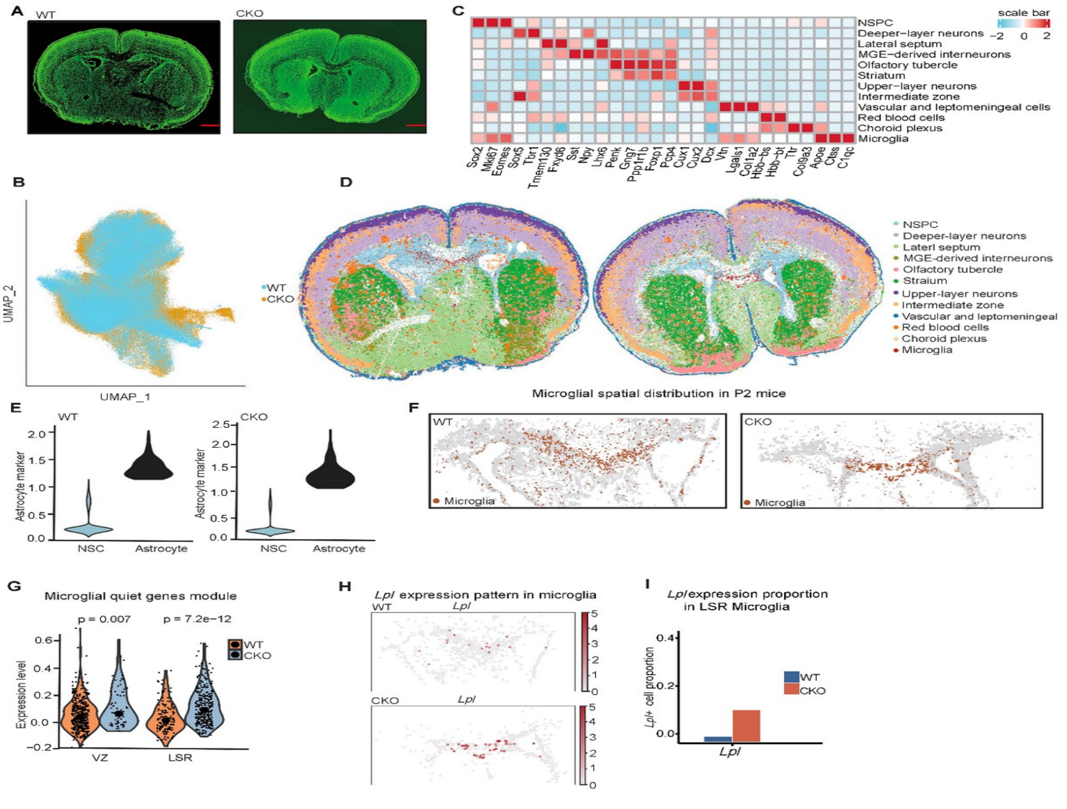

Figure S5

Figure S5. Spatial transcriptomics reveals the alters microglial spatial distribution in *Ccn1* CKO mice.

A. ssDNA staining of the brain sections of P2 WT and CKO mice in Stereo-seq. (Scale bar, 50 μm.)

B. Integrative UMAP of P2 WT and CKO Stereo-seq data.

C. Heatmap of cell type-specific genes to identify cell type clusters.

D. The spatial distribution of cell type clusters of P2 WT and CKO by spatial transcriptomic analysis.

E. Violin plot visualizing the astrocyte marker genes in NSC and Astrocyte to distinguishing NSC and astrocytes in P2 WT and CKO.

F. Microglial spatial distribution in P2 WT and CKO by spatial transcriptomic analysis.

G. Violin plot visualizing the microglial quiet genes module in P2 WT vs CKO microglia in VZ and LSR regions by spatial transcriptomic analysis.

H-I. Microglial activation marker *Lpl* was upregulated in LSR microglia in P2 CKO mice, confirmed with the spatial expression pattern analysis **(H)** and statistical analysis for its expression in microglia **(I)** by spatial transcriptomic analysis.

Data information: in **D-I**, data were form Stereo-seq data analysis.

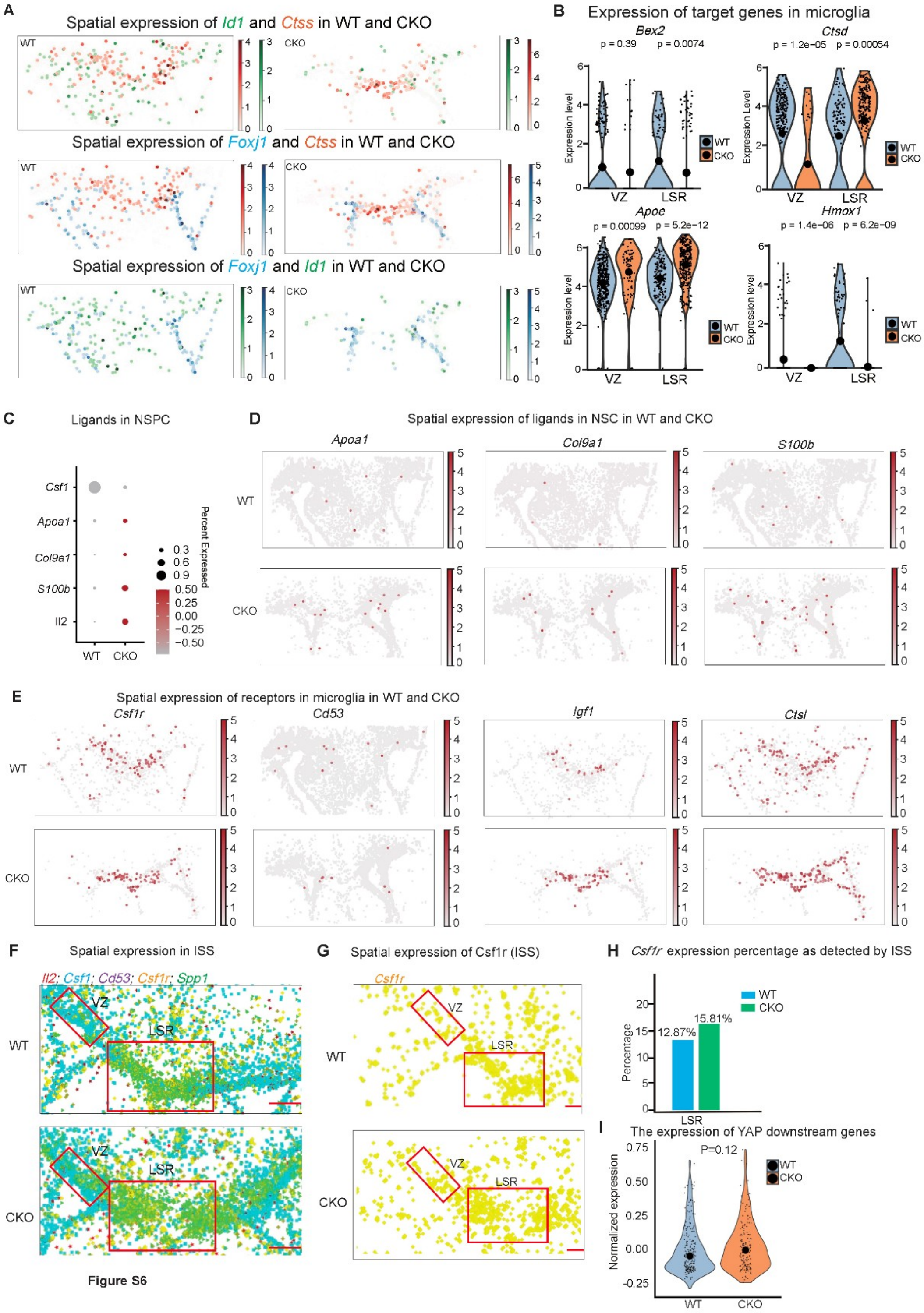
A
Spatial expression of Id1 and Ctss in WT and CKO
Spatial expression of Foxj1 and Ctss in WT and CKO
Spatial expression of Foxj1 and Id1 in WT and CKO
WT
CKO
B
Expression of target genes in microglia
Bex2
p = 0.39 p = 0.0074
Ctsd
p = 1.2e−05 p = 0.00054
Apoe
p = 0.00099 p = 5.2e−12
Hmox1
p = 1.4e−06 p = 6.2e−09
Expression level
VZ
LSR
WT
CKO
C
Ligands in NSPC
Csf1
Apoa1
Col9a1
S100b
Il2
WT CKO
Percent Expressed
0.3
0.6
0.9
0.50
0.25
0.00
−0.25
−0.50
D
Spatial expression of ligands in NSC in WT and CKO
Apoa1
Col9a1
S100b
E
Spatial expression of receptors in microglia in WT and CKO
Csf1r
Cd53
Igf1
Ctsl
F
Spatial expression in ISS
Il2; Csf1; Cd53; Csf1r; Spp1
G
Spatial expression of Csf1r (ISS)
Csf1r
H
Csf1r expression percentage as detected by ISS
Percentage
12.87%
15.81%
LSR
I
The expression of YAP downstream genes
P=0.12
Normalized expression

Figure S6

Figure S6. *Ccn1* regulates the spatial expression of *Csf1/Il2* to influence NSC-microglia interaction.

A. The spatial distribution of NSCs marker gene *Id1*, Ependymal cells marker gene *Foxj1*, and Microglial marker gene *Ctss* in spatial transcriptomic data.

B. Violin plot visualizing the expression of other target genes examples (*Bex2, Ctsd, Apoe, Hmox1*) of ligands *Csf1* and *Il2* in VZ and LSR microglia in WT and CKO mice.

C. The normalized expression levels of other predicted ligands in NSPC in WT and CKO mice.

D. The spatial expression patterns of other predicted ligands in NSC in WT and CKO mice.

E. The expression spatial patterns of other predicted receptors in microglia in WT and CKO mice

F. Expression patterns of ligands-receptors and target genes (*Il2, Csf1, Cd53, Csf1r, Spp1*) in coronal sections of P2 WT/CKO as detected using ISS, illustrated by pseudo-colors. (scale bar, 100 μm).

G-H. Expression patterns of the *Csf1r* in coronal sections of WT/CKO mouse brains at P2 as detected using ISS **(G)** and statistical analysis for its expression levels **(H)** using ISS data. (scale bar, 100 μm).

I. The expression of YAP downstream genes in microglia in P2 WT and CKO scRNA-seq data.

Data information: in **A-E**, data were form Stereo-seq data analysis.
