# Supplementary material for: Spatial transcriptomics revealed the regulatory role of CCN1 to microglia distribution through region-specific cellular interactions
Source: Genes Dis. 2025 Dec 9;13(6):101969. doi: 10.1016/j.gendis.2025.101969 (PMC13486995; doi:10.1016/j.gendis.2025.101969)
Supplement: Supplementary file 2 [file mmc2.docx]

Supplemental table 1. Microglial activation genes module

**Activate genes**

Srgn Gpr84 Ccl5 Igsf6 Ms4a6d Il1rn Cybb Cxcl10 Tnf Cxcl16 Il1r2 Il15ra Slc7a5 Mmp14 Slc39a14 Fmnl2 Spp1 Il2rg Slc7a11 Slc13a3 Gabbr1 Cd86

Il1b Il6 Il18

Ccl2 Ccl3 Ccl4 Cxcl2 Srxn1 Csf1 Cd63

Lgals3 Fcgr4 Arg1 App Tyrobp Atpif1 Pten

Supplemental table 2. Microglial quiescent genes module

**Quiescent genes**

Cx3cr1 Gpr34 P2ry12 Plxdc2 Olfml3 Slc2a5 Ighm Arhgap5 Tmem119 Sall1 Hpgd P2ry13 Nav2 Lpcat2 Scoc

Bin1 Aif1 Pmepa1 Srgap2 Siglech Marcks F11r

Adgrg1 Lair1 Slco2b1 Col27a1 Susd3 Csmd3 Tanc2 Dnajb1 Fgd2 Tmem173 Fchsd2 Nav3 Crybb1 Rgs3

Supplemental table 3: Microglia CKO vs WT genes expression in E17.5

|  | p_val | avg_log2FCsig |
| --- | --- | --- |
| Stard7 | 310E-05 | 3418561 significant Up |
| Yif1b | 0000865 | -273695 significant Down |
| Psmd1 | 0000919 | -167129 significant Down |
| Aebp2 | 0000992 | 2348399 significant Up |
| Polg | 0001397 | -312214 significant Down |
| Xist | 0001833 | 0814972 significant Up |
| Sgce | 0001844 | 1501457 significant Up |
| Lzts3 | 0001995 | 3280864 significant Up |
| Wdr73 | 0001995 | 3845481 significant Up |
| Trim59 | 0002098 | 1467028 significant Up |
| Rpl18 | 0002582 | -086654 significant Down |
| Zdhhc1 | 0003116 | 288718 significant Up |
| Cyr61 | 0003156 | -436122 significant Down |
| Rpl8 | 0003417 | -052628 Down |
| Ube2i | 000343 | -123027 significant Down |
| Dynll1 | 0003556 | 0753277 significant Up |
| Rpl9 | 0004915 | -057211 Down |
| Rps27a | 0004915 | -051965 Down |
| Mettl21a | 0005178 | 3714507 significant Up |
| Ndst3 | 0005178 | 3345709 significant Up |
| Sgol2a | 0005178 | 4106816 significant Up |
| Pck2 | 0005273 | -294312 significant Down |
| Ddx3y | 0005273 | -328012 significant Down |
| 1700123O | 0005318 | -179661 significant Down |
| Rpl23a | 0005365 | -048406 Down |
| Bicd2 | 0005376 | 1122046 significant Up |
| Rtn3 | 0005642 | -093749 significant Down |
| Tsix | 0005831 | 1853937 significant Up |
| Rpl41 | 000698 | -059384 significant Down |
| Tpgs2 | 000718 | 086747 significant Up |
| Spsb3 | 0007269 | 2340284 significant Up |
| Wsb2 | 0007277 | -191565 significant Down |
| Gpx1 | 0007541 | -136702 significant Down |
| Ralgps1 | 0007684 | 2001632 significant Up |
| Gm5617 | 0008071 | 158402 significant Up |
| Rps15a | 0008107 | -052257 Down |
| Borcs7 | 0008296 | -229857 significant Down |
| Elmod3 | 0008351 | 3246239 significant Up |
| Rft1 | 0008351 | 2855039 significant Up |
| Insig1 | 0008397 | 1558232 significant Up |
| Npc2 | 0008439 | -111999 significant Down |
| Chchd2 | 0008622 | -059739 significant Down |
| Ebf4 | 0008711 | -234027 significant Down |
| Coq9 | 0008711 | -315608 significant Down |
| Hint3 | 0008711 | -249198 significant Down |
| Mon2 | 0008711 | -283569 significant Down |
| Aatf | 0008711 | -297386 significant Down |
| Snhg9 | 0008711 | -281281 significant Down |

| Pdlim1 | 0008711 | -476171 significant Down |
| --- | --- | --- |
| Bphl | 0008938 | 2188294 significant Up |
| Trmt6 | 0009289 | -231079 significant Down |
| Spc24 | 0009729 | 2740673 significant Up |
| Oxsr1 | 0009729 | 3247857 significant Up |
| Rpl30 | 0009791 | -049035 Down |
| Mif | 0009968 | -077424 significant Down |
| Epm2aip1 | 001026 | 1592514 significant Up |
| Dnttip2 | 0010723 | -144571 significant Down |
| Rps20 | 0010861 | -068 significant Down |
| Rnf149 | 0010881 | -197945 significant Down |
| Fam207a | 0011062 | 2614403 significant Up |
| Atg7 | 001113 | 1670372 significant Up |
| Srek1ip1 | 0011245 | -158836 significant Down |
| Btf3 | 0011295 | -050481 Down |
| Tomm40l | 0011307 | 2545148 significant Up |
| Tubgcp2 | 0011334 | 2054666 significant Up |
| Prr13 | 0011477 | -326763 significant Down |
| Rps21 | 0011788 | -054883 Down |
| Spry2 | 0012003 | 1440987 significant Up |
| Dnajb4 | 00122 | -151983 significant Down |
| Cux1 | 0012311 | -108891 significant Down |
| Gpn3 | 0012525 | -265312 significant Down |
| Dapk1 | 0012525 | -264962 significant Down |
| Supt3 | 0012756 | 1952359 significant Up |
| Rpf1 | 0012842 | -186738 significant Down |
| Fam184a | 001321 | 2223054 significant Up |
| Cables2 | 0013262 | 2688419 significant Up |
| Nphp4 | 0013262 | 3048413 significant Up |
| Trim56 | 0013262 | 2939646 significant Up |
| Col1a2 | 0013262 | 4028516 significant Up |
| Reep1 | 0013262 | 3232645 significant Up |
| Vstm2b | 0013262 | 3901062 significant Up |
| Gm10643 | 0013262 | 2698285 significant Up |
| Ddo | 0013262 | 2872131 significant Up |
| Mier2 | 0013262 | 2699082 significant Up |
| Ndufa4l2 | 0013262 | 6074455 significant Up |
| Lrrn3 | 0013262 | 3174434 significant Up |
| Zfp518a | 0013262 | 2816196 significant Up |
| Siah1a | 0013434 | 1846614 significant Up |
| Phf2 | 0013891 | 1858926 significant Up |
| Fut8 | 0013924 | 2820857 significant Up |
| Orai1 | 0014159 | -175767 significant Down |
| Scyl3 | 0014245 | -245561 significant Down |
| Gramd1b | 0014245 | -268929 significant Down |
| Dzip1l | 0014245 | -25116 significant Down |
| Naa16 | 0014245 | -314197 significant Down |
| Setd8 | 0014424 | 1810408 significant Up |
| Rpl3 | 0014707 | -053261 Down |
| Prpf6 | 0014943 | -183079 significant Down |

| Gtf3c6 | 0014943 | -159521 significant Down |
| --- | --- | --- |
| Eml4 | 001566 | -173314 significant Down |
| Mipol1 | 0015848 | 2022577 significant Up |
| Hyal2 | 0016714 | -174796 significant Down |
| Ap3b1 | 0016963 | -174745 significant Down |
| Picalm | 0017202 | -169641 significant Down |
| Aqr | 0017456 | -130786 significant Down |
| Ankrd13a | 0017773 | -237345 significant Down |
| Cecr2 | 0017773 | -222585 significant Down |
| Kdm5a | 001818 | -131901 significant Down |
| Ubl7 | 0018808 | -162284 significant Down |
| Adamts1 | 0018836 | 2658583 significant Up |
| Mettl6 | 0018836 | -197861 significant Down |
| Usp11 | 0019173 | -204384 significant Down |
| Ubap1 | 0019954 | 1672833 significant Up |
| Dctn5 | 002013 | 1224327 significant Up |
| Papd5 | 0020203 | -303096 significant Down |
| Gm1673 | 0020805 | 088235 significant Up |
| Dctn4 | 0020827 | -213621 significant Down |
| Usp10 | 0021025 | -187334 significant Down |
| Esd | 0021078 | -17601 significant Down |
| Tbc1d23 | 0021129 | -222163 significant Down |
| Timm9 | 0021519 | 2594865 significant Up |
| Jun | 0021568 | 1186871 significant Up |
| Dynlt1c | 0021669 | -218307 significant Down |
| Rnf24 | 0021807 | 2951767 significant Up |
| Rhou | 0021807 | 2921871 significant Up |
| Rasa2 | 0021807 | 3277234 significant Up |
| Mbd1 | 0021807 | 2676176 significant Up |
| Ccdc22 | 0021807 | 2488395 significant Up |
| Tgfbr1 | 0022007 | -189601 significant Down |
| Usf3 | 0022274 | 3776993 significant Up |
| Tspyl1 | 0022295 | 1774179 significant Up |
| Tmem2 | 0022365 | 1120701 significant Up |
| Rrnad1 | 0022365 | -23978 significant Down |
| Tbc1d14 | 0022409 | -210192 significant Down |
| Sec11c | 0022476 | -23259 significant Down |
| Qsox2 | 002309 | -223534 significant Down |
| Etv3 | 002309 | -298646 significant Down |
| Rarres2 | 002309 | -347491 significant Down |
| Mre11a | 002309 | -192237 significant Down |
| Arih2 | 002309 | -259427 significant Down |
| Gnaz | 002309 | -351782 significant Down |
| Polrmt | 002309 | -265747 significant Down |
| Fam193b | 002309 | -251533 significant Down |
| Scaf4 | 002309 | -346317 significant Down |
| Kdm5d | 002309 | -235203 significant Down |
| Zbtb45 | 0023467 | 2699698 significant Up |
| Fbxl19 | 0023467 | 2698466 significant Up |
| Fam149a | 0023467 | 2301848 significant Up |

| Fam229b | 0023467 | 2658986 significant Up |
| --- | --- | --- |
| Pcbp3 | 0023467 | 2397656 significant Up |
| Trim23 | 0023467 | 2373728 significant Up |
| Svip | 0023589 | -201671 significant Down |
| Uqcrh | 0024113 | -067959 significant Down |
| Ssr4 | 0024225 | -121284 significant Down |
| Klhdc4 | 0025025 | -194676 significant Down |
| Rpl38 | 0025164 | -042189 Down |
| Rffl | 0025236 | 2195181 significant Up |
| Mcc | 0025236 | 2081584 significant Up |
| Narf | 0025253 | -163476 significant Down |
| Rps6kb1 | 0025408 | -124235 significant Down |
| Fzd1 | 0025792 | 2793356 significant Up |
| Slc12a4 | 0025792 | 2280131 significant Up |
| Aes | 0026017 | 0829197 significant Up |
| Rps4x | 0026131 | -054972 Down |
| Gm2000 | 0026454 | -155334 significant Down |
| Phf8 | 0026454 | -162829 significant Down |
| Tspan5 | 0026525 | 3784964 significant Up |
| Ercc6l2 | 0026686 | 1838683 significant Up |
| Cuta | 0027024 | -099071 significant Down |
| Rad51ap1 | 0027119 | 2767093 significant Up |
| Wdr4 | 0027119 | 2231094 significant Up |
| Vrk1 | 0027162 | -166303 significant Down |
| Donson | 0027476 | 2504732 significant Up |
| Zfp40 | 0027476 | 2191529 significant Up |
| Akr1b3 | 0028009 | -254947 significant Down |
| Eif5a | 0028056 | -069368 significant Down |
| Bin3 | 002809 | -302114 significant Down |
| Gm37494 | 0028152 | 2112646 significant Up |
| Psmb2 | 0028433 | -081277 significant Down |
| Rpsa | 0028641 | -038647 Down |
| Pdap1 | 0029118 | -087513 significant Down |
| Laptm4b | 0029122 | 1918262 significant Up |
| Cep112 | 0029253 | 2389888 significant Up |
| Nrbp1 | 0029371 | -175929 significant Down |
| Msl2 | 0029423 | 1073231 significant Up |
| Lrwd1 | 0029527 | -094576 significant Down |
| Mettl2 | 0029527 | -153077 significant Down |
| Carnmt1 | 0029588 | 1883464 significant Up |
| Fam199x | 0029734 | -197125 significant Down |
| Nudcd1 | 0029734 | 2462964 significant Up |
| Ercc1 | 0029805 | -138172 significant Down |
| Asf1a | 0030317 | 1012791 significant Up |
| Mat2b | 0030318 | -114677 significant Down |
| Psmd7 | 003041 | -103109 significant Down |
| Pex13 | 0030436 | 1101809 significant Up |
| Igfbp4 | 0030443 | -25411 significant Down |
| Spop | 0031067 | 1098238 significant Up |
| Med27 | 0031129 | 2094883 significant Up |

| Nyap1 | 0031129 | 2074516 significant Up |
| --- | --- | --- |
| Eif4e3 | 0031129 | 1866801 significant Up |
| Klhl25 | 0031129 | 1995337 significant Up |
| Zkscan17 | 0031129 | 198743 significant Up |
| Zfp943 | 0031129 | 2074847 significant Up |
| Bag4 | 0031208 | 0505963 Up |
| Efcab12 | 0031251 | 1826189 significant Up |
| Pak4 | 0031268 | 1651979 significant Up |
| Tmem38a | 0031268 | 1813898 significant Up |
| Rpl5 | 0031338 | -043428 Down |
| Rpl32 | 0031359 | -04467 Down |
| Fbxo21 | 003146 | -255825 significant Down |
| Me1 | 003146 | -217301 significant Down |
| Rnf6 | 003146 | 2215609 significant Up |
| Trim27 | 0032675 | 1300929 significant Up |
| Mrps26 | 0032786 | -158697 significant Down |
| Tbcc | 0032819 | -07079 significant Down |
| Lmcd1 | 0032938 | 2029416 significant Up |
| Pdss2 | 0033107 | 1911511 significant Up |
| Snapc3 | 0033271 | -240873 significant Down |
| Gpr146 | 0033271 | -182636 significant Down |
| Slc6a8 | 0033271 | -182071 significant Down |
| Bloc1s6 | 0033271 | 1897356 significant Up |
| Cryz | 0033271 | 1704776 significant Up |
| Gstt1 | 0033271 | 2079596 significant Up |
| Rnf5 | 0033333 | -149918 significant Down |
| Gabarapl1 | 0033367 | -195167 significant Down |
| Ifitm3 | 0033386 | 1624044 significant Up |
| Polr2i | 0033525 | -112647 significant Down |
| Lats1 | 0034236 | -18916 significant Down |
| Leng1 | 0034236 | 2244866 significant Up |
| Cdc16 | 0034268 | -153423 significant Down |
| Iglon5 | 003428 | 149928 significant Up |
| Pdcd4 | 0034657 | 1468702 significant Up |
| Nagk | 0034921 | -243836 significant Down |
| Sf3b4 | 0034945 | -035113 Down |
| Cnih2 | 0035121 | -147352 significant Down |
| Borcs6 | 003517 | -32795 significant Down |
| Iars | 003517 | -232466 significant Down |
| Nr2c2ap | 0035191 | 2292758 significant Up |
| Mrto4 | 0036316 | -171259 significant Down |
| Snx5 | 0036481 | -151312 significant Down |
| Wdr1 | 0036617 | 1311829 significant Up |
| Cstf2t | 0036727 | 1561197 significant Up |
| Prmt3 | 0036856 | -144709 significant Down |
| Snrpb2 | 0037096 | 1568736 significant Up |
| Sobp | 0037162 | -187801 significant Down |
| Nagpa | 0037162 | -261218 significant Down |
| Fam219a | 0037162 | 1347373 significant Up |
| Pcbd1 | 0037162 | 1796274 significant Up |

| Fcgr3 | 0037167 | -561504 significant Down |
| --- | --- | --- |
| Pnpla7 | 0037167 | -222097 significant Down |
| Pxmp4 | 0037167 | -354073 significant Down |
| P2ry1 | 0037167 | -227151 significant Down |
| Atxn7l2 | 0037167 | -227628 significant Down |
| Cd52 | 0037167 | -449159 significant Down |
| P2rx7 | 0037167 | -242381 significant Down |
| Cep41 | 0037167 | -229099 significant Down |
| Hdac11 | 0037167 | -235124 significant Down |
| Syn2 | 0037167 | -177785 significant Down |
| Pnmal1 | 0037167 | -206502 significant Down |
| Kcnc3 | 0037167 | -212316 significant Down |
| Paox | 0037167 | -209587 significant Down |
| Angptl6 | 0037167 | -205282 significant Down |
| Rpl10-ps3 | 0037167 | -387822 significant Down |
| Igdcc3 | 0037167 | -224059 significant Down |
| Nf2 | 0037167 | -181723 significant Down |
| Pnpt1 | 0037167 | -247072 significant Down |
| Prkca | 0037167 | -272192 significant Down |
| Tmem94 | 0037167 | -220976 significant Down |
| Lhfpl2 | 0037167 | -267215 significant Down |
| Cacna1d | 0037167 | -252555 significant Down |
| Spryd3 | 0037167 | -284036 significant Down |
| Hmgxb3 | 0037167 | -203294 significant Down |
| Slc25a28 | 0037167 | -220417 significant Down |
| Mpp1 | 0037167 | -421649 significant Down |
| Arxes1 | 0037167 | -203006 significant Down |
| Eif2s3y | 0037167 | -267684 significant Down |
| Gtf2h2 | 0037386 | 1339071 significant Up |
| Cdc42bpb | 0037399 | 1476647 significant Up |
| Cnbp | 0037427 | 0628342 significant Up |
| Zdhhc3 | 0037807 | -164784 significant Down |
| Ept1 | 0037807 | 219741 significant Up |
| Cnot6l | 0038068 | -129878 significant Down |
| Apbb1 | 0038068 | -205905 significant Down |
| Praf2 | 0038265 | 1291541 significant Up |
| Tox3 | 0038389 | 1152173 significant Up |
| Sh3glb1 | 0038766 | -091277 significant Down |
| Plcd4 | 0039131 | 1923973 significant Up |
| Nudcd2 | 0039147 | 0858982 significant Up |
| Zfp444 | 0039248 | -161699 significant Down |
| Med23 | 0039248 | -164067 significant Down |
| Hsd17b4 | 0039425 | 1050939 significant Up |
| Rpl35a | 0039471 | -042792 Down |
| Sorbs2 | 0039697 | 1584822 significant Up |
| Cdk1 | 003973 | 1974253 significant Up |
| Eif4a2 | 0039844 | 0936728 significant Up |
| Slc3a2 | 0039846 | 0961481 significant Up |
| Rnf220 | 0039857 | 2211242 significant Up |
| Thoc3 | 0040071 | 155224 significant Up |

| AU022252 | 0040403 | 1545102 significant Up |
| --- | --- | --- |
| Atp2a2 | 0040513 | 0996031 significant Up |
| Skiv2l | 0040674 | -09073 significant Down |
| Csrp2 | 0041074 | 1760102 significant Up |
| Cdc37l1 | 0041289 | -079232 significant Down |
| Ddit4 | 0041289 | 10355 significant Up |
| Psmg4 | 0041403 | -145272 significant Down |
| Fam136a | 0041453 | -143356 significant Down |
| Gjc1 | 0041453 | 2489021 significant Up |
| Tada1 | 0041688 | -193679 significant Down |
| Ywhah | 0042066 | -105929 significant Down |
| Abcd2 | 0042128 | 1784494 significant Up |
| Mkrn1 | 0042562 | -210633 significant Down |
| Fstl1 | 0042816 | 1476078 significant Up |
| Stt3b | 0042837 | 0984947 significant Up |
| Rpl7a | 0043028 | -048444 Down |
| Rps18 | 0043028 | -043515 Down |
| Fuz | 004321 | -158256 significant Down |
| Proser1 | 0043239 | -14028 significant Down |
| Myl6 | 0043564 | -112592 significant Down |
| Syf2 | 0043587 | -080181 significant Down |
| Gnptg | 004362 | 1380681 significant Up |
| Kazn | 0043753 | -150523 significant Down |
| Pyroxd1 | 0043753 | -126949 significant Down |
| Mccc2 | 0043753 | -166101 significant Down |
| Pbx4 | 0043753 | 2240019 significant Up |
| Smad4 | 004398 | 1546296 significant Up |
| Rock2 | 0044188 | 1334956 significant Up |
| Mrpl14 | 0044482 | 0982463 significant Up |
| Nek7 | 0044909 | -211022 significant Down |
| Zfp639 | 0044909 | 1611152 significant Up |
| Senp3 | 0044909 | 1708665 significant Up |
| Pih1d1 | 0044974 | 1943112 significant Up |
| Orc6 | 0045889 | 118676 significant Up |
| Med9 | 0045903 | -133043 significant Down |
| Pinx1 | 0045903 | -162876 significant Down |
| Ddx19a | 0045918 | -180454 significant Down |
| A830080D | 0045918 | -135104 significant Down |
| Amd1 | 0045949 | -164538 significant Down |
| Gbf1 | 0045949 | -141411 significant Down |
| Gm973 | 0046114 | -035932 Down |
| Sec24a | 0046114 | -137175 significant Down |
| Rela | 0046114 | 1447946 significant Up |
| Lsm14b | 0046452 | 0984424 significant Up |
| Usf1 | 004658 | 178614 significant Up |
| Rps2 | 0046868 | -066348 significant Down |
| Spire1 | 0046946 | -11494 significant Down |
| Fuca1 | 0047007 | -174576 significant Down |
| Rps3a1 | 0047671 | -043087 Down |
| Cmc1 | 0047825 | 0528863 Up |

| Faf2 | 0047933 | -164955 significant Down |
| --- | --- | --- |
| Tmem80 | 0048141 | -149265 significant Down |
| Phf12 | 0048141 | -066476 significant Down |
| Tmem44 | 0048141 | -14506 significant Down |
| 2700097O | 0048616 | 1428285 significant Up |
| Mak16 | 0048628 | 1334669 significant Up |
| Bex1 | 0048768 | -107924 significant Down |
| Mdn1 | 0049139 | -197565 significant Down |
| Zc3hc1 | 0049139 | -181588 significant Down |
| C030037D | 0049139 | -191325 significant Down |
| Otud7b | 0049255 | -171732 significant Down |
| Metap1 | 0049255 | -188218 significant Down |
| Fbl | 0049329 | -125184 significant Down |
| Trim35 | 0049651 | -12531 significant Down |
| Wbp2 | 0049769 | -13859 significant Down |
| Smad5 | 0049781 | 1327616 significant Up |
| Igf1r | 0049845 | 1482449 significant Up |
| Mier1 | 0050593 | -138973 Down |
| Malsu1 | 0050634 | 1312403 Up |
| Sbds | 0050933 | -169533 Down |
| Npm1 | 0050987 | -049355 Down |
| Rps29 | 0050987 | -049895 Down |
| G2e3 | 0051235 | 1352922 Up |
| Pelo | 005139 | 167814 Up |
| Fkbp2 | 0051396 | 0934313 Up |
| Nae1 | 0051829 | 1055929 Up |
| Arl6ip6 | 0052333 | 1246682 Up |
| Fbxl6 | 0052893 | -080596 Down |
| Nob1 | 0053717 | 1445433 Up |
| 5430416N | 0054259 | -163532 Down |
| Rpl21 | 0054494 | -047304 Down |
| Psmd2 | 0054588 | 0868064 Up |
| Npepl1 | 005473 | 1428135 Up |
| Prkab1 | 005479 | -213556 Down |
| Mfap4 | 005479 | -224016 Down |
| Fyb | 005479 | -324869 Down |
| Ids | 005479 | -195382 Down |
| Rbm48 | 005479 | 2693018 Up |
| Isoc1 | 005479 | 2064565 Up |
| G0s2 | 0055137 | 3690271 Up |
| Eef1g | 0055144 | -060077 Down |
| 2410015M | 0055189 | -073755 Down |
| Paip2 | 0055194 | -063245 Down |
| Fam50a | 0055374 | -133118 Down |
| Ap4m1 | 0055412 | 2301177 Up |
| Ncapd3 | 0055412 | 1908925 Up |
| AY036118 | 0055435 | -015293 Down |
| Nuak2 | 0055559 | 2147581 Up |
| Cers6 | 0055559 | 2593243 Up |
| Edc4 | 0055559 | 25377 Up |

| Zfp748 | 0055559 | 4504168 Up |
| --- | --- | --- |
| Vars2 | 0055559 | 3044246 Up |
| Stard4 | 0055559 | 2798002 Up |
| Rpp25l | 0055684 | 1162483 Up |
| Psmg3 | 0056132 | -265178 Down |
| Nhp2 | 0056416 | -152366 Down |
| Mrps5 | 0056777 | -066469 Down |
| Lrrc8d | 0057058 | -108169 Down |
| Yeats4 | 0057073 | -086411 Down |
| Tmed4 | 0057251 | -111005 Down |
| Eif6 | 0057307 | -091198 Down |
| Eif3h | 0057707 | -027498 Down |
| Cebpz | 0057807 | -115488 Down |
| Ogfod2 | 0057812 | -157656 Down |
| Ube2m | 0057941 | -076128 Down |
| Dhx8 | 0058286 | 083278 Up |
| Tial1 | 0058399 | 0854573 Up |
| Ncbp2 | 0058477 | -174656 Down |
| Maf | 0058636 | -232572 Down |
| Nr3c1 | 0058636 | -116556 Down |
| Kmt2e | 0059011 | -081643 Down |
| Rpl10a | 0059126 | -044274 Down |
| Phf20l1 | 0059357 | 1401906 Up |
| Ccdc34 | 0059403 | 0890145 Up |
| Purb | 0059403 | 1261576 Up |
| Brd2 | 0059531 | 1233 Up |
| Chpt1 | 0059537 | -132244 Down |
| Rab23 | 0059552 | 2122476 Up |
| Tmem18 | 0059552 | 1957128 Up |
| Dusp22 | 0059552 | 2102187 Up |
| Tcf19 | 0059552 | 336152 Up |
| Nutf2 | 0059557 | -120747 Down |
| Orai3 | 0059681 | -148625 Down |
| Enpp5 | 0059681 | -195298 Down |
| Git1 | 0060077 | 2138509 Up |
| Fmnl3 | 0060077 | 3000446 Up |
| Lcorl | 0060751 | 2961389 Up |
| Mxd1 | 0060751 | 1406982 Up |
| Dym | 0060751 | 0805862 Up |
| Srcap | 0060834 | 1798378 Up |
| Hn1 | 0060917 | -06729 Down |
| Ifi30 | 0060972 | -298602 Down |
| Cog2 | 0060972 | -153158 Down |
| Med7 | 0061122 | -09672 Down |
| Ccdc90b | 00612 | 1261195 Up |
| Vcl | 0061233 | -22702 Down |
| Rgl1 | 0061233 | 1849034 Up |
| Sox6 | 0061406 | -164645 Down |
| Rps5 | 0062113 | -046055 Down |
| Riok3 | 0062608 | -345073 Down |

| Mrpl36 | 0062786 | -131729 Down |
| --- | --- | --- |
| Rps25 | 0062845 | -058209 Down |
| Polr2k | 006305 | -07392 Down |
| Acbd5 | 0063447 | -11094 Down |
| Rnft1 | 0063577 | -104429 Down |
| Ttll12 | 0063577 | -080283 Down |
| Nudt19 | 0063581 | -192057 Down |
| Rbl1 | 0063723 | 2070279 Up |
| S1pr3 | 0063723 | 3384973 Up |
| Rbmx2 | 0063723 | 3083921 Up |
| Tsen15 | 0063753 | -12537 Down |
| Zfp788 | 0063781 | 1846654 Up |
| Bcat2 | 0063781 | 1971294 Up |
| Zfp105 | 0063781 | 2163506 Up |
| Lca5l | 0063781 | 1890293 Up |
| Prmt10 | 0063781 | 3596104 Up |
| Dlg5 | 006386 | 1687312 Up |
| Ppic | 006386 | 1659387 Up |
| Phc3 | 0063924 | 135225 Up |
| Usp9x | 006398 | -053791 Down |
| Rps9 | 0064136 | -049394 Down |
| Rexo4 | 0064274 | -154207 Down |
| Ptpn23 | 0064274 | -262011 Down |
| Acsf2 | 0064274 | -209487 Down |
| Ccnb1 | 0064274 | -223657 Down |
| 2510002D2 | 0064274 | -162613 Down |
| Esco1 | 0064274 | -127341 Down |
| Plp1 | 0064274 | -154297 Down |
| Plekha3 | 0064274 | 1879435 Up |
| Stk3 | 0064274 | 243644 Up |
| Capzb | 0064341 | -06933 Down |
| Ptpn1 | 0065276 | 1173959 Up |
| Mios | 0065544 | -161993 Down |
| Jund | 0065954 | 0621126 Up |
| Ap2b1 | 0066061 | -061475 Down |
| Med13 | 0066095 | -050567 Down |
| Ganab | 0066712 | -12347 Down |
| Mllt1 | 0066712 | 1139231 Up |
| Gpx7 | 006701 | 150565 Up |
| Itm2a | 006701 | 1673188 Up |
| Adgrb1 | 0067543 | -09155 Down |
| Erlin2 | 0067543 | 089161 Up |
| Rfxank | 006755 | 2032224 Up |
| Crlf1 | 006755 | 1776388 Up |
| Frs2 | 006755 | 2276828 Up |
| Gm12942 | 006755 | 1722663 Up |
| Bud31 | 0067594 | -019038 Down |
| Nif3l1 | 0067721 | -207621 Down |
| Ptgds | 0067721 | -149526 Down |
| Cox6b2 | 0067721 | -235982 Down |

| P3h4 | 0067721 | -144957 Down |
| --- | --- | --- |
| Ubxn7 | 0067884 | -131723 Down |
| Stra13 | 0067934 | -112506 Down |
| Rbbp5 | 0068256 | 1594207 Up |
| Slc7a1 | 0068256 | 1967755 Up |
| Tmem178b | 0068256 | 2102755 Up |
| Rtkn | 0068256 | 1705633 Up |
| Cx3cl1 | 0068256 | 1695176 Up |
| Rdh5 | 0068256 | 1621783 Up |
| Fbxo32 | 0068256 | 2282668 Up |
| Plxna3 | 0068256 | 176718 Up |
| Prmt5 | 0068339 | -152044 Down |
| Ep300 | 0068573 | -108019 Down |
| Chchd5 | 006865 | -110252 Down |
| Rnf19a | 006865 | -148969 Down |
| Ap3s1 | 0068691 | -017558 Down |
| Rock1 | 0069156 | 0472175 Up |
| Hist1h2bc | 0069537 | -265997 Down |
| Ndufs5 | 006958 | -052821 Down |
| Alkbh3 | 0069599 | -116132 Down |
| Adam9 | 0069599 | -11184 Down |
| Psen1 | 0069599 | -248522 Down |
| 2310035C2 | 0069599 | -1779 Down |
| Cdkn2d | 0069599 | 0156352 Up |
| BC030336 | 0069599 | 1565244 Up |
| Hbs1l | 006974 | 1065626 Up |
| Rraga | 0069875 | -078765 Down |
| Ttll3 | 0070287 | 1200971 Up |
| Tbc1d20 | 0070287 | -145705 Down |
| Foxk2 | 0070682 | 117538 Up |
| Sp4 | 0070682 | -172925 Down |
| Akt1 | 0070869 | -103105 Down |
| Acadm | 0070869 | 0828025 Up |
| Tmbim4 | 0070958 | -054089 Down |
| Tmem183a | 0071127 | -119558 Down |
| Hmgn5 | 0071127 | -121494 Down |
| Aph1a | 0071127 | 1330748 Up |
| Pgm3 | 0071224 | -126472 Down |
| Daglb | 007132 | -10242 Down |
| Fam208b | 007132 | 0475503 Up |
| Fam65b | 007132 | -08467 Down |
| Blvra | 007132 | 1809581 Up |
| Dlgap4 | 0071505 | 1225815 Up |
| Eif2ak3 | 0071564 | 2149784 Up |
| Chst11 | 0071564 | 195034 Up |
| Slc18a2 | 0071564 | 2136495 Up |
| Rpl4 | 0071698 | -039686 Down |
| Rpl18a | 0071713 | -042565 Down |
| Hdac3 | 0071854 | 1462861 Up |
| Lpin2 | 0071875 | -239628 Down |

| Rrm2 | 0072025 | 1669386 Up |
| --- | --- | --- |
| Kctd5 | 0072025 | 1401467 Up |
| Tpd52 | 0072589 | -129548 Down |
| Gse1 | 0072589 | -137814 Down |
| Sf3b6 | 0072682 | -079463 Down |
| Smpd2 | 0072711 | -060703 Down |
| Zfp395 | 0072711 | 1368203 Up |
| Neb | 0072985 | 072654 Up |
| Dlgap3 | 0072985 | 1113332 Up |
| Rcc1 | 0072985 | 1679439 Up |
| Phtf2 | 0072985 | 1545046 Up |
| Depdc5 | 0072985 | 1256063 Up |
| Cdt1 | 0072985 | 1558828 Up |
| Prr11 | 0072985 | 1469241 Up |
| Zfp160 | 0072985 | 1217186 Up |
| Slk | 0072985 | 13397 Up |
| Lrrc47 | 0073694 | 1648493 Up |
| Sugt1 | 007392 | 1425249 Up |
| Raver2 | 0074494 | -162052 Down |
| Rab2a | 0074512 | -077139 Down |
| Hnrnpc | 0074675 | -067733 Down |
| l7Rn6 | 0074718 | 1039883 Up |
| Cdc23 | 0074902 | -076154 Down |
| Phf10 | 0074902 | 0594383 Up |
| Srrm2 | 007497 | 0603188 Up |
| Mecr | 0075074 | 1973505 Up |
| Rnf121 | 0075074 | 1380623 Up |
| Mrps31 | 0075223 | -243547 Down |
| Pwp1 | 0075223 | -138782 Down |
| Pkig | 0075666 | -094805 Down |
| Mipep | 0075771 | 1797125 Up |
| Fam63a | 0075771 | 1859221 Up |
| Fam179b | 0075771 | 2249252 Up |
| Topors | 0075883 | 0326173 Up |
| Pax6 | 0075984 | 1084138 Up |
| Pold2 | 0075984 | 0711337 Up |
| Ptprj | 0076129 | -303127 Down |
| Psmc4 | 007632 | -072057 Down |
| Ssx2ip | 0076349 | 1023764 Up |
| Idnk | 007656 | 1338058 Up |
| Tbck | 007656 | 074141 Up |
| Fhad1 | 0077235 | 076778 Up |
| Srrt | 0077718 | -242747 Down |
| Tmx3 | 0077873 | 1417037 Up |
| Zfp362 | 0078697 | -089676 Down |
| Exoc5 | 0078793 | 0803613 Up |
| Snrpb | 0078913 | 0435178 Up |
| Slc30a6 | 0078987 | 2106115 Up |
| Pcyox1l | 0079377 | 0987099 Up |
| Trappc12 | 0079495 | 1310678 Up |

| Sf3a3 | 0079604 | -050977 Down |
| --- | --- | --- |
| Patz1 | 0079604 | 1042217 Up |
| Hspbp1 | 0079646 | -119223 Down |
| Lta4h | 0079646 | 1945256 Up |
| Wbp4 | 0079983 | 0979788 Up |
| Rpl13 | 0079999 | -039822 Down |
| Slc30a10 | 0080178 | 3189154 Up |
| Slc25a40 | 0080178 | 1470703 Up |
| Sgpp1 | 0080178 | 1292396 Up |
| Prkdc | 0080178 | 1248247 Up |
| Dgcr14 | 0080178 | 2463867 Up |
| Mrpl41 | 0081168 | -081814 Down |
| Zfos1 | 0081547 | -060698 Down |
| Lyrm2 | 0082133 | -093384 Down |
| Kansl1 | 0082133 | 1082082 Up |
| Dnajb6 | 0082541 | 0800398 Up |
| Gorasp2 | 0082747 | 1642673 Up |
| Amotl2 | 0082832 | 1003713 Up |
| Klf15 | 0082832 | -158977 Down |
| Trnt1 | 0082832 | -120017 Down |
| Alkbh6 | 0082832 | -122006 Down |
| Slc16a2 | 0082832 | 2380239 Up |
| Rtfdc1 | 0082989 | 0847164 Up |
| Rps10 | 0083776 | -032762 Down |
| 1500011K1 | 00839 | 112843 Up |
| Pin1 | 008392 | 0980474 Up |
| Arhgef12 | 0084103 | -105565 Down |
| Rnf20 | 0084451 | -090181 Down |
| Ciz1 | 0084647 | -215788 Down |
| Rgs19 | 0084647 | -228815 Down |
| Dera | 0084647 | -117662 Down |
| Hdac7 | 0084647 | -146099 Down |
| Decr2 | 0084647 | -147471 Down |
| Tti1 | 0084732 | 1184939 Up |
| Fahd2a | 0084732 | -148167 Down |
| Uxs1 | 0084791 | 1603478 Up |
| Strip1 | 0084791 | 0554244 Up |
| Spred3 | 0084791 | 1422379 Up |
| Nrp1 | 0084791 | 1607766 Up |
| Timeless | 0084791 | 1327766 Up |
| Utp23 | 0084791 | 1360108 Up |
| Hic2 | 0084791 | 1297474 Up |
| Gucy1b3 | 0084791 | 4184838 Up |
| Prkrir | 008509 | 1909288 Up |
| B3glct | 008549 | 1010721 Up |
| Hspa13 | 0085624 | 0792338 Up |
| Tmem160 | 0085624 | -093396 Down |
| Ngdn | 0085624 | -092829 Down |
| Xrn1 | 0086105 | -035852 Down |
| Spcs2 | 008625 | 1024057 Up |

| Rreb1 | 0086351 | 3057205 Up |
| --- | --- | --- |
| Men1 | 0086351 | 146606 Up |
| Uqcrc2 | 0086467 | -085229 Down |
| R3hdm1 | 0086473 | -073403 Down |
| Zkscan1 | 0086528 | 1254735 Up |
| Ankrd37 | 0086532 | 2370678 Up |
| Map4 | 0087043 | -078253 Down |
| Ube2g1 | 0087043 | 1290246 Up |
| Gng11 | 0087312 | 1138831 Up |
| Bicc1 | 0087312 | 1412095 Up |
| Zfp287 | 0087312 | 2233845 Up |
| Drg1 | 0088133 | -184581 Down |
| Atf5 | 0088133 | 1864207 Up |
| mt-Nd5 | 0088379 | 0345745 Up |
| Pmf1 | 0088696 | 232637 Up |
| Tsr2 | 0088696 | 1907356 Up |
| Wdr83os | 0088808 | -118718 Down |
| Glyr1 | 0088808 | -113196 Down |
| Rpl31 | 0088903 | -047787 Down |
| Foxg1 | 0088992 | 0241726 Up |
| Aldh2 | 0089091 | -125237 Down |
| Ccdc93 | 0089186 | -158995 Down |
| Flad1 | 0089186 | -175095 Down |
| Sphk2 | 0089186 | -170424 Down |
| Emp3 | 0089186 | -448226 Down |
| Elmo1 | 0089186 | -238074 Down |
| Gm8730 | 0089186 | -247883 Down |
| Usp33 | 0089573 | 2519776 Up |
| Mri1 | 0089616 | 1250022 Up |
| Vps13a | 0089616 | 0939939 Up |
| Bicd1 | 0090006 | 1762982 Up |
| Paxbp1 | 009028 | 1075369 Up |
| Mns1 | 009073 | 1202522 Up |
| Phb2 | 0091217 | -037356 Down |
| Brd4 | 009153 | 0592773 Up |
| Cox4i1 | 009163 | -036444 Down |
| Svbp | 0091668 | -087264 Down |
| Acd | 0091732 | 1329676 Up |
| Tmem230 | 0091771 | -151742 Down |
| Rnf7 | 0091817 | -045518 Down |
| Zfhx3 | 0092006 | -111928 Down |
| Mtss1l | 0092078 | -148285 Down |
| Ndufb7 | 0092576 | -115558 Down |
| Cdc42ep4 | 0092808 | 1280973 Up |
| Prkar2b | 0092808 | -283539 Down |
| Igbp1 | 0092949 | -087999 Down |
| Kat6b | 0092949 | 2031037 Up |
| Pcdh17 | 0093013 | -138943 Down |
| Epha5 | 0093043 | -177163 Down |
| Pkm | 0093081 | -092362 Down |

| D1Ertd622 | 0093152 | 2319313 Up |
| --- | --- | --- |
| Rplp1 | 0093164 | -035821 Down |
| Slirp | 0093369 | -089244 Down |
| Tbc1d13 | 009392 | -191932 Down |
| Slc12a9 | 009392 | -140389 Down |
| Foxj2 | 009392 | -135616 Down |
| Ccl25 | 009392 | -125446 Down |
| Hpgd | 009392 | -195574 Down |
| Gpt2 | 009392 | -133641 Down |
| Rbl2 | 009392 | -150999 Down |
| Zfp1 | 009392 | -178984 Down |
| Auh | 0094197 | -066876 Down |
| Dnm2 | 0094482 | -101553 Down |
| Supt6 | 0094482 | -11271 Down |
| Klhl24 | 0094482 | 1190594 Up |
| Psmd14 | 0094516 | -099237 Down |
| Rpl17 | 009455 | -025589 Down |
| Hebp1 | 009495 | 1642208 Up |
| Smim10l1 | 0095724 | 1260222 Up |
| Coa5 | 0095981 | 2911137 Up |
| Zfp961 | 0096331 | 1766165 Up |
| C1galt1c1 | 0096331 | 111934 Up |
| C87436 | 0096571 | 0826909 Up |
| Epb41 | 0096622 | -152256 Down |
| Preb | 0096622 | -180517 Down |
| Heatr3 | 0097072 | 1408268 Up |
| Pknox1 | 0097072 | -131545 Down |
| Dus3l | 0097072 | -132047 Down |
| Foxn3 | 009777 | 1383567 Up |
| Stxbp3 | 009848 | 0832655 Up |
| Rnf14 | 009857 | -125795 Down |
| Mcts1 | 0098765 | -165224 Down |
| Cenpq | 0098798 | 1679261 Up |
| Zfp84 | 0098853 | -145237 Down |
| Spats2 | 0098853 | -146652 Down |
| Ric1 | 0098853 | -106216 Down |
| Mrpl43 | 0098987 | -094723 Down |
| Nckap1 | 0099204 | 0573109 Up |
| BC005561 | 0099344 | -106526 Down |
| Gm3764 | 0099455 | -086122 Down |
| A430106G | 0099929 | 0279914 Up |
| Saal1 | 0099929 | 124013 Up |
| Hist1h2ab | 0099929 | 0982037 Up |
| Bag1 | 010012 | -102251 Down |
| Nub1 | 0100138 | 210324 Up |
| Ube3c | 0100342 | 1324423 Up |
| Sh3rf1 | 0100655 | 1082812 Up |
| Adam10 | 0100824 | -082425 Down |
| Rngtt | 0101112 | 0800812 Up |
| Commd4 | 0101399 | -088104 Down |

| Spc25 | 010149 | 0352321 Up |
| --- | --- | --- |
| Lrrfip2 | 010149 | 1024532 Up |
| Golph3l | 010149 | -142688 Down |
| Hmg20a | 010149 | -176939 Down |
| Desi1 | 010149 | -230069 Down |
| Eef1b2 | 0101819 | -063923 Down |
| H3f3b | 0101862 | 0230698 Up |
| Ndufaf2 | 0101882 | -103813 Down |
| Tmed2 | 0101922 | -085781 Down |
| Gng3 | 0102059 | 0948277 Up |
| Chchd7 | 0102059 | 1008817 Up |
| Uchl5 | 0102114 | -219137 Down |
| Hnrnph3 | 0102436 | -06249 Down |
| Ube2a | 0102436 | 1714936 Up |
| Sox2 | 0102449 | 045657 Up |
| Rabac1 | 0103187 | 128257 Up |
| Erh | 0103217 | 031508 Up |
| Nfs1 | 0103786 | -141941 Down |
| Trp53i13 | 0103786 | -12013 Down |
| Rpl22l1 | 0103939 | 0558698 Up |
| Kcnd3 | 0103991 | -098989 Down |
| Fpgt | 0103991 | -136408 Down |
| Ostm1 | 0103991 | -11249 Down |
| 2810474O | 0104209 | -093581 Down |
| Hs3st1 | 0104581 | 2098636 Up |
| Nkiras1 | 0104581 | 1872164 Up |
| Ppp1r14b | 0104657 | -068714 Down |
| S100a10 | 0104686 | -311204 Down |
| Fbxw7 | 0105241 | 1333051 Up |
| Adgrb2 | 0105241 | 080668 Up |
| Sgta | 010567 | 0732004 Up |
| Asxl2 | 0105854 | -084478 Down |
| Fam58b | 0105854 | 1501522 Up |
| Tob2 | 0106068 | -116902 Down |
| Ccna2 | 010608 | 0787444 Up |
| Samd8 | 010608 | 119237 Up |
| Tdp2 | 0106709 | -078628 Down |
| Cttnbp2 | 0107177 | -15477 Down |
| Ddx24 | 0107394 | -070869 Down |
| Zfp637 | 0107915 | 0653925 Up |
| Utp18 | 0108017 | -119755 Down |
| Rxrb | 0108124 | -085014 Down |
| Hddc2 | 0108276 | -067377 Down |
| Arpc5 | 0108363 | -06069 Down |
| Ap2m1 | 0108412 | 0770251 Up |
| Scml4 | 0108624 | 0427754 Up |
| Faap20 | 0109102 | 1073101 Up |
| Prps1 | 0109111 | 1972065 Up |
| Pbx3 | 0109142 | 1059576 Up |
| Etfdh | 0109142 | 0876527 Up |

| Sh3bgrl | 0109233 | -072321 Down |
| --- | --- | --- |
| Mtx2 | 010924 | 0626593 Up |
| Mdh2 | 0109727 | -055033 Down |
| Ltn1 | 0110183 | 1761604 Up |
| Fance | 0110183 | 1180745 Up |
| Rce1 | 0110183 | 1427948 Up |
| Slc39a10 | 0110805 | -082289 Down |
| Tpbg | 0110808 | 0884398 Up |
| Alms1 | 011124 | 1131119 Up |
| Ddah2 | 0111518 | 0566508 Up |
| Ost4 | 011164 | -069213 Down |
| Kras | 0112111 | 0676515 Up |
| Ube3a | 0112286 | -097775 Down |
| Orc2 | 0112384 | -108953 Down |
| Rps17 | 0112434 | -038114 Down |
| Secisbp2 | 0112803 | 0768067 Up |
| Ino80b | 0113047 | 1886718 Up |
| Mrfap1 | 0113448 | -052608 Down |
| Clock | 0113468 | -11657 Down |
| Pou3f4 | 0113659 | 1057826 Up |
| Pak2 | 0113664 | 1238 Up |
| Ak1 | 0113781 | 1928894 Up |
| Acp1 | 0113822 | -10459 Down |
| Pcm1 | 0114001 | 0845844 Up |
| Spata6 | 0114619 | 1625879 Up |
| Hist1h2ae | 0114619 | 1805283 Up |
| Kansl2 | 0114778 | 1278612 Up |
| Mrps24 | 011493 | 0579909 Up |
| Cbx5 | 0114995 | 0696964 Up |
| Rsu1 | 0115609 | -113337 Down |
| Jakmip2 | 0115735 | -063424 Down |
| Hist1h2ap | 0115747 | 1995609 Up |
| Rbm27 | 0115747 | 1225259 Up |
| Nme2 | 0116376 | 0667808 Up |
| Oser1 | 0116376 | 1737464 Up |
| Tmem51 | 0116596 | 0952436 Up |
| Mpc2 | 0116621 | 0874382 Up |
| Dnajc8 | 0116636 | -034728 Down |
| Arpc1b | 0116721 | -153739 Down |
| Ankrd12 | 011677 | 0886866 Up |
| Csnk1g1 | 0116888 | -131505 Down |
| Snrnp25 | 0116888 | -141889 Down |
| Flii | 0116888 | -082835 Down |
| Ccdc177 | 0116888 | -078243 Down |
| Fkbp8 | 0117323 | 0730866 Up |
| Wsb1 | 0117466 | -078946 Down |
| Hook3 | 0117661 | 137025 Up |
| Mapk8ip1 | 0117661 | -064188 Down |
| Ppp2r5e | 0117728 | -103434 Down |
| Wbp1l | 0117728 | 1282938 Up |

| Fam193a | 0118203 | 0991368 Up |
| --- | --- | --- |
| Tmem165 | 0118429 | 0698913 Up |
| Sparcl1 | 0118614 | 2445714 Up |
| Comt | 0118641 | 1221662 Up |
| Sardh | 0119193 | 1136873 Up |
| Srsf1 | 0119224 | -090213 Down |
| Arhgef4 | 0119379 | 1810311 Up |
| Cxxc1 | 0119397 | -117809 Down |
| Taf11 | 0119632 | -061684 Down |
| Csmd1 | 0119632 | 0892944 Up |
| Hibadh | 0120589 | -073737 Down |
| Atp5j2 | 0120629 | -034737 Down |
| Arrdc3 | 0120684 | 2244278 Up |
| Lrpprc | 0120787 | 074992 Up |
| Gng10 | 0120787 | -147964 Down |
| Rnf141 | 0120787 | -120903 Down |
| Tnks | 0120787 | -099598 Down |
| Snx17 | 0121 | 0630901 Up |
| Cald1 | 012144 | 2095439 Up |
| Nedd4l | 012144 | -117081 Down |
| Taf12 | 0121524 | 1027173 Up |
| Nup50 | 0121524 | 1077064 Up |
| Akr1b10 | 0121533 | -109747 Down |
| Ptdss2 | 0121535 | 0895205 Up |
| Drap1 | 012181 | -052514 Down |
| Plch1 | 0121837 | 1754852 Up |
| Sat2 | 0122153 | 0731289 Up |
| Ammecr1 | 0122153 | 0903344 Up |
| Eef1a1 | 0122919 | -02778 Down |
| Supt4a | 0123193 | -051539 Down |
| Lrig2 | 012338 | -199469 Down |
| Igsf11 | 012338 | -208985 Down |
| Gm26724 | 012338 | -174109 Down |
| Large | 012338 | -204955 Down |
| Eif4h | 0123579 | -0845 Down |
| Dap | 0123784 | -12251 Down |
| Emc8 | 0123789 | 1138645 Up |
| Fam171a1 | 0123909 | 1527679 Up |
| Reps1 | 0123909 | 1189361 Up |
| Gstz1 | 0123909 | 1228732 Up |
| Ptprg | 0123909 | 106396 Up |
| Etnk1 | 0124304 | 0748788 Up |
| Mbd3 | 0124334 | 0746166 Up |
| Phpt1 | 0124529 | -065149 Down |
| Prkag1 | 0124752 | -154584 Down |
| Fgd1 | 012479 | -218046 Down |
| 2810006K2 | 0125398 | -137152 Down |
| Mpv17 | 0125587 | -041721 Down |
| Bdp1 | 0125587 | 0714178 Up |
| Rfx7 | 0125587 | 1057912 Up |

| 9430016H0 | 0125587 | -152264 Down |
| --- | --- | --- |
| Tipin | 012578 | -072646 Down |
| Fam126b | 0126034 | -063357 Down |
| Rhcg | 0126034 | -126709 Down |
| Mex3c | 0126077 | 0916216 Up |
| Galnt11 | 0126087 | 1433429 Up |
| Cep57 | 0126208 | -121298 Down |
| Mrps25 | 0126208 | 074738 Up |
| Snx13 | 0126321 | -085867 Down |
| Rplp2 | 0126442 | -030165 Down |
| Polr2g | 0126493 | 0446455 Up |
| Snca | 0126694 | 2616147 Up |
| Mrpl54 | 0127021 | -089968 Down |
| Gm10076 | 0127553 | 0919421 Up |
| Ccdc59 | 0127707 | 116694 Up |
| Stim2 | 0127914 | 0997396 Up |
| Twistnb | 0128132 | -14865 Down |
| Rpl27 | 0128184 | -016153 Down |
| Sod1 | 0128502 | 0685339 Up |
| Slc7a2 | 0128763 | 1482122 Up |
| Wipf2 | 0128763 | 1473893 Up |
| Pacsin2 | 0128763 | 1732396 Up |
| Eva1b | 012877 | 2212098 Up |
| Dhcr7 | 012877 | 1550166 Up |
| Gdi1 | 0128852 | -035785 Down |
| Cdk8 | 0129673 | -075045 Down |
| Tssc4 | 0129673 | 120554 Up |
| Ctdnep1 | 0129708 | -114953 Down |
| Usp19 | 0129844 | 0778562 Up |
| Ankrd44 | 0129867 | -189458 Down |
| Smim1 | 0129867 | -195464 Down |
| Zfp827 | 0129867 | -162869 Down |
| Vps13b | 0129867 | -155423 Down |
| Mina | 0129867 | -144152 Down |
| Jtb | 0129899 | -09091 Down |
| Rps28 | 0129999 | -031784 Down |
| Ppp1ca | 0130388 | 0438144 Up |
| Lamc1 | 013039 | 3641281 Up |
| Atf1 | 013039 | 1504581 Up |
| Tsta3 | 0130391 | -020522 Down |
| Zfp931 | 0130763 | 1207594 Up |
| Tex264 | 0130763 | 116547 Up |
| Pik3r2 | 0131255 | -086868 Down |
| Zbtb1 | 0131255 | -095678 Down |
| Ubr3 | 0131322 | 2800695 Up |
| Sorl1 | 0131457 | -060124 Down |
| Dennd6a | 0131457 | -109323 Down |
| Bcorl1 | 0131457 | -0869 Down |
| Lamtor5 | 0131667 | -069964 Down |
| Scnm1 | 01319 | -031344 Down |

| Rfc2 | 0132256 | 1072064 Up |
| --- | --- | --- |
| Wbp11 | 013276 | -024526 Down |
| Nr2f2 | 0132933 | 2728416 Up |
| Tysnd1 | 0132933 | 1870003 Up |
| Ppm1d | 0133156 | -11554 Down |
| Anapc5 | 0133686 | 0488865 Up |
| Tnfrsf19 | 0133777 | 0935147 Up |
| Rbm10 | 0133777 | 1094815 Up |
| Tor2a | 0134083 | 1458674 Up |
| Rtn2 | 0134083 | 1026455 Up |
| Ipo7 | 0134535 | 1126046 Up |
| Alkbh5 | 0134963 | 0706071 Up |
| Tmed1 | 0135157 | -077865 Down |
| 2310011J0 | 0135157 | 0952254 Up |
| Trip4 | 0135388 | 1023184 Up |
| Sec23b | 0135583 | 0901185 Up |
| Ikzf2 | 0135653 | 0773606 Up |
| Tpst2 | 0135653 | -131975 Down |
| Yipf4 | 0135777 | -065309 Down |
| Tamm41 | 0136182 | -17216 Down |
| Taf1b | 0136182 | -118269 Down |
| A330048O | 0136182 | 1856243 Up |
| Siah1b | 0136182 | 1760364 Up |
| Trnau1ap | 01362 | -078477 Down |
| Prr7 | 01362 | 1394576 Up |
| Usp1 | 01362 | 0849893 Up |
| Ddx39 | 0136377 | -133722 Down |
| Wdr3 | 0136616 | -165586 Down |
| Fkbp14 | 0136616 | -321673 Down |
| Nfkbib | 0136616 | -133412 Down |
| Tlcd1 | 0136616 | -214571 Down |
| Jak2 | 0136616 | -193782 Down |
| C920006O | 0136616 | -159254 Down |
| Cttnbp2nl | 0136849 | 1702663 Up |
| Cox7a2l | 013707 | -018204 Down |
| Pdcd6 | 0137322 | -046909 Down |
| Zfp511 | 0137356 | -114722 Down |
| Eri3 | 0137597 | -126434 Down |
| Sdhb | 01377 | 1181618 Up |
| Mkln1 | 01377 | 0982695 Up |
| Dbn1 | 0137979 | -073261 Down |
| Zfp710 | 0138044 | -134426 Down |
| Dlx6os1 | 0138692 | 1457173 Up |
| Ppie | 0138692 | -095319 Down |
| Mzt1 | 0138864 | -140652 Down |
| Macf1 | 0139149 | 052848 Up |
| Atp6v1b2 | 0139173 | 0299075 Up |
| Ptpn9 | 0139173 | -081255 Down |
| Kat7 | 0139173 | -086764 Down |
| Lrpap1 | 0139378 | -118035 Down |

| Cybrd1 | 0139407 | 2013631 Up |
| --- | --- | --- |
| Rilpl1 | 0139573 | 2473544 Up |
| 3110082I1 | 0139573 | 0852055 Up |
| Rb1 | 0139811 | -135479 Down |
| Arfgef1 | 0139811 | 0582095 Up |
| Jam2 | 0139811 | 1000023 Up |
| Chm | 0139811 | 080268 Up |
| Pdhb | 0139834 | 0895945 Up |
| Khdrbs3 | 0139933 | 0585039 Up |
| Ndufa11 | 0140417 | -04603 Down |
| Rad23a | 0140539 | 1137219 Up |
| Cers4 | 0140596 | 0920203 Up |
| Pts | 0140596 | -063418 Down |
| Pdha1 | 0141011 | 118371 Up |
| Alg5 | 0141018 | -105058 Down |
| Sema4b | 0141018 | -141034 Down |
| Scap | 0141018 | -115123 Down |
| Zfp799 | 0141018 | -115787 Down |
| Mtpap | 0141018 | -111332 Down |
| Paqr7 | 014137 | 1591562 Up |
| Tmem168 | 014137 | 279134 Up |
| Dnal4 | 0141774 | 0807284 Up |
| Ptges3 | 0141835 | 0760727 Up |
| Dzank1 | 0141958 | 1628501 Up |
| Nudt1 | 0141958 | 1861905 Up |
| Rpia | 0141958 | 1982359 Up |
| Leng9 | 0141958 | 2077444 Up |
| Gm16536 | 0141958 | 233866 Up |
| Gskip | 0141958 | 216612 Up |
| Dhx57 | 0141958 | 1989272 Up |
| Samd14 | 0142098 | -12432 Down |
| Zfp651 | 0142172 | -103336 Down |
| BC052040 | 0142172 | 1394391 Up |
| A730056A | 0142172 | 1386399 Up |
| mt-Nd4l | 0142939 | 0523308 Up |
| Ccdc107 | 0143068 | -1133 Down |
| Pak1 | 0143068 | -097718 Down |
| Gcdh | 0143068 | -161883 Down |
| Strbp | 0143283 | 0683145 Up |
| Polr3k | 0143287 | -152795 Down |
| Tceanc2 | 0143633 | -16736 Down |
| Pou3f1 | 0143633 | -311649 Down |
| Mical3 | 0143633 | -14877 Down |
| Lins1 | 0143633 | -110852 Down |
| Gm2115 | 0143633 | -107706 Down |
| Rara | 0143633 | -175845 Down |
| Pigh | 0143633 | -10948 Down |
| Pou6f1 | 0143633 | -114532 Down |
| Mfsd5 | 0143633 | -267123 Down |
| Pkd1 | 0143633 | -185974 Down |

| Bnip1 | 0143633 | -152576 Down |
| --- | --- | --- |
| Ampd3 | 0144132 | 0987805 Up |
| Asna1 | 0144915 | -100656 Down |
| Hmg20b | 0144915 | 0800576 Up |
| Tmem9 | 0145122 | -08246 Down |
| Arl6ip5 | 0145122 | 0902957 Up |
| Ccnh | 0145122 | 0856356 Up |
| Ndnl2 | 0145122 | 1037238 Up |
| Hsdl1 | 0145234 | 1250686 Up |
| Kctd20 | 0145234 | 1437274 Up |
| Ppp2cb | 0145381 | 0547528 Up |
| Park7 | 014612 | -058194 Down |
| Utrn | 0146121 | 3485499 Up |
| Hba-a2 | 0146121 | 3391809 Up |
| Copb1 | 0146157 | -113798 Down |
| Smek2 | 0146405 | 0531518 Up |
| Sh3pxd2b | 0146544 | -04978 Down |
| Dnajc18 | 0146544 | -183815 Down |
| Wdr83 | 0146611 | -221104 Down |
| Srprb | 0146611 | 2089234 Up |
| Ptov1 | 0147788 | 0629613 Up |
| Eea1 | 0148232 | -144803 Down |
| AW011738 | 0148362 | -145633 Down |
| Ttll1 | 0148362 | -214787 Down |
| Psmb8 | 0148362 | -246797 Down |
| Ebp | 0148362 | -26883 Down |
| Ptpn4 | 0148362 | 1351584 Up |
| Wdsub1 | 0148362 | 1078017 Up |
| Nfil3 | 0148362 | 156399 Up |
| Pick1 | 0148362 | 146864 Up |
| Chic1 | 0148362 | 1116922 Up |
| Ndufb2 | 0148445 | 072627 Up |
| Mrpl39 | 0149489 | 1483897 Up |
| Eps15 | 0149706 | 1150493 Up |
| Yes1 | 0149706 | 0779318 Up |
| Mcm3ap | 0149706 | 1442949 Up |
| Lama5 | 0149707 | 2239954 Up |
| Ccdc30 | 0149707 | 3916993 Up |
| Antxr1 | 0149707 | 1992375 Up |
| Slc35e1 | 0149707 | 1952293 Up |
| Mlh1 | 0149707 | 1646457 Up |
| Atad5 | 0149707 | 208071 Up |
| Rubcn | 0149707 | 1760525 Up |
| Ikbkg | 0149707 | 1871696 Up |
| Golim4 | 0149857 | 0260615 Up |
| Nsg1 | 015009 | -143163 Down |
| Ddx46 | 0150215 | -096869 Down |
| Hnrnpa3 | 0150671 | 0703665 Up |
| 9530068E0 | 0150747 | 076277 Up |
| Tmem141 | 0150922 | -155483 Down |

| B3galt1 | 0150922 | -095202 Down |
| --- | --- | --- |
| Phospho2 | 0150922 | -104563 Down |
| Tram1l1 | 0150922 | -291134 Down |
| Nol9 | 0150922 | -199226 Down |
| 2700038G | 0150922 | -15536 Down |
| Dnase2a | 0150922 | -112172 Down |
| Arid3b | 0150922 | -103097 Down |
| Fuca2 | 0150922 | -108918 Down |
| Usp36 | 0150922 | -172972 Down |
| 2700049A0 | 0150922 | -134692 Down |
| Fam210a | 0150922 | -238035 Down |
| Lipa | 0150922 | -211457 Down |
| Gareml | 0150922 | -109141 Down |
| Tbc1d19 | 0151069 | 1356263 Up |
| Rad54l2 | 0151069 | 1201456 Up |
| Gid4 | 0151069 | 1444486 Up |
| Nfe2l1 | 0151069 | 0342888 Up |
| Fam3a | 0151069 | 1718825 Up |
| Uqcc2 | 0151154 | -067358 Down |
| Ift22 | 0151439 | -142706 Down |
| Cmtm4 | 0151439 | -061479 Down |
| Dync1li2 | 0151439 | -116499 Down |
| Timp2 | 0151992 | -116035 Down |
| Ikbkb | 0151999 | 133953 Up |
| Robo2 | 0152141 | -116063 Down |
| Zbtb41 | 0152235 | -093454 Down |
| Sh3glb2 | 0152235 | -083842 Down |
| Pstk | 0152235 | -132627 Down |
| Elk3 | 0152235 | 1316359 Up |
| Mettl1 | 0152235 | -107544 Down |
| Wdr70 | 0152235 | -076305 Down |
| Slitrk2 | 0152235 | 1485948 Up |
| Cltc | 015234 | 0569158 Up |
| Med19 | 0152826 | -127266 Down |
| Mkks | 0152826 | -174586 Down |
| Kalrn | 0152826 | -134806 Down |
| Dpm2 | 0153035 | -075872 Down |
| Trappc3 | 0153035 | 0978469 Up |
| Snrpd3 | 0153053 | 095568 Up |
| Lrig1 | 015308 | 1378607 Up |
| Elp6 | 015308 | 3480568 Up |
| Ascc3 | 015308 | 2547661 Up |
| Tbc1d10a | 015308 | 1610426 Up |
| Nbas | 015308 | 3230644 Up |
| Fam13b | 015308 | 1428939 Up |
| Prrg3 | 015308 | 1581762 Up |
| Pisd | 0153537 | -088043 Down |
| Dpy19l1 | 0153537 | -115525 Down |
| Rnf187 | 0154148 | -065891 Down |
| Marf1 | 0154416 | 1195574 Up |

| Glt8d1 | 0154485 | -088817 Down |
| --- | --- | --- |
| Sema5b | 0154515 | -102397 Down |
| Aimp1 | 0154675 | -121566 Down |
| Tsga10 | 0154755 | -098299 Down |
| 9430038I0 | 0154755 | -094247 Down |
| Cherp | 0154755 | -115606 Down |
| Slc25a38 | 0154755 | -100724 Down |
| Stxbp1 | 0154755 | 1095142 Up |
| Ccdc43 | 0154755 | 1270393 Up |
| Ptcd2 | 0154755 | 0901009 Up |
| Zc3h7b | 0154777 | 1885544 Up |
| Ldoc1l | 0155325 | 0975877 Up |
| Cnn3 | 0155682 | 06116 Up |
| Lsm6 | 015602 | -068557 Down |
| Vangl2 | 0156657 | 0559691 Up |
| Coq2 | 0157081 | 1131718 Up |
| Sptlc2 | 0157081 | 0831354 Up |
| Sacs | 0157081 | 1012949 Up |
| BC026585 | 0157081 | 159815 Up |
| Gle1 | 015733 | -117167 Down |
| Ppil4 | 0157444 | 0727739 Up |
| Rps6 | 0157466 | -046794 Down |
| Id3 | 0157536 | 3336894 Up |
| Mphosph1 | 01576 | -075061 Down |
| Plcl1 | 0157775 | 386424 Up |
| Zfp217 | 0157775 | 1511744 Up |
| Gm14418 | 0157775 | 1646207 Up |
| Eml2 | 0157775 | 1638425 Up |
| 3110040N | 0157775 | 1707485 Up |
| Cyb561d2 | 0157775 | 1834414 Up |
| Slc36a1 | 0157775 | 1473127 Up |
| Kat2a | 0157775 | 1583188 Up |
| Gbe1 | 0157775 | 2493833 Up |
| Taf5 | 0157775 | 2122871 Up |
| Gspt2 | 0157775 | 1853057 Up |
| Foxo4 | 0157775 | 1427518 Up |
| Pmpca | 0158092 | -087452 Down |
| H2-D1 | 0158092 | -345683 Down |
| Rad17 | 0158092 | 1514779 Up |
| Dusp6 | 0158194 | 1567286 Up |
| Gns | 0158194 | -036674 Down |
| Tmem189 | 015849 | -113078 Down |
| Tmem56 | 015849 | -092811 Down |
| Lin37 | 015849 | -125413 Down |
| Zfp938 | 015849 | -10417 Down |
| Slc6a15 | 015849 | -071449 Down |
| Map4k3 | 015849 | -089715 Down |
| 2700046G | 015849 | -093815 Down |
| Gm26853 | 015849 | -073651 Down |
| Atp13a3 | 0158985 | -142722 Down |

| Lars2 | 0159335 | -099783 Down |
| --- | --- | --- |
| Napa | 0159574 | -100493 Down |
| Scfd1 | 0159574 | -050852 Down |
| Rps7 | 0159632 | -027771 Down |
| Zswim6 | 0159669 | -132441 Down |
| Klhdc3 | 0159669 | -155402 Down |
| Nrip1 | 015977 | 0722252 Up |
| Slc25a11 | 015992 | 1478721 Up |
| Ginm1 | 0159974 | 0703324 Up |
| Tnfaip6 | 0160288 | 172803 Up |
| Setd7 | 0160288 | 1385998 Up |
| Mphosph9 | 0160288 | 1295969 Up |
| Homer1 | 0160288 | 1405155 Up |
| Bcdin3d | 0160288 | 1474197 Up |
| Txndc11 | 0160288 | 1364049 Up |
| Uba1 | 016094 | 0607219 Up |
| Prpf18 | 0161356 | -118653 Down |
| Ucp2 | 0161356 | -136475 Down |
| Prkaa1 | 0161356 | -230969 Down |
| Pcnxl3 | 0161356 | -06347 Down |
| Tbc1d9b | 0161356 | 110762 Up |
| Ints2 | 0161356 | 1138883 Up |
| Trappc9 | 0161356 | 1132896 Up |
| Snw1 | 0161702 | -057644 Down |
| Thoc7 | 0161702 | 0764735 Up |
| Yrdc | 0161763 | 1090437 Up |
| Fam104a | 0162374 | 0490237 Up |
| Ap2s1 | 0163107 | 0541123 Up |
| Srrd | 0163225 | -141 Down |
| Bcap29 | 0163225 | 1193962 Up |
| Mrpl49 | 0163272 | 059969 Up |
| Chtop | 0163619 | 0612485 Up |
| Lgr4 | 0163764 | 1195388 Up |
| Bloc1s2 | 0163764 | 1142358 Up |
| Rpl22 | 0163834 | -041879 Down |
| Foxp1 | 0163912 | -083551 Down |
| Dbf4 | 0164119 | -129709 Down |
| Dbp | 0164119 | -140656 Down |
| Sbf1 | 0164119 | -098961 Down |
| Mthfsl | 0164119 | 2419874 Up |
| Spen | 0164483 | -050675 Down |
| Lsg1 | 0164578 | 2638052 Up |
| Rassf1 | 0164952 | 1522394 Up |
| Mllt10 | 0165262 | 07131 Up |
| Rbbp9 | 0165262 | 1057306 Up |
| Pbrm1 | 0165883 | 0885389 Up |
| Rpl14 | 016607 | -038501 Down |
| Zer1 | 0166168 | 1681016 Up |
| Tsacc | 0166168 | 1508003 Up |
| Plxna1 | 0166168 | 1429571 Up |

| 2810454H0 | 0166168 | 1196584 Up |
| --- | --- | --- |
| Sbsn | 0166168 | 14139 Up |
| Col12a1 | 0166168 | 128068 Up |
| Gucd1 | 0166168 | 1142953 Up |
| Nek4 | 0166168 | 1991059 Up |
| Zfpm2 | 0166168 | 1388422 Up |
| Strn | 0166168 | 1518932 Up |
| Sac3d1 | 0166168 | 1316512 Up |
| Mtfr1 | 0166342 | -054687 Down |
| Smad3 | 0166342 | -077445 Down |
| A730017C2 | 0166342 | -034844 Down |
| Cetn2 | 0167037 | -04811 Down |
| Jmjd1c | 0167406 | -065866 Down |
| Grcc10 | 0167433 | -05816 Down |
| Snrpe | 0167686 | -037617 Down |
| Smad1 | 016775 | 1566882 Up |
| Zw10 | 016775 | 2856934 Up |
| D430019H | 016775 | 1813483 Up |
| Chchd1 | 0167783 | -016793 Down |
| Fam102b | 0168029 | 063946 Up |
| Crnkl1 | 0168029 | 0652568 Up |
| Mrpl46 | 0168108 | -084964 Down |
| Rpp14 | 0168108 | -089817 Down |
| Ppm1k | 0168108 | 11863 Up |
| Coa6 | 0168108 | 181405 Up |
| Uck2 | 0168167 | -10166 Down |
| Slc35f6 | 0168167 | -112778 Down |
| Primpol | 0168167 | -090732 Down |
| Cep70 | 0168167 | -10939 Down |
| Syngr1 | 0168167 | -076724 Down |
| Ndfip1 | 0168167 | -107076 Down |
| Lrfn4 | 0168167 | -083635 Down |
| 2610008E1 | 0168167 | 1281509 Up |
| Arhgef9 | 0168167 | 1366724 Up |
| Gm26669 | 0168167 | 1072145 Up |
| Tuba1a | 0168329 | 0276604 Up |
| Psmd4 | 0168873 | -079884 Down |
| Mettl14 | 0169067 | 1471088 Up |
| Snrnp40 | 0169306 | -039141 Down |
| Mpnd | 0169306 | 0915252 Up |
| AU040320 | 0169646 | 144773 Up |
| Grpel2 | 0169646 | 1359138 Up |
| Cdk2ap1 | 0169777 | -055206 Down |
| Pdrg1 | 0170316 | -138303 Down |
| Sdc3 | 0170316 | -105944 Down |
| Ppargc1a | 0170316 | -0897 Down |
| Haus2 | 0170316 | 1814479 Up |
| Bola3 | 0170365 | 1075922 Up |
| Pard6g | 0170365 | 0702092 Up |
| Ssna1 | 017061 | 0778029 Up |

| Hnrnpab | 0170833 | 0517162 Up |
| --- | --- | --- |
| Hnrnph1 | 0171366 | -049041 Down |
| Kdelr2 | 0172047 | 0311862 Up |
| Selt | 0172175 | 0662517 Up |
| Zzz3 | 0172175 | -103127 Down |
| Rps19 | 0172801 | -022595 Down |
| Apoa1bp | 0172919 | -059277 Down |
| Mrps7 | 0172989 | 061553 Up |
| Sorcs2 | 0173143 | 0784357 Up |
| Fam69b | 0173143 | 0337931 Up |
| Fam32a | 0173342 | -033579 Down |
| Cwc27 | 01737 | -117174 Down |
| Dazap1 | 0174157 | 171479 Up |
| Abce1 | 0174157 | -105964 Down |
| Git2 | 0174824 | 0919266 Up |
| Exosc2 | 0174893 | 1346422 Up |
| Gabra4 | 0174893 | 1130385 Up |
| Aste1 | 0174893 | 1711185 Up |
| Rnf217 | 0174893 | 1708988 Up |
| Emid1 | 0174893 | 1434521 Up |
| Sgcd | 0174893 | 1307901 Up |
| Zfp354a | 0174893 | 1888463 Up |
| Nin | 0174893 | 0939743 Up |
| Gm5141 | 0174893 | 1288138 Up |
| Farp1 | 0174893 | 1320056 Up |
| Tfrc | 0174893 | 1388184 Up |
| Kif20b | 0174893 | 1663074 Up |
| Tceanc | 0174893 | 1802127 Up |
| C330027C0 | 0174893 | 3034627 Up |
| Lyn | 0175192 | -19404 Down |
| Kdm4c | 0175192 | -068532 Down |
| Psmb10 | 0175192 | -096038 Down |
| Arid5b | 0175192 | -131381 Down |
| Pias4 | 0175192 | 1383787 Up |
| Patl1 | 0175192 | 103421 Up |
| Rab7 | 017536 | 0711882 Up |
| Tfb1m | 0175469 | 1304394 Up |
| G3bp1 | 0175698 | 0282405 Up |
| Ide | 0175911 | -079876 Down |
| Aak1 | 0175911 | 0805902 Up |
| Safb2 | 0175911 | 1008596 Up |
| Rbck1 | 0176204 | -025112 Down |
| Zfp467 | 0176207 | 1675716 Up |
| Atp6v0e2 | 0176672 | -063234 Down |
| Asap2 | 0176672 | 080596 Up |
| Dlg1 | 0176688 | -102581 Down |
| Ric8 | 0176688 | -092771 Down |
| Col4a1 | 0176688 | 3354429 Up |
| Ndufc2 | 0176716 | 0415982 Up |
| Cox5b | 0176716 | -051787 Down |

| Gm11808 | 0177046 | 017655 Up |
| --- | --- | --- |
| Hadh | 017756 | -055666 Down |
| Fto | 0177689 | 0444053 Up |
| Dph3 | 0177689 | -092832 Down |
| Mycbp2 | 0177689 | 0560206 Up |
| Magi1 | 0177715 | -048981 Down |
| Fundc2 | 0177715 | -070091 Down |
| B9d1 | 0178779 | -098065 Down |
| Nucb1 | 0179431 | -140209 Down |
| Lcmt1 | 0179564 | -079591 Down |
| Clstn1 | 0179564 | 0582918 Up |
| Yaf2 | 0179642 | 0203385 Up |
| Rpl23 | 0179682 | -017517 Down |
| Myo9a | 0179892 | -133156 Down |
| Cab39 | 0180537 | -104705 Down |
| Ubtf | 0180537 | -106196 Down |
| Nadk | 018122 | -101576 Down |
| Wdr60 | 018122 | -063237 Down |
| Rspry1 | 0181539 | 0981304 Up |
| Rrp8 | 018159 | -116352 Down |
| Runx1t1 | 0182433 | -165964 Down |
| S100pbp | 0182433 | -089921 Down |
| Map2k7 | 0182433 | -135955 Down |
| Oxnad1 | 0182433 | -088186 Down |
| Natd1 | 0182433 | 1363951 Up |
| Wars | 0182433 | 0666486 Up |
| Gdpd2 | 0182433 | 118478 Up |
| Pde5a | 0182592 | 1807933 Up |
| Lyrm4 | 0182592 | 0858047 Up |
| Nubp1 | 0183235 | -13573 Down |
| Arhgap12 | 0183235 | -098872 Down |
| Ncaph | 0183451 | 1393589 Up |
| Hectd3 | 0183451 | 1159761 Up |
| Ube3b | 0183451 | 1202843 Up |
| Cops5 | 018365 | 0907285 Up |
| Smarcal1 | 0183954 | 1143582 Up |
| Sdccag8 | 0183954 | 1532011 Up |
| Klhl23 | 0183954 | 1294754 Up |
| Acp6 | 0183954 | 1297156 Up |
| Ift172 | 0183954 | 1347199 Up |
| Pex5 | 0183954 | 0829558 Up |
| Zfp653 | 0183954 | 1408226 Up |
| Cradd | 0183954 | 1148669 Up |
| Slc38a9 | 0183954 | 1378399 Up |
| Eif2ak2 | 0183954 | 1849416 Up |
| Morn4 | 0183954 | 1190622 Up |
| Lsm14a | 0184565 | 0240303 Up |
| Bzw1 | 0184745 | 081672 Up |
| Rnf13 | 018493 | -077003 Down |
| Orc4 | 0185034 | 0396581 Up |

| Zfp60 | 0185034 | 0775444 Up |
| --- | --- | --- |
| Crebrf | 0185301 | -125444 Down |
| Tsnax | 0186069 | 1007543 Up |
| Ddx41 | 0186069 | 0811647 Up |
| Srsf9 | 0186144 | 0618692 Up |
| Cntfr | 0187093 | -045338 Down |
| Ryk | 0187093 | 1360718 Up |
| Atp6v1d | 0187389 | -039145 Down |
| Cdk5rap2 | 0187404 | 0546313 Up |
| Foxo3 | 0187535 | -150484 Down |
| Camsap1 | 0187535 | 0698521 Up |
| Arhgap32 | 0187535 | 1165989 Up |
| Psmc5 | 0188236 | -085414 Down |
| Fam134a | 0188288 | 0448286 Up |
| Zfp386 | 0188437 | -084464 Down |
| Fbxl15 | 0188437 | -088095 Down |
| Phtf1 | 0189124 | -023494 Down |
| Epha4 | 0189861 | 061861 Up |
| Alad | 0189886 | 019883 Up |
| Map2k1 | 0189886 | -108471 Down |
| Spsb4 | 0189886 | -151219 Down |
| Tor1b | 0189894 | -095227 Down |
| Pla2g12a | 0189894 | -147865 Down |
| Tmem218 | 0189894 | -093637 Down |
| Sacm1l | 0189894 | 0610044 Up |
| Abhd6 | 0189894 | 0753869 Up |
| Rpgrip1l | 0189961 | -073158 Down |
| Sft2d3 | 0189961 | 1139286 Up |
| Hiatl1 | 0189961 | 1019256 Up |
| Timm50 | 0190031 | 0985084 Up |
| H1f0 | 0190047 | -033151 Down |
| Ppp1r11 | 0190137 | 0768044 Up |
| Smim15 | 0190278 | -09735 Down |
| Exosc5 | 0190745 | -100604 Down |
| Pitpnc1 | 0190886 | 1188726 Up |
| Hmgb2 | 0190934 | 0651321 Up |
| Mrps35 | 0191312 | -129553 Down |
| N4bp1 | 0191312 | -109592 Down |
| Pcbp4 | 0191457 | 0499529 Up |
| Rpl28 | 0191606 | -020038 Down |
| Itih5 | 0191699 | 098147 Up |
| Trp53i11 | 0191699 | 0931701 Up |
| Cdan1 | 0191699 | 0267616 Up |
| Serinc2 | 0191699 | 1031219 Up |
| Kif22 | 0191699 | 102796 Up |
| Mdm1 | 0191699 | 1035773 Up |
| Trib2 | 0191699 | 1538265 Up |
| Fbxo34 | 0191699 | 0825032 Up |
| Senp5 | 0191699 | 0994388 Up |
| Stradb | 0192447 | -096133 Down |

| Cndp2 | 0192447 | -123769 Down |
| --- | --- | --- |
| Ergic2 | 0192481 | -074638 Down |
| Aggf1 | 0192481 | -073425 Down |
| Mgat2 | 0193354 | -073857 Down |
| Gid8 | 0193354 | 1470894 Up |
| Birc2 | 0193354 | 0429529 Up |
| Nolc1 | 0193354 | 105769 Up |
| Gba | 0193359 | 1167745 Up |
| Ctnnal1 | 0193359 | 078432 Up |
| Whrn | 0193359 | 0807516 Up |
| Aurkb | 0193359 | 0935549 Up |
| Fntb | 0193359 | -021908 Down |
| Bin1 | 0193516 | 0206489 Up |
| Itm2c | 0193532 | 0596697 Up |
| Slc1a2 | 0193847 | -064922 Down |
| Prpf19 | 0193847 | -145405 Down |
| Polr3gl | 019401 | -187414 Down |
| Kcnk10 | 019401 | -075496 Down |
| Nvl | 019401 | 0854531 Up |
| E130308A1 | 019401 | 1100278 Up |
| Nr2f1 | 019401 | 1237557 Up |
| Slc35a1 | 0194783 | -060976 Down |
| Adamts18 | 0194872 | -025005 Down |
| Hipk1 | 0194872 | -16457 Down |
| Gar1 | 0194872 | -091519 Down |
| Fxn | 0195174 | 0479263 Up |
| Dld | 0195808 | 0312181 Up |
| Ubc | 0196217 | -035504 Down |
| Cops8 | 0196222 | -084175 Down |
| Gnb2l1 | 019642 | -021769 Down |
| Thyn1 | 019656 | -052784 Down |
| Dennd2a | 0196867 | -063194 Down |
| Dzip1 | 0196867 | -116057 Down |
| Tppp3 | 0196867 | 1843856 Up |
| Rdm1 | 0197013 | -265024 Down |
| Ddx42 | 0197555 | 1070014 Up |
| Pnpla2 | 0197578 | -090084 Down |
| Aga | 0197578 | -039957 Down |
| Atr | 0197578 | -06097 Down |
| B4galt1 | 0197578 | 0642866 Up |
| Mcat | 0197578 | 097199 Up |
| Dpp9 | 0197578 | 1488693 Up |
| Ildr2 | 0197688 | -106663 Down |
| Trmt13 | 0197688 | -128064 Down |
| Srd5a3 | 0197688 | -141998 Down |
| Eepd1 | 0197735 | 041775 Up |
| Eci2 | 0197741 | 0838898 Up |
| Ppih | 0197841 | 0945752 Up |
| C130071C0 | 0198357 | 0295716 Up |
| Ptn | 019853 | 0382021 Up |

| Cct4 | 0198959 | 0318671 Up |
| --- | --- | --- |
| Ly6e | 0199221 | -29837 Down |
| Ggh | 0199339 | -145153 Down |
| Mrps2 | 0200216 | 1182297 Up |
| Dlx6 | 0200216 | 1099041 Up |
| Mcm5 | 0200216 | 0890737 Up |
| Lrrc14 | 0200216 | 136744 Up |
| Gcat | 0200216 | 0964335 Up |
| Rnf8 | 0200216 | 0880843 Up |
| Suv39h1 | 0200216 | 0688564 Up |
| Anp32a | 0200301 | 0558677 Up |
| Srcin1 | 0200648 | -120196 Down |
| Cxx1b | 0200648 | -088403 Down |
| Ythdf3 | 0200648 | 1036205 Up |
| St8sia2 | 0200648 | 195641 Up |
| Shfm1 | 0201234 | 0389203 Up |
| Hax1 | 0201234 | -0846 Down |
| Slc35d1 | 0201283 | -055878 Down |
| Fam177a | 0201283 | -101794 Down |
| Rps27 | 0201495 | -020316 Down |
| Arl2bp | 0201666 | -101441 Down |
| Golph3 | 0201797 | -080587 Down |
| Rbm7 | 020228 | -126153 Down |
| Klf6 | 0202877 | 0592492 Up |
| Smc4 | 0202944 | 1150731 Up |
| Mprip | 0202944 | 115296 Up |
| Zfp445 | 0203113 | 0365169 Up |
| Tbp | 0203113 | 0700135 Up |
| Vldlr | 0203113 | 0903832 Up |
| Rab12 | 0203233 | -042981 Down |
| Suclg1 | 0203309 | 0770729 Up |
| Brk1 | 0203447 | -068405 Down |
| Sec23ip | 0203955 | 1211865 Up |
| Malat1 | 020409 | 0214034 Up |
| Negr1 | 0204334 | 0567628 Up |
| Dst | 0204334 | -119852 Down |
| Rnpepl1 | 0204334 | -130309 Down |
| Vps39 | 0204334 | -082801 Down |
| 2010320M | 0204334 | -114753 Down |
| Rab14 | 0205019 | -024006 Down |
| Oat | 0205045 | 0490704 Up |
| Irf2bpl | 0205227 | 0492275 Up |
| Tacc2 | 0205486 | 0630814 Up |
| Fam134c | 0205486 | 0875175 Up |
| Pitrm1 | 0205668 | -124526 Down |
| Shisa9 | 0205668 | 1005092 Up |
| Leng8 | 0205952 | 0576266 Up |
| Arl5b | 0205962 | -103042 Down |
| Mast3 | 0205962 | -146417 Down |
| Bst2 | 0205962 | -417791 Down |

| Zfp62 | 0205962 | -125533 Down |
| --- | --- | --- |
| Rab11fip4 | 0205962 | -164956 Down |
| Dip2c | 0205962 | -162699 Down |
| Wdr90 | 0205962 | -183821 Down |
| Fbxl17 | 0205962 | -133351 Down |
| Glo1 | 0206745 | -084925 Down |
| Tcf12 | 020704 | -038442 Down |
| Samm50 | 0207219 | -108867 Down |
| Mgea5 | 0207219 | -045026 Down |
| Kcne1l | 0207219 | -073796 Down |
| Dpysl5 | 0207219 | 0648311 Up |
| Dab2ip | 0207451 | -144104 Down |
| Impa1 | 0207451 | -1036 Down |
| Wdr46 | 0207451 | -113265 Down |
| Leo1 | 0207451 | 1107807 Up |
| Cul4b | 0207451 | 2463659 Up |
| Cog4 | 0207747 | 071254 Up |
| Rsbn1 | 0207747 | -121771 Down |
| Tm2d1 | 0207917 | -051576 Down |
| Sdhaf4 | 0208282 | 1133958 Up |
| Ormdl2 | 0208283 | 1088139 Up |
| Trim28 | 020887 | 0741291 Up |
| Tmem87a | 0209006 | 0985279 Up |
| Glipr2 | 0209006 | 0489874 Up |
| Cox8a | 0209054 | -03121 Down |
| Exoc4 | 0209105 | -127266 Down |
| Rpl13a | 0209249 | -016301 Down |
| Ivd | 0209705 | 1507604 Up |
| Hnrnpr | 0209768 | 088297 Up |
| Emd | 0210149 | -092643 Down |
| Adgrl2 | 0210203 | -071913 Down |
| Ptp4a3 | 0210203 | -102491 Down |
| Gfer | 0210203 | -110679 Down |
| Rnf145 | 0210203 | 0743198 Up |
| Acat1 | 0210424 | 0465653 Up |
| Mcl1 | 0210545 | -094671 Down |
| Caml | 0210662 | -016915 Down |
| Ubxn2b | 0211226 | -064441 Down |
| C2cd3 | 0211226 | -056812 Down |
| Vps26b | 0211226 | -054743 Down |
| Spa17 | 0211226 | -069136 Down |
| Gm561 | 0211226 | -054857 Down |
| Atp6v0a2 | 021133 | 1248534 Up |
| Gm26735 | 0211469 | -11511 Down |
| Lmo4 | 0211507 | 0243832 Up |
| Rps26 | 0211846 | -022092 Down |
| Ube2c | 0211851 | -059699 Down |
| Usp37 | 0211851 | -125127 Down |
| Tcp11l2 | 0211851 | -10814 Down |
| Mdm4 | 0212035 | 0925216 Up |

| Pank3 | 0212035 | 0568836 Up |
| --- | --- | --- |
| Elof1 | 0212073 | -090952 Down |
| Gtf2e2 | 0213103 | 0249683 Up |
| Dis3l | 021322 | 0312695 Up |
| Znhit2 | 0213288 | 0631784 Up |
| Creb5 | 0213586 | -066188 Down |
| Npl | 0213622 | 0160563 Up |
| Slc39a3 | 0213622 | -049958 Down |
| Rapgef2 | 0213622 | 0255729 Up |
| Gpi1 | 0213707 | -087542 Down |
| Ntm | 0214331 | 0378098 Up |
| Myo6 | 0214331 | -058879 Down |
| Zcchc3 | 0214411 | -117852 Down |
| Bccip | 0214419 | 10855 Up |
| Pkp4 | 0214486 | -178521 Down |
| Kbtbd4 | 0214486 | -228287 Down |
| Slc20a1 | 0214486 | -113221 Down |
| Slc9a8 | 0214486 | -10682 Down |
| Abcb8 | 0214486 | -107377 Down |
| Mir124a-1 | 0214486 | -124952 Down |
| Mrpl33 | 0215962 | 0891659 Up |
| Myl9 | 0216495 | -117138 Down |
| Mrpl35 | 0216495 | -087081 Down |
| Bri3bp | 0216506 | -09289 Down |
| Mob1b | 021679 | -135432 Down |
| Braf | 021679 | -044834 Down |
| Mplkip | 021679 | 0527154 Up |
| Ikzf5 | 0216912 | 1249372 Up |
| Ddx39b | 02172 | 0701793 Up |
| Syt11 | 0217427 | 0463802 Up |
| Mmp14 | 0217516 | 0501094 Up |
| Raf1 | 0217524 | -051915 Down |
| Hdac2 | 0217927 | 0820287 Up |
| Traf7 | 0218543 | -039874 Down |
| Rtcb | 0218543 | -094422 Down |
| Man1c1 | 0218684 | -045786 Down |
| Rnf170 | 0218684 | -039946 Down |
| Pim3 | 0218684 | -028531 Down |
| Rcan1 | 0218684 | 1272579 Up |
| Trappc8 | 0218684 | 0955928 Up |
| Nfia | 0219283 | 033059 Up |
| Stmn2 | 0219328 | 0487894 Up |
| Grk6 | 0219352 | 3231184 Up |
| Shd | 0219565 | 0779645 Up |
| Smg7 | 0219565 | -076901 Down |
| Sirpa | 0219565 | -102828 Down |
| Sigmar1 | 0219565 | -244365 Down |
| Vwa5a | 0219565 | -063211 Down |
| Adsl | 0219565 | -087715 Down |
| Bcas2 | 0219583 | 056746 Up |

| Alyref | 0219781 | 1478467 Up |
| --- | --- | --- |
| Tcf25 | 0220249 | -043694 Down |
| Rbm15b | 0220428 | -025442 Down |
| Cnrip1 | 0220428 | 0997435 Up |
| Ranbp1 | 0220435 | 0393611 Up |
| Baz2b | 0220504 | 0441041 Up |
| Stk39 | 0220584 | 0400804 Up |
| Tmem198b | 0220584 | 1941249 Up |
| Pum3 | 022067 | -109649 Down |
| Fbxw5 | 0221554 | -087201 Down |
| Samd1 | 0221554 | -048022 Down |
| Tmem131 | 0221554 | 0905043 Up |
| Aif1l | 0221554 | 0895402 Up |
| Top3b | 0221988 | 0325917 Up |
| Cit | 0221988 | -021099 Down |
| Ubxn6 | 0222443 | -08183 Down |
| Ralbp1 | 0222443 | -041647 Down |
| Fam216a | 0222443 | 0401997 Up |
| Ggnbp2 | 0222822 | -064729 Down |
| Zbtb8os | 0223001 | 0627311 Up |
| Ppil1 | 0223001 | 1025548 Up |
| Shisa5 | 022326 | -319363 Down |
| Fem1a | 022326 | -129561 Down |
| Zak | 022326 | -132229 Down |
| Mafk | 022326 | 1896729 Up |
| Smim12 | 0223525 | 1495414 Up |
| Fnbp1l | 0224318 | 1026362 Up |
| Hspa4l | 0224543 | 0589692 Up |
| Khsrp | 0224543 | -078884 Down |
| Ndufb10 | 0225088 | -047394 Down |
| Rpl19 | 0225289 | -038636 Down |
| Knop1 | 0226183 | -038954 Down |
| Utp14b | 0226328 | 1176044 Up |
| Igfbp2 | 0226638 | 0888907 Up |
| Zeb2 | 022676 | 0611316 Up |
| Dnajc24 | 0226805 | -060888 Down |
| Ghitm | 0227036 | 0590425 Up |
| Ccs | 0227058 | -067924 Down |
| 1810022K0 | 0227174 | -069675 Down |
| Zfp553 | 022748 | -120182 Down |
| Pih1d2 | 022748 | -080937 Down |
| Utp6 | 022748 | -126416 Down |
| Agtpbp1 | 022748 | -11722 Down |
| Mterf4 | 0227546 | 1910256 Up |
| Rnaseh1 | 0227546 | 1179835 Up |
| Gprasp2 | 0227546 | 112951 Up |
| Cetn3 | 0227877 | 0343059 Up |
| Sdccag3 | 0228199 | -073602 Down |
| Itgb1 | 0228229 | 0378562 Up |
| Pank2 | 0228858 | -057698 Down |

| Tnik | 0228858 | 0590635 Up |
| --- | --- | --- |
| Ncaph2 | 0228858 | 1211767 Up |
| Otx2 | 0228867 | 1034661 Up |
| Chchd6 | 0229336 | 1272197 Up |
| Rlbp1 | 0230408 | -034734 Down |
| Sephs1 | 0230408 | 2184166 Up |
| Tm9sf3 | 023069 | 0405038 Up |
| Vgll4 | 0230725 | -042632 Down |
| Cldnd1 | 0230725 | -032571 Down |
| Mt2 | 0231145 | 0679089 Up |
| Idi1 | 0231498 | 0474856 Up |
| Glud1 | 02315 | 0401739 Up |
| Unc50 | 0231618 | 0322655 Up |
| Ccdc181 | 0232289 | -081355 Down |
| Efna4 | 0232289 | -16614 Down |
| Osgin2 | 0232289 | -115809 Down |
| Wdr78 | 0232289 | -086694 Down |
| Ctsc | 0232289 | -159025 Down |
| Prcp | 0232289 | -153738 Down |
| Mis12 | 0232289 | -085803 Down |
| Mroh1 | 0232289 | -080493 Down |
| Fam53c | 0232289 | -293222 Down |
| 1810030O | 0232289 | -090804 Down |
| Med14 | 0232289 | -080962 Down |
| Gm16586 | 0232289 | -133208 Down |
| Hivep2 | 0232289 | 2008219 Up |
| Elp4 | 0232728 | -107301 Down |
| 0610012G | 0232812 | -107168 Down |
| Acads | 0233516 | -048788 Down |
| Uqcr10 | 0233529 | -050767 Down |
| Rab11b | 0233529 | 0514798 Up |
| Sde2 | 0234162 | 0760245 Up |
| C2cd5 | 0234162 | 0942895 Up |
| Trim26 | 0234162 | 1282505 Up |
| Bola2 | 0234165 | -060031 Down |
| Ccni | 02342 | 0578013 Up |
| Mtch1 | 0234772 | 0353694 Up |
| Ilkap | 0235325 | 0771199 Up |
| Metap1d | 0235325 | 0755781 Up |
| Rab3gap1 | 0235325 | -068103 Down |
| Ldhb | 023549 | 0280872 Up |
| Tubgcp3 | 0235595 | -126644 Down |
| Ebag9 | 0235595 | -085137 Down |
| Cadm2 | 0235734 | 0499377 Up |
| Mtmr14 | 0235956 | 1066398 Up |
| Nab1 | 0236165 | 0264702 Up |
| Clptm1 | 0236332 | -070687 Down |
| Nicn1 | 0236332 | -062862 Down |
| Dhx29 | 0236332 | -058279 Down |
| Gmpr2 | 0236332 | -082587 Down |

| Rbfa | 0236332 | -072936 Down |
| --- | --- | --- |
| Mgst3 | 0236406 | -120914 Down |
| Ppp5c | 0236406 | 1287771 Up |
| Immp1l | 0236653 | -093614 Down |
| Zfp148 | 023722 | -068933 Down |
| Gna13 | 023722 | 0396427 Up |
| Pqbp1 | 0237441 | -031334 Down |
| Apoo | 0237441 | 1870011 Up |
| Cstb | 023748 | -080422 Down |
| Zfp207 | 023748 | 029673 Up |
| Swi5 | 0238014 | 0389882 Up |
| Cd81 | 0238815 | 0538936 Up |
| Dnaja1 | 0238986 | 0382404 Up |
| Tiprl | 0239583 | -117538 Down |
| Tmod3 | 0239787 | -026701 Down |
| Sdhaf1 | 0239787 | 2177569 Up |
| Snrnp48 | 0240262 | -075167 Down |
| Cops4 | 0240923 | -017304 Down |
| Rp9 | 0240987 | 0459027 Up |
| Tsc1 | 0241574 | -107345 Down |
| D930016D | 0241574 | -089654 Down |
| 6330418K0 | 0241574 | -100027 Down |
| Foxred1 | 0241574 | -097648 Down |
| Tmem17 | 0241574 | -098991 Down |
| Kctd12 | 0241574 | -147443 Down |
| Trib1 | 0241574 | -109628 Down |
| Pigq | 0241574 | -075665 Down |
| Atl3 | 0241574 | -149596 Down |
| Sec63 | 024163 | 0692343 Up |
| Gabarap | 0241696 | -021093 Down |
| Nmd3 | 0242185 | 102039 Up |
| Ranbp2 | 0242185 | 2413822 Up |
| Cpd | 0242185 | 1009623 Up |
| Sos1 | 0242185 | 0877287 Up |
| Gatad2a | 0242258 | 038502 Up |
| Gzf1 | 0242607 | 0742222 Up |
| Rprd1b | 0242607 | 1870542 Up |
| Bnip3 | 0242607 | 1249075 Up |
| Clip3 | 0243166 | -076262 Down |
| Zbtb22 | 0243543 | 0177178 Up |
| Emg1 | 0243548 | -07456 Down |
| Eif4b | 0243548 | -073668 Down |
| N6amt1 | 0243626 | -173211 Down |
| Gm26917 | 0243626 | 1298568 Up |
| Lrrc4b | 0243684 | -031582 Down |
| S100a11 | 0243684 | -019898 Down |
| Golga3 | 0243914 | -074297 Down |
| Ankrd26 | 0243914 | -079418 Down |
| Sorcs1 | 0243914 | -082177 Down |
| Camk1d | 0243976 | -107009 Down |

| Hip1 | 0243976 | -063753 Down |
| --- | --- | --- |
| Gper1 | 0243976 | -074903 Down |
| Zc3h18 | 0243976 | -100529 Down |
| Ank | 0243976 | -076976 Down |
| Gnb1 | 0244382 | 0418949 Up |
| Nop10 | 0244551 | -071484 Down |
| Gab1 | 0244623 | 0964983 Up |
| Ndufs6 | 0245129 | 0634316 Up |
| Stt3a | 0246007 | -089748 Down |
| Med4 | 0246037 | 0741833 Up |
| Tprgl | 024606 | 0492616 Up |
| Hnrnpul1 | 0246542 | -068807 Down |
| Zfp292 | 0246821 | -118383 Down |
| Dnajc2 | 0247011 | 0648005 Up |
| Gtf3c1 | 0247145 | 0979845 Up |
| Dhx40 | 0247145 | 081126 Up |
| Naa38 | 0247404 | 0470352 Up |
| Smarcc2 | 0247404 | 1771867 Up |
| Ufm1 | 0248685 | 0188309 Up |
| Cenpv | 0248907 | 0508031 Up |
| Whsc1 | 0249217 | 1028703 Up |
| Ing4 | 0249309 | -139092 Down |
| Spred1 | 0249943 | -047759 Down |
| Parp1 | 0249943 | 0581411 Up |
| Srp9 | 0249985 | 0840049 Up |
| Ewsr1 | 0250031 | -042146 Down |
| Smek1 | 0250372 | 045707 Up |
| Plrg1 | 02504 | 1160964 Up |
| Cox7a2 | 025047 | -057181 Down |
| Canx | 0251079 | 0571773 Up |
| Zcrb1 | 0251079 | 0921518 Up |
| Ptpn18 | 0251117 | -12899 Down |
| Atg4b | 0251117 | -097651 Down |
| Gtpbp6 | 0251117 | -074537 Down |
| 2310022A1 | 0251117 | -100125 Down |
| Pepd | 0251117 | -113225 Down |
| Akap12 | 0251117 | 074038 Up |
| B4galt7 | 0251117 | -100828 Down |
| Ttc38 | 0251117 | -093425 Down |
| Cdc42ep2 | 0251117 | -054249 Down |
| Zfp618 | 0251117 | 1709276 Up |
| Znrf2 | 0251117 | 1514713 Up |
| Emp1 | 0251117 | 1585979 Up |
| Katna1 | 0251556 | -102219 Down |
| Rcor1 | 0251556 | -139777 Down |
| Pink1 | 025169 | -03438 Down |
| Prkd3 | 025169 | 0603907 Up |
| 5031425E2 | 0251793 | -073764 Down |
| Gpm6a | 025188 | -043943 Down |
| Nphp1 | 0251957 | 0761802 Up |

| Atg16l1 | 0252436 | -14045 Down |
| --- | --- | --- |
| Ethe1 | 0252436 | -108782 Down |
| Lca5 | 0252436 | -050479 Down |
| Ddhd1 | 0252436 | -068682 Down |
| Zfp414 | 0252436 | -065279 Down |
| Akirin2 | 0253093 | -053282 Down |
| Yap1 | 0253429 | 1220324 Up |
| Srr | 0253429 | 1538203 Up |
| Pcx | 0253429 | 1043274 Up |
| Tuba1c | 0253429 | -224519 Down |
| Ndufb8 | 0253569 | 0280459 Up |
| Oaz1 | 0253736 | -022825 Down |
| Tceb2 | 0253736 | -025726 Down |
| Rpl27a | 0253933 | -034122 Down |
| Rplp0 | 0253939 | -023158 Down |
| mt-Atp8 | 025394 | -069257 Down |
| Tsc22d3 | 0254164 | -094605 Down |
| Fam204a | 0254952 | 0854869 Up |
| Rundc3a | 0255945 | 0371994 Up |
| Eif3k | 0256224 | -026048 Down |
| Naa35 | 0256271 | -042293 Down |
| Commd2 | 0256313 | 0668985 Up |
| Snrpf | 025644 | -019083 Down |
| Tmeff1 | 0257566 | -093116 Down |
| Tpgs1 | 0257566 | 0601945 Up |
| Dpysl3 | 0258051 | -016693 Down |
| Trip12 | 0258243 | 1446987 Up |
| Cachd1 | 0258808 | 0929241 Up |
| Gusb | 0259041 | 085035 Up |
| AU019823 | 0259041 | -035563 Down |
| Smpd1 | 0259041 | 0443734 Up |
| Faim | 0259119 | -040969 Down |
| Bach2 | 0259443 | -050969 Down |
| Irf2 | 0259443 | 0150136 Up |
| Shprh | 0259443 | -062237 Down |
| Fundc1 | 0259443 | -053827 Down |
| Dnajb9 | 0259443 | 1963793 Up |
| Atf4 | 0259476 | 0299792 Up |
| Tlk2 | 0259752 | -131777 Down |
| Ldlrad3 | 0259782 | -057171 Down |
| Wdr74 | 0259782 | 1346283 Up |
| Srgap2 | 026023 | 0495281 Up |
| Mpp5 | 026023 | 0451748 Up |
| Atxn10 | 0260266 | -025514 Down |
| Abi1 | 0260611 | 0480704 Up |
| Kif21b | 026092 | -059403 Down |
| Hsd17b7 | 026092 | -082351 Down |
| Fat4 | 026092 | -062855 Down |
| Plod1 | 026092 | -057125 Down |
| Rgs10 | 026092 | -135199 Down |

| Gan | 026092 | -075416 Down |
| --- | --- | --- |
| Map2k6 | 026092 | -086684 Down |
| Cln5 | 026092 | -043735 Down |
| Rbm14 | 026092 | -070207 Down |
| Lrp2 | 026092 | 1727575 Up |
| Kat8 | 026092 | 1228768 Up |
| Rora | 026092 | 1617911 Up |
| Sfmbt1 | 026092 | 1439213 Up |
| Fam188a | 026092 | 1236838 Up |
| Smo | 0260977 | -059849 Down |
| Tmem203 | 0261164 | -072557 Down |
| Tmem127 | 0261164 | -0691 Down |
| Cdc14a | 0261164 | -063176 Down |
| Fndc4 | 0261164 | -085903 Down |
| Fam53a | 0261164 | -092878 Down |
| Zik1 | 0261164 | -026779 Down |
| AI413582 | 0261164 | -175773 Down |
| Atp5l | 026129 | -029788 Down |
| Mbnl1 | 0261321 | -049728 Down |
| Drg2 | 0261321 | -087672 Down |
| Copz1 | 0261564 | 0581039 Up |
| Nbr1 | 0261564 | 1191858 Up |
| Eif3g | 0261892 | -048487 Down |
| Mbtd1 | 0262241 | -06028 Down |
| Cox5a | 0262293 | 0373148 Up |
| Siva1 | 0262403 | 0719893 Up |
| Tanc1 | 0262496 | 1384512 Up |
| Ccdc9 | 0262496 | 1102666 Up |
| Plekhb1 | 0262496 | 1221115 Up |
| Ccdc71 | 0262496 | 1008666 Up |
| Dopey1 | 0262496 | 2444189 Up |
| Lztr1 | 0262496 | -11287 Down |
| DHRSX | 0262496 | -12249 Down |
| U2surp | 0262928 | 0601754 Up |
| Rnd3 | 0263476 | 0479529 Up |
| Sec24c | 0263918 | 1303292 Up |
| Brwd1 | 0264542 | 087456 Up |
| Pfkl | 0264542 | -078648 Down |
| Mzt2 | 0264552 | -048569 Down |
| Sqstm1 | 0264903 | -058096 Down |
| Cited2 | 0264903 | 1235588 Up |
| Pfn1 | 0265092 | -061879 Down |
| Atp1b1 | 0265107 | -040003 Down |
| Luzp1 | 0265398 | -07818 Down |
| Arap2 | 0265398 | -074577 Down |
| Hist3h2ba | 0265577 | 1030799 Up |
| H1fx | 0265976 | -052185 Down |
| Akt1s1 | 0266126 | -095209 Down |
| Ylpm1 | 0266126 | 0605075 Up |
| Tmx2 | 0266997 | 0798828 Up |

| Gyg | 0267082 | -054827 Down |
| --- | --- | --- |
| D10Jhu81e | 0267082 | -088088 Down |
| Ifitm2 | 0267082 | 0552063 Up |
| Smad2 | 0267082 | 1734839 Up |
| Trrap | 0267304 | -055856 Down |
| Dis3l2 | 0267409 | 0742166 Up |
| Gabpb1 | 0267409 | 0664843 Up |
| Pdlim5 | 0267409 | 0825545 Up |
| Caap1 | 0267409 | 1727221 Up |
| Asb3 | 0267409 | 0673107 Up |
| Sesn1 | 026813 | -109346 Down |
| Palm | 026813 | -086545 Down |
| Trim2 | 0268294 | 0159288 Up |
| Txndc9 | 0268349 | -039945 Down |
| Larp4b | 0268616 | 0304951 Up |
| Cox19 | 0268616 | -099584 Down |
| D030056L2 | 0268616 | -027754 Down |
| Casp8ap2 | 0268616 | 0902454 Up |
| Yy1 | 0268906 | -051825 Down |
| Cox20 | 0268906 | 0388659 Up |
| Sptan1 | 0268926 | 034189 Up |
| Atg3 | 0268926 | 0905664 Up |
| Ppm1g | 0269285 | 0549299 Up |
| Nenf | 026939 | -078852 Down |
| Scg5 | 026939 | -067374 Down |
| Smim14 | 0269581 | -120428 Down |
| Sec31a | 0269581 | -031968 Down |
| Gprasp1 | 0269618 | 0660757 Up |
| Eif4e | 0269895 | 0642701 Up |
| Ermard | 0270099 | -030145 Down |
| Copa | 0270704 | -052239 Down |
| Klhdc2 | 0270704 | 0902073 Up |
| Tmem14a | 0270985 | -057123 Down |
| Ccdc97 | 0270985 | -073306 Down |
| Chst2 | 0270985 | -10116 Down |
| Tmem199 | 0270985 | -070795 Down |
| Rhot2 | 0270985 | -077855 Down |
| Insig2 | 0270985 | 0946413 Up |
| Dpp6 | 0270985 | 1243415 Up |
| E130006D0 | 0270985 | 0898818 Up |
| Pex12 | 0270985 | 0947644 Up |
| Commd5 | 0270985 | 101761 Up |
| E4f1 | 0270985 | 1231777 Up |
| Rsph1 | 0270985 | 111851 Up |
| Ncl | 0271307 | 0525934 Up |
| Hspe1 | 0271307 | -031926 Down |
| Rabep1 | 0271528 | -052823 Down |
| Smc5 | 0271528 | 100904 Up |
| Dhx32 | 0271703 | 0595986 Up |
| Fastkd2 | 0271784 | 1108193 Up |

| Nup35 | 0271784 | 1034002 Up |
| --- | --- | --- |
| Adprhl2 | 0271784 | 2234114 Up |
| Slc37a3 | 0271784 | 1313753 Up |
| Haus8 | 0271784 | 0955073 Up |
| Brms1l | 0271784 | 1313315 Up |
| Zfp768 | 0271784 | -208842 Down |
| Lcor | 0271784 | -1556 Down |
| Aasdhppt | 0271803 | 0743345 Up |
| Nde1 | 0272388 | 1092999 Up |
| Thap4 | 027273 | 1491535 Up |
| Gpr27 | 027273 | 1799943 Up |
| Tmem161a | 027273 | 1610308 Up |
| Gm11713 | 027273 | 1958542 Up |
| Gtpbp8 | 027273 | 2177677 Up |
| Cltb | 0273313 | 0855406 Up |
| Tns1 | 0273313 | -041794 Down |
| Htt | 0273313 | -044571 Down |
| Mrps30 | 0273313 | -100534 Down |
| Zbtb12 | 0273313 | -101965 Down |
| Ptpn2 | 0273313 | -101966 Down |
| Unc119b | 0273313 | 0898491 Up |
| Wbp5 | 0273717 | 0216083 Up |
| Srp72 | 027412 | -032803 Down |
| Cript | 027412 | -140227 Down |
| Med10 | 027412 | -060464 Down |
| Sh3gl1 | 0274874 | 0694883 Up |
| Ccdc112 | 0274874 | 015257 Up |
| Zzef1 | 0275119 | 1043199 Up |
| 2410089E0 | 0275119 | 1042281 Up |
| Mut | 0275119 | 0878483 Up |
| Rps13 | 027524 | -035323 Down |
| Surf4 | 027548 | -060716 Down |
| Gatad1 | 0275727 | -057272 Down |
| Pbdc1 | 0275727 | 0580265 Up |
| Psph | 0276039 | 0756925 Up |
| Arl5a | 027605 | 09237 Up |
| Rnf44 | 027605 | 0689447 Up |
| Irf3 | 0276205 | 0648772 Up |
| B3gat3 | 0276284 | -052297 Down |
| Arhgef10l | 0276284 | 1039032 Up |
| Erc1 | 0276284 | 0640245 Up |
| Rbbp7 | 0276421 | 0335297 Up |
| Diaph2 | 0276693 | -116541 Down |
| Myl12b | 0276827 | 0440916 Up |
| Scand1 | 0276845 | 0642883 Up |
| Zc3h14 | 0278534 | -04441 Down |
| Rab8a | 027861 | 0673085 Up |
| Kbtbd11 | 027864 | -078357 Down |
| C1d | 0278763 | 0604469 Up |
| 11-Sep | 0279094 | 0738932 Up |

| Nr2c2 | 027919 | 0956957 Up |
| --- | --- | --- |
| Mgrn1 | 027924 | -032552 Down |
| Guk1 | 028013 | 0803304 Up |
| Atp5k | 0280482 | 0192117 Up |
| Suco | 0281043 | 2391122 Up |
| Wtap | 0281278 | 0413673 Up |
| Rab3gap2 | 0281295 | 0577916 Up |
| Rpap1 | 0281295 | 0827848 Up |
| Rbsn | 0281295 | 0835337 Up |
| Cdca3 | 0281295 | 1047967 Up |
| Mki67 | 0281295 | 0657509 Up |
| Sumf2 | 0281295 | -120915 Down |
| Zfp398 | 0281295 | -153189 Down |
| Cwf19l2 | 0281295 | -166277 Down |
| Eif2d | 0281312 | -056904 Down |
| Kcnip3 | 0281312 | -062987 Down |
| Dhrs3 | 0281312 | -050749 Down |
| Vps33a | 0281312 | -047372 Down |
| Shkbp1 | 0281312 | -099036 Down |
| Tmtc3 | 0281312 | -172746 Down |
| Prkcd | 0281312 | -067432 Down |
| Usp40 | 0281312 | 1426439 Up |
| Rell1 | 0281312 | 1156653 Up |
| Ncam2 | 0281312 | 1346552 Up |
| Sf3b3 | 0281388 | -158082 Down |
| 2210016F1 | 0281388 | -055658 Down |
| Nfkb1 | 0281388 | 1322317 Up |
| Igsf8 | 0281847 | -015972 Down |
| Utp3 | 0281847 | -05394 Down |
| Nt5c3b | 0281847 | -092017 Down |
| Smap1 | 0282238 | -06201 Down |
| Phb | 0282644 | -044793 Down |
| Commd8 | 0282644 | 1487674 Up |
| Dut | 028269 | -032885 Down |
| Ugp2 | 028269 | 0638183 Up |
| Dnajc1 | 028269 | -06306 Down |
| Sccpdh | 0282819 | 1082164 Up |
| Ykt6 | 0282819 | -050776 Down |
| Ufd1l | 0282819 | 0529328 Up |
| Meaf6 | 0282933 | 0640816 Up |
| Eprs | 0283053 | -066712 Down |
| Arfip2 | 0283409 | 0984001 Up |
| Dtnbp1 | 0283409 | -046982 Down |
| Polr2d | 0283623 | 0946601 Up |
| Rnf166 | 0283921 | -194717 Down |
| Cmtm7 | 0283921 | -264581 Down |
| Pvrl2 | 0283921 | -107637 Down |
| Arfrp1 | 0284064 | 0186993 Up |
| Atp6v1h | 0284064 | -111961 Down |
| Lekr1 | 0284312 | 1530387 Up |

| Mks1 | 0284312 | 1199735 Up |
| --- | --- | --- |
| H2-DMa | 0284312 | 2285197 Up |
| Fbxl3 | 0284797 | -036621 Down |
| Actn4 | 0285196 | 0257003 Up |
| Gtf2h5 | 028531 | -041718 Down |
| Uqcrb | 0286065 | 0459284 Up |
| Eif3l | 028641 | 0518631 Up |
| Zmiz1 | 0286632 | -051633 Down |
| Dnm1l | 0287317 | 061958 Up |
| Tnfaip8 | 0287772 | -063577 Down |
| Sema6a | 0288414 | 0487285 Up |
| Nnt | 028859 | 0337445 Up |
| Fos | 0288883 | 0421973 Up |
| Gigyf1 | 0289317 | -041266 Down |
| Cebpzos | 0289317 | -057719 Down |
| Gclm | 0289317 | 0547125 Up |
| Ppa1 | 0289573 | 0563397 Up |
| Ctdspl2 | 0289623 | -09406 Down |
| Rragc | 0289623 | -089901 Down |
| Slain2 | 0289623 | -03593 Down |
| Mrps11 | 0289623 | -048676 Down |
| Ip6k1 | 0289623 | -063137 Down |
| Acaa1a | 0289793 | -034906 Down |
| Ice1 | 0290919 | -050387 Down |
| Fam174a | 0290919 | 0946987 Up |
| Fmnl2 | 0290919 | 0998753 Up |
| Arfip1 | 0291029 | 0843793 Up |
| Ipo4 | 0291029 | -116694 Down |
| Cdk14 | 0291335 | 02199 Up |
| Grb2 | 0291335 | -038052 Down |
| Nemf | 0291335 | -160127 Down |
| Atp5b | 0291493 | -04977 Down |
| Rpl36 | 0291542 | -01909 Down |
| Cxxc4 | 0291591 | -06828 Down |
| Rab2b | 0291865 | 071917 Up |
| Hsd17b11 | 0291903 | -053506 Down |
| Spr | 0291903 | -046131 Down |
| Cpsf1 | 0291903 | -035157 Down |
| Tm7sf2 | 0291903 | -02234 Down |
| 1110034G | 0291903 | 146912 Up |
| Hilpda | 0291903 | 2986365 Up |
| Ints4 | 0291903 | 1110017 Up |
| Gpr83 | 0291903 | 1230343 Up |
| Apba3 | 0291903 | 3375165 Up |
| Rnf113a2 | 0291903 | 2233054 Up |
| Tdrd3 | 0291903 | 0965894 Up |
| H2afj | 0291915 | 0430655 Up |
| Mxi1 | 0291915 | -048625 Down |
| Smc1a | 0291961 | -098436 Down |
| Ndufb6 | 0291961 | -063176 Down |

| Arfgap2 | 029324 | 0566071 Up |
| --- | --- | --- |
| Wdr45b | 029324 | 0796755 Up |
| Parp2 | 0293344 | 1181445 Up |
| Ccpg1 | 0293486 | -041218 Down |
| Ndufa13 | 0293508 | -022608 Down |
| Ralb | 0294372 | -180013 Down |
| Slc25a20 | 0294372 | -067144 Down |
| Sertad2 | 0294372 | -06826 Down |
| Trabd | 0294383 | 0715501 Up |
| Pea15a | 0294477 | -043067 Down |
| Yipf5 | 0294627 | -047854 Down |
| Ptx3 | 0294627 | 0782499 Up |
| Fau | 0294849 | -047908 Down |
| Gorab | 0295137 | -137195 Down |
| 5031439G | 0295137 | -157365 Down |
| 4932438H2 | 0295137 | -10697 Down |
| Cdc42ep3 | 0295137 | -171494 Down |
| Nfat5 | 0295237 | 1046228 Up |
| Ptpra | 0295268 | -078474 Down |
| Bod1l | 029567 | 021935 Up |
| Zfp608 | 0295682 | -035194 Down |
| Ctr9 | 0295682 | 0389985 Up |
| Atp1a2 | 0296021 | 0722809 Up |
| Lman2l | 0296157 | -080885 Down |
| Creb3 | 0296157 | -24763 Down |
| Serpine2 | 0296222 | 2818761 Up |
| Ptges2 | 0296222 | 1562248 Up |
| Usp50 | 0296222 | 1233398 Up |
| Ninl | 0296222 | 1598999 Up |
| Mafb | 0296222 | 3011526 Up |
| Siah2 | 0296222 | 1217485 Up |
| Usp45 | 0296222 | 2147526 Up |
| Acot7 | 0296222 | 1645243 Up |
| Alkbh2 | 0296222 | 1207232 Up |
| Pitpnm2 | 0296222 | 1863527 Up |
| Nucb2 | 0296222 | 2241569 Up |
| Gga2 | 0296222 | 1018432 Up |
| Mmp11 | 0296222 | 1438614 Up |
| Zfp58 | 0296222 | 1350691 Up |
| Crim1 | 0296222 | 1262506 Up |
| A430046D | 0296222 | 36071 Up |
| Atraid | 029664 | -016062 Down |
| 1110001J0 | 0296897 | 0363507 Up |
| Emc4 | 0296918 | -048504 Down |
| Bcl11a | 0296918 | -082674 Down |
| 1110032A0 | 0296918 | 0866762 Up |
| Xpr1 | 0296918 | -07605 Down |
| Stx6 | 0298019 | 0621027 Up |
| Mier3 | 0298149 | -031315 Down |
| Smg6 | 0298248 | 0284479 Up |

| Rap2b | 0298248 | -043819 Down |
| --- | --- | --- |
| Senp7 | 0298328 | 0995382 Up |
| U2af2 | 0298715 | -052154 Down |
| Bag2 | 0298907 | 1011551 Up |
| Pcyt1a | 0298907 | 2772966 Up |
| Nfic | 0299026 | -061136 Down |
| Nelfcd | 0299187 | 0493926 Up |
| Sc5d | 0299187 | 1027127 Up |
| Gap43 | 0299707 | 1009366 Up |
| Tmem106c | 030049 | 0677416 Up |
| Ankrd40 | 0300729 | 0449692 Up |
| Armc9 | 0300987 | 0546646 Up |
| 2900026A0 | 0300987 | 0831878 Up |
| Mtmr2 | 0300987 | 1781898 Up |
| Hdac5 | 0300987 | 0626798 Up |
| Zfp942 | 0300987 | 0709423 Up |
| Rps6ka3 | 0300987 | 098048 Up |
| Vcpip1 | 0300987 | -120367 Down |
| Kiz | 0300987 | -086185 Down |
| Aph1b | 0300987 | -103797 Down |
| Slc35a5 | 0300987 | -092451 Down |
| Psmd13 | 0301234 | -051583 Down |
| Nap1l1 | 0302303 | 039862 Up |
| Taok3 | 0302351 | 0354598 Up |
| Aspm | 0302759 | 0439552 Up |
| Gad1 | 0302759 | 2196754 Up |
| Unc13b | 0302759 | 0907759 Up |
| Ptdss1 | 0302759 | 0916845 Up |
| Polr3d | 0302759 | 1235785 Up |
| Zfp263 | 0302759 | 0965873 Up |
| Bak1 | 0302759 | 1330048 Up |
| Ccdc174 | 0302847 | -042432 Down |
| Arid2 | 0302847 | 0468104 Up |
| Dlc1 | 0303007 | 3084141 Up |
| Rnaseh2c | 0303048 | -09967 Down |
| Limd2 | 0303076 | 0370694 Up |
| Mrps9 | 0303489 | 0151951 Up |
| 1700088E0 | 0303489 | -08053 Down |
| Wrb | 0303489 | -08358 Down |
| Slbp | 0303676 | -07004 Down |
| Fam96b | 0304098 | 0847546 Up |
| Crtc3 | 0304204 | 0664772 Up |
| Pfdn6 | 0305721 | 0259618 Up |
| Ogfrl1 | 0306576 | -067354 Down |
| Gm6710 | 0306576 | -060599 Down |
| Cpxm1 | 0306645 | -092859 Down |
| Qpctl | 0306645 | -117681 Down |
| Neurl4 | 0306645 | -097379 Down |
| Phactr1 | 0306645 | -152636 Down |
| Map3k12 | 0306645 | -087677 Down |

| Rnf168 | 0306645 | -184881 Down |
| --- | --- | --- |
| Enox2 | 0306645 | -096839 Down |
| Cuedc2 | 0307111 | 0329007 Up |
| Frmd4a | 030718 | 1597134 Up |
| Vps41 | 030718 | 0935072 Up |
| Ube2d1 | 0307426 | 0981063 Up |
| Tmem50a | 0307572 | -041124 Down |
| Rars | 0307583 | 1008618 Up |
| Epc1 | 0307583 | 0540544 Up |
| Plod3 | 0307917 | -047991 Down |
| Slc18b1 | 0307917 | -067451 Down |
| Tmub2 | 0307917 | -023326 Down |
| Clta | 0307936 | -02004 Down |
| Zfp131 | 0307973 | -073626 Down |
| Rnf115 | 0307973 | 1709527 Up |
| Ndufv3 | 0308093 | -038854 Down |
| Fmn2 | 0308117 | 0805457 Up |
| Jmjd8 | 0308117 | 0929187 Up |
| Cox7c | 0308216 | -039827 Down |
| Pum2 | 0308336 | -03039 Down |
| Maged2 | 0308336 | 0587254 Up |
| Rnf130 | 0308345 | 1025671 Up |
| Fam135a | 0308462 | 140617 Up |
| Ankzf1 | 0308462 | 1067487 Up |
| Tmem185b | 0308462 | 1357731 Up |
| Ppox | 0308462 | 1205578 Up |
| Exo5 | 0308462 | 111511 Up |
| Ldb2 | 0308462 | 1631839 Up |
| Lin54 | 0308462 | 0996685 Up |
| Myo7a | 0308462 | 1141523 Up |
| Chac2 | 0308462 | 1191529 Up |
| Hspa2 | 0308462 | 228541 Up |
| Nudt13 | 0308462 | 122182 Up |
| 3110043O | 0308462 | 2150663 Up |
| Aldh9a1 | 0309283 | 0390617 Up |
| Eif4a3 | 0309446 | 1386234 Up |
| Lman2 | 0309618 | 0788533 Up |
| Fmo1 | 0309618 | 0777542 Up |
| Atp6v1g1 | 0309948 | 0356324 Up |
| Taf1 | 0310091 | 0507769 Up |
| 2310036O | 0310117 | 067908 Up |
| Cyfip1 | 0310896 | -076304 Down |
| Spcs1 | 0311065 | -037593 Down |
| Zranb2 | 0311065 | -048541 Down |
| Ift43 | 0311162 | 0362202 Up |
| Pofut1 | 031117 | 0472189 Up |
| Msantd3 | 031117 | 0186075 Up |
| Tchp | 031117 | -203053 Down |
| Cnpy4 | 031117 | -102962 Down |
| Zfp317 | 031117 | -068538 Down |

| Usp34 | 0311332 | -041766 Down |
| --- | --- | --- |
| Maf1 | 0311332 | -05931 Down |
| Srp68 | 0311846 | -122703 Down |
| Wasl | 0311936 | -0186 Down |
| Csdc2 | 0311936 | 0521151 Up |
| Rsrp1 | 0312784 | -102948 Down |
| Ubac1 | 0312792 | -107266 Down |
| Veph1 | 0312879 | 1061754 Up |
| Pomgnt1 | 0313346 | 0517805 Up |
| Socs2 | 0313346 | 0150745 Up |
| Trappc5 | 0313743 | -112306 Down |
| Btf3l4 | 0313861 | -035775 Down |
| Itpr2 | 031388 | 1719732 Up |
| Iqcb1 | 031388 | 1259807 Up |
| Plp2 | 031388 | 1387935 Up |
| Gins4 | 0314106 | -042917 Down |
| Lima1 | 0314106 | -049625 Down |
| Usp3 | 0314505 | -123769 Down |
| Syne2 | 0314548 | 1036377 Up |
| Epn1 | 0314928 | -044242 Down |
| Scaper | 0315294 | -069625 Down |
| Sap30bp | 0315294 | -028042 Down |
| Xrcc4 | 0315294 | -057174 Down |
| PISD | 0315294 | -048141 Down |
| Mydgf | 0315505 | -065817 Down |
| Cox6a1 | 0315821 | -043861 Down |
| Nip7 | 0316087 | -070692 Down |
| Ccnd3 | 0316087 | -133917 Down |
| Hbp1 | 0316087 | 0843715 Up |
| Leprot | 031609 | 0511423 Up |
| Caprin1 | 0316126 | 027866 Up |
| Zfp157 | 0316147 | 0866496 Up |
| Rbm15 | 0316389 | -095457 Down |
| Cdk5 | 0316389 | -087368 Down |
| Dusp3 | 0316389 | 1027353 Up |
| Llph | 031651 | -078514 Down |
| Wls | 0317513 | 0970323 Up |
| Eif2b1 | 0317513 | 1049761 Up |
| Ick | 0317513 | 0831968 Up |
| Ccdc47 | 0317519 | -028778 Down |
| Ndufs3 | 0317825 | -087417 Down |
| Prmt1 | 0317924 | -016006 Down |
| Rnmt | 0318156 | -085059 Down |
| Tfdp2 | 0318156 | 119731 Up |
| Asap1 | 0318156 | 0900963 Up |
| Spg21 | 0318169 | -05382 Down |
| Dock7 | 0318169 | -018291 Down |
| Zfp24 | 0318229 | 0785668 Up |
| Ergic1 | 0318245 | 0718384 Up |
| Elmo2 | 0318448 | -174644 Down |

| Extl2 | 0318448 | -087557 Down |
| --- | --- | --- |
| Alg6 | 0318448 | -165491 Down |
| Mogs | 0318448 | -109299 Down |
| Zfp446 | 0318448 | -11978 Down |
| Adi1 | 0318448 | -163665 Down |
| Snapin | 0318455 | 0651638 Up |
| Rexo2 | 0318455 | 1963439 Up |
| Eif3m | 0318667 | -101955 Down |
| Cirbp | 0318667 | 0546556 Up |
| Pcbp2 | 0318667 | -026353 Down |
| Tmed5 | 0318721 | -078577 Down |
| Hmox2 | 032028 | -038115 Down |
| Tmem184c | 0320517 | 0384718 Up |
| Pag1 | 0320517 | 0582011 Up |
| Pigyl | 0320517 | 0500243 Up |
| Col22a1 | 032061 | 0466195 Up |
| Adamts3 | 0321031 | 0761072 Up |
| Ppm1m | 0321031 | -01631 Down |
| Lmnb2 | 0321031 | 1110428 Up |
| Plscr3 | 0321031 | 2035818 Up |
| Klhl11 | 0321031 | 0955987 Up |
| Pdzd2 | 0321031 | 2340478 Up |
| C1qtnf6 | 0321031 | 1442305 Up |
| Nrm | 0321031 | 1278706 Up |
| Klf9 | 0321031 | 1222932 Up |
| Ablim1 | 0321031 | 1157454 Up |
| Magi2 | 0321577 | 077228 Up |
| Osbpl2 | 0321577 | -09409 Down |
| Tmem67 | 0321577 | -081444 Down |
| Fuom | 0321577 | -074224 Down |
| Armcx5 | 0321577 | -087209 Down |
| Ldha | 032212 | -056831 Down |
| Acyp1 | 032224 | 0530907 Up |
| Ift81 | 0322438 | 0384899 Up |
| Vegfa | 0322438 | -08805 Down |
| Kbtbd2 | 0322961 | 1485209 Up |
| Plekhg2 | 0322961 | 1454964 Up |
| Rmnd5a | 0323122 | -123297 Down |
| Ldah | 0323122 | -143913 Down |
| 2610507B1 | 0323584 | -07551 Down |
| Arvcf | 0323584 | 1472074 Up |
| Ssu72 | 032368 | 0779577 Up |
| Tmem65 | 0323956 | -054816 Down |
| Tagln3 | 0324173 | -048525 Down |
| BC004004 | 0324173 | -05605 Down |
| Nup62 | 0324866 | -022857 Down |
| Mical1 | 0324866 | 1022923 Up |
| Ccdc25 | 0324866 | 0396283 Up |
| Snx4 | 0324953 | -081686 Down |
| Ddx50 | 0324953 | 1095956 Up |

| Wwc1 | 0325104 | 041499 Up |
| --- | --- | --- |
| Dcaf6 | 0325267 | 0909212 Up |
| Rassf4 | 0325267 | 085685 Up |
| Brip1os | 0325267 | 1161483 Up |
| Cox11 | 0325267 | 0881795 Up |
| Osbpl11 | 0325267 | 0846945 Up |
| Timm17b | 0325911 | 0803694 Up |
| Otub1 | 0326223 | -032338 Down |
| Ppp1r9a | 0326223 | 0177013 Up |
| Zcchc24 | 0326357 | 1068997 Up |
| 1110059E2 | 0326357 | 1274207 Up |
| Sat1 | 0326607 | 0175335 Up |
| Snhg6 | 0326607 | 0691679 Up |
| Arl8a | 0326767 | -046078 Down |
| St7 | 0327095 | 1007725 Up |
| Rpf2 | 0327095 | 0880441 Up |
| Drosha | 0327095 | 0840916 Up |
| Nt5m | 0327374 | 0634719 Up |
| Dhrs1 | 0327646 | 0479629 Up |
| Akna | 0328081 | -037126 Down |
| Fdx1l | 0328081 | 0422317 Up |
| Klf7 | 0328094 | 027954 Up |
| Cdc42 | 0329105 | 0355314 Up |
| Smim8 | 0329284 | -079609 Down |
| Stau1 | 0329284 | 1384413 Up |
| Hsd17b12 | 0329437 | 0278172 Up |
| Uqcrc1 | 0329437 | -062057 Down |
| Mrpl42 | 0329437 | -045589 Down |
| Adk | 0330004 | 0187422 Up |
| Kdm5c | 0330004 | 0659828 Up |
| Calcoco1 | 0330004 | 151473 Up |
| Pcmtd1 | 0330289 | -028939 Down |
| Gigyf2 | 0330289 | 054747 Up |
| Ski | 0330501 | -059449 Down |
| Tbc1d12 | 0330509 | 0686527 Up |
| Ube2f | 0330509 | -062471 Down |
| Idh3a | 0330509 | -085019 Down |
| Tnks1bp1 | 0330544 | -101619 Down |
| Zbtb17 | 0330544 | -076177 Down |
| Mark4 | 0330544 | -092381 Down |
| Ano10 | 0330544 | -113859 Down |
| Zfp81 | 0330544 | -131815 Down |
| Snx32 | 0330544 | -108417 Down |
| D15Ertd62 | 0330544 | -112401 Down |
| Eif3a | 033131 | -029565 Down |
| Hnrnpu | 0331716 | 0381771 Up |
| Yipf1 | 0331951 | -076193 Down |
| Usp14 | 0331951 | -029049 Down |
| Mynn | 0331956 | -041833 Down |
| Zfp800 | 0331956 | -062291 Down |

| Cyb5r3 | 0331956 | -067869 Down |
| --- | --- | --- |
| Ttc9c | 0331956 | -05182 Down |
| Scarb2 | 0331997 | 1408453 Up |
| Hacd3 | 0331997 | 070776 Up |
| Actr1a | 0331997 | -049784 Down |
| Herpud2 | 0331997 | -083439 Down |
| 1810010H2 | 0332002 | -060984 Down |
| Dctn3 | 0332154 | -026616 Down |
| Myo1b | 0332209 | 0350932 Up |
| Ramp1 | 0332209 | 039191 Up |
| Pex14 | 0332209 | -071161 Down |
| Cd2bp2 | 0332209 | 1043686 Up |
| Zfp26 | 0332209 | 0251383 Up |
| Rnf144a | 0332209 | 0649867 Up |
| Zfp280c | 0332209 | 0285368 Up |
| 1810026J2 | 0332209 | 0318037 Up |
| Mms22l | 0332209 | -054083 Down |
| Nans | 0332209 | -080507 Down |
| Katnb1 | 0332209 | -075282 Down |
| Shmt2 | 0332209 | -15168 Down |
| Arf3 | 0332209 | -071195 Down |
| Rps6ka6 | 0332209 | -068114 Down |
| Clns1a | 0332406 | -076825 Down |
| Bub3 | 0332406 | 0524042 Up |
| Msmo1 | 0332486 | 0369065 Up |
| Prpf31 | 0333033 | -044312 Down |
| Dock1 | 0333096 | 0544418 Up |
| Sirt7 | 0333096 | -05435 Down |
| 2310033P0 | 0333214 | 0632083 Up |
| Gnl3 | 0333214 | -069028 Down |
| Tmem242 | 0333214 | -063105 Down |
| Thoc1 | 0333214 | -091604 Down |
| Aip | 0333214 | 0252005 Up |
| Megf9 | 0333253 | 0642533 Up |
| Arhgef25 | 0333253 | 1100846 Up |
| Znrf3 | 0333253 | 0916693 Up |
| Hnrnpd | 0333544 | 0222114 Up |
| Ccnd1 | 0333668 | -097116 Down |
| Ccpg1os | 0333741 | 0586167 Up |
| Clasp1 | 0333775 | -049787 Down |
| Ddx17 | 0333839 | 062736 Up |
| Gtf3c5 | 0333931 | 1372927 Up |
| Gpsm2 | 0333931 | 0832017 Up |
| Pkd2 | 0333931 | 1233768 Up |
| Dcun1d3 | 0333931 | 1413698 Up |
| Phlpp2 | 0333931 | 1455002 Up |
| Plpp1 | 0333931 | 1518024 Up |
| Rcl1 | 0333931 | 1332745 Up |
| Casp7 | 0333931 | 2864177 Up |
| Ssr1 | 0334039 | -064426 Down |

| Zcchc11 | 0334216 | 052004 Up |
| --- | --- | --- |
| Qdpr | 0334344 | 1256378 Up |
| Chd1 | 0334581 | 0647523 Up |
| H2afy | 0334582 | 0368681 Up |
| Higd1a | 0334612 | -044455 Down |
| Ppp6c | 0336673 | 0556659 Up |
| Fbxo28 | 0336863 | 0760753 Up |
| Kctd2 | 0336863 | 1138174 Up |
| Pygl | 0336863 | 0755542 Up |
| Brms1 | 0336863 | 0772219 Up |
| Amer1 | 0336863 | 1183657 Up |
| Gm10020 | 0336863 | 1094184 Up |
| Stk11 | 0336913 | 0613365 Up |
| 1110004E0 | 0336913 | 060261 Up |
| St8sia4 | 0336919 | 078013 Up |
| Zmat3 | 0336919 | 1102141 Up |
| Ppp1r8 | 0336919 | 105268 Up |
| Gcfc2 | 0336919 | 0601894 Up |
| Tmem135 | 0336919 | 0361566 Up |
| Arhgap42 | 0336919 | -145465 Down |
| Isca1 | 0336919 | 0602154 Up |
| Tmem161b | 0336919 | 0713145 Up |
| Cenph | 0336919 | 0448386 Up |
| Camk2n1 | 0337043 | 015615 Up |
| Dnajc9 | 0337043 | -064926 Down |
| Rsrc1 | 0337051 | 1145423 Up |
| Tm9sf2 | 0337051 | 0625816 Up |
| Zfp330 | 0337401 | -082918 Down |
| Dmtf1 | 0337401 | -015125 Down |
| Echs1 | 0337662 | 0880072 Up |
| Lage3 | 0337662 | 1103028 Up |
| Mrpl20 | 0337707 | 0393236 Up |
| Tspyl4 | 0338236 | -123846 Down |
| Btd | 0338477 | -017096 Down |
| Ccdc28b | 0338693 | 0179371 Up |
| Lbr | 0338915 | 0291037 Up |
| Cul1 | 0338932 | -028166 Down |
| Nxf1 | 0339978 | -030529 Down |
| Sri | 0340016 | -039358 Down |
| Gde1 | 0340016 | -05191 Down |
| Aco2 | 0340016 | -082128 Down |
| Pole3 | 0340073 | -026389 Down |
| Exosc4 | 0340073 | -137198 Down |
| Poldip3 | 0340073 | 0373298 Up |
| Rap2a | 0340549 | 0458019 Up |
| Fnbp1 | 0340549 | -070191 Down |
| Scd2 | 0341368 | 0322272 Up |
| Pgam1 | 0341368 | -02974 Down |
| Pdcd11 | 0341385 | -093514 Down |
| Chmp5 | 0341414 | -066968 Down |

| Cnpy2 | 0341414 | -104125 Down |
| --- | --- | --- |
| Serp1 | 0341568 | 05041 Up |
| Nsd1 | 0341568 | -039411 Down |
| Clasp2 | 0341771 | 0988095 Up |
| Galk2 | 0341817 | 018638 Up |
| Tecpr1 | 0341817 | -07519 Down |
| Pan3 | 0341817 | -060257 Down |
| Slc35a4 | 0341817 | -056621 Down |
| Trpc4ap | 0342007 | -043884 Down |
| Arhgap5 | 0342101 | -0554 Down |
| Aplp1 | 0342199 | -03971 Down |
| Fam234b | 0342416 | -039767 Down |
| Alas1 | 0342416 | -069054 Down |
| Tmem100 | 0342416 | -079419 Down |
| Ring1 | 0342416 | -04277 Down |
| Afap1l2 | 0342416 | 0866021 Up |
| Rexo1 | 0342532 | -125234 Down |
| Plec | 0342532 | -133184 Down |
| Znhit6 | 0342532 | 0595647 Up |
| Atp6v0b | 0342739 | -064499 Down |
| Psenen | 0342739 | -038102 Down |
| Bcl9l | 034277 | -020669 Down |
| Cdk17 | 034277 | 0567189 Up |
| Cryzl1 | 034277 | 062508 Up |
| Gmppa | 0342933 | -07145 Down |
| Mycbp | 0342933 | -240761 Down |
| Mdfic | 0342933 | -091963 Down |
| Rnf122 | 0342933 | -089378 Down |
| Slc44a2 | 0342933 | -071865 Down |
| Dcxr | 0342933 | -169681 Down |
| Dus1l | 0342933 | -074321 Down |
| Zdhhc14 | 0342933 | -049628 Down |
| Gpr137 | 0342933 | -064405 Down |
| Mast2 | 0343066 | 0416588 Up |
| Gtf2ird1 | 0343066 | -072149 Down |
| Cand2 | 0343066 | -073532 Down |
| 15-Sep | 034308 | -042001 Down |
| mt-Nd3 | 034325 | 019475 Up |
| Nsf | 0343265 | 0151413 Up |
| Selenbp1 | 0343754 | 1052193 Up |
| Rgs3 | 0343754 | 2365425 Up |
| Zswim8 | 0343754 | 1264864 Up |
| Scoc | 0344044 | -029733 Down |
| Gstp1 | 0345158 | 0352242 Up |
| Rgma | 0345158 | 086515 Up |
| Lsm3 | 0345265 | -043544 Down |
| Mia3 | 034531 | -051091 Down |
| Srsf4 | 034531 | 1500855 Up |
| Chd6 | 0345817 | 0465465 Up |
| Ddr1 | 0345946 | 0732377 Up |

| Prkacb | 0346127 | 042387 Up |
| --- | --- | --- |
| Ankrd46 | 0346139 | 0623479 Up |
| Bcl2l1 | 0346178 | -040436 Down |
| Chtf8 | 0346817 | 0830328 Up |
| Lias | 0347014 | -080449 Down |
| Dctpp1 | 0347014 | -042097 Down |
| Ptgs1 | 034716 | 1017638 Up |
| Prmt6 | 034716 | 1090296 Up |
| Snx30 | 034716 | 0539991 Up |
| Azin2 | 034716 | 0911957 Up |
| Ncapg | 034716 | 0888051 Up |
| Pgm2l1 | 034716 | 1492384 Up |
| Zfr2 | 034716 | 117962 Up |
| Bcas3 | 034716 | 0981045 Up |
| Glrx | 034716 | 0831682 Up |
| Cyp4f16 | 034716 | 0853846 Up |
| Syp | 034716 | 1175759 Up |
| Xrcc6bp1 | 034716 | 1178538 Up |
| Phip | 0347827 | -058074 Down |
| Ctnnd1 | 0348321 | 0911612 Up |
| Baz1b | 034868 | 0773814 Up |
| Pnisr | 0348733 | 0355496 Up |
| Igsf9 | 0348836 | 0587544 Up |
| Fbxo44 | 0348836 | 1532532 Up |
| Kmt2d | 0349241 | 0957065 Up |
| Mllt4 | 0349412 | -038189 Down |
| Fxr1 | 0349412 | 0440037 Up |
| Cisd2 | 0349697 | -078076 Down |
| Vps37b | 0349866 | -07607 Down |
| Zfp36l2 | 0350161 | -054264 Down |
| Ubn2 | 035017 | 0585298 Up |
| Tnrc6c | 035017 | 0256253 Up |
| Mrps12 | 035017 | 0480441 Up |
| Emp2 | 035017 | -07853 Down |
| Hmgb1 | 0350824 | 0289763 Up |
| E430025E2 | 0350935 | -073442 Down |
| Nlgn3 | 0351544 | -051353 Down |
| Xpo6 | 0351544 | -025261 Down |
| Actr3 | 0351636 | -026193 Down |
| Herc2 | 0351778 | -115127 Down |
| Wscd1 | 0351778 | 0247306 Up |
| Mrpl45 | 0351778 | -026661 Down |
| Arl6 | 0351778 | -068018 Down |
| Crls1 | 0351779 | -090785 Down |
| Eif2b3 | 0351863 | -026313 Down |
| Nova2 | 0351863 | -045037 Down |
| Igdcc4 | 0351863 | -068716 Down |
| Hdgf | 0353419 | 0336502 Up |
| Kdm4a | 0353632 | -145515 Down |
| Dpp8 | 0353839 | -06086 Down |

| Erbb4 | 0354146 | -115067 Down |
| --- | --- | --- |
| Aimp2 | 0354146 | -051524 Down |
| Stambp | 0354146 | -081979 Down |
| Wwox | 0354146 | -037045 Down |
| Ccng1 | 0354146 | -122618 Down |
| Lig3 | 0354146 | -027482 Down |
| G6pdx | 0354146 | -122184 Down |
| Erp44 | 0354225 | 0401367 Up |
| Alyref2 | 0354464 | 1317163 Up |
| Cc2d2a | 0354464 | 1210375 Up |
| Spsb2 | 0354464 | 1104321 Up |
| Atp6v0a1 | 0354464 | 1034541 Up |
| Pafah1b3 | 0355016 | 036692 Up |
| Ubqln1 | 0355099 | -056762 Down |
| Strn4 | 0355099 | 0515294 Up |
| Pdcd6ip | 0355465 | -0667 Down |
| Ttc28 | 0355567 | -025996 Down |
| AI854517 | 0355567 | -034104 Down |
| BC029722 | 0355615 | -067228 Down |
| Sdc4 | 0355615 | -084951 Down |
| Trp53rkb | 0355615 | -074191 Down |
| Gas6 | 0355615 | -050977 Down |
| Zfp612 | 0355615 | -042343 Down |
| Cep295 | 0355615 | -079496 Down |
| 0610010F0 | 0355615 | -051193 Down |
| Cast | 0355615 | -241677 Down |
| Arsb | 0355615 | 0425349 Up |
| Lmf2 | 0355615 | -064802 Down |
| Tecr | 0355642 | 0225007 Up |
| Rprd1a | 0355773 | 0526193 Up |
| Api5 | 0356134 | -03384 Down |
| Prpf39 | 0356603 | 2204857 Up |
| Ssbp3 | 0356694 | -045424 Down |
| Ube2j2 | 0356694 | -063041 Down |
| Ckap4 | 0356694 | -068153 Down |
| Ift27 | 0356694 | -03269 Down |
| Morn2 | 0356694 | 0758519 Up |
| Pam | 0356955 | 0990956 Up |
| Gnb4 | 0356955 | 2036335 Up |
| Zfr | 0357368 | 0762289 Up |
| Efhd2 | 0357398 | -030597 Down |
| Pde6d | 0357561 | -080682 Down |
| Psma6 | 0357795 | 0338611 Up |
| Btg1 | 0358031 | 0426685 Up |
| Zfp664 | 0358073 | 0598301 Up |
| Sdc2 | 0358073 | -145093 Down |
| Ier3 | 0358229 | 2432236 Up |
| Hsp90ab1 | 0358412 | -043518 Down |
| Ubb | 0358412 | 0168671 Up |
| Rps24 | 0358412 | -026247 Down |

| Rprm | 0359245 | 120345 Up |
| --- | --- | --- |
| Exosc9 | 0360653 | -029172 Down |
| Arnt | 0360653 | -13878 Down |
| Mettl16 | 0360653 | -097843 Down |
| Ddx52 | 0360706 | -056116 Down |
| Zdbf2 | 0360718 | 0829875 Up |
| Sv2a | 0360718 | 0685939 Up |
| Pdgfrl | 0360718 | 0675925 Up |
| Necab2 | 0360718 | 0893792 Up |
| Zbtb2 | 0360718 | 0722778 Up |
| Cbarp | 0360718 | 119946 Up |
| Rogdi | 0360718 | 155425 Up |
| Robo1 | 0360718 | 0792362 Up |
| F8a | 0360718 | 0746975 Up |
| Abca1 | 0360795 | 0478627 Up |
| Ptprs | 0360991 | 0932625 Up |
| Kcnb1 | 0361017 | 0397567 Up |
| Slc4a2 | 0361017 | 029953 Up |
| Zfp282 | 0361017 | 0520145 Up |
| Sugp2 | 0361017 | -047776 Down |
| 1700086L1 | 0361017 | 0270877 Up |
| Ufc1 | 0361099 | 0340859 Up |
| Ube2k | 0361099 | 0457735 Up |
| Rere | 0361302 | 1489904 Up |
| Tmem245 | 0361302 | 0763002 Up |
| 3-Sep | 0361757 | -056407 Down |
| Arhgap1 | 0362094 | -055119 Down |
| Mcur1 | 0362094 | -049413 Down |
| Ccnl2 | 0362243 | -035301 Down |
| Adrm1 | 0362441 | 0712383 Up |
| Stk16 | 03625 | -065294 Down |
| Man1a2 | 03625 | -068166 Down |
| Rnf138 | 03625 | -066566 Down |
| Enah | 0363221 | 0467891 Up |
| Pum1 | 0364161 | 0212653 Up |
| Tmem209 | 0364193 | -019957 Down |
| Fam173b | 0364193 | 0261117 Up |
| Zwint | 0364578 | -021221 Down |
| Setd3 | 0364578 | 0990963 Up |
| Crot | 0365194 | -049426 Down |
| Zfp771 | 0365194 | -059149 Down |
| Prdm16 | 0365194 | 056281 Up |
| Inhbb | 0365382 | 1254294 Up |
| Tmem214 | 0365382 | 1157879 Up |
| Wnt7a | 0365382 | 1649613 Up |
| BC005537 | 0365438 | -020274 Down |
| Traf3 | 036545 | 0840954 Up |
| Ppp1r3c | 036545 | 0262022 Up |
| Dcaf17 | 036545 | -029487 Down |
| Nup85 | 036545 | -101865 Down |

| Erdr1 | 0365477 | -034639 Down |
| --- | --- | --- |
| Rpe | 0365575 | 035757 Up |
| Ascl1 | 0365575 | 0731961 Up |
| Nlk | 0365575 | 067069 Up |
| Pcyt2 | 0365575 | 0587281 Up |
| Top2b | 0365651 | 0559019 Up |
| Ubxn1 | 0365651 | -038241 Down |
| Rps23 | 0365966 | -021508 Down |
| Ruvbl2 | 0365984 | -036342 Down |
| Pvrl3 | 0366019 | -076347 Down |
| Dnajc7 | 0366224 | -021302 Down |
| Polr2l | 0366442 | 0514934 Up |
| Herc1 | 0366442 | 0208195 Up |
| Rbbp6 | 036666 | -028507 Down |
| Blvrb | 0367045 | -021089 Down |
| Fh1 | 0367045 | 0363233 Up |
| Polr2b | 0367045 | 0544893 Up |
| Apopt1 | 0367045 | 040036 Up |
| Gart | 0367045 | 0336301 Up |
| Cd320 | 0367045 | 0687411 Up |
| Nfyc | 0367279 | -019887 Down |
| Bace1 | 0367279 | 0299912 Up |
| Ece2 | 0367279 | 0780593 Up |
| Atg9a | 0368589 | -06171 Down |
| Aven | 0368589 | -103556 Down |
| Nup93 | 0368589 | 0375671 Up |
| Aagab | 0368589 | 0383183 Up |
| Thoc5 | 0368589 | -081957 Down |
| Naga | 0368589 | -072218 Down |
| Pfkm | 0368589 | -017813 Down |
| Gpr156 | 0368589 | -051713 Down |
| Commd1 | 0368664 | -040213 Down |
| Son | 0368792 | -043755 Down |
| Cdh2 | 0369356 | 0241483 Up |
| 2310009B1 | 0369404 | -101109 Down |
| Dapk3 | 0370016 | -022248 Down |
| Nt5c3 | 0370016 | 0656384 Up |
| Zfp369 | 0370073 | 1723419 Up |
| Ilk | 0370183 | -070167 Down |
| Vps72 | 0370232 | -028989 Down |
| Tspan7 | 0370232 | -04366 Down |
| Mrps15 | 0370241 | 1766381 Up |
| Cul2 | 0370538 | -07731 Down |
| Ak6 | 0370776 | 0996229 Up |
| Mfn1 | 0370919 | 0772809 Up |
| Cry2 | 0370985 | 0408103 Up |
| Sh2b1 | 0370985 | 1726285 Up |
| Lpcat3 | 0371292 | 1089053 Up |
| Nhp2l1 | 0371428 | 0202431 Up |
| 9-Sep | 037156 | 0407717 Up |

| Hnrnpa0 | 0371902 | 0286536 Up |
| --- | --- | --- |
| Tpi1 | 0371949 | 0287489 Up |
| Dcaf12 | 0372509 | -040368 Down |
| Irak1 | 0372509 | -096487 Down |
| Mospd2 | 0372509 | -02931 Down |
| Fgfr1op2 | 0372751 | -118463 Down |
| Vps4a | 0373373 | -038715 Down |
| Ubxn8 | 0373462 | -055172 Down |
| 2700029M | 0373621 | -040791 Down |
| Rpl37a | 0373624 | -02569 Down |
| Brd1 | 0373819 | 0913717 Up |
| Tomm22 | 0374432 | 0267351 Up |
| Nck2 | 0374603 | 0573939 Up |
| Hace1 | 0374603 | 0435336 Up |
| Rhobtb2 | 0374603 | 0689787 Up |
| Cul9 | 0374603 | 0466012 Up |
| Nfya | 0374603 | 083554 Up |
| Cep120 | 0374603 | 0380953 Up |
| Il1rapl1 | 0374603 | 0803851 Up |
| Hibch | 037495 | -063641 Down |
| Wrap73 | 037495 | -084463 Down |
| Akap8l | 037495 | -087142 Down |
| Vars | 037495 | -099411 Down |
| Otud5 | 037495 | 1420973 Up |
| Peli1 | 0374987 | -048951 Down |
| Uqcrfs1 | 0375497 | 024027 Up |
| Ephb2 | 0375543 | 0382333 Up |
| Pank1 | 0375543 | 0608257 Up |
| Urm1 | 0375587 | -108382 Down |
| Tmem222 | 0375587 | -126989 Down |
| Csnk2a2 | 0376155 | 1218444 Up |
| Ints8 | 0376508 | 0970726 Up |
| Gphn | 0376508 | 0984523 Up |
| Mrpl30 | 0376756 | 0538004 Up |
| Mien1 | 0376756 | 0722765 Up |
| Uap1 | 0376977 | 0509108 Up |
| Cldn34c1 | 0376977 | 0788883 Up |
| Mtmr10 | 0376977 | -04712 Down |
| Eif4ebp1 | 0376977 | -018078 Down |
| Ing2 | 0376977 | -085162 Down |
| Sh3bp5l | 0376977 | -037906 Down |
| Gm26782 | 0376977 | -182687 Down |
| Stk25 | 0377153 | 06412 Up |
| Ncbp3 | 0377786 | 035378 Up |
| Pbx1 | 0378002 | 0556042 Up |
| Gps1 | 0378329 | 0329455 Up |
| Wdr82 | 0378419 | 0669976 Up |
| Fosb | 0378848 | -077997 Down |
| Fam98b | 0378848 | 0785735 Up |
| Usf2 | 0379035 | 0482036 Up |

| Dusp11 | 0379182 | 0415584 Up |
| --- | --- | --- |
| Atf6b | 0379474 | -051425 Down |
| Tada3 | 0379474 | 0506634 Up |
| Junb | 0379474 | 0993989 Up |
| Aktip | 0379474 | 0708516 Up |
| Stard3nl | 0379474 | 0861933 Up |
| Inpp5f | 0379616 | -11535 Down |
| Fam208a | 0380429 | 0466776 Up |
| Mrps22 | 0380828 | 0194414 Up |
| Gtf2f1 | 0380828 | -111528 Down |
| Nr1h2 | 0380828 | 0979615 Up |
| Zfp652 | 0380828 | 1139745 Up |
| Rpl29 | 0381266 | -01615 Down |
| Rpl7 | 0381362 | -016269 Down |
| Pon2 | 0381586 | -020946 Down |
| Ccdc39 | 0381854 | -034568 Down |
| Cd200 | 0381854 | -072721 Down |
| Gpank1 | 0381854 | -065818 Down |
| Cnp | 0382158 | -14716 Down |
| Selo | 0382158 | -20415 Down |
| Creg2 | 0382158 | 1694801 Up |
| Vps18 | 0382158 | 1313219 Up |
| Prelid1 | 0382379 | -095798 Down |
| Dnajc15 | 0382569 | -135936 Down |
| Ndrg2 | 0383054 | 0370895 Up |
| Rnpc3 | 0383108 | -068478 Down |
| Noc2l | 0383108 | -077557 Down |
| Ppp4c | 0383499 | 0597704 Up |
| Ctbp1 | 0384311 | -032813 Down |
| Rbm25 | 038496 | 0235897 Up |
| Denr | 0385077 | -057863 Down |
| Ctdspl | 0385309 | 0902732 Up |
| Dram2 | 0385678 | 0547987 Up |
| Casp6 | 0385678 | 0680833 Up |
| Arxes2 | 0385678 | 0495413 Up |
| Plbd2 | 0385901 | -052409 Down |
| Hexa | 0385901 | -127152 Down |
| Ckap2l | 0386169 | 0415631 Up |
| Nisch | 0386506 | -042261 Down |
| Pdpn | 0386913 | 0564602 Up |
| Parl | 0387568 | 0519866 Up |
| Rabgap1 | 0387798 | 0661975 Up |
| Colgalt1 | 038784 | 0899033 Up |
| Hist1h4d | 038784 | 0962838 Up |
| Morc3 | 038784 | 0795521 Up |
| Xrn2 | 0387992 | -048142 Down |
| Sft2d2 | 0388725 | -159574 Down |
| Lypla2 | 0388725 | -051564 Down |
| Zfp275 | 0388725 | -046324 Down |
| Gmeb2 | 0388814 | 0354442 Up |

| Tmem64 | 0388814 | 0219758 Up |
| --- | --- | --- |
| Mad1l1 | 0388814 | -022276 Down |
| Trf | 0388814 | 1321388 Up |
| Pros1 | 0388814 | 0448056 Up |
| Marcks | 0388906 | 0159216 Up |
| Pnp | 0389079 | -113156 Down |
| Ubxn4 | 0389134 | -024808 Down |
| Tmem5 | 038927 | 0462614 Up |
| Ralgapa1 | 0390062 | 1029969 Up |
| Kif1b | 0390304 | 0327285 Up |
| Cfap36 | 0390677 | -042299 Down |
| Dnajc10 | 0390807 | -077609 Down |
| Zfp512b | 0390807 | 0473496 Up |
| Tmem29 | 0390807 | -046736 Down |
| Rest | 0390829 | -029455 Down |
| Cdk5rap3 | 0390829 | 0807667 Up |
| Ndfip2 | 0391774 | 0170281 Up |
| Rab11a | 0391774 | -044537 Down |
| Psmd9 | 0392127 | -057456 Down |
| Spast | 0392206 | -072398 Down |
| Arf4 | 0393111 | 0457031 Up |
| Cox7b | 0393111 | -037902 Down |
| Lsm5 | 0393146 | 0540267 Up |
| Zc3h15 | 0393165 | -036246 Down |
| Eif1b | 0393837 | 0157369 Up |
| Olfm1 | 039389 | -041672 Down |
| Plk4 | 0394481 | -173732 Down |
| Msra | 0394481 | -10388 Down |
| Ddi2 | 0394481 | 3209574 Up |
| Tmem123 | 0394481 | 1832888 Up |
| Tspan14 | 0394481 | 1162067 Up |
| Timm21 | 0394789 | -059269 Down |
| Bad | 0395078 | 1532234 Up |
| Ccdc6 | 0395183 | -06943 Down |
| Prdx6 | 0395379 | 0448169 Up |
| C1qtnf4 | 0395407 | -022729 Down |
| Cstf1 | 0395407 | 0557178 Up |
| Gm4631 | 0395407 | -04558 Down |
| C130026L2 | 0395407 | -02806 Down |
| Tgfb1 | 0395407 | 0817204 Up |
| Xab2 | 0395407 | -023341 Down |
| Adprm | 0395407 | -056767 Down |
| Wrap53 | 0395407 | -070989 Down |
| Rptor | 0395407 | 032288 Up |
| Tbc1d22a | 0395407 | -124424 Down |
| Ptk7 | 0395407 | -034619 Down |
| Sh3pxd2a | 0395407 | -040686 Down |
| 4932443I1 | 0395407 | -052598 Down |
| Tomm40 | 0395448 | 0175556 Up |
| Ift20 | 0395448 | 0599593 Up |

| Atp5d | 0395881 | -029239 Down |
| --- | --- | --- |
| Ppp1cb | 0395927 | 0373373 Up |
| Ctc1 | 0395977 | 0428977 Up |
| Aldh6a1 | 0395977 | 0406739 Up |
| Tnks2 | 0396371 | 0216447 Up |
| Lhx2 | 0396371 | -027027 Down |
| Fkbp9 | 0397017 | -089371 Down |
| Elf2 | 0397017 | 0757686 Up |
| Rilpl2 | 0397017 | 0599426 Up |
| St7l | 0397045 | -115107 Down |
| Grik5 | 0397045 | -084287 Down |
| Sf3a2 | 0397045 | -095965 Down |
| Rab10os | 0397045 | -088337 Down |
| Arhgap39 | 0397045 | -100684 Down |
| Bcl7c | 0397708 | -068609 Down |
| Plce1 | 0398454 | 0818665 Up |
| Triap1 | 0398831 | -068286 Down |
| Vps53 | 0398831 | -029422 Down |
| Hspa9 | 0399043 | 0277302 Up |
| Coq10b | 0399349 | 0690633 Up |
| Coq5 | 0399349 | 0358514 Up |
| Kdm7a | 0399349 | 0539903 Up |
| Got2 | 0399349 | 0403894 Up |
| Btbd17 | 0399349 | 0311679 Up |
| Bhlhe40 | 0399378 | 1141867 Up |
| Nxn | 0399378 | 0732206 Up |
| Syngr2 | 0399378 | 0306903 Up |
| Phf21b | 0399378 | 0296896 Up |
| Nell2 | 0399378 | 1347567 Up |
| Cdip1 | 0399378 | 0497951 Up |
| Sike1 | 0399638 | -030907 Down |
| Uso1 | 0400177 | -041507 Down |
| Hey1 | 0400177 | 0493902 Up |
| Phxr4 | 0400177 | 0480932 Up |
| Nfxl1 | 0400693 | 0295937 Up |
| Brsk1 | 0400693 | 0277064 Up |
| Oxa1l | 0400873 | -030023 Down |
| Mrpl17 | 0400873 | 074766 Up |
| Usp5 | 0400989 | 0675039 Up |
| Stx8 | 0401032 | -022673 Down |
| Mrpl27 | 0401032 | -058274 Down |
| Pdlim7 | 0401032 | 0446546 Up |
| Tspan31 | 040112 | 0884273 Up |
| Dnajb1 | 0401852 | -095884 Down |
| Zmat2 | 0402695 | -021725 Down |
| Sdhc | 0403267 | -048735 Down |
| Papd7 | 0403349 | 0490981 Up |
| Ctnna1 | 0404096 | -065393 Down |
| Grhpr | 0404312 | 1049391 Up |
| Stk38 | 0404312 | -022598 Down |

| Hexb | 0404745 | -05697 Down |
| --- | --- | --- |
| Ccdc32 | 0404854 | 0442624 Up |
| Ing3 | 0404854 | -049579 Down |
| Sowahc | 0404854 | 0399651 Up |
| Copg1 | 0405208 | -16692 Down |
| Ctsa | 0405289 | -068062 Down |
| Klhl9 | 0405289 | -048968 Down |
| Psap | 040657 | -030598 Down |
| Srsf10 | 0406834 | -037991 Down |
| Zcchc10 | 0407042 | -077113 Down |
| Map2k3 | 0407042 | -179793 Down |
| Rps6kb2 | 0407042 | -081311 Down |
| Eif2ak4 | 0407042 | 1085282 Up |
| Pde4dip | 0407042 | 0508638 Up |
| Izumo4 | 0407042 | 0858851 Up |
| Appl2 | 0407042 | 0778982 Up |
| Cwc25 | 0407042 | 1282356 Up |
| Mcm4 | 0407042 | 131201 Up |
| Calr | 0407139 | 0359178 Up |
| Pogk | 0407767 | 0860758 Up |
| Pold3 | 0408381 | -059202 Down |
| Ssh2 | 0408381 | -110675 Down |
| Spata13 | 0408381 | -042007 Down |
| Six3 | 0408462 | -058597 Down |
| Atp6ap1 | 0408728 | -081009 Down |
| Jam3 | 0409223 | 1032415 Up |
| Mrpl57 | 0409223 | -034695 Down |
| Fubp3 | 0409223 | 0719901 Up |
| Hcfc1r1 | 0409223 | 0587507 Up |
| Pfkfb3 | 0409247 | -022949 Down |
| Fbrs | 0409247 | 0486605 Up |
| Zfp410 | 0409247 | -051039 Down |
| Cipc | 0409247 | -026407 Down |
| Ccdc85b | 0409247 | 0172868 Up |
| Zdhhc16 | 0409247 | -046675 Down |
| Eif2s2 | 0409349 | -041452 Down |
| Pabpn1 | 0409928 | 0329552 Up |
| Desi2 | 0409977 | -03314 Down |
| Avl9 | 0410277 | 049988 Up |
| Prkrip1 | 0410403 | 0676085 Up |
| Gtf2h4 | 0410403 | 0238933 Up |
| Ndufaf4 | 041112 | 079673 Up |
| Plekha5 | 041112 | 1035087 Up |
| L3mbtl3 | 041112 | 0819177 Up |
| Pcnt | 041112 | 0554468 Up |
| Zfp811 | 041112 | 1144017 Up |
| Amotl1 | 0411305 | 0746887 Up |
| Ints1 | 0411735 | -03646 Down |
| Pcyox1 | 0411735 | -066179 Down |
| Riok1 | 0411735 | -053475 Down |

| Nudt3 | 0412203 | 0440804 Up |
| --- | --- | --- |
| Fstl5 | 0412856 | 0414474 Up |
| Rufy3 | 0412856 | 0501077 Up |
| Uhrf1 | 041288 | -023688 Down |
| Cdc25b | 041288 | -117685 Down |
| Pard6b | 041288 | -037844 Down |
| Pno1 | 041288 | 0495314 Up |
| Memo1 | 041288 | -03683 Down |
| Tmem178 | 041288 | -033715 Down |
| Mus81 | 041288 | -020586 Down |
| Chd7 | 0413508 | 0534899 Up |
| Hmbs | 041397 | -063933 Down |
| Sfxn5 | 0414208 | -032224 Down |
| Grpel1 | 0414257 | 0152257 Up |
| Higd2a | 0414257 | 1282837 Up |
| Cdv3 | 0414257 | 054105 Up |
| Trmt112 | 0414257 | -126016 Down |
| Becn1 | 0416567 | -048045 Down |
| Ndufb3 | 0416802 | 0202473 Up |
| Nrarp | 0417209 | 0327121 Up |
| Trip11 | 0417209 | 0667422 Up |
| Zfand3 | 0417278 | 053968 Up |
| Arpc5l | 0417847 | 0227574 Up |
| Slu7 | 0418118 | -086726 Down |
| Itgb8 | 0418118 | 0568377 Up |
| Fzd5 | 0418204 | 0378742 Up |
| Pdlim2 | 0418204 | -189232 Down |
| Edf1 | 041833 | -015322 Down |
| Mad2l2 | 041877 | -03467 Down |
| Gfpt1 | 041877 | -026937 Down |
| Tmem70 | 041877 | -049526 Down |
| Tle3 | 0418807 | 102888 Up |
| Fam120a | 0419039 | -030918 Down |
| Fasn | 0419039 | 0637012 Up |
| Gabbr1 | 0419039 | 0609396 Up |
| Bmpr1b | 0419838 | -100208 Down |
| Nrbp2 | 0419838 | -091213 Down |
| Inpp4a | 0419838 | 1471579 Up |
| 2900060B1 | 0419838 | 0905431 Up |
| A430005L1 | 0419838 | 1381142 Up |
| Cep68 | 0419838 | 2102019 Up |
| Taf2 | 0419838 | 0885126 Up |
| Nme4 | 0419838 | 1507343 Up |
| Zfp536 | 0419908 | -103563 Down |
| Slc38a10 | 0419908 | -080096 Down |
| Scrib | 0419908 | -113604 Down |
| Rtn4 | 0419952 | -042422 Down |
| Snrpg | 042034 | 0440474 Up |
| Ext2 | 0420528 | -03273 Down |
| Mapkap1 | 0420638 | 0971771 Up |

| Rpl36a | 0421514 | -029399 Down |
| --- | --- | --- |
| Raph1 | 0421637 | 0363459 Up |
| Rnasel | 0421637 | 0285003 Up |
| Tmem246 | 0421778 | -030381 Down |
| Dnmt1 | 0421778 | 1162723 Up |
| Med1 | 0421778 | 2123498 Up |
| Ranbp9 | 0421778 | 0988657 Up |
| Pfdn2 | 0422088 | 076305 Up |
| Gpatch8 | 0422157 | 0319029 Up |
| Actr1b | 0422435 | 0826271 Up |
| Zfp945 | 0422435 | 111436 Up |
| Taz | 0422435 | 0867537 Up |
| Slc25a23 | 0422444 | 119508 Up |
| Pi4k2a | 0422444 | -035977 Down |
| Lyrm5 | 0422444 | -062359 Down |
| Add1 | 0422795 | -015988 Down |
| Ddah1 | 0422795 | 0290482 Up |
| Mrpl10 | 0423064 | 0862526 Up |
| Zfp74 | 0423372 | -01718 Down |
| Tmem60 | 0423706 | 0732972 Up |
| Rbm28 | 0423706 | 1258153 Up |
| Rnf216 | 0423765 | -035065 Down |
| Epb41l4ao | 0423765 | -049927 Down |
| Utp14a | 0423765 | -060194 Down |
| Mdh1 | 0424259 | 0234439 Up |
| Klhl7 | 0425061 | -027684 Down |
| Ddx3x | 0425068 | 0489056 Up |
| Efcab2 | 0425284 | -024173 Down |
| Focad | 0425284 | -039297 Down |
| Atp13a1 | 0425284 | -039481 Down |
| Pes1 | 0425284 | -045356 Down |
| Hist1h1c | 0425494 | -083276 Down |
| Taf6 | 0425709 | -08955 Down |
| Synj2bp | 0425709 | 0951342 Up |
| Pdcd5 | 0425979 | 0297923 Up |
| Prrc2b | 0426206 | -029625 Down |
| Fkbp1a | 0426319 | 0464607 Up |
| Rangap1 | 0426552 | -179482 Down |
| Srebf1 | 0426552 | 075702 Up |
| Cfl2 | 0426689 | -044729 Down |
| Upf3b | 0427125 | -02026 Down |
| Nrxn2 | 0427125 | 0534111 Up |
| Egr1 | 0427659 | -04634 Down |
| Atxn2 | 0427803 | 0543551 Up |
| Sub1 | 0427843 | 0320797 Up |
| Kcnn2 | 0427859 | 0423336 Up |
| Plagl1 | 0428172 | -082708 Down |
| Stx7 | 0428218 | 0379468 Up |
| Trove2 | 0428218 | 0571319 Up |
| Abat | 0428218 | -058794 Down |

| H2afv | 0428687 | 0311619 Up |
| --- | --- | --- |
| Nufip2 | 0428956 | -106838 Down |
| Smarca1 | 0428956 | 0452019 Up |
| Rnaset2b | 0428956 | -112159 Down |
| Kifap3 | 0429408 | -032151 Down |
| Ywhae | 0429852 | -026767 Down |
| Pja2 | 0430711 | 1014437 Up |
| Dcx | 0430928 | -046342 Down |
| Pgm2 | 0431596 | -067823 Down |
| Anapc7 | 0431623 | -056345 Down |
| Nat14 | 0431623 | -028834 Down |
| Rgl2 | 0431623 | -071819 Down |
| Kdm6a | 0431623 | -084481 Down |
| Amz2 | 0431623 | 2253387 Up |
| Mtdh | 0431713 | 034867 Up |
| Sra1 | 0432391 | 077966 Up |
| Sall3 | 0432391 | 0449607 Up |
| Entpd2 | 0432405 | 1012147 Up |
| Pex7 | 0432405 | 0631898 Up |
| Zc3h7a | 0432405 | 0693743 Up |
| Zhx3 | 0432867 | -088632 Down |
| Ctso | 0432867 | -065359 Down |
| St8sia1 | 0432867 | -065697 Down |
| Smg9 | 0432867 | -105222 Down |
| Sp2 | 0432867 | -117231 Down |
| Gramd3 | 0432867 | -076997 Down |
| Rnf41 | 0433049 | 0626556 Up |
| Alkbh7 | 0433049 | 0306197 Up |
| Adrbk1 | 0433049 | 0743877 Up |
| Asrgl1 | 0433289 | 0618258 Up |
| Dpp3 | 0433314 | -049682 Down |
| Tk1 | 0433376 | -13744 Down |
| Miat | 0433566 | -073509 Down |
| Pde7a | 0433566 | 1018033 Up |
| Mark2 | 0433566 | 0922441 Up |
| Kif5c | 0433693 | 0878005 Up |
| 1110065P2 | 0433693 | 0469561 Up |
| Pcsk1n | 0433693 | -046436 Down |
| Txn2 | 0433774 | 0362281 Up |
| Gspt1 | 0433774 | -031932 Down |
| Snrpa | 0434019 | -06676 Down |
| Sar1b | 0434978 | -031981 Down |
| Adss | 0435209 | 070399 Up |
| Pigp | 0435243 | -122017 Down |
| Ehmt2 | 0435303 | -111879 Down |
| Cntrl | 0436183 | -066219 Down |
| Dcaf8 | 0436245 | 0574482 Up |
| Rbm17 | 0436245 | 0449128 Up |
| 1500011B0 | 0436674 | 0934173 Up |
| Cers2 | 0437248 | -046774 Down |

| Fcf1 | 0437248 | 0765864 Up |
| --- | --- | --- |
| Armcx3 | 0437248 | 0695413 Up |
| G3bp2 | 0437641 | 0569928 Up |
| Vegfb | 0437659 | -049626 Down |
| Rasal2 | 0437659 | 0427 Up |
| Hmgn1 | 0437708 | -024025 Down |
| Isy1 | 0437777 | 0513827 Up |
| Peg12 | 0437777 | 0952285 Up |
| Eri1 | 0437777 | 1257843 Up |
| Tmem38b | 0437777 | 133597 Up |
| Snhg20 | 0437777 | 1352134 Up |
| Tmem164 | 0437777 | 1187846 Up |
| Gosr2 | 0437903 | -022139 Down |
| Telo2 | 0437903 | -024018 Down |
| Mrps17 | 0438007 | -024945 Down |
| Tarbp2 | 043881 | -069762 Down |
| Ccdc80 | 043881 | 0361594 Up |
| Idh3g | 0439881 | -072461 Down |
| Pgrmc1 | 044029 | -022212 Down |
| Fabp7 | 0441301 | 0545063 Up |
| Trim44 | 0441654 | 0324956 Up |
| Etfa | 0443086 | 0571497 Up |
| Ube2q2 | 0443185 | 0538388 Up |
| Ncoa1 | 0443185 | 0566191 Up |
| Slc38a1 | 0443185 | 0778266 Up |
| Mapk8 | 0443185 | -052725 Down |
| Heg1 | 0443526 | -056562 Down |
| Ino80 | 0443526 | 1334878 Up |
| Gas8 | 0443526 | 1337162 Up |
| Dstn | 0443697 | -030554 Down |
| Snd1 | 0443754 | 1651731 Up |
| Synrg | 0443754 | 0426017 Up |
| Pim1 | 0443754 | -049728 Down |
| Msantd4 | 0444345 | -040355 Down |
| Ngly1 | 0444345 | -073334 Down |
| Naaa | 0444638 | 0784507 Up |
| Cap1 | 0444865 | -11351 Down |
| Cep350 | 0444865 | 1093828 Up |
| Tex261 | 0445242 | 0614481 Up |
| Nol11 | 0445242 | -0566 Down |
| Sema6d | 0445476 | 126574 Up |
| Flna | 0445476 | 0752986 Up |
| Cd63 | 0445622 | -026045 Down |
| Pard3b | 0446128 | -086489 Down |
| Rnf40 | 0446128 | -149165 Down |
| Rab3ip | 0446128 | -083418 Down |
| Grip1 | 0446128 | -11975 Down |
| Trim47 | 0446128 | -231794 Down |
| B3gnt5 | 0446128 | -184377 Down |
| Map3k2 | 0446128 | -109263 Down |

| Pcyt1b | 0446128 | -046931 Down |
| --- | --- | --- |
| Gm20604 | 0446128 | -066896 Down |
| Porcn | 0446128 | 1283114 Up |
| Gars | 0446297 | -039758 Down |
| Ppp2r2b | 0446327 | 0655662 Up |
| Mgat5 | 0446803 | 0248367 Up |
| Nkap | 0446803 | -156893 Down |
| Dcakd | 0446923 | 0432107 Up |
| Oard1 | 0446923 | 0438735 Up |
| Kcmf1 | 0446934 | 0920821 Up |
| Bckdha | 0446934 | 0971224 Up |
| Capza1 | 0446959 | -040729 Down |
| Vmp1 | 0446959 | -043014 Down |
| 1110059G | 0447265 | 073115 Up |
| Mfap1a | 0447554 | 06972 Up |
| Chkb | 0447554 | 0446299 Up |
| Apool | 0447554 | 0384152 Up |
| Rabggtb | 0449754 | -018591 Down |
| Med30 | 0449754 | -04751 Down |
| Zfp609 | 0449754 | -10481 Down |
| Cenpf | 0449919 | -17239 Down |
| Fars2 | 0449919 | 0379266 Up |
| Stk4 | 045026 | -050111 Down |
| Rint1 | 0450498 | 044929 Up |
| Tln1 | 0450673 | -0259 Down |
| Kdm5b | 0450673 | -041513 Down |
| Dact3 | 0450735 | -042523 Down |
| Sepn1 | 0450735 | 048213 Up |
| Dnaja2 | 0451492 | 0590293 Up |
| Blmh | 0451492 | 0456142 Up |
| Hint2 | 045204 | -110392 Down |
| Bmyc | 0452115 | -049104 Down |
| Sfr1 | 0452344 | -036057 Down |
| Kif3b | 0452461 | 1740773 Up |
| Chst12 | 0452461 | 2295951 Up |
| Fbxo25 | 0452461 | 1218403 Up |
| 2810001G | 0452461 | 1529336 Up |
| Ssb | 0453919 | -026392 Down |
| Adgrb3 | 0454118 | 0312754 Up |
| Man2a2 | 0454118 | -077002 Down |
| Rab6b | 0454818 | 031208 Up |
| Zmym4 | 045526 | -017333 Down |
| Uba5 | 0455309 | 1217616 Up |
| Maml3 | 0455533 | -046016 Down |
| Eif2b5 | 0455615 | -090622 Down |
| Fam160b1 | 0455615 | -033466 Down |
| Cdca7 | 0455758 | 1316373 Up |
| Cir1 | 0456003 | 0412937 Up |
| H19 | 0456243 | 0547893 Up |
| Mcm6 | 0456243 | 0604901 Up |

| Ctbp2 | 0456243 | 0866571 Up |
| --- | --- | --- |
| Pycrl | 045633 | -016514 Down |
| Map3k7 | 045633 | 0463323 Up |
| Timm44 | 045633 | 1862933 Up |
| Edem2 | 0456401 | 0534004 Up |
| Cep44 | 0456401 | 0294609 Up |
| Cul5 | 0456447 | 0530654 Up |
| Numa1 | 0456792 | -090853 Down |
| Vamp2 | 0456792 | -035722 Down |
| Odf2 | 0456792 | 0565567 Up |
| Gm11627 | 0456989 | 0489783 Up |
| Snx2 | 0457329 | -041899 Down |
| Mrpl1 | 0457569 | -062453 Down |
| Cpsf3 | 0457569 | 0723638 Up |
| Ube2e3 | 0457735 | 0236995 Up |
| Nsa2 | 0457735 | 0629215 Up |
| Commd3 | 0457963 | 0328586 Up |
| Raly | 0457963 | 0390001 Up |
| Med28 | 0458024 | 1239455 Up |
| Chrac1 | 0458315 | -034309 Down |
| Dbi | 0459572 | 0241974 Up |
| Stk40 | 0459617 | -052478 Down |
| Cfap69 | 0459617 | -053175 Down |
| Usp46 | 0459617 | -053061 Down |
| Tbc1d17 | 0459617 | -057349 Down |
| Trim12a | 0459617 | -22832 Down |
| Coro1a | 0459617 | -113217 Down |
| Cmtm6 | 0459617 | -084014 Down |
| Rims2 | 0459617 | -064263 Down |
| Mb21d2 | 0459617 | -066666 Down |
| Nsmf | 0459617 | 0902116 Up |
| Gpsm1 | 0459617 | 2355037 Up |
| Rab36 | 0459617 | 0878159 Up |
| Phlda1 | 0459617 | 2683005 Up |
| Efnb3 | 0459617 | 0996808 Up |
| Dnal1 | 0459617 | 0872877 Up |
| Pbk | 0459617 | 1160493 Up |
| Armcx2 | 0459617 | 0630069 Up |
| Ubr1 | 0460094 | 0539619 Up |
| Card19 | 0460094 | 1163564 Up |
| RP23-207N | 0460094 | 0452993 Up |
| Tmem98 | 0460094 | -09461 Down |
| Ftsj1 | 0460094 | -197886 Down |
| Nelfb | 0460364 | 0299019 Up |
| Pla2g16 | 0460364 | -026638 Down |
| Tgs1 | 0460364 | 0663247 Up |
| Ythdc1 | 0460502 | -04091 Down |
| Ak3 | 0460515 | -054142 Down |
| 0610010K1 | 0460515 | 0289645 Up |
| Bckdk | 0461187 | 0649064 Up |

| Serf1 | 046142 | 0276398 Up |
| --- | --- | --- |
| Fbrsl1 | 046172 | -052606 Down |
| Srsf6 | 0462204 | 0432368 Up |
| Ahctf1 | 0462348 | 019332 Up |
| Pcgf3 | 0462348 | -114685 Down |
| Tbc1d16 | 0462348 | 0793993 Up |
| Asnsd1 | 0462577 | -060703 Down |
| Plxnc1 | 0462577 | 047618 Up |
| Rpp30 | 0462577 | -069823 Down |
| Thoc2 | 0462902 | -057593 Down |
| Ubqln2 | 046301 | 0597927 Up |
| Sf3b1 | 0463021 | 0262452 Up |
| Lhfp | 0463777 | 0632238 Up |
| Cox6b1 | 046415 | -018871 Down |
| Fth1 | 046424 | -06263 Down |
| Gm17750 | 0464272 | 0286705 Up |
| Tyms | 0464272 | 0792339 Up |
| Frg1 | 0464513 | 0623426 Up |
| Slc9a3r1 | 0465044 | 0465495 Up |
| Rap1gds1 | 0465201 | 0834043 Up |
| Htra2 | 0465201 | 0682873 Up |
| Get4 | 0465201 | -104645 Down |
| Ilf2 | 0465499 | 0660525 Up |
| Cdk16 | 0465502 | -076775 Down |
| Tbrg1 | 0466084 | -073202 Down |
| Hnrnpll | 0466314 | -0325 Down |
| Rala | 04666 | -029879 Down |
| Clint1 | 0466832 | -031245 Down |
| Snn | 0466832 | -077162 Down |
| Ndufaf3 | 0466879 | -025707 Down |
| Ech1 | 0467251 | 0392952 Up |
| Arid1a | 0467403 | 0435594 Up |
| Uqcc1 | 0467419 | 1818447 Up |
| Zfp383 | 0467419 | 132522 Up |
| Snrpn | 0467732 | 0492269 Up |
| Mapre2 | 0467859 | -054381 Down |
| Flot1 | 0467859 | 0219335 Up |
| Ap1s2 | 0467859 | 0648823 Up |
| Zfp212 | 0467886 | -060872 Down |
| Snapc2 | 0467886 | -029073 Down |
| Banp | 0467886 | -036772 Down |
| Slc35b2 | 0467886 | -072761 Down |
| Ppp2r5c | 0467886 | 0685869 Up |
| Cyp20a1 | 0467959 | -055735 Down |
| Setdb1 | 0467959 | -064047 Down |
| Mybbp1a | 0467959 | -07415 Down |
| Dach1 | 0467959 | -07686 Down |
| Wdr12 | 0467959 | 0569181 Up |
| Prcc | 0467959 | 0369515 Up |
| Fam81a | 0467959 | 0580911 Up |

| Atad2 | 0467959 | 0996188 Up |
| --- | --- | --- |
| Ipo9 | 046848 | -057797 Down |
| Prkar2a | 046848 | -024847 Down |
| Ndufs2 | 0469973 | -029645 Down |
| Timm10 | 0470112 | 053838 Up |
| Exosc10 | 0470646 | -048763 Down |
| Chd4 | 0471028 | 0729278 Up |
| Wbp1 | 0471514 | -016211 Down |
| Ahsa2 | 0471767 | 0246009 Up |
| Arhgap21 | 0472481 | 0368394 Up |
| Ndrg3 | 047261 | 0378664 Up |
| Lig1 | 047261 | 0271449 Up |
| Abl1 | 0472829 | 2015738 Up |
| Klhl32 | 0472829 | 0882242 Up |
| Lrrc41 | 0472829 | 0386037 Up |
| Trafd1 | 0472829 | 1097664 Up |
| Rps11 | 0473068 | -021488 Down |
| Mpped2 | 0473257 | 0462729 Up |
| Madd | 0473331 | -024564 Down |
| Samhd1 | 0473331 | -07 Down |
| Atpaf1 | 0473331 | -151743 Down |
| Hk2 | 0473331 | -050435 Down |
| Cd3eap | 0473331 | -053363 Down |
| Hirip3 | 0473331 | -051029 Down |
| Dmxl2 | 0473331 | 0359443 Up |
| Spred2 | 0473331 | -037814 Down |
| Mtmr6 | 0473331 | -065456 Down |
| Il10rb | 0473331 | -063454 Down |
| Dirc2 | 0473331 | -055392 Down |
| Zfyve19 | 0473331 | 1108918 Up |
| Tollip | 0473331 | 0621134 Up |
| Dimt1 | 0473331 | 0916415 Up |
| Med20 | 0473331 | 0951606 Up |
| Aldh18a1 | 0473331 | 0710037 Up |
| Smchd1 | 0473828 | -028999 Down |
| Rev3l | 0474726 | 0954715 Up |
| P4ha1 | 0474726 | 0523667 Up |
| Fyttd1 | 0474726 | 0518967 Up |
| Ipo5 | 0474951 | -071497 Down |
| Eapp | 0474951 | 0410593 Up |
| Dda1 | 0475822 | 0337643 Up |
| Htatsf1 | 0475822 | 0440317 Up |
| Dip2b | 0475822 | -059175 Down |
| Rpgrip1 | 0475833 | 0674302 Up |
| Cdc37 | 0475833 | 0234355 Up |
| Tram1 | 0476432 | 1387147 Up |
| Lancl1 | 0476432 | 0583366 Up |
| Pelp1 | 0476432 | 036658 Up |
| Mbnl2 | 0476432 | -053484 Down |
| Cdc40 | 0476454 | -051443 Down |

| Mboat2 | 0476506 | 0538076 Up |
| --- | --- | --- |
| Arid4b | 0476536 | -036444 Down |
| Cdc123 | 0476791 | -03143 Down |
| Klf3 | 0476791 | -049666 Down |
| Fkbp3 | 0477404 | -031837 Down |
| Vps35 | 0477404 | 0288449 Up |
| Eno1 | 0478173 | -026693 Down |
| Vps50 | 047838 | -049815 Down |
| Ric3 | 047838 | -039241 Down |
| Npm3 | 047838 | -068469 Down |
| Nudt16l1 | 0478522 | 0233224 Up |
| Gnaq | 0478586 | -021687 Down |
| Mtch2 | 047882 | 0198277 Up |
| Ngfrap1 | 0478848 | 0513743 Up |
| Stx12 | 0479072 | -05044 Down |
| Sclt1 | 0479532 | 0863961 Up |
| Ppfia1 | 0479532 | -04245 Down |
| Ict1 | 0479535 | 0343574 Up |
| Sox9 | 0479535 | 0409526 Up |
| Pex19 | 0479751 | -051917 Down |
| Lmna | 0479751 | -258046 Down |
| Asphd2 | 0479751 | 0985204 Up |
| Mycn | 0479751 | 0778496 Up |
| Ubr2 | 0479751 | 038379 Up |
| Tcof1 | 0479751 | 0606705 Up |
| Rpl23a-ps3 | 0479751 | 0984824 Up |
| Grb10 | 0479818 | 0928514 Up |
| Ola1 | 0479818 | -132622 Down |
| Ccnd2 | 0479945 | 075017 Up |
| Ppil3 | 0480338 | -090983 Down |
| Znhit3 | 0480338 | -018953 Down |
| Trio | 0480338 | 0628705 Up |
| Eid2 | 0480416 | -016737 Down |
| Uimc1 | 0480445 | 18101 Up |
| Pcca | 0480445 | 183321 Up |
| Map2k5 | 0480445 | -178177 Down |
| Snrnp200 | 0481422 | 0655933 Up |
| Znrd1 | 0481422 | -022203 Down |
| Nsg2 | 0481898 | 106576 Up |
| Ppia | 0481959 | -036891 Down |
| Iah1 | 0481998 | -084523 Down |
| Aurkaip1 | 0482334 | 043958 Up |
| Crip2 | 0482516 | 0302562 Up |
| Grb14 | 0482647 | 1040873 Up |
| Fnbp4 | 0482647 | 1426029 Up |
| Sass6 | 0482647 | 1303201 Up |
| Wdr19 | 0482647 | 1004124 Up |
| Chl1 | 0482647 | 1512451 Up |
| Ap3b2 | 0482647 | 1141774 Up |
| Pdp2 | 0482647 | 0960824 Up |

| Arrb2 | 0482647 | 1754389 Up |
| --- | --- | --- |
| Smg8 | 0482647 | 0993686 Up |
| Zfp472 | 0482647 | 1875711 Up |
| Dtymk | 0483329 | 0230014 Up |
| Trim9 | 0483494 | -030023 Down |
| Uchl3 | 0483761 | -03337 Down |
| Fbxo18 | 0483903 | -024849 Down |
| Twf2 | 0484188 | 0272064 Up |
| Psme2 | 0484712 | -159672 Down |
| Pmpcb | 0484712 | 0741227 Up |
| Nfu1 | 0485308 | 1034883 Up |
| Zfpl1 | 0485308 | 1203929 Up |
| Plpp3 | 0485382 | 0235725 Up |
| Dusp12 | 0485756 | 2005237 Up |
| Celsr2 | 0485756 | 0323155 Up |
| Agl | 0485756 | 0592426 Up |
| Rnf157 | 0485756 | 0654186 Up |
| Map3k10 | 0485756 | -09688 Down |
| Ube2q1 | 0486265 | 025044 Up |
| Pgp | 0486865 | 143373 Up |
| Gtf2i | 0487137 | 0633522 Up |
| Psma5 | 0487199 | -023169 Down |
| Taf3 | 0487268 | -041092 Down |
| Cwc22 | 0487268 | -035752 Down |
| Aldh4a1 | 0487268 | -028354 Down |
| Pusl1 | 0487268 | -027031 Down |
| 1700096K1 | 0487268 | -069442 Down |
| Rnf181 | 0487268 | -030395 Down |
| Tm6sf1 | 0487268 | -062239 Down |
| Nek1 | 0487268 | -036021 Down |
| Gm26532 | 0487268 | -084923 Down |
| Rcor2 | 0487268 | -069144 Down |
| Ctdsp1 | 0487268 | 068081 Up |
| Mrgbp | 0487268 | 0604116 Up |
| Zfp655 | 0487268 | 0516489 Up |
| Pdcd2l | 0487268 | 0772875 Up |
| AI987944 | 0487268 | 064477 Up |
| Cdpf1 | 0487268 | 0658563 Up |
| Kif11 | 0487268 | 0406125 Up |
| Mbtps2 | 0487268 | 05451 Up |
| D3Ertd254 | 0487268 | 0671498 Up |
| Kin | 0487811 | 0434654 Up |
| Cdh4 | 0487811 | 0634593 Up |
| 4931406C0 | 0487811 | 0677671 Up |
| Psmb7 | 0488102 | -021436 Down |
| Shisa4 | 0488475 | -042821 Down |
| Gabarapl2 | 0488953 | 0740746 Up |
| C330007P0 | 0489316 | -045976 Down |
| Nme5 | 0489316 | 087235 Up |
| Smarca5 | 0489746 | 0315816 Up |

| Srsf2 | 0489746 | 0285715 Up |
| --- | --- | --- |
| Fbxw9 | 0490034 | -018749 Down |
| Ap1g1 | 0490034 | 0613651 Up |
| Atp2b4 | 049048 | 1559989 Up |
| Morf4l1 | 0490492 | -025981 Down |
| Plin3 | 0490726 | -053157 Down |
| Naf1 | 0490726 | 1361644 Up |
| Peli2 | 0490726 | 0658723 Up |
| Rsl1d1 | 0490739 | -048574 Down |
| Rpl6 | 049093 | -021979 Down |
| Ier5 | 0491703 | -021538 Down |
| Neto2 | 0491703 | -047992 Down |
| Nufip1 | 0491703 | -134943 Down |
| Ypel1 | 0491703 | -053722 Down |
| Phf20 | 0491703 | 0517779 Up |
| Aup1 | 0491703 | 0475361 Up |
| Fbln2 | 0491703 | -034818 Down |
| Zfp318 | 0491703 | 120687 Up |
| Tet2 | 0492969 | -018408 Down |
| Dlst | 0492969 | -055463 Down |
| Gtf2f2 | 0492969 | -049538 Down |
| Igf2r | 0492969 | -068787 Down |
| Ostf1 | 0492969 | -08984 Down |
| Gt(ROSA)2 | 0492969 | 0723472 Up |
| Dleu7 | 0492969 | 0921317 Up |
| Echdc1 | 0493019 | -029262 Down |
| Dck | 0493019 | -085789 Down |
| Akirin1 | 049458 | -045175 Down |
| Pdia4 | 0494715 | 0643318 Up |
| Ddt | 0494715 | 0494529 Up |
| Kif1bp | 0495662 | 0382802 Up |
| Tsc22d2 | 0495662 | 0509021 Up |
| Elavl4 | 0495917 | -030368 Down |
| Prpf4b | 0496451 | 0453282 Up |
| Ssbp1 | 0496451 | -038434 Down |
| Fermt2 | 0496494 | 0475954 Up |
| D8Ertd738 | 0496978 | 046494 Up |
| Epc2 | 0497053 | 0297815 Up |
| Safb | 0497053 | 0720003 Up |
| E130114P1 | 0497053 | 057825 Up |
| Dusp1 | 0497053 | 0491655 Up |
| Nrxn1 | 0497053 | 039446 Up |
| Rcn1 | 0497252 | 0454451 Up |
| Kmt2a | 0497529 | 0944095 Up |
| Twf1 | 0497977 | 0321719 Up |
| Cap2 | 049814 | 0979672 Up |
| Rnd1 | 049814 | 070444 Up |
| Plcb3 | 049814 | 1610601 Up |
| Abcb7 | 049814 | 0884202 Up |
| Cml1 | 049814 | 1513983 Up |

| Athl1 | 049814 | 0980951 Up |
| --- | --- | --- |
| Arpc2 | 0498836 | -065704 Down |
| Bsg | 0498836 | -026649 Down |
| Vps9d1 | 0498871 | 1036181 Up |
| Bloc1s5 | 0498871 | 0314381 Up |
| Isyna1 | 0498871 | -053356 Down |
| Col4a5 | 0498871 | -068239 Down |
| 2610301B2 | 0499335 | 0213158 Up |
| Wipi1 | 0499335 | 0846912 Up |
| Trappc10 | 0499335 | -046124 Down |
| BC003331 | 0499335 | -046458 Down |
| Impad1 | 0499705 | 0239712 Up |
| Midn | 0499705 | 0192239 Up |
| Gstm1 | 0500214 | 04608 Up |
| Pik3r3 | 0501423 | -063783 Down |
| Numbl | 0501423 | -03363 Down |
| Zfp358 | 0501423 | -050923 Down |
| Cdon | 0501423 | -064813 Down |
| Lemd3 | 0501423 | -045518 Down |
| Cystm1 | 0501423 | -038149 Down |
| Ncoa6 | 0501423 | 0998812 Up |
| Slc16a6 | 0501423 | 0638341 Up |
| Egln3 | 0501423 | 077479 Up |
| Zfhx2 | 0501423 | 0613174 Up |
| Btg3 | 0501423 | 0962719 Up |
| Cramp1l | 0501423 | 0524587 Up |
| Otud4 | 0501839 | -031597 Down |
| Kcnh7 | 0502058 | 0647351 Up |
| Bckdhb | 0502058 | 0648677 Up |
| Ltv1 | 0502058 | 0427347 Up |
| Calu | 0502764 | -090789 Down |
| Emc3 | 0503143 | -095533 Down |
| Ift80 | 0503812 | -036738 Down |
| Amdhd2 | 0503812 | -075906 Down |
| Brcc3 | 0503812 | -150383 Down |
| Xrcc1 | 0503812 | 068114 Up |
| Dhps | 0503812 | 0475692 Up |
| Dxo | 0503812 | 0608325 Up |
| Lsm4 | 0503942 | -022562 Down |
| Hp1bp3 | 0504144 | 0365862 Up |
| Vps26a | 0504173 | 0401585 Up |
| Hsph1 | 0504555 | -079165 Down |
| Bzw2 | 0504555 | 0427015 Up |
| Cbr1 | 0504555 | 0618383 Up |
| Wdr20 | 0505164 | -019628 Down |
| Ccdc58 | 0505164 | -075485 Down |
| Fdps | 0505168 | 021618 Up |
| Slc25a51 | 0505776 | -15642 Down |
| Pmm2 | 0505776 | 0899015 Up |
| C1qbp | 0505858 | -024134 Down |

| S100a6 | 0506452 | -280071 Down |
| --- | --- | --- |
| Prex1 | 0506849 | -015635 Down |
| Acox1 | 0506849 | -020652 Down |
| Cadm1 | 0507027 | 0305572 Up |
| Rab22a | 0507518 | 025895 Up |
| Ptprd | 0507518 | 0388326 Up |
| Clk1 | 0507576 | -034 Down |
| Plekho1 | 0507576 | -04812 Down |
| Stx4a | 0507737 | -022608 Down |
| Rps19bp1 | 0507737 | 056448 Up |
| Prkcsh | 0507802 | -04262 Down |
| Tmem55b | 0507802 | -021207 Down |
| Tbl1xr1 | 0507802 | 0515935 Up |
| Hnrnpa2b1 | 0508551 | 0208762 Up |
| Sf3b5 | 0508839 | -049495 Down |
| Ndufb4 | 0508839 | -064376 Down |
| Ybx1 | 0509181 | 0238264 Up |
| Imp3 | 0509502 | -022116 Down |
| Slc35f1 | 0509502 | -061594 Down |
| Ufl1 | 0511002 | 0332124 Up |
| Kars | 0511002 | 1397784 Up |
| Sncaip | 0511002 | 0587221 Up |
| Tgfb2 | 0511002 | -06617 Down |
| mt-Nd6 | 0511292 | 0840192 Up |
| Phf14 | 0511812 | -027807 Down |
| Bbip1 | 0511812 | 0305718 Up |
| Mrpl9 | 0511814 | -04533 Down |
| Sestd1 | 0512172 | 0232669 Up |
| Gak | 0512172 | 0319191 Up |
| Rrp9 | 0512172 | 0244307 Up |
| AK010878 | 0512172 | -072594 Down |
| Nkain4 | 051231 | 0210116 Up |
| Sars | 0513124 | 0633325 Up |
| Cenpa | 0513528 | -095972 Down |
| Mapk3 | 0513528 | 0477434 Up |
| Nadk2 | 0513528 | 0714578 Up |
| Mt3 | 0513666 | 0280216 Up |
| Ubl3 | 0513666 | -02542 Down |
| Usp22 | 0513666 | 0608997 Up |
| Phf23 | 0513792 | 0759811 Up |
| Dpf2 | 0513792 | 1028279 Up |
| Lmo2 | 0513895 | 2025421 Up |
| Pi4k2b | 0513895 | 103432 Up |
| Ets1 | 0513895 | 1331155 Up |
| Gemin5 | 0513895 | 0872613 Up |
| R3hcc1 | 0513895 | 0803487 Up |
| Ccdc74a | 0513895 | 076116 Up |
| Pola1 | 0513895 | 1259583 Up |
| Ccbl2 | 0513895 | 0825944 Up |
| Use1 | 0514895 | -065117 Down |

| Gamt | 0515459 | 0879538 Up |
| --- | --- | --- |
| Zfp592 | 0515793 | 0156348 Up |
| Bmper | 0515793 | -064309 Down |
| Efna2 | 0515793 | -14034 Down |
| Fam227a | 0515793 | -025709 Down |
| Tmem201 | 0515793 | 0478709 Up |
| Mib2 | 0515793 | 0888645 Up |
| Brap | 0515793 | 0444361 Up |
| Hpgds | 0515793 | 1075404 Up |
| Lrrn1 | 0515793 | 0894261 Up |
| Myo9b | 0515793 | -01893 Down |
| Dvl2 | 0515793 | 0633526 Up |
| Rfng | 0515793 | 04743 Up |
| Iqgap2 | 0515793 | 0566892 Up |
| Ppp3r1 | 0515924 | -033799 Down |
| Gpatch2 | 0516078 | -02753 Down |
| Zfp113 | 0516078 | -080618 Down |
| Spon1 | 0516078 | -025679 Down |
| Avpi1 | 0516078 | -231926 Down |
| Lix1l | 0516078 | 0361611 Up |
| Faf1 | 0516078 | 0485307 Up |
| Nrf1 | 0516078 | 0609821 Up |
| Ppp1r12c | 0516078 | 0787066 Up |
| Lss | 0516078 | 0838181 Up |
| Pxk | 0516078 | 0750162 Up |
| Ptk2 | 0516078 | 0600352 Up |
| Cd99l2 | 0516078 | 0423251 Up |
| Ckb | 0517276 | 0187488 Up |
| Ndufb9 | 0517281 | 0202273 Up |
| Selk | 0517572 | 0784553 Up |
| Zfp106 | 0517765 | 0756105 Up |
| Rsbn1l | 0517765 | -039039 Down |
| Zfp219 | 0517765 | -044234 Down |
| Ankfy1 | 0517765 | 0386904 Up |
| Spg20 | 0517769 | 0275088 Up |
| Rev1 | 0517769 | -092587 Down |
| Cbx6 | 0517804 | 0588938 Up |
| Wbscr22 | 0517804 | 1745439 Up |
| Slc25a3 | 051781 | 0262784 Up |
| Ndufa3 | 0518302 | -025558 Down |
| Arl8b | 0518325 | 0385569 Up |
| Dr1 | 0518469 | 0433229 Up |
| Churc1 | 0518469 | 0418593 Up |
| Med31 | 0518469 | -041846 Down |
| Scaf1 | 0518756 | -023434 Down |
| Dusp8 | 0518756 | -028108 Down |
| Arhgef1 | 0518756 | 0664514 Up |
| Nog | 0518756 | 0852681 Up |
| Ptp4a1 | 0519452 | -016738 Down |
| Rsf1 | 0519452 | 040789 Up |

| Arl3 | 0519571 | 0287575 Up |
| --- | --- | --- |
| Polr2f | 0519571 | -024464 Down |
| Pou3f2 | 0519571 | 0195104 Up |
| Scaf11 | 0519571 | 0771603 Up |
| Pnpla8 | 0521395 | 0612904 Up |
| Kidins220 | 0522162 | -042498 Down |
| Vit | 0522162 | 053631 Up |
| Sbk1 | 052247 | -050051 Down |
| Pigk | 052247 | 039019 Up |
| Slc25a39 | 052247 | 0530083 Up |
| Chmp7 | 0522809 | 0257889 Up |
| Vprbp | 0522809 | -065239 Down |
| Dap3 | 0522809 | -039302 Down |
| Cttn | 0522809 | -04367 Down |
| Smox | 0524818 | -04751 Down |
| Prkcdbp | 0524818 | 0487585 Up |
| Glmp | 0525132 | -033955 Down |
| Cse1l | 0525132 | 0444487 Up |
| Sort1 | 0525132 | 0724117 Up |
| Rdx | 0525399 | -030841 Down |
| Zfp90 | 0525656 | 0213481 Up |
| Trmt2a | 0525656 | 0416295 Up |
| Hprt | 0525656 | 0281734 Up |
| Mapk10 | 0525656 | -053745 Down |
| A2m | 0525893 | 1595389 Up |
| Sf3a1 | 0526214 | 046768 Up |
| 2700060E0 | 0526288 | 0222509 Up |
| Zcchc6 | 0526875 | 0247933 Up |
| Sox8 | 0526875 | -078504 Down |
| Zfp512 | 0526875 | 0504937 Up |
| Acbd6 | 0527061 | -050118 Down |
| Pabpc4 | 0527143 | -068491 Down |
| Rpl10 | 0527781 | -038453 Down |
| Pnrc1 | 0527855 | 0575479 Up |
| Pard6a | 0528496 | -055353 Down |
| Aqp4 | 0528496 | -026567 Down |
| Ccdc53 | 0528496 | -019233 Down |
| Vps37d | 0528496 | 0224258 Up |
| Anapc4 | 0528882 | -017405 Down |
| Tenm3 | 0528919 | -021174 Down |
| Cspp1 | 0529212 | 0306116 Up |
| Rab11fip2 | 052925 | 0628461 Up |
| Chka | 052925 | -046523 Down |
| Tab2 | 052925 | 0843836 Up |
| Nfyb | 052925 | 140774 Up |
| Mat2a | 0529392 | 0776127 Up |
| Slc23a2 | 0529905 | 0807266 Up |
| 2900093K2 | 0529905 | 0640699 Up |
| Rbm38 | 0529905 | 1349782 Up |
| Mthfsd | 0529905 | 0683241 Up |

| Pofut2 | 0529905 | 2747415 Up |
| --- | --- | --- |
| Lrriq1 | 0529905 | 0900656 Up |
| Flywch1 | 0529905 | 0532648 Up |
| Fam198a | 0529905 | 0757846 Up |
| Ccdc124 | 0529948 | -031694 Down |
| Per3 | 0530374 | 0243136 Up |
| Zc3h4 | 0530374 | 018799 Up |
| Fam175a | 0530374 | -08892 Down |
| Intu | 0530374 | 1154813 Up |
| Sema4a | 0530374 | 0514639 Up |
| Elovl1 | 0530374 | 0332591 Up |
| Ckap2 | 0530374 | -112813 Down |
| Rnf182 | 0530374 | 1027997 Up |
| Cd47 | 053125 | -07115 Down |
| Ndufa2 | 0531628 | 022454 Up |
| Umps | 0531905 | 0181744 Up |
| Tomm70a | 0531905 | -025437 Down |
| Bpnt1 | 0531905 | 08747 Up |
| Blcap | 0531905 | 1135514 Up |
| Pde4b | 0531905 | 2384256 Up |
| Ptpn13 | 0531905 | 0564654 Up |
| Ccm2 | 0531905 | 1122142 Up |
| Gpaa1 | 0531905 | 0953954 Up |
| Smg1 | 0531997 | 0267231 Up |
| Mrps6 | 0532618 | 0388582 Up |
| Sox3 | 0532618 | 0564905 Up |
| Cope | 0532988 | 0211649 Up |
| Vamp3 | 053339 | -029496 Down |
| Rac1 | 053368 | 054607 Up |
| Usp47 | 0534284 | 0356021 Up |
| Pafah1b1 | 0534308 | 0281153 Up |
| Odc1 | 0534635 | -019713 Down |
| Tpm4 | 0534636 | -067101 Down |
| Cnih1 | 0534636 | 036393 Up |
| Glg1 | 0534636 | 1094133 Up |
| Efcab14 | 0534755 | 0836297 Up |
| Dchs1 | 0534755 | -034919 Down |
| Armc1 | 0535104 | -048476 Down |
| Chd2 | 0535104 | 0739022 Up |
| Gpx4 | 0535327 | -046305 Down |
| Lims1 | 0536608 | 0710891 Up |
| Ppp1r7 | 0536869 | 0457499 Up |
| Rbpj | 0537137 | -03395 Down |
| Gnl2 | 0538419 | 0203441 Up |
| Pigf | 0538419 | -065628 Down |
| Cyba | 0538915 | -0602 Down |
| Fgd4 | 0539677 | 0654908 Up |
| Snx1 | 0540078 | 0468505 Up |
| Unc119 | 0540078 | -045059 Down |
| Nbea | 054029 | -023966 Down |

| Sap30l | 054029 | 065934 Up |
| --- | --- | --- |
| Serpinb6a | 054029 | -147165 Down |
| Tceal3 | 054029 | -04176 Down |
| Jkamp | 0541007 | 0359532 Up |
| 10-Sep | 0541065 | -023355 Down |
| Gapvd1 | 0541666 | 0232047 Up |
| Snx14 | 0542556 | -172469 Down |
| Coq6 | 0542556 | -1576 Down |
| Lgals3 | 0542556 | -428701 Down |
| Gpr180 | 0542556 | -106642 Down |
| Gm26699 | 0543178 | -037027 Down |
| Ube2n | 0544396 | -104727 Down |
| Srp14 | 0544861 | 0512887 Up |
| Ppp2ca | 0544861 | -017122 Down |
| Trim33 | 0545096 | 1157521 Up |
| Scamp5 | 0545161 | -015905 Down |
| Pdcd7 | 0545161 | -01955 Down |
| Mthfs | 0545161 | -022562 Down |
| Zfp57 | 0545161 | -022773 Down |
| Sart3 | 0545161 | 0492234 Up |
| Gabrb3 | 0545161 | 0415071 Up |
| Sik3 | 0545161 | 026201 Up |
| Fxr2 | 0545161 | -03974 Down |
| Lemd2 | 0545161 | -090696 Down |
| Rrp36 | 0545161 | 0434059 Up |
| Imp4 | 0545197 | -109394 Down |
| Elavl2 | 0545197 | -025381 Down |
| Hspb11 | 0545197 | -051126 Down |
| Tanc2 | 0545197 | -047881 Down |
| Klhl13 | 0545197 | 025321 Up |
| Dmd | 0545197 | 0302764 Up |
| Pcmtd2 | 0545222 | -022005 Down |
| Ints3 | 0545222 | -076653 Down |
| Rhbdd2 | 0545222 | 1452884 Up |
| Zdhhc4 | 0545222 | 0525059 Up |
| Tomm5 | 0545884 | -018487 Down |
| Ndufa10 | 0545953 | -028544 Down |
| Stag2 | 0545953 | -030337 Down |
| Ccdc190 | 0546164 | 0478927 Up |
| Jmjd7 | 0546164 | 0614555 Up |
| Vps16 | 0546164 | 0238436 Up |
| Mfsd8 | 0546164 | 0527375 Up |
| Trp53inp1 | 0546164 | 0749283 Up |
| 1110017D1 | 0546164 | 2141239 Up |
| Adgrl3 | 0546164 | -059657 Down |
| Uspl1 | 0546164 | 0873487 Up |
| Pradc1 | 0546164 | 0572274 Up |
| Arhgef10 | 0546164 | 0574662 Up |
| Zswim4 | 0546164 | 0659142 Up |
| A130014A | 0546164 | 0649716 Up |

| Nup133 | 0546164 | 0764477 Up |
| --- | --- | --- |
| Flnb | 0546164 | 0738316 Up |
| Efna5 | 0546164 | 0280687 Up |
| 1500015O | 0546164 | 0869825 Up |
| Ap1ar | 0546274 | 1016276 Up |
| Hist1h1b | 0546274 | 1989474 Up |
| Reep2 | 0546274 | 1032437 Up |
| Tagln2 | 0546838 | -136988 Down |
| Tspan15 | 0546838 | -023087 Down |
| Tsc22d4 | 0547226 | -036228 Down |
| Snapc5 | 0548496 | 0305876 Up |
| Actr6 | 0548737 | 0740312 Up |
| Coasy | 0548737 | 0260824 Up |
| Cks2 | 0548737 | -025392 Down |
| Camk2g | 0548737 | 0618443 Up |
| Mageh1 | 0549009 | -026943 Down |
| Rbm5 | 0549009 | 0703917 Up |
| Ndufa1 | 0549009 | -061884 Down |
| Hmgcr | 0549326 | 0488575 Up |
| Fmr1 | 054965 | -029641 Down |
| Pid1 | 054965 | 0174343 Up |
| Ythdf2 | 054967 | 0333728 Up |
| Rbm18 | 0550088 | 0543475 Up |
| Socs3 | 0550088 | 091877 Up |
| Ap3d1 | 0550522 | 0260761 Up |
| Tctex1d2 | 0550522 | -016769 Down |
| Cep170 | 0550543 | 1015308 Up |
| Lsm7 | 055115 | 0449443 Up |
| Fbxo3 | 0551392 | 0207919 Up |
| Cic | 0551392 | 0737272 Up |
| Ik | 055267 | -018248 Down |
| Atad3a | 0553782 | -018994 Down |
| Dusp16 | 0553782 | 0609792 Up |
| Cgrrf1 | 0553782 | -059139 Down |
| Ankib1 | 0553782 | 0254122 Up |
| Pnkp | 0553782 | 0236002 Up |
| Neo1 | 0553782 | 0739179 Up |
| Poldip2 | 0553782 | 0371127 Up |
| Kpna1 | 0553782 | 049973 Up |
| Amot | 0553782 | 0659195 Up |
| Cmip | 0553841 | -033523 Down |
| Ppp3cb | 0553841 | 0704812 Up |
| Gkap1 | 0554053 | -038607 Down |
| Ift74 | 0554053 | 0580231 Up |
| Taok1 | 0555721 | -028757 Down |
| Map2 | 0555911 | 0163423 Up |
| H2afz | 0556021 | 0231595 Up |
| Gnai1 | 055654 | -08495 Down |
| Rabl2 | 055654 | -04898 Down |
| Csrp1 | 0556742 | 0461703 Up |

| Gm21092 | 0557424 | 0174556 Up |
| --- | --- | --- |
| Ppt1 | 05577 | -024176 Down |
| Csf1 | 0558191 | -232951 Down |
| 2310057M | 0558191 | -058881 Down |
| Cabin1 | 0558191 | -111081 Down |
| Phka2 | 0558191 | -082282 Down |
| Hells | 0558701 | 0154333 Up |
| Agps | 0558701 | 0886444 Up |
| Agap3 | 0558701 | 1024298 Up |
| Apobec3 | 0558701 | 0282475 Up |
| Zfp740 | 0558701 | 07335 Up |
| Wdr45 | 0558701 | 0885468 Up |
| Dcun1d5 | 0559042 | 1067362 Up |
| Tmx4 | 0559951 | 0307606 Up |
| Ddb1 | 0559951 | -074579 Down |
| Mef2d | 056015 | -0345 Down |
| Unc45a | 056015 | -015335 Down |
| Tbce | 056015 | 0520692 Up |
| Smim13 | 056015 | 0187331 Up |
| Ppif | 056015 | -126959 Down |
| Cpq | 056015 | -016334 Down |
| Abca3 | 056015 | 0433513 Up |
| Ralgds | 056015 | 0422032 Up |
| Eps15l1 | 056015 | -044936 Down |
| Hist1h4i | 056015 | 0245768 Up |
| Ppm1a | 0560489 | -040718 Down |
| Asxl1 | 0560699 | 1211469 Up |
| Meis1 | 0560699 | 1540287 Up |
| Galnt16 | 0560699 | 0981038 Up |
| Thap7 | 0560699 | 1598237 Up |
| Tusc2 | 0560732 | -026218 Down |
| Oxr1 | 0560732 | -034375 Down |
| Paf1 | 0560732 | 0481785 Up |
| Mdp1 | 0560732 | 1002529 Up |
| Gabpa | 0561515 | -028046 Down |
| Ctnna2 | 0561515 | 0699641 Up |
| Far1 | 056188 | -096212 Down |
| Lamb2 | 056188 | 0799529 Up |
| Idh2 | 0562056 | 0226508 Up |
| Mtf2 | 0562265 | 0619118 Up |
| Nsfl1c | 0562553 | 0456953 Up |
| Hnrnpdl | 0562606 | 0438982 Up |
| Rmdn3 | 0562667 | 0555628 Up |
| Lcmt2 | 0562667 | 0553159 Up |
| Gatm | 0562667 | 2823932 Up |
| Ints12 | 0562667 | -046207 Down |
| Tmem39b | 0562667 | 0433271 Up |
| Nat8l | 0562667 | 0551112 Up |
| Alg11 | 0562667 | 018093 Up |
| Inpp5k | 0562667 | 2501929 Up |

| Rab3c | 0562667 | 0748727 Up |
| --- | --- | --- |
| Ift88 | 0562667 | 0280972 Up |
| Entpd7 | 0562667 | 0810906 Up |
| Mtm1 | 0562667 | 0666621 Up |
| B230118H | 0562821 | -048784 Down |
| Snx18 | 0563047 | 1016408 Up |
| Commd10 | 0563047 | 0395199 Up |
| Akr1e1 | 0563047 | 0918662 Up |
| Zbtb20 | 0564179 | 0166166 Up |
| Rad23b | 056453 | 0153142 Up |
| Cct5 | 056489 | -019417 Down |
| Hnrnpf | 0565382 | -017021 Down |
| Tm2d2 | 0565644 | 0352978 Up |
| Smc6 | 0565957 | -034296 Down |
| Asah1 | 0565957 | 0385132 Up |
| Astn1 | 0566558 | -028818 Down |
| Zeb2os | 0566643 | -092438 Down |
| Sgcb | 0566643 | -046659 Down |
| Zbed3 | 0566643 | -028641 Down |
| Elp2 | 0566643 | -036197 Down |
| Stam | 0566643 | -091467 Down |
| Tmem101 | 0566643 | 0266945 Up |
| Ranbp3 | 0566643 | 0312958 Up |
| Spin1 | 0567146 | 0313576 Up |
| Itpr1 | 0567169 | 0441725 Up |
| Gdpd1 | 0567169 | -01889 Down |
| Rnf219 | 0567169 | 0273018 Up |
| Ctps | 0567169 | -025186 Down |
| Ahcyl2 | 0567169 | -053915 Down |
| Ist1 | 0567169 | -056843 Down |
| Fbxo30 | 0567169 | -15333 Down |
| Rab5b | 0567169 | -073009 Down |
| Manf | 0567499 | 0342443 Up |
| Cul3 | 0567546 | -056432 Down |
| Slc39a1 | 0567546 | 0532503 Up |
| Ogt | 0568119 | 0246115 Up |
| Trp53 | 0568395 | 046779 Up |
| Xiap | 0568395 | 0210471 Up |
| Dclk2 | 0568405 | -097648 Down |
| Zfp397 | 0568405 | 021964 Up |
| Rbm33 | 0568405 | 0286786 Up |
| Kif21a | 0569163 | 0360755 Up |
| Scamp1 | 0569771 | -058308 Down |
| Arhgef6 | 0569771 | -061748 Down |
| Fam178a | 0569771 | -076083 Down |
| Ppp2r2a | 0570026 | 0162388 Up |
| Uqcr11 | 0570033 | 031613 Up |
| Tmem237 | 0570324 | 070098 Up |
| Ndufa5 | 0570984 | 0572411 Up |
| Ash2l | 05714 | 1137535 Up |

| Phactr2 | 05714 | 0548206 Up |
| --- | --- | --- |
| Cbx4 | 05714 | 0275048 Up |
| Thap11 | 05714 | -063211 Down |
| Ifnar2 | 05714 | -071891 Down |
| Katnbl1 | 0572341 | 0652376 Up |
| Mkrn2 | 0572341 | 0502713 Up |
| Usp25 | 0572341 | 0465995 Up |
| Fez2 | 0572341 | 0908777 Up |
| Ddx26b | 0572341 | 0552135 Up |
| Fam122a | 0572854 | 0687588 Up |
| Atn1.1 | 0572854 | 031945 Up |
| Gpx8 | 0573487 | 0451231 Up |
| Mbd2 | 0573487 | 0178328 Up |
| Rnf103 | 0574041 | -067449 Down |
| Tyrobp | 0574041 | -094925 Down |
| Cep89 | 0574041 | -075391 Down |
| Ttc13 | 0574041 | -204261 Down |
| Mgat1 | 0574041 | -139277 Down |
| Nat9 | 0574041 | -043481 Down |
| Slc25a14 | 0574041 | -049231 Down |
| Pls3 | 0574041 | -258886 Down |
| Gm13111 | 0574041 | -063414 Down |
| Grsf1 | 0574087 | 0208673 Up |
| Ube2e2 | 0574087 | 0300818 Up |
| Serpinh1 | 0574241 | 0386018 Up |
| Ttyh3 | 0574246 | 0169144 Up |
| Fam3c | 0574363 | -04346 Down |
| Szrd1 | 0574363 | 0858925 Up |
| Snx27 | 0575101 | 0194002 Up |
| Cd24a | 0575231 | -062307 Down |
| Col11a1 | 0575305 | 1283429 Up |
| Cmtm5 | 0575305 | 0823314 Up |
| Cdc42ep1 | 0575305 | 1505178 Up |
| Sdhaf3 | 0575336 | 073055 Up |
| Fam118b | 0575336 | 0258541 Up |
| Ube2z | 0575336 | -161384 Down |
| Eif1a | 057547 | -106329 Down |
| Clpp | 057547 | 1120524 Up |
| Arpc4 | 0575651 | 066096 Up |
| Fdx1 | 0575707 | 0623689 Up |
| Eif2s1 | 0577237 | -047054 Down |
| Ensa | 0577237 | -015973 Down |
| Slc12a2 | 0578077 | 0580341 Up |
| Mpdu1 | 0579188 | -022923 Down |
| Cops2 | 0579188 | 0276408 Up |
| Ndufa9 | 0579188 | 0532104 Up |
| Clic4 | 0579188 | -068627 Down |
| Nipa1 | 0579407 | 0207564 Up |
| Slc17a5 | 0579407 | 038541 Up |
| 4930430F0 | 0579407 | 0524183 Up |

| Atg14 | 0579407 | 024603 Up |
| --- | --- | --- |
| Ajuba | 0579407 | 0617364 Up |
| Spice1 | 0579407 | 0627272 Up |
| Epb41l3 | 0579407 | 0812774 Up |
| Ndc80 | 0579407 | 0292766 Up |
| Kdm3b | 0579407 | 0709011 Up |
| Dcp2 | 0579407 | 0598912 Up |
| Tmem35 | 0579407 | 0377581 Up |
| Skiv2l2 | 0579407 | 0461193 Up |
| Slc31a1 | 0579645 | -036019 Down |
| Gpat4 | 0579645 | -042054 Down |
| Traf4 | 0579645 | -019951 Down |
| Sav1 | 0579645 | -022569 Down |
| Mgll | 0579645 | 045327 Up |
| Saysd1 | 0579645 | 0400377 Up |
| Nedd8 | 0579837 | 0614583 Up |
| 1810058I2 | 058006 | -04157 Down |
| Rcn2 | 058006 | -017693 Down |
| Mex3d | 0580183 | -04857 Down |
| Nrxn3 | 0580183 | -028475 Down |
| Brd3 | 0580788 | 027229 Up |
| Dtd1 | 0581347 | -153549 Down |
| Clip2 | 0581347 | -039922 Down |
| Lsp1 | 0581347 | -050597 Down |
| Nol8 | 0581347 | -028421 Down |
| Pcf11 | 0582474 | 0657488 Up |
| Yme1l1 | 0582545 | 0409659 Up |
| Mcm2 | 058315 | -044962 Down |
| Sgip1 | 058315 | -040714 Down |
| Irak1bp1 | 0583353 | -036592 Down |
| Tmed3 | 0583772 | 0404429 Up |
| Dhx16 | 0583876 | 0340067 Up |
| Ccdc86 | 0583876 | -066285 Down |
| Map4k4 | 0584192 | -029847 Down |
| Kcnk2 | 0584362 | 0221701 Up |
| Snrpd1 | 0584507 | -023199 Down |
| Mmadhc | 0584813 | -02731 Down |
| Msh2 | 0584813 | -021082 Down |
| Mdm2 | 0584813 | 1153747 Up |
| Trim37 | 0584813 | 0442694 Up |
| Wdr43 | 0584813 | -073967 Down |
| Gpbp1l1 | 0585098 | -031443 Down |
| Ift122 | 0585098 | 0216464 Up |
| Mbd6 | 0585098 | -091546 Down |
| Supt20 | 0585098 | 1493721 Up |
| Srsf3 | 058528 | -041709 Down |
| Ccl27a | 0585571 | -040164 Down |
| Sdha | 0585602 | -05923 Down |
| Aldoc | 0585602 | 0483518 Up |
| Gcc2 | 0585764 | 0972297 Up |

| Tmed9 | 0585884 | 0174679 Up |
| --- | --- | --- |
| Morf4l2 | 0585884 | -017468 Down |
| Kif1a | 0586016 | 0646729 Up |
| Chsy1 | 0586016 | 0444468 Up |
| Fam175b | 0586016 | 0276516 Up |
| Trap1 | 0586137 | -082381 Down |
| Gin1 | 0586137 | 0846669 Up |
| Abhd16a | 0586137 | 0330879 Up |
| Smndc1 | 0586432 | -025957 Down |
| Actr2 | 0587721 | 045486 Up |
| Txnl4a | 0588009 | -020332 Down |
| Dab1 | 0588009 | 0415615 Up |
| Psip1 | 0588127 | 0337366 Up |
| Pkn1 | 05885 | -037761 Down |
| Rabl6 | 05885 | 0507302 Up |
| Rras | 05885 | 0685312 Up |
| 2900052N | 05885 | 0585567 Up |
| Nf1 | 05885 | 045389 Up |
| Zranb1 | 0588668 | 0779354 Up |
| Jarid2 | 05894 | 0214349 Up |
| Atp1b2 | 05894 | -023483 Down |
| Gm16286 | 0589665 | 0560076 Up |
| Agpat4 | 0589894 | 0929695 Up |
| Adck4 | 0589894 | 1385658 Up |
| H2-Ke6 | 0590076 | -045033 Down |
| Hmgcs2 | 0590089 | 1105665 Up |
| Fem1b | 0590089 | 072466 Up |
| Ankmy2 | 0590089 | 0453125 Up |
| Spata7 | 0590089 | 1159368 Up |
| Msl3 | 0590089 | 0880236 Up |
| Nampt | 0590089 | -073593 Down |
| Olfml3 | 0590102 | -097889 Down |
| Ctbs | 0590102 | -092547 Down |
| Tnfrsf1a | 0590102 | -041524 Down |
| Atm | 0590102 | -065091 Down |
| Chchd10 | 0590102 | -236242 Down |
| Nudt14 | 0590102 | -069472 Down |
| Clip4 | 0590102 | -073321 Down |
| Gm14698 | 0590102 | -040711 Down |
| Zbtb10 | 0590714 | 0299293 Up |
| Zfp867 | 0590714 | -018721 Down |
| Mrpl12 | 059134 | -06447 Down |
| Ttc3 | 0591622 | -019145 Down |
| Tceal8 | 0592057 | -03901 Down |
| Rab21 | 0592074 | -068123 Down |
| Rad50 | 0592074 | -045172 Down |
| Ube2w | 0592074 | 0271229 Up |
| Cand1 | 0592074 | 031655 Up |
| Ctnnbl1 | 0592786 | 1220437 Up |
| Fam73a | 0592786 | -028787 Down |

| Cnot6 | 0593074 | 0438963 Up |
| --- | --- | --- |
| Anapc1 | 0593615 | 0715546 Up |
| Csnk1g3 | 0593615 | 0684898 Up |
| Vamp7 | 0593615 | 0212536 Up |
| Pnrc2 | 0593615 | 1515388 Up |
| Cnih4 | 0593799 | 0305419 Up |
| Sdcbp | 059456 | 0243404 Up |
| Cdc26 | 059456 | -029881 Down |
| Ubap2 | 0595053 | 0801364 Up |
| Arl4a | 0595053 | 1437563 Up |
| Iws1 | 0595053 | 0532643 Up |
| Casp2 | 0595689 | -054006 Down |
| Carm1 | 0595689 | -043603 Down |
| Rrm2b | 0595689 | -040165 Down |
| Csad | 0595689 | -017872 Down |
| Zfp704 | 0596243 | 040047 Up |
| E2f5 | 0596243 | -018268 Down |
| Nudt2 | 0596243 | 0580943 Up |
| Id2 | 0596243 | -0327 Down |
| Ube2t | 0596377 | -022748 Down |
| Ttl | 0596377 | 0856025 Up |
| Mepce | 0596377 | 0443095 Up |
| Fgd6 | 0596377 | -052429 Down |
| Tmtc2 | 0596377 | 0264312 Up |
| Pomt2 | 0596377 | 0293634 Up |
| Wdr37 | 0596377 | 0163493 Up |
| A830010M | 0596377 | 0322682 Up |
| Lrp3 | 0596476 | -039081 Down |
| Gtf2h1 | 0596476 | -042239 Down |
| Taf13 | 0596519 | 0569201 Up |
| Golm1 | 0596674 | -091366 Down |
| Bap1 | 0596674 | 1048651 Up |
| Zfp462 | 0597161 | -087774 Down |
| Cdc25a | 0597462 | 0560228 Up |
| Atg5 | 0597462 | 0512877 Up |
| Hist1h1e | 0597778 | 046933 Up |
| Fam76a | 0597778 | 031151 Up |
| Scrg1 | 0597778 | 0420004 Up |
| Bfar | 0597778 | 0555331 Up |
| Wiz | 0597972 | -06687 Down |
| Pphln1 | 0597972 | 0529509 Up |
| Pmm1 | 0598137 | 0269513 Up |
| Senp6 | 0598253 | 0155568 Up |
| Nono | 0598253 | -02664 Down |
| Notch1 | 0598253 | 0351242 Up |
| Pfdn4 | 0598874 | -03861 Down |
| Hspa14 | 0598874 | 0367701 Up |
| Acin1 | 0599689 | -029587 Down |
| Lmnb1 | 0600042 | -046486 Down |
| Gm20342 | 0600085 | 0467647 Up |

| Eef2k | 0600085 | 0862114 Up |
| --- | --- | --- |
| Mapkapk2 | 0600085 | 0807305 Up |
| Gsn | 0600085 | 1435086 Up |
| Rassf2 | 0600085 | 038621 Up |
| Otud6b | 0600085 | 0403667 Up |
| Top2a | 0600085 | 0414785 Up |
| Dcaf5 | 0600085 | 0851422 Up |
| Rfc4 | 0600085 | 0481597 Up |
| Ahnak | 0600085 | -131266 Down |
| Mlf2 | 0600156 | 0256058 Up |
| Ndufaf7 | 0601423 | 0396803 Up |
| Fads2 | 0601423 | 0612494 Up |
| Suz12 | 060198 | 0556719 Up |
| Ppid | 0603376 | 0259101 Up |
| Ppp1cc | 0603376 | 0689228 Up |
| Spg7 | 0603489 | 1038423 Up |
| Nlgn2 | 0603489 | 114182 Up |
| 9930021J0 | 0604076 | -061508 Down |
| Ankrd13c | 0604076 | 029461 Up |
| Yif1a | 0604076 | 0538694 Up |
| Arl6ip1 | 0604434 | -045547 Down |
| Specc1 | 0604852 | -058644 Down |
| Pmvk | 0604852 | 0901047 Up |
| Chfr | 0605045 | 0914883 Up |
| Dlx5 | 0605045 | 1420015 Up |
| Prdm5 | 0605045 | 0695905 Up |
| Sipa1l2 | 0605045 | 0869678 Up |
| Map4k5 | 0605045 | 093658 Up |
| Sil1 | 0605045 | 1254794 Up |
| Cask | 0605045 | 0860389 Up |
| D17Wsu92 | 0605045 | 101441 Up |
| Necap2 | 0606062 | -046927 Down |
| Ankle2 | 0606062 | -054473 Down |
| Ift46 | 0606062 | -023111 Down |
| Zmym2 | 0606062 | -015663 Down |
| Ngrn | 0606062 | -057159 Down |
| Dhx38 | 0606062 | 1152354 Up |
| Tax1bp3 | 0606062 | 0982157 Up |
| Mrps14 | 0606163 | -022615 Down |
| Cyp4f15 | 0606278 | 0921759 Up |
| Klhl20 | 0606278 | -037204 Down |
| Smyd2 | 0606366 | -055221 Down |
| Pld1 | 0606366 | -060267 Down |
| Pgm1 | 0606366 | -179137 Down |
| Naa25 | 0606366 | -082402 Down |
| Smpd3 | 0606366 | -148443 Down |
| Slc36a4 | 0606366 | -064456 Down |
| Lsm11 | 0606366 | -043053 Down |
| Fsd1 | 0606366 | -077747 Down |
| Asun | 0606366 | -193737 Down |

| Tmem126a | 0606432 | 1546756 Up |
| --- | --- | --- |
| Cdk11b | 0606602 | 0916729 Up |
| Rab24 | 0606602 | 0581077 Up |
| Sumo3 | 0607362 | 2159268 Up |
| Psmc2 | 0607367 | 0647379 Up |
| Pygb | 0607773 | -037511 Down |
| Glmn | 0607773 | -016304 Down |
| B630005N | 0607773 | 07853 Up |
| Megf10 | 0607932 | -072421 Down |
| Kdelr1 | 0608635 | 0348149 Up |
| Ctnnd2 | 0608635 | -032567 Down |
| Mapt | 0609199 | 0349323 Up |
| Tmem11 | 0609199 | -060996 Down |
| Zdhhc20 | 0609199 | -055164 Down |
| Atox1 | 0609438 | -033161 Down |
| Tspo | 0609646 | -03237 Down |
| Ube4a | 0609646 | 0298169 Up |
| Mapre1 | 0609713 | 0712906 Up |
| Anxa2 | 0609828 | -088352 Down |
| Rc3h1 | 0609944 | 0240656 Up |
| Qser1 | 0609944 | 0520166 Up |
| Zbtb44 | 0609944 | 0165693 Up |
| Trp53bp1 | 0609944 | 0339478 Up |
| Ppp1r15a | 0609944 | 0810149 Up |
| Clk4 | 0609944 | 0860406 Up |
| AW549877 | 0609944 | 0608045 Up |
| Tjp2 | 0609944 | 0981725 Up |
| B4galt3 | 0610191 | -016676 Down |
| Zmynd19 | 0610191 | -030771 Down |
| Magee1 | 0610191 | -026435 Down |
| Lyar | 0610232 | 0716383 Up |
| Srpr | 0610232 | 0706267 Up |
| Zfp617 | 0610339 | -029394 Down |
| Dohh | 0610339 | -030046 Down |
| Pddc1 | 0610339 | -037275 Down |
| Eny2 | 0611812 | -020769 Down |
| Capn2 | 0612158 | -137916 Down |
| Dhx36 | 0612509 | 0712296 Up |
| Sema6c | 061357 | 0772933 Up |
| Arhgef18 | 061357 | 0372554 Up |
| Dhrs4 | 061357 | 0456477 Up |
| Taf9b | 061357 | 0509957 Up |
| Vimp | 061377 | 0463273 Up |
| 1810037I1 | 0613864 | 0150384 Up |
| Dhcr24 | 0614182 | 0239138 Up |
| Nom1 | 0614182 | 0886011 Up |
| Smco4 | 0614182 | 1120949 Up |
| Mtmr3 | 0614182 | 1103007 Up |
| Nop9 | 0614472 | 0711705 Up |
| Arhgap31 | 0614472 | 1125635 Up |

| Arsk | 0614738 | 0695712 Up |
| --- | --- | --- |
| Cxx1a | 0614738 | 0633073 Up |
| Rpn1 | 0615338 | 0747064 Up |
| Pebp1 | 0615405 | -025438 Down |
| Celf4 | 0616184 | 0344732 Up |
| Tmem107 | 0616186 | -019592 Down |
| Fam8a1 | 0616186 | -131326 Down |
| Agpat3 | 0616186 | -078461 Down |
| 2-Mar | 0616186 | 0185483 Up |
| Xpnpep1 | 0617223 | 0810502 Up |
| Kmt2c | 0617227 | 0180521 Up |
| Ezr | 0617227 | 0386989 Up |
| Rlim | 0617907 | 0474557 Up |
| Prpf38a | 0618293 | 0349612 Up |
| Golgb1 | 0619215 | 0994651 Up |
| Prpf8 | 0619215 | 0474224 Up |
| Iscu | 0619402 | 0363054 Up |
| Wdr6 | 0619886 | -028768 Down |
| Pigt | 0619886 | 0520435 Up |
| Csmd2 | 0620169 | 0699923 Up |
| Rab3a | 0620169 | 2220183 Up |
| Tubb4a | 0620169 | 1231885 Up |
| Tmem109 | 0620169 | 0204705 Up |
| Mmd2 | 0620429 | -024134 Down |
| Sec13 | 0621012 | 0552687 Up |
| Csnk2a1 | 0621334 | -058296 Down |
| Chd9 | 0621617 | 0643914 Up |
| Sel1l | 0621617 | 0652571 Up |
| Eif1ax | 0622021 | 031235 Up |
| Frmd8 | 0622023 | -109414 Down |
| Fat1 | 0622041 | -042018 Down |
| Znrf1 | 0622041 | -098123 Down |
| Decr1 | 062254 | 1080198 Up |
| Mllt3 | 062254 | 053254 Up |
| Dbt | 0622603 | -038427 Down |
| Hdac6 | 0622603 | -045449 Down |
| Pdxdc1 | 0622603 | 0782682 Up |
| Gulp1 | 0622828 | -045906 Down |
| Kctd3 | 0622828 | -044495 Down |
| Knstrn | 0622828 | -113332 Down |
| Exoc8 | 0622828 | -04662 Down |
| Atxn7 | 0622828 | -05334 Down |
| Kcnn3 | 0622828 | 0926291 Up |
| Slit2 | 0622828 | 1147027 Up |
| Txnl1 | 0623162 | -018949 Down |
| Pet100 | 0623291 | 0189209 Up |
| Setbp1 | 0623291 | 021877 Up |
| Fam162a | 0623291 | -067657 Down |
| Slc25a1 | 0623291 | 174678 Up |
| Slc30a9 | 062368 | 0220941 Up |

| Arpc3 | 0624029 | 0181932 Up |
| --- | --- | --- |
| Rnf2 | 0624141 | -061629 Down |
| Rfxap | 0624141 | -078424 Down |
| Alg2 | 0624141 | -022362 Down |
| Tmem120a | 0624141 | -069062 Down |
| Ppp6r1 | 0624141 | -08279 Down |
| Cep19 | 0624141 | -049779 Down |
| Sox4 | 0624372 | 0304393 Up |
| Fryl | 0624847 | -05529 Down |
| Klhdc10 | 0624847 | -031386 Down |
| Ifngr2 | 0624847 | -023934 Down |
| Cpsf7 | 0624847 | -055762 Down |
| Eif4a1 | 0625379 | -019905 Down |
| Cpne2 | 0626581 | 0474677 Up |
| Tmem57 | 0627608 | -043928 Down |
| Cdk10 | 0627645 | 0277044 Up |
| Coro2b | 0627645 | 0740092 Up |
| Tapbp | 0627645 | 0435979 Up |
| Btrc | 0627645 | 0509614 Up |
| Brwd3 | 0627645 | 1264503 Up |
| Ier3ip1 | 0627932 | -026456 Down |
| Gstm5 | 062805 | -015099 Down |
| Dnajc5 | 0628402 | -043966 Down |
| Tmem243 | 0628402 | -082492 Down |
| Fam19a5 | 0628402 | -062217 Down |
| Chmp1a | 0628402 | 0389495 Up |
| Jmjd6 | 0628402 | 0299188 Up |
| 2700081O | 0628402 | 0326809 Up |
| Acot2 | 0628422 | 0437712 Up |
| Nlgn1 | 0628422 | 0247791 Up |
| Fubp1 | 0628741 | 0155934 Up |
| Sarnp | 0629092 | 0574538 Up |
| Mlc1 | 0629308 | -03326 Down |
| Msl1 | 0629308 | 0211676 Up |
| Ptp4a2 | 0630876 | 033042 Up |
| Aurka | 0630978 | -090209 Down |
| Mycl | 0630978 | -016957 Down |
| Cdca8 | 0630978 | -018612 Down |
| Cpeb2 | 0630978 | -024031 Down |
| Gins2 | 0630978 | -039318 Down |
| Itpk1 | 0630978 | -141834 Down |
| Rfk | 0631092 | 1978292 Up |
| Lzts2 | 0631092 | 1014743 Up |
| Tor1aip2 | 0631127 | -037503 Down |
| Lsm12 | 0631656 | -152275 Down |
| Tmem33 | 0631999 | -085043 Down |
| Carkd | 0631999 | 075644 Up |
| Celsr1 | 0633004 | 0203156 Up |
| Mta2 | 0633004 | -019564 Down |
| Gnl3l | 0633004 | -020053 Down |

| Tomm34 | 0633004 | 16232 Up |
| --- | --- | --- |
| Mthfd2l | 0633689 | -020444 Down |
| Mvk | 0633689 | -052914 Down |
| Cyhr1 | 0634405 | 0222826 Up |
| Tspan6 | 0634405 | -019928 Down |
| Nme7 | 0634548 | 1698637 Up |
| Cspg5 | 0634779 | 0221977 Up |
| Eif5 | 0634884 | -030083 Down |
| Pycard | 0635 | -03607 Down |
| Psmd10 | 0635 | -046406 Down |
| Ccser2 | 0635081 | -015293 Down |
| Ctage5 | 0635248 | 0292408 Up |
| Adgrl1 | 0635248 | 0225931 Up |
| Ogn | 0635248 | 0364752 Up |
| Dynll2 | 0635295 | -032453 Down |
| Dgkd | 0635455 | 2432534 Up |
| Accs | 0635455 | 0988229 Up |
| Txlna | 0635455 | 0666661 Up |
| Tbc1d10b | 0635455 | 0861394 Up |
| Terf2 | 0635455 | 0836276 Up |
| Plod2 | 0635455 | 0639866 Up |
| Coil | 0635455 | 0337558 Up |
| Pde12 | 0635455 | 0391794 Up |
| Hnrnpk | 0635465 | 0235619 Up |
| Eif4e2 | 0636919 | -100888 Down |
| Sms | 0636919 | -055186 Down |
| Sqle | 0637469 | 0354837 Up |
| Tmed7 | 0637469 | -016857 Down |
| Fdft1 | 0637595 | -065281 Down |
| Kctd10 | 0637943 | 2244074 Up |
| Nek9 | 0637943 | 0240138 Up |
| Trmt1 | 0637943 | -096024 Down |
| Hmgb3 | 0638057 | 0217023 Up |
| Igfbpl1 | 0638076 | -087129 Down |
| Ipo11 | 0638076 | -041159 Down |
| Hmgcs1 | 0638503 | 0383021 Up |
| Dync1h1 | 0638716 | 0357057 Up |
| Zfand2b | 0639481 | -055456 Down |
| Surf1 | 0639481 | -053243 Down |
| Frem2 | 0639481 | 0244838 Up |
| Fkbp15 | 0639481 | -146801 Down |
| Slc25a33 | 0639481 | -110284 Down |
| Sun1 | 0639481 | -056158 Down |
| Slc7a10 | 0639481 | -04899 Down |
| Tmem108 | 0639481 | -102639 Down |
| Btbd2 | 0639481 | -066154 Down |
| Stat3 | 0639481 | -125545 Down |
| Ccdc40 | 0639481 | -022321 Down |
| Wdr35 | 0639481 | 165798 Up |
| Kctd6 | 0639481 | -032472 Down |

| Hacd2 | 0639481 | -0369 Down |
| --- | --- | --- |
| D8Ertd82e | 0639481 | 1434637 Up |
| 0610009O | 0639481 | -058134 Down |
| Col4a2 | 0639481 | 4219014 Up |
| Pgap1 | 0639652 | -033397 Down |
| Hacd1 | 0639652 | 0554339 Up |
| Phactr3 | 0639652 | -036529 Down |
| Mtap | 0639652 | 0276597 Up |
| Ip6k2 | 0639652 | -023064 Down |
| Slf1 | 0639652 | -016964 Down |
| Seh1l | 0639652 | -136201 Down |
| Pak1ip1 | 0640407 | 0179138 Up |
| Lamp2 | 0640407 | 1104919 Up |
| Zfp281 | 0640937 | 0256715 Up |
| Ehbp1 | 0640937 | 0218629 Up |
| Ssrp1 | 0641263 | -015358 Down |
| Taf10 | 0641263 | 0196891 Up |
| Rab1b | 064264 | 0912654 Up |
| Ltbr | 0642802 | 0404409 Up |
| Atp9a | 0642802 | 0262477 Up |
| Rhog | 0642802 | 1083351 Up |
| Nipsnap1 | 0642802 | 033227 Up |
| Axin1 | 0642802 | 0421324 Up |
| Lrif1 | 0642945 | -015236 Down |
| Exosc7 | 0642945 | 0508008 Up |
| Abcc5 | 0642945 | 0306325 Up |
| Tle1 | 0642995 | 0175325 Up |
| Bax | 0643235 | 0854839 Up |
| Dad1 | 0643744 | 0299775 Up |
| Reep3 | 0643757 | 083503 Up |
| Kdm2b | 0644527 | -027404 Down |
| Vrk3 | 0644527 | 030298 Up |
| Insr | 0645092 | 2277162 Up |
| Creld2 | 0645092 | 1049065 Up |
| Hdhd2 | 0645376 | 0288565 Up |
| Pnkd | 064586 | 0445799 Up |
| Ulk2 | 064586 | 0379596 Up |
| Tob1 | 064586 | -123532 Down |
| Mn1 | 0645874 | -032818 Down |
| H2-K1 | 0645874 | -170564 Down |
| Mpdz | 0646663 | -032367 Down |
| Eif2ak1 | 0646663 | 0463159 Up |
| 1700021F0 | 0646663 | -042567 Down |
| Pipox | 0646663 | 0281563 Up |
| Ttc5 | 0646663 | 0337105 Up |
| Tomm6 | 0646663 | 0885775 Up |
| Fam117b | 0646663 | 0168925 Up |
| Bex2 | 0647211 | 0492584 Up |
| Esf1 | 0647565 | -024027 Down |
| Stx18 | 0647627 | -040563 Down |

| Fam126a | 0647664 | -038136 Down |
| --- | --- | --- |
| Fam114a2 | 0647664 | -053419 Down |
| Itga6 | 0647664 | 0638239 Up |
| Cebpb | 0647664 | 2605335 Up |
| C530008M | 0648064 | -031693 Down |
| Dnajc17 | 0648071 | -084919 Down |
| Etv1 | 0648071 | 1460213 Up |
| Ccdc127 | 0648071 | 0770145 Up |
| Apmap | 0648593 | -055064 Down |
| Eml1 | 0648593 | 0368102 Up |
| 1810032O | 0648593 | -019044 Down |
| Smarcad1 | 0648849 | 0600526 Up |
| Eif4g3 | 0649577 | 029013 Up |
| Pa2g4 | 0649577 | -041718 Down |
| Rnaseh2b | 0649577 | 0489802 Up |
| Ypel3 | 0650021 | 0611189 Up |
| Vti1b | 0650724 | 1006096 Up |
| Mrpl47 | 0650899 | 0439157 Up |
| Nfatc3 | 0650899 | 0198964 Up |
| Zfp821 | 0650899 | 0506429 Up |
| Vps11 | 0650899 | 0493139 Up |
| Flot2 | 0650899 | 0600548 Up |
| Alg13 | 0650899 | -039879 Down |
| Schip1.1 | 0650899 | 06928 Up |
| Prr3 | 0650899 | -052202 Down |
| Sh3bgrl3 | 065172 | -029321 Down |
| Zmym3 | 065214 | -062436 Down |
| Dusp18 | 0652887 | 0157975 Up |
| Casc4 | 0652887 | 0345141 Up |
| Polr2m | 0653051 | -055207 Down |
| Dtx3 | 0653051 | 0717359 Up |
| Prkar1a | 0653078 | 0248638 Up |
| Efnb1 | 0653118 | 041837 Up |
| Sec24b | 0653137 | -088144 Down |
| Creb1 | 0654106 | -021415 Down |
| Nudcd3 | 0654106 | 0237751 Up |
| 4921524J1 | 0654346 | 1605836 Up |
| Ap3m1 | 0654346 | 0304163 Up |
| Dennd1b | 0654603 | 0232464 Up |
| Sirt3 | 0654603 | 0190502 Up |
| Cfap97 | 0654603 | -137499 Down |
| Glb1 | 0654603 | -087999 Down |
| Ptrhd1 | 0654603 | -029767 Down |
| Neat1 | 0654603 | -057612 Down |
| Ctps2 | 0654603 | -024674 Down |
| Btg2 | 0654669 | -065221 Down |
| Bet1l | 0654855 | -016108 Down |
| Fsd1l | 0654855 | 0221408 Up |
| Ppib | 0655943 | -024303 Down |
| Nras | 0656087 | 0234528 Up |

| Atf6 | 0656317 | -022769 Down |
| --- | --- | --- |
| Fcer1g | 0656317 | -069059 Down |
| Spopl | 0656317 | -049663 Down |
| Alg14 | 0656317 | -165194 Down |
| Dtx1 | 0656317 | -020899 Down |
| Tspan9 | 0656317 | -0475 Down |
| Map3k1 | 0656317 | 1819983 Up |
| Senp1 | 0656317 | -026608 Down |
| Klhl6 | 0656317 | -08128 Down |
| Pex6 | 0656317 | -020032 Down |
| Zbtb43 | 0656317 | 1344515 Up |
| Plcb4 | 0656317 | 0918965 Up |
| Cog8 | 0656317 | 0985526 Up |
| Baz2a | 0656317 | 1419394 Up |
| Dnajb2 | 0657318 | 0394354 Up |
| Racgap1 | 0657318 | 0167613 Up |
| Snx24 | 0657318 | 0254418 Up |
| Zfp706 | 0657685 | 0508566 Up |
| Sppl2a | 0658795 | -027201 Down |
| Abl2 | 0659219 | 0608046 Up |
| Pip5k1a | 0659219 | 0994315 Up |
| Mterf3 | 0659219 | 0736328 Up |
| Cthrc1 | 0659219 | 30565 Up |
| Arpp19 | 0659791 | -018317 Down |
| Arhgef26 | 0660348 | 0426733 Up |
| Nynrin | 0660443 | 0571224 Up |
| Bcl11b | 0661093 | -145131 Down |
| 5730455P1 | 0661251 | -038904 Down |
| Ccnt1 | 0662411 | -033815 Down |
| Chd3 | 0662846 | -073892 Down |
| Hnrnpm | 066357 | 0325098 Up |
| Dgcr6 | 0665468 | 0182258 Up |
| Luc7l2 | 0665701 | -015363 Down |
| Rrm1 | 0665734 | 0885748 Up |
| Prim1 | 0666329 | -067566 Down |
| Elp3 | 0666329 | 044525 Up |
| Lgals1 | 0666329 | -374249 Down |
| Cnn2 | 0666407 | -28909 Down |
| Golga1 | 0666493 | 2001523 Up |
| Cd82 | 0666493 | 0537433 Up |
| Sdad1 | 0666493 | 0592122 Up |
| Plekha7 | 0666493 | 0671841 Up |
| Sephs2 | 0666493 | 196914 Up |
| Pcid2 | 0666493 | 2258036 Up |
| 1190007I0 | 0666493 | 0571021 Up |
| Snapc1 | 0666493 | 018896 Up |
| Gnpnat1 | 0666493 | 099303 Up |
| Ccar2 | 0666493 | 1022486 Up |
| 1300002E1 | 0666493 | 0497223 Up |
| Yod1 | 0666493 | -1105 Down |

| Leprotl1 | 0666853 | -028632 Down |
| --- | --- | --- |
| Rab34 | 0666853 | -024975 Down |
| Mrpl19 | 0666853 | 027532 Up |
| Ddx6 | 0667548 | -032794 Down |
| Nsmce4a | 0667781 | -022249 Down |
| Lrrc49 | 0667867 | 0621939 Up |
| Sar1a | 066878 | 0337142 Up |
| Sox12 | 066878 | -03109 Down |
| Setd2 | 066878 | 0629848 Up |
| Slc22a17 | 0668788 | -050469 Down |
| Kxd1 | 0668788 | 1129537 Up |
| Ddx10 | 0669694 | -032018 Down |
| Cdkn2aipnl | 0669694 | -024015 Down |
| Golga5 | 0669694 | 0150788 Up |
| Zbtb33 | 0669694 | -021991 Down |
| Sez6 | 0670283 | -035278 Down |
| Csnk2b | 067029 | -044365 Down |
| Fbxo22 | 0670485 | -045562 Down |
| Epb41l5 | 0670674 | 0406175 Up |
| Mrpl24 | 0670894 | -02476 Down |
| Vta1 | 0670918 | -037875 Down |
| Sox1 | 0671231 | 0189114 Up |
| Cyth2 | 0671522 | -026408 Down |
| Zbtb18 | 0671597 | 0690517 Up |
| Camsap2 | 0671963 | 1994329 Up |
| Ptcd3 | 0671963 | 0343833 Up |
| Slc48a1 | 0671963 | 0399406 Up |
| Nfkbil1 | 0671963 | -021136 Down |
| Atp6v1c1 | 067313 | -049062 Down |
| Arnt2 | 067313 | 045032 Up |
| Phlda3 | 067333 | -067252 Down |
| Tubgcp4 | 067333 | -200251 Down |
| Cry1 | 067333 | -029374 Down |
| Mnat1 | 067333 | -033434 Down |
| Spef2 | 067333 | -03309 Down |
| Fbxo45 | 067333 | 0353577 Up |
| Ltbp3 | 067333 | 0240377 Up |
| Nt5c2 | 067333 | -04409 Down |
| Rbm12 | 067333 | -022423 Down |
| Tmem167b | 067333 | 1058897 Up |
| Tmem42 | 067333 | 0565086 Up |
| Ppp2r3d | 067333 | 0576866 Up |
| Cbx8 | 067333 | 0888794 Up |
| Iqcg | 067333 | 0844328 Up |
| Vps4b | 0673469 | 0598777 Up |
| Gramd1a | 0673469 | 0729089 Up |
| Cnot7 | 0674009 | 0155501 Up |
| Tprkb | 0674009 | 0788941 Up |
| Rdh14 | 0674009 | 025335 Up |
| Rsph9 | 0674009 | 0263545 Up |

| Slc2a1 | 0674338 | 0197796 Up |
| --- | --- | --- |
| Ninj1 | 0674538 | -062688 Down |
| Tubb2a | 0674663 | 019964 Up |
| Sgpl1 | 0674668 | -048469 Down |
| Epb41l2 | 0674898 | 0410296 Up |
| Akr1a1 | 0675897 | -029396 Down |
| Rbmx | 0676559 | -017356 Down |
| Polr2c | 0676559 | 0341883 Up |
| Pycr2 | 067729 | 1326607 Up |
| Papss1 | 0677677 | 056623 Up |
| Fam103a1 | 0677677 | -033048 Down |
| Slc38a2 | 067826 | -033034 Down |
| Napg | 067826 | 0252335 Up |
| Pbxip1 | 0678745 | 0845859 Up |
| Vcam1 | 0678745 | -047566 Down |
| Mrpl23 | 0678745 | -074364 Down |
| Rrp1 | 0678745 | 0460925 Up |
| Tbl1x | 0678745 | 0198556 Up |
| Magt1 | 0678775 | 0320099 Up |
| Ccnl1 | 0680363 | -026015 Down |
| Appl1 | 0680363 | -02446 Down |
| Glod4 | 0680363 | 0232581 Up |
| Gtf3c4 | 0680638 | -088722 Down |
| Polr3f | 0680638 | -030421 Down |
| Pik3c2a | 0680638 | -029358 Down |
| Pcdh9 | 0680638 | -037207 Down |
| 1810043G | 0680638 | -03767 Down |
| Rtf1 | 0681022 | -050116 Down |
| Paics | 0681022 | 0215259 Up |
| Ndufa12 | 0681473 | -032848 Down |
| Lsm10 | 0681496 | 0237056 Up |
| Opa3 | 0681496 | 0187379 Up |
| Zfp322a | 0681496 | 0292013 Up |
| Fzd8 | 0681496 | 020272 Up |
| Psma3 | 0681934 | -017976 Down |
| Tfpt | 0682233 | 054776 Up |
| Cars | 0682233 | 0713575 Up |
| Spryd7 | 0682233 | 0467657 Up |
| Rpa1 | 0682233 | -092721 Down |
| Gtf2a1 | 0682934 | 0290511 Up |
| Hrsp12 | 0682934 | -098194 Down |
| Dmwd | 0683266 | 1671536 Up |
| Nim1k | 0683266 | -048198 Down |
| Tpr | 0684161 | 0183617 Up |
| Impdh2 | 0684297 | -025921 Down |
| Cluap1 | 0684599 | 0313178 Up |
| Pfdn1 | 0684749 | 0330984 Up |
| Neu1 | 0684919 | 207907 Up |
| Sh3kbp1 | 0684919 | -017578 Down |
| Vdac3 | 0685727 | -021465 Down |

| Ntmt1 | 0686008 | -112616 Down |
| --- | --- | --- |
| Rfc3 | 0686031 | -024467 Down |
| Slc25a36 | 0686031 | -021899 Down |
| Snx16 | 0686734 | 0265554 Up |
| Arhgap17 | 0686734 | 0161546 Up |
| Txlng | 0686734 | 0160713 Up |
| Eif3c | 0686762 | 0392929 Up |
| Tuba1b | 068701 | 0487315 Up |
| Cib2 | 068713 | 0400802 Up |
| Tars | 068713 | 0297469 Up |
| Fnta | 06872 | 054309 Up |
| Lrrcc1 | 0687837 | 0645954 Up |
| Ufsp2 | 0687837 | 1708643 Up |
| Tbcel | 0687837 | 0609403 Up |
| Prpsap1 | 0687837 | 0840201 Up |
| Bach1 | 0687837 | 1427602 Up |
| Mark1 | 0687927 | -019266 Down |
| Wipi2 | 0687927 | -024544 Down |
| Aars | 0687927 | 1029501 Up |
| Arpc1a | 0688124 | -066527 Down |
| Tsc22d1 | 0688124 | -097891 Down |
| Vkorc1 | 0688345 | 0195218 Up |
| Xpot | 0688345 | 12354 Up |
| Txndc5 | 0688351 | -110953 Down |
| Cnot1 | 0688648 | 0298336 Up |
| Zdhhc6 | 0688648 | 0271654 Up |
| Mcm7 | 0689149 | -100194 Down |
| Pex16 | 0690512 | 0307409 Up |
| Slc35c2 | 0690512 | 1672886 Up |
| Mtg2 | 0690512 | -193631 Down |
| Ppat | 0690512 | -067962 Down |
| N4bp2l1 | 0690512 | -025052 Down |
| Zbtb4 | 0690512 | -024465 Down |
| Cmc4 | 0690512 | -042916 Down |
| Zbtb6 | 0690512 | 0821711 Up |
| Gtpbp10 | 0690512 | 0682043 Up |
| Pccb | 0690512 | 0976433 Up |
| Pik3r4 | 0690512 | 126412 Up |
| Hexim1 | 0690512 | 090735 Up |
| Lin52 | 0690512 | 0274948 Up |
| Btbd7 | 0690512 | 0974725 Up |
| Znrd1as | 0690512 | 0719679 Up |
| Fam111a | 0690512 | 1195264 Up |
| Zcchc12 | 0690512 | 0429975 Up |
| Ago1 | 069096 | 0356053 Up |
| Dhfr | 069096 | 0332873 Up |
| B4gat1 | 069096 | 1288813 Up |
| Clip1 | 069096 | 0172987 Up |
| Lztfl1 | 0692015 | 025597 Up |
| Max | 069258 | 133839 Up |

| 2810417H1 | 069258 | 1806143 Up |
| --- | --- | --- |
| Stub1 | 0692705 | -030122 Down |
| Ccnt2 | 0693383 | -01555 Down |
| Tmem263 | 0693383 | -085977 Down |
| Naa50 | 069359 | 0899159 Up |
| Hat1 | 0693751 | 0536968 Up |
| Fam195b | 0693751 | -018589 Down |
| Rbbp4 | 0694864 | 028707 Up |
| Dapp1 | 0695064 | -039005 Down |
| Krr1 | 0695064 | -059061 Down |
| Cdkal1 | 0695064 | -056552 Down |
| E530001K1 | 0695064 | 0209068 Up |
| Timm22 | 0695231 | 0569557 Up |
| Mms19 | 0695231 | 0381884 Up |
| Mef2a | 0695469 | -033181 Down |
| Rnf214 | 0695469 | 0210049 Up |
| Gps2 | 0695469 | 0421327 Up |
| Acap2 | 0695469 | 0427865 Up |
| Dyrk1a | 0695469 | 0217835 Up |
| Timm13 | 0696382 | -025622 Down |
| Hmgn2 | 0696768 | 0276798 Up |
| Npepps | 0696899 | 0391613 Up |
| Tnpo1 | 0697073 | -020016 Down |
| Rb1cc1 | 0697073 | 0942751 Up |
| Cotl1 | 0697241 | 0437745 Up |
| Cbx3 | 0697627 | 0265384 Up |
| Atp5a1 | 0697827 | 0166102 Up |
| Tead1 | 0697959 | 0500008 Up |
| Pgk1 | 0697959 | 0291338 Up |
| Angel2 | 0697959 | 0433355 Up |
| Ttc9b | 0697959 | -10401 Down |
| Btbd1 | 0697959 | -046297 Down |
| Ywhaq | 069799 | -034913 Down |
| Crat | 0698113 | -091471 Down |
| Macrod2 | 0698113 | 1748573 Up |
| F3 | 0698113 | 0325869 Up |
| Odf2l | 0698113 | 0417418 Up |
| Srgap1 | 0698113 | 0384406 Up |
| Haus4 | 0698113 | -09764 Down |
| Sfrp5 | 0698113 | -097005 Down |
| Fam69a | 0698113 | -023841 Down |
| Pin4 | 0698277 | 0308002 Up |
| Dctn6 | 0698978 | -044424 Down |
| Limd1 | 0698978 | -121118 Down |
| Wdr18 | 0698978 | -036739 Down |
| Zfx | 0698978 | -036649 Down |
| Pcmt1 | 0699302 | -024661 Down |
| Smarcc1 | 0699536 | 0162772 Up |
| Urod | 0699586 | 0450656 Up |
| Necap1 | 0700273 | 0281713 Up |

| Tdp1 | 0700273 | -038956 Down |
| --- | --- | --- |
| Senp2 | 0700273 | 0221501 Up |
| Olig2 | 0700273 | 0897717 Up |
| Atp1b3 | 0700458 | 0853276 Up |
| Rnf11 | 070073 | 0360397 Up |
| Tjp1 | 070073 | 0224555 Up |
| Epha3 | 0700971 | 0294386 Up |
| 1110037F0 | 0701414 | 0181344 Up |
| Toporsos | 0701414 | -081538 Down |
| Txnip | 0701626 | -127774 Down |
| Pou2f2 | 0701626 | -016202 Down |
| Arl13b | 0701626 | 0193938 Up |
| Arhgdia | 070173 | 0401482 Up |
| Plaa | 0702136 | -155612 Down |
| Nup214 | 0702319 | 0859132 Up |
| Dcbld1 | 0702319 | 2193144 Up |
| Specc1l | 0702319 | 0365763 Up |
| Ifnar1 | 0702319 | 1137164 Up |
| Nln | 0702597 | -029796 Down |
| Tcea1 | 0703236 | 0169143 Up |
| 6-Mar | 070335 | 1015096 Up |
| Fam76b | 0703746 | 0718711 Up |
| Vcan | 0703802 | -018377 Down |
| Notch2 | 0704967 | 0401136 Up |
| Dennd5a | 0705031 | 0372248 Up |
| Zfp326 | 0705809 | -056951 Down |
| Kpna3 | 0705809 | 0189096 Up |
| Rap2c | 0705809 | 0162804 Up |
| Vasp | 0706989 | -044131 Down |
| Socs6 | 0706989 | -023587 Down |
| Capn10 | 0707854 | 0386836 Up |
| Cep250 | 0707854 | 0176022 Up |
| Usp42 | 0707854 | -023638 Down |
| Bcl2l13 | 0707854 | 0305429 Up |
| Bambi | 0707854 | 0282594 Up |
| Apitd1 | 0707854 | -022302 Down |
| Adrbk2 | 0707854 | -016841 Down |
| Papd4 | 0707854 | 0806637 Up |
| Them4 | 0707854 | 1185935 Up |
| Toe1 | 0707854 | 0739385 Up |
| Creb3l2 | 0707854 | 1181001 Up |
| Ccdc8 | 0707854 | 0606902 Up |
| Arntl | 0707854 | 0620119 Up |
| Bbs2 | 0707854 | 0642885 Up |
| Mmgt2 | 0707854 | 0838339 Up |
| Fbf1 | 0707854 | 1244822 Up |
| Rictor | 0707854 | 0797378 Up |
| Ddx23 | 0707854 | 0641634 Up |
| Shoc2 | 0707997 | 1261716 Up |
| Haus7 | 0708088 | 0341868 Up |

| Abcd3 | 0708625 | 0305768 Up |
| --- | --- | --- |
| 1810043H0 | 0708625 | -082325 Down |
| Slc24a5 | 0708898 | -026994 Down |
| Ube2j1 | 0709066 | 0274599 Up |
| Gpn1 | 07096 | -037446 Down |
| Bcl7b | 07096 | -03421 Down |
| Cbfb | 07096 | -032618 Down |
| Slc50a1 | 0709962 | -045483 Down |
| Ehmt1 | 0709962 | 0441705 Up |
| Erbb2ip | 0709962 | 0353226 Up |
| Nap1l5 | 0712036 | -024827 Down |
| Mau2 | 0712036 | -016973 Down |
| Cul4a | 0712036 | 0161822 Up |
| Nsrp1 | 0712036 | 0487204 Up |
| Myl12a | 0712683 | 024547 Up |
| Nes | 0712683 | 0269119 Up |
| Gng2 | 0712757 | 0867517 Up |
| Stip1 | 0712757 | -022884 Down |
| Ptprf | 0712867 | 0388595 Up |
| Nipsnap3b | 0712887 | 0338752 Up |
| 1110004F1 | 0713434 | -033848 Down |
| Immt | 07136 | 0503278 Up |
| Ncstn | 0713654 | 0522723 Up |
| Sin3a | 0713654 | 0345743 Up |
| Tpm2 | 0714126 | -016965 Down |
| Ahdc1 | 0714126 | 0397193 Up |
| Trim41 | 0714126 | 0613973 Up |
| Pip4k2a | 0714126 | -177791 Down |
| Wdtc1 | 0714126 | -053451 Down |
| Thumpd3 | 0714126 | -082314 Down |
| E2f4 | 0714126 | -044959 Down |
| 9-Mar | 0714126 | -146912 Down |
| Sipa1 | 0714126 | -044742 Down |
| Psmd6 | 071443 | 0178063 Up |
| Csnk1e | 071443 | 0393252 Up |
| Copb2 | 07149 | 0652946 Up |
| Speg | 0714996 | -026186 Down |
| 1110051M | 0714996 | -027956 Down |
| Nck1 | 0714996 | -032097 Down |
| Ndel1 | 0714996 | -061534 Down |
| Fam172a | 0714996 | 020424 Up |
| Bcl7a | 0715535 | -034588 Down |
| Nrep | 0715675 | 1366205 Up |
| Shroom3 | 0715751 | -03635 Down |
| Usp39 | 0715751 | -017848 Down |
| Mex3b | 0715751 | -025384 Down |
| Zscan26 | 0715751 | 0161406 Up |
| Timmdc1 | 0715751 | 0171533 Up |
| Akap9 | 071585 | 0614645 Up |
| Ap2a1 | 071602 | -032546 Down |

| Pacs2 | 071602 | -01954 Down |
| --- | --- | --- |
| Nipbl | 0716247 | -022901 Down |
| Fgfbp3 | 0716247 | 1075906 Up |
| Arhgef7 | 0716617 | 0329224 Up |
| Sall2 | 0716617 | 1417588 Up |
| Anapc15 | 0716678 | 1037302 Up |
| Etv5 | 0716678 | 0888217 Up |
| Btbd10 | 0716909 | 0470075 Up |
| Xbp1 | 0716909 | 0664245 Up |
| Wnt7b | 0716909 | 0781162 Up |
| Cct7 | 0717872 | -023535 Down |
| Nek6 | 0717988 | 0499881 Up |
| Pkia | 0718313 | -024352 Down |
| Arf6 | 0718841 | 061609 Up |
| U2af1 | 0719037 | 051137 Up |
| Slc15a2 | 0720788 | 0273355 Up |
| Pgls | 0721884 | -048285 Down |
| Meg3 | 0721884 | 0175905 Up |
| Anapc10 | 0722038 | 096359 Up |
| Rcbtb2 | 0722038 | 0197394 Up |
| Mrps28 | 0722038 | 0263328 Up |
| Haghl | 0722038 | 0318424 Up |
| Ift57 | 0722038 | 0371481 Up |
| Uchl1 | 0722719 | 0825761 Up |
| Exoc6b | 0722998 | 0442205 Up |
| Cep162 | 0722998 | 0191162 Up |
| Stmn4 | 0722998 | -062187 Down |
| Npc1 | 0722998 | 0197742 Up |
| Lrch2 | 0722998 | 0362367 Up |
| Dpm1 | 0723415 | 0182657 Up |
| Dedd | 0723534 | 084059 Up |
| Ccdc50 | 0723865 | 0180409 Up |
| Lsm1 | 0724226 | 0300971 Up |
| Elk4 | 0724244 | 0183873 Up |
| Slc44a1 | 0724244 | -022826 Down |
| B4galt2 | 0724244 | -023426 Down |
| Psmb1 | 0724609 | 0348534 Up |
| Dalrd3 | 0725179 | -017549 Down |
| Akap6 | 0725179 | -030304 Down |
| Supt5 | 0725179 | -02069 Down |
| Fn1 | 0725349 | 0509518 Up |
| 8-Mar | 0725349 | 0298743 Up |
| Stard10 | 0725349 | -019739 Down |
| Wrn | 0725349 | -138074 Down |
| Cacng4 | 0725349 | 0330779 Up |
| Cmpk2 | 0725349 | 0324355 Up |
| Cox16 | 0725349 | 1088675 Up |
| Mrpl44 | 0725349 | 0800731 Up |
| Mertk | 0725349 | 0542942 Up |
| Lrrc8b | 0725349 | 0724399 Up |

| Pptc7 | 0725349 | 0561778 Up |
| --- | --- | --- |
| Agfg2 | 0725349 | 0589337 Up |
| Brpf1 | 0725349 | 0534861 Up |
| Nomo1 | 0725349 | -124749 Down |
| Cend1 | 0725349 | 1090422 Up |
| Cnot10 | 0725349 | 055216 Up |
| Nrbf2 | 0725349 | 0563036 Up |
| Lyrm9 | 0725349 | 0376575 Up |
| Slc25a19 | 0725349 | 0440801 Up |
| Rhpn1 | 0725349 | 053881 Up |
| Tnrc6a | 0725381 | 0226654 Up |
| Clcn4 | 072705 | 0274649 Up |
| Pgap2 | 072705 | -099228 Down |
| Ap1s1 | 0727399 | 0356599 Up |
| 2700094K1 | 0727846 | -024049 Down |
| Nus1 | 0728074 | 0954488 Up |
| Stx5a | 0728074 | 1468099 Up |
| Tspan13 | 0728113 | 0197164 Up |
| Gsta4 | 0728669 | -039031 Down |
| Ugdh | 0728669 | 0435496 Up |
| Mettl9 | 0729051 | 0502799 Up |
| Mocs2 | 0729066 | 0354342 Up |
| Fndc3a | 0729759 | 0276216 Up |
| Gpd2 | 0729998 | -110685 Down |
| Prpf38b | 0730091 | 0987294 Up |
| Cycs | 0730091 | -035671 Down |
| Dok5 | 0730265 | 0208715 Up |
| Mxra8 | 0730265 | 0390762 Up |
| Zfp574 | 0730265 | 0413266 Up |
| Nkiras2 | 0730265 | 0356373 Up |
| Pik3r1 | 0730265 | 0777413 Up |
| Zbtb14 | 0730265 | 0479434 Up |
| Pik3c3 | 0730265 | 2149592 Up |
| Gucy1a3 | 0730265 | -04638 Down |
| Zc3h12c | 0730265 | -08612 Down |
| Cd276 | 0730265 | -049033 Down |
| Myo1e | 0730265 | -040872 Down |
| Klc1 | 073084 | 0779162 Up |
| Mpzl1 | 0730894 | -027252 Down |
| Phf6 | 0730894 | -01534 Down |
| Osbpl9 | 0730995 | 1368695 Up |
| Naa40 | 0730995 | -057769 Down |
| Ttf1 | 0731346 | 0853344 Up |
| Abhd11 | 0731346 | 0323444 Up |
| Pcbd2 | 0731346 | 0430507 Up |
| Sptssa | 0731586 | 0542548 Up |
| Fbxl5 | 0731603 | 0268471 Up |
| Slc38a3 | 0731603 | 0541551 Up |
| Ntn4 | 0731603 | 0624358 Up |
| Adgra3 | 0731751 | 0150893 Up |

| Socs7 | 0731751 | -022472 Down |
| --- | --- | --- |
| Rnh1 | 0731928 | 0574381 Up |
| Dynlt1f | 0731928 | -038189 Down |
| Hypk | 0732792 | 0271106 Up |
| Lfng | 0733566 | 0238412 Up |
| Deb1 | 0733566 | 0211541 Up |
| Lnp | 0733566 | -064622 Down |
| Smim11 | 0733825 | -023108 Down |
| Fam181b | 0734183 | 0360677 Up |
| Hsdl2 | 0734183 | 0323864 Up |
| Ccng2 | 0734183 | 0274826 Up |
| Rbm22 | 0734183 | 0541595 Up |
| P4hb | 0734764 | -067604 Down |
| Atf7ip | 0735668 | 0265108 Up |
| Fgfr2 | 0735757 | 0224766 Up |
| Cox6c | 0735917 | 0177213 Up |
| Cpe | 0736388 | -030439 Down |
| Ttc33 | 0737022 | 0405288 Up |
| Mapk8ip2 | 0737022 | 0242912 Up |
| Mrps18b | 0737022 | 0162528 Up |
| Psmc3 | 0737084 | 0192918 Up |
| Golga4 | 0737212 | 0525526 Up |
| Zfp451 | 073742 | 0411367 Up |
| Ndufv2 | 0737708 | -02619 Down |
| Cdc73 | 0738403 | 0281691 Up |
| Aig1 | 0738403 | -031212 Down |
| Abhd17b | 0738403 | -034967 Down |
| Dync1li1 | 0738403 | 0467194 Up |
| Arid4a | 0738403 | 0466581 Up |
| Ptgr2 | 0738403 | 0253432 Up |
| Cnppd1 | 0738988 | -048751 Down |
| Cds2 | 0738988 | -077094 Down |
| Scpep1 | 0738988 | 0205159 Up |
| Lxn | 0738991 | 0799259 Up |
| Nfasc | 0739186 | 0530799 Up |
| Ska2 | 0739186 | 1026566 Up |
| Rnf139 | 0739186 | 1120403 Up |
| Osbpl1a | 0739186 | 1071712 Up |
| Fibp | 0739328 | 0397342 Up |
| Slc25a17 | 0739328 | 0510008 Up |
| Eif3e | 0739587 | -022208 Down |
| Bcl2 | 0739749 | -018791 Down |
| Nup88 | 0740529 | 0853811 Up |
| Wdfy3 | 0740545 | -019347 Down |
| Smarce1 | 0741156 | 0839223 Up |
| Zmynd11 | 0741593 | 0583577 Up |
| Tulp4 | 0741593 | 0204151 Up |
| Adam17 | 0742428 | 0383036 Up |
| Kpna4 | 0742599 | 0292853 Up |
| Cisd1 | 0742905 | 0224409 Up |

| Luc7l | 0742905 | 0773214 Up |
| --- | --- | --- |
| Uba2 | 0742906 | -077435 Down |
| Lin9 | 0742989 | -018317 Down |
| Crybb1 | 0742989 | 029615 Up |
| Iqub | 0742989 | 022499 Up |
| Rcor3 | 0742989 | 0364414 Up |
| Mapre3 | 0742989 | 1206858 Up |
| Hscb | 0742989 | 0567946 Up |
| Wasf3 | 0742989 | 0619365 Up |
| Borcs5 | 0742989 | 039734 Up |
| Cln3 | 0742989 | 0778021 Up |
| Ccdc191 | 0742989 | 0417594 Up |
| Usp51 | 0742989 | 0609583 Up |
| Mvb12b | 0743548 | -032209 Down |
| Pole4 | 0743597 | -040477 Down |
| Derl1 | 0743597 | -051098 Down |
| Fam214b | 0745407 | -060665 Down |
| Os9 | 0746394 | -015413 Down |
| Dstyk | 0746397 | 072819 Up |
| Sp9 | 0746397 | 0622821 Up |
| Manea | 0746397 | 0512745 Up |
| Nacc1 | 0746397 | 0464625 Up |
| Gnpda2 | 0746418 | 0256933 Up |
| Ifngr1 | 0746418 | -02994 Down |
| Abhd4 | 0746418 | 0918764 Up |
| Crb2 | 0746525 | 0430614 Up |
| Ampd2 | 0746525 | -024381 Down |
| Cenpe | 0746525 | 030178 Up |
| Inppl1 | 0746525 | 0328628 Up |
| Ptrh2 | 0746525 | 0311843 Up |
| Atp11b | 0746525 | -053444 Down |
| Klhdc8b | 0746525 | -02981 Down |
| Fzr1 | 0746525 | -077998 Down |
| Zfp101 | 0746525 | -036153 Down |
| Rasgrp2 | 0746525 | -25345 Down |
| Nhlrc2 | 0746525 | -015091 Down |
| Bms1 | 0746974 | 0549816 Up |
| Rnf38 | 0746974 | -058164 Down |
| Lonp1 | 0746974 | -174356 Down |
| Zbtb38 | 0747053 | 034482 Up |
| Gm8186 | 0747053 | 0580987 Up |
| Exoc7 | 0747299 | -02999 Down |
| Rap1b | 0747349 | -043822 Down |
| Acadl | 0747349 | 0541459 Up |
| Mrpl21 | 0747349 | 0282715 Up |
| Srsf7 | 074745 | -024144 Down |
| Mtfr1l | 0748966 | 1148288 Up |
| 5-Mar | 0748966 | -015945 Down |
| Mrps36 | 0750451 | 0724852 Up |
| Csnk1g2 | 0750451 | -029859 Down |

| Smarcd3 | 0751134 | 031688 Up |
| --- | --- | --- |
| Zfp277 | 0751134 | -096783 Down |
| Rae1 | 0751321 | -02241 Down |
| Tcaf1 | 0751321 | -030745 Down |
| Pou2f1 | 0751444 | -043576 Down |
| Ankrd10 | 0751705 | 0168893 Up |
| Dcaf13 | 0751705 | 1206152 Up |
| Zfp30 | 0753829 | -016967 Down |
| Pcgf2 | 0753829 | -045025 Down |
| Smpdl3a | 0754749 | 0599573 Up |
| Col5a2 | 0754947 | 0471959 Up |
| Snrnp27 | 0755249 | -056902 Down |
| Ezh2 | 0755249 | 0327758 Up |
| Strn3 | 0755249 | 0879388 Up |
| Atp6v1a | 0755482 | -045367 Down |
| Cdc42bpa | 0755703 | -021462 Down |
| Tvp23b | 0755999 | 0732693 Up |
| BC003965 | 0755999 | -034924 Down |
| Anp32e | 0756201 | 0509934 Up |
| Swt1 | 0756271 | -089787 Down |
| Sidt2 | 0756271 | 0420196 Up |
| Slc4a7 | 0756271 | 0342825 Up |
| Ptbp3 | 0756271 | -041041 Down |
| Lap3 | 0756271 | 0316959 Up |
| Csde1 | 0756834 | 0291953 Up |
| Map7d1 | 0758222 | -133936 Down |
| Psmg1 | 0758222 | -028872 Down |
| Dync2li1 | 075867 | 0277016 Up |
| Ednrb | 0758998 | 0242945 Up |
| Tnrc6b | 0758998 | 0215418 Up |
| Jagn1 | 075934 | 1424507 Up |
| Zyx | 075934 | -104442 Down |
| Ppp2r3c | 075934 | -03123 Down |
| Cd151 | 0759908 | 1887818 Up |
| Azi2 | 0759908 | 0410255 Up |
| Dnajc13 | 0759908 | 0705241 Up |
| Rap1gap | 0760765 | 030609 Up |
| Fign | 0760765 | 0394536 Up |
| Rab13 | 0760765 | 0489571 Up |
| Mtor | 0760765 | 0371315 Up |
| Loxl1 | 0760765 | 0303024 Up |
| Kdm4b | 0760765 | 0167329 Up |
| Pgd | 0760898 | -036898 Down |
| Dnajc21 | 0760898 | -074684 Down |
| Cdo1 | 0760898 | -048772 Down |
| Cs | 0761122 | -017687 Down |
| Ndufb5 | 0761157 | 0588513 Up |
| Coq4 | 0761285 | 0353878 Up |
| Taok2 | 0761285 | 032984 Up |
| Scd1 | 0761285 | 0875217 Up |

| Ccdc186 | 0761285 | 0370694 Up |
| --- | --- | --- |
| Eid1 | 0761822 | -015074 Down |
| Slc27a1 | 0762224 | -031311 Down |
| Rpa2 | 0762295 | 037199 Up |
| Plagl2 | 0762866 | 0672638 Up |
| D430042O | 0762866 | 0445936 Up |
| Ncln | 0762866 | 0423338 Up |
| Slc4a3 | 0762899 | 0219243 Up |
| Pigs | 0762899 | -037646 Down |
| Pacs1 | 0762899 | 0708048 Up |
| Tesk1 | 0762899 | -050089 Down |
| Arhgap18 | 0762899 | -097016 Down |
| Tbl3 | 0762899 | -016557 Down |
| Zfyve27 | 0762899 | -033621 Down |
| Pitpna | 0763197 | 0218789 Up |
| Abhd14a | 0763374 | 0263344 Up |
| Bmpr1a | 0763374 | 0450939 Up |
| Etf1 | 0765082 | -063356 Down |
| Lrp8 | 0765082 | -0581 Down |
| Tex9 | 0765082 | 0350301 Up |
| Mras | 0765082 | 01584 Up |
| Slc22a23 | 0765082 | 0231194 Up |
| Sybu | 0765082 | -062517 Down |
| Fam124a | 076533 | 0418094 Up |
| Peg3 | 0765443 | -028549 Down |
| Cenpb | 0765689 | 0635649 Up |
| Sbf2 | 0765689 | 0719347 Up |
| Fscn1 | 0766355 | -028239 Down |
| Rgcc | 0766557 | 0232001 Up |
| Atp2c1 | 0767225 | -073807 Down |
| Phc2 | 0767346 | 0406477 Up |
| Aftph | 0767725 | 0319022 Up |
| Chmp6 | 0767725 | -084937 Down |
| Ctnnbip1 | 0768296 | 0424949 Up |
| Huwe1 | 0768296 | 0428099 Up |
| Zfp532 | 0768762 | 1483256 Up |
| Cpne3 | 0769094 | 0534901 Up |
| Wdr47 | 0770192 | -082438 Down |
| B2m | 0770192 | 0223743 Up |
| Ccnc | 0770192 | 0261998 Up |
| Cdk13 | 0770192 | -037011 Down |
| Pdcd2 | 0770192 | -017142 Down |
| Usp24 | 0770192 | 0645922 Up |
| As3mt | 0770192 | 0269872 Up |
| Tgfbrap1 | 077081 | 0865396 Up |
| Mok | 077081 | 0727747 Up |
| Fam45a | 077081 | 0471349 Up |
| Hist3h2a | 0771007 | -02344 Down |
| Erlec1 | 0771442 | -03176 Down |
| Klf13 | 0772178 | 0994386 Up |

| Dtna | 0772458 | -024932 Down |
| --- | --- | --- |
| Wdr89 | 0773341 | 1139069 Up |
| Ier2 | 0773341 | 0933097 Up |
| N4bp2l2 | 0773534 | -103618 Down |
| Sepw1 | 0773781 | 0291903 Up |
| Snrk | 077457 | 145439 Up |
| Mphosph8 | 0774719 | 0662289 Up |
| 2610001J0 | 0775005 | -022643 Down |
| Nifk | 0775479 | 024403 Up |
| Ubr4 | 0775479 | 0345762 Up |
| Secisbp2l | 077549 | 0657888 Up |
| Pias1 | 077549 | -04637 Down |
| Creg1 | 0776261 | 0288361 Up |
| Afap1 | 0776261 | 0726575 Up |
| Phc1 | 0776261 | 0231891 Up |
| Prrc1 | 0776261 | 0531377 Up |
| Soat1 | 0777454 | -03494 Down |
| Zkscan8 | 0777711 | -049888 Down |
| Stox2 | 0777915 | -02656 Down |
| Psme1 | 0777915 | -045856 Down |
| Txnrd1 | 0778356 | -015912 Down |
| Mrpl22 | 0778356 | 0281996 Up |
| Phactr4 | 0778529 | 0734799 Up |
| 2510039O | 0778668 | 069437 Up |
| Gsap | 0778668 | 0630181 Up |
| Lmo3 | 0778668 | 0330174 Up |
| Zbtb11 | 0778668 | 0518135 Up |
| Casq1 | 0778668 | 1161846 Up |
| Tcp11l1 | 0778668 | 1118559 Up |
| Inafm2 | 0778668 | 0398039 Up |
| Rbm12b2 | 0778668 | 0334816 Up |
| Cpt1c | 0778668 | 0324783 Up |
| Ano1 | 0778668 | 0751189 Up |
| Fdxacb1 | 0778668 | 0474762 Up |
| Ddit3 | 0778668 | 1038675 Up |
| Dhrs7 | 0778668 | 0343824 Up |
| Tbc1d7 | 0778668 | 1064707 Up |
| Abcd1 | 0778668 | 0399578 Up |
| Cmpk1 | 0779264 | -050054 Down |
| Fam210b | 0779264 | 031471 Up |
| Araf | 0779264 | 0650059 Up |
| Dph6 | 0779379 | 0341303 Up |
| Celf3 | 0779379 | -065871 Down |
| Pef1 | 0779379 | 0187529 Up |
| Nrg1 | 0779379 | 015267 Up |
| Dlat | 0779379 | 0188363 Up |
| Ppp1r9b | 0779379 | -038031 Down |
| Gca | 0779379 | 0160457 Up |
| Apbb2 | 0779379 | -027452 Down |
| D10Wsu10 | 0779379 | -017008 Down |

| Pip4k2b | 0779379 | -05318 Down |
| --- | --- | --- |
| Gsto1 | 0779379 | -030126 Down |
| Cadm3 | 0779397 | -074602 Down |
| Dcaf15 | 0779397 | -153562 Down |
| Map2k2 | 0779574 | 0281628 Up |
| Prr12 | 0779606 | 0693076 Up |
| Phrf1 | 0779606 | -015822 Down |
| Ccdc167 | 0779606 | 0176185 Up |
| Selm | 0779606 | -061572 Down |
| Mrpl51 | 0780291 | 0203971 Up |
| Rgs20 | 0781433 | 0478519 Up |
| Sec23a | 0781433 | 0266891 Up |
| 4932438A1 | 0781913 | 0153041 Up |
| 0610009B2 | 0781913 | 0205052 Up |
| Glrx5 | 0781913 | 0326633 Up |
| Idh3b | 0782458 | 0181651 Up |
| Dctn2 | 0782937 | -018026 Down |
| BC005624 | 0783403 | -027213 Down |
| Kpnb1 | 0783403 | 0982984 Up |
| Gng12 | 0783403 | -029862 Down |
| Ctdsp2 | 0783403 | 0361704 Up |
| Tigd2 | 0783781 | 0167629 Up |
| Sumo1 | 0783912 | -060824 Down |
| Plat | 078677 | 1371461 Up |
| Cklf | 078677 | 0559852 Up |
| Abt1 | 078677 | 0177342 Up |
| Mettl22 | 078677 | 1368051 Up |
| Dgcr8 | 078677 | 0899853 Up |
| Arih1 | 0786771 | -020643 Down |
| Gldc | 0787274 | 0168502 Up |
| Spag7 | 0787421 | 0567042 Up |
| Daam1 | 078765 | 0866052 Up |
| 1700025G | 0788313 | 0541733 Up |
| Zfp354c | 0788362 | 045874 Up |
| Gnao1 | 0788414 | -039885 Down |
| Pdzph1 | 0788764 | 016442 Up |
| Sart1 | 078916 | 0324556 Up |
| Elavl1 | 078932 | 0347057 Up |
| Atf2 | 0789518 | 0287697 Up |
| Cfap20 | 0790899 | -051446 Down |
| Ralgps2 | 0791082 | 0184016 Up |
| Amfr | 0791235 | -054019 Down |
| Wdr61 | 0791235 | -037994 Down |
| Ireb2 | 0791322 | 0294946 Up |
| Gmnn | 0791322 | 059709 Up |
| Mga | 0792042 | -017276 Down |
| 9130401M | 0792042 | -016943 Down |
| Smap2 | 0792042 | 1532111 Up |
| Myef2 | 0792134 | 0165772 Up |
| Birc5 | 0793214 | -09874 Down |

| Fbxw11 | 0793214 | -060638 Down |
| --- | --- | --- |
| Nudt21 | 0793254 | -029421 Down |
| Zcchc7 | 0793572 | -081608 Down |
| Rrs1 | 0793956 | -019019 Down |
| Hsp90b1 | 0795455 | 0277028 Up |
| Tonsl | 079557 | 0380661 Up |
| Zmat5 | 0795853 | 0284593 Up |
| Ttc19 | 0795853 | -025618 Down |
| Srek1 | 0795853 | -112512 Down |
| Dph5 | 079596 | -063026 Down |
| Vkorc1l1 | 079596 | -020983 Down |
| Ube2g2 | 079596 | -041685 Down |
| Fancl | 079596 | -148919 Down |
| Gltp | 079596 | 0342952 Up |
| Vac14 | 079596 | 0373437 Up |
| Dhrs13 | 079596 | 1580516 Up |
| Trmt1l | 079669 | 0300312 Up |
| Rab33b | 079669 | 1240271 Up |
| Hey2 | 079669 | 1083931 Up |
| Mast4 | 079669 | 0710158 Up |
| Gm17501 | 079669 | 1318264 Up |
| Nosip | 079669 | 0347385 Up |
| Copz2 | 079669 | -039185 Down |
| Nubpl | 079669 | 0429652 Up |
| Gm29508 | 079669 | 0349975 Up |
| Atl2 | 079669 | 0280709 Up |
| Mmgt1 | 079669 | 046724 Up |
| Hdac8 | 079669 | 0316191 Up |
| Lin7c | 079682 | -018326 Down |
| Emc7 | 0797942 | 0402221 Up |
| Yae1d1 | 0797946 | -024894 Down |
| Rprd2 | 0798253 | -019076 Down |
| Hagh | 0798253 | 1526747 Up |
| Rbms1 | 0798253 | 0531947 Up |
| Bag5 | 0798253 | 0286034 Up |
| Cebpg | 0798499 | 1116468 Up |
| Sft2d1 | 0798499 | 0282095 Up |
| Mib1 | 0798499 | 0276713 Up |
| Pld3 | 0798499 | -098122 Down |
| Synj1 | 0798499 | 1295453 Up |
| Trim8 | 0798774 | 0319696 Up |
| Gad2 | 0798881 | 0305201 Up |
| Fbxo9 | 0798881 | -073334 Down |
| Zdhhc17 | 0798881 | 0333589 Up |
| Pmp22 | 0799868 | -033058 Down |
| Herpud1 | 0801157 | -033364 Down |
| Fam168b | 0801303 | 0166537 Up |
| Nmt1 | 0801303 | 0277511 Up |
| Hsf2 | 0802099 | 0364743 Up |
| Cxxc5 | 0802099 | 1536225 Up |

| Eif2b4 | 080282 | 0545454 Up |
| --- | --- | --- |
| Pten | 0803256 | 0332592 Up |
| Bnip2 | 0803256 | -107063 Down |
| Clu | 0803762 | 0282453 Up |
| Ep400 | 0803864 | 034248 Up |
| Nudt9 | 0803864 | 044358 Up |
| Prrx1 | 0803901 | 0162207 Up |
| Pcdh8 | 0803901 | 0849837 Up |
| Crk | 0804127 | 0957747 Up |
| Exosc8 | 080413 | 089029 Up |
| Thrap3 | 08045 | -018555 Down |
| Atp6v1f | 08053 | -015851 Down |
| Gmcl1 | 0805618 | 0415147 Up |
| Dcaf11 | 0805618 | 0476127 Up |
| Nfe2l2 | 0805618 | 0480914 Up |
| Upf1 | 0805618 | -015856 Down |
| Sesn3 | 0805618 | 0182003 Up |
| Txndc17 | 0805677 | 0219907 Up |
| Clcc1 | 0806208 | 0175355 Up |
| Pias2 | 0806208 | -042003 Down |
| Sec62 | 0806399 | 0150893 Up |
| Scai | 080646 | -025842 Down |
| Uckl1 | 080646 | 2159961 Up |
| Arhgap35 | 080646 | 1527574 Up |
| Ttyh2 | 080646 | 0232412 Up |
| Tef | 080646 | 0356349 Up |
| Ogdh | 0807795 | 088951 Up |
| Brd8 | 0808532 | -035943 Down |
| Tmem47 | 0808532 | 0392596 Up |
| Smn1 | 08088 | -172175 Down |
| Pi4ka | 08088 | -042575 Down |
| Cpsf3l | 08088 | -076966 Down |
| Mrps23 | 080905 | 0236932 Up |
| Aifm1 | 080905 | -021124 Down |
| 2410002F2 | 0810326 | -029391 Down |
| Srrm1 | 081055 | 0485668 Up |
| Agpat1 | 0810877 | 0498354 Up |
| Fam65a | 0810877 | 0996652 Up |
| Sptbn1 | 0811453 | 1477413 Up |
| Ilf3 | 0812134 | -025386 Down |
| Vps36 | 0812134 | 0432492 Up |
| Zfand6 | 0812265 | 0678462 Up |
| Clcn3 | 0812265 | -043464 Down |
| Gtf2a2 | 0812265 | 0631336 Up |
| Pold4 | 0812385 | -08763 Down |
| Zbtb37 | 0812633 | 0580835 Up |
| Tmem97 | 0812633 | 0203316 Up |
| Dgkb | 0812633 | -019893 Down |
| Pxdc1 | 0812633 | 0563315 Up |
| Ss18 | 0812633 | -034108 Down |

| Zfp428 | 0813678 | 0374291 Up |
| --- | --- | --- |
| Asb7 | 0814057 | 0242597 Up |
| Gmps | 0814299 | 0726247 Up |
| Parn | 081482 | 0741916 Up |
| Setx | 081482 | -021177 Down |
| Slc33a1 | 081482 | 032659 Up |
| Edc3 | 081482 | 0154968 Up |
| Nprl2 | 081482 | 0286382 Up |
| Daxx | 081482 | -098795 Down |
| 2310061I0 | 081482 | 0431051 Up |
| Ikbkap | 081482 | 0200376 Up |
| Smu1 | 0815323 | 0209398 Up |
| D5Ertd579 | 0815323 | -020444 Down |
| Mfsd2a | 0815492 | 0833749 Up |
| Purg | 0815492 | -035807 Down |
| Nr2f6 | 0815492 | 1654665 Up |
| Pdhx | 0815611 | -026908 Down |
| 0610030E2 | 0815611 | -037818 Down |
| Maoa | 0815611 | -069564 Down |
| Cdh11 | 0815993 | 0665914 Up |
| Tnpo3 | 0816534 | -04773 Down |
| Pcna | 0816534 | 0402021 Up |
| Podxl2 | 0816534 | -040142 Down |
| Med6 | 0816699 | -017845 Down |
| Helz | 0816699 | 0342657 Up |
| Mettl23 | 0817239 | 0165982 Up |
| Sec61b | 081758 | 0359393 Up |
| Eef1d | 081758 | 0553372 Up |
| Cxcr4 | 0818713 | 2385654 Up |
| Sh3bp1 | 0818713 | -043016 Down |
| Impact | 0818713 | 043989 Up |
| Man1b1 | 0818954 | 0564229 Up |
| Slc30a7 | 0818954 | 0412514 Up |
| 3110082J2 | 0818954 | 0659954 Up |
| Cacna1a | 0818954 | 223153 Up |
| Ubald2 | 08192 | 0193844 Up |
| Apip | 0819254 | 1033811 Up |
| Pttg1 | 0819254 | -079064 Down |
| Bola1 | 0819894 | 037804 Up |
| Rnaset2a | 0820907 | 1467708 Up |
| Hes6 | 082221 | 0380348 Up |
| Tceal1 | 082221 | 0209972 Up |
| Phf21a | 0822753 | -052672 Down |
| Jak1 | 0822753 | 1002414 Up |
| Tmem176b | 0822753 | 0626386 Up |
| Acad9 | 0822861 | 0199136 Up |
| Tmem115 | 0822861 | -113176 Down |
| Smarcd1 | 0823035 | 0323363 Up |
| Dlgap1 | 0824447 | -052434 Down |
| Chd3os | 0824462 | -050566 Down |

| Dhdds | 0824462 | 0445364 Up |
| --- | --- | --- |
| Gfod2 | 0824462 | -114771 Down |
| Ncoa7 | 0824462 | -021703 Down |
| Cdk5r1 | 0824462 | -087181 Down |
| Ddx27 | 0824897 | 0353287 Up |
| Fam168a | 0825128 | 050758 Up |
| Hspa4 | 0825129 | -03364 Down |
| Xpo1 | 0825257 | -021967 Down |
| Dlx1 | 0826578 | 0264835 Up |
| Ccdc173 | 0826578 | 0604754 Up |
| Trp53rka | 082705 | 0453702 Up |
| Tm9sf4 | 0827346 | 062062 Up |
| Efnb2 | 0827346 | 0832611 Up |
| Tmem256 | 0827488 | 0394214 Up |
| Fchsd2 | 082894 | 1418008 Up |
| Fcho2 | 082894 | 0375855 Up |
| Whsc1l1 | 0828984 | 0779953 Up |
| Scarb1 | 0829303 | 0308918 Up |
| Prdx1 | 0829319 | -036181 Down |
| Spats2l | 0829391 | -030738 Down |
| Ddx55 | 0829391 | -017646 Down |
| Dync2h1 | 0829391 | -036985 Down |
| Utp11l | 0829584 | -090479 Down |
| Elovl6 | 0829922 | 0552775 Up |
| Tmem259 | 0829922 | 0701066 Up |
| Litaf | 0829922 | -027813 Down |
| Hiat1 | 0829922 | 0540631 Up |
| Timp3 | 0829956 | 0388767 Up |
| Chordc1 | 0830127 | 0681343 Up |
| Pam16 | 0831065 | -064971 Down |
| Myadm | 0831421 | 0997007 Up |
| Sharpin | 0831421 | 0923806 Up |
| Snx12 | 0831421 | -11528 Down |
| Fam63b | 0831421 | 1336616 Up |
| Myh10 | 0832084 | 0366525 Up |
| Lpcat1 | 0832124 | 0210627 Up |
| Tmcc1 | 0832944 | -121139 Down |
| Jmy | 0832944 | -033065 Down |
| Mgat4b | 0832944 | 0181474 Up |
| Ankrd16 | 083305 | 0229688 Up |
| Nup188 | 083305 | 0271907 Up |
| Dnajc11 | 083305 | -023251 Down |
| Hapln3 | 083305 | -027962 Down |
| Zfp868 | 083305 | 0159301 Up |
| Tln2 | 083305 | -033711 Down |
| Rmnd1 | 083305 | 0191309 Up |
| Sgsm2 | 083305 | 0264614 Up |
| Kbtbd7 | 083305 | 0251135 Up |
| Msh6 | 083305 | -035407 Down |
| Pcnxl4 | 083305 | -029058 Down |

| Magohb | 0833589 | 1020545 Up |
| --- | --- | --- |
| Erf | 0833589 | 1049643 Up |
| Llgl1 | 0833589 | -058388 Down |
| Rnf165 | 0833589 | -089195 Down |
| Psma7 | 0834583 | -021149 Down |
| Metrn | 0834775 | 0413457 Up |
| Tmem87b | 0835165 | 0590155 Up |
| Tpcn1 | 0835165 | 0319989 Up |
| Zfp646 | 0835165 | 072075 Up |
| Acvr2b | 0835165 | -125091 Down |
| Tcf20 | 0835165 | 0477512 Up |
| Txnrd2 | 0835165 | -104527 Down |
| Tmem30a | 0835225 | -033338 Down |
| Slc39a7 | 0835225 | 018652 Up |
| Anapc11 | 0835372 | 0194581 Up |
| Ntn1 | 0835683 | 1246013 Up |
| Ccdc136 | 0835683 | 0223963 Up |
| Rbm8a | 0836617 | 0248083 Up |
| Mrpl55 | 0836804 | 0184439 Up |
| Cept1 | 083695 | 0165352 Up |
| Mtus1 | 083695 | 1003721 Up |
| Ptch1 | 083695 | -022454 Down |
| Dynlt1a | 083695 | 1257945 Up |
| Mrpl28 | 0837155 | 0227102 Up |
| Ppme1 | 0837407 | 0423354 Up |
| Grina | 0838172 | -018818 Down |
| 4930453N | 0838172 | 0280173 Up |
| Fam171a2 | 0838172 | -032315 Down |
| Sdf4 | 0838254 | 0187656 Up |
| Taf15 | 0838254 | -016962 Down |
| Fbxo11 | 0838254 | 0424323 Up |
| Sap18 | 0838474 | -029447 Down |
| Snx3 | 0838826 | -032809 Down |
| Ube2v2 | 0838898 | -01501 Down |
| Cbx1 | 0839415 | -023753 Down |
| Nabp2 | 0839586 | 0498931 Up |
| Tox2 | 0840194 | -020384 Down |
| Uba6 | 0840194 | -063957 Down |
| Tcf7l1 | 0840194 | -04797 Down |
| Bcr | 0840194 | -020871 Down |
| Dnajc4 | 0840194 | -015298 Down |
| Itgb1bp1 | 0840442 | 0224918 Up |
| Lipg | 0840827 | -064069 Down |
| Dgcr2 | 0841477 | 0249806 Up |
| Hif1a | 0841936 | -023147 Down |
| Akap13 | 0842514 | -018403 Down |
| Btaf1 | 0843297 | 039768 Up |
| Zfp638 | 0843918 | -073533 Down |
| Acaa2 | 0843918 | 0556422 Up |
| 1810013L2 | 0844613 | 0209773 Up |

| Ube2e1 | 0844788 | 015331 Up |
| --- | --- | --- |
| Ivns1abp | 0844788 | 0228079 Up |
| Taldo1 | 0845516 | 0197205 Up |
| Arl4c | 0845585 | 0782134 Up |
| Scg3 | 0845585 | -02064 Down |
| Vezf1 | 0845585 | 042247 Up |
| Gnb2 | 0846076 | 0205419 Up |
| Dnttip1 | 0846146 | 0238663 Up |
| Slc25a12 | 0846227 | 0623621 Up |
| Kmt2b | 0846227 | -129801 Down |
| Swap70 | 0846227 | 0232353 Up |
| Cenpt | 0846227 | 0187626 Up |
| Zfp426 | 0846227 | 0472482 Up |
| Usp15 | 0846227 | -022754 Down |
| Srp19 | 0846314 | -04807 Down |
| Micu2 | 0846685 | -042714 Down |
| Kat6a | 0846856 | 0181164 Up |
| Pds5b | 0847006 | 0241307 Up |
| Zcchc9 | 0847006 | -033033 Down |
| Pak3 | 0847006 | 1086506 Up |
| Itfg1 | 0847739 | 0236638 Up |
| Terf1 | 0848525 | 0500152 Up |
| Mtf1 | 084894 | 0523612 Up |
| Med11 | 084894 | 0558442 Up |
| Tmem59 | 0850488 | 0206115 Up |
| Cdk4 | 0850746 | 0151245 Up |
| Degs1 | 0850815 | 0279708 Up |
| Eif1ad | 0850815 | -062295 Down |
| Khdrbs2 | 0851371 | 0346555 Up |
| Arfgap1 | 0851371 | 0184582 Up |
| Tbcd | 0851371 | -065468 Down |
| Fam120b | 0851371 | -015826 Down |
| Rlf | 0851446 | 0524747 Up |
| Kpna6 | 0851446 | 0359993 Up |
| Nop14 | 0851446 | 0866149 Up |
| Arhgap33 | 0851446 | 0818932 Up |
| Gm5914 | 0851446 | 0361395 Up |
| Wdr48 | 0851446 | 1919517 Up |
| Hba-a1 | 0851446 | -043454 Down |
| Mtmr4 | 0851446 | 0154263 Up |
| Pik3ca | 0851494 | 032399 Up |
| Mysm1 | 0851494 | 0825639 Up |
| Limch1 | 0851494 | 0639383 Up |
| Psmc1 | 0851777 | 116681 Up |
| Nudt5 | 0852291 | 0273854 Up |
| Jade1 | 0852291 | 017567 Up |
| Akt2 | 0852291 | 0208623 Up |
| Gm14305 | 0853581 | 0194279 Up |
| Med29 | 0853581 | 0523644 Up |
| Rif1 | 0853952 | 0491971 Up |

| Ten1 | 0853952 | -072186 Down |
| --- | --- | --- |
| Mpst | 0853952 | 1065583 Up |
| Ftsj3 | 085513 | 0461337 Up |
| Klhl5 | 085513 | -036316 Down |
| Zfp68 | 085513 | 0359689 Up |
| Snhg18 | 085513 | -032467 Down |
| Las1l | 085513 | 0654351 Up |
| Prkra | 0855487 | -026318 Down |
| Suds3 | 0855487 | 0418952 Up |
| Nptn | 0855487 | -039741 Down |
| Pdzd11 | 0855787 | -057838 Down |
| Fopnl | 0855982 | -027323 Down |
| Src | 085599 | -024467 Down |
| Irgq | 085599 | 0558154 Up |
| Hmgxb4 | 085599 | -031172 Down |
| Opa1 | 085599 | -020774 Down |
| Sin3b | 0856592 | 074437 Up |
| Cct2 | 085683 | 02934 Up |
| Chd8 | 085723 | 0183628 Up |
| Tgif2 | 0857937 | -038818 Down |
| Prdx3 | 0857937 | -030612 Down |
| Rp2 | 0857937 | 1140006 Up |
| Tet3 | 0858826 | 0760999 Up |
| Mmd | 0858826 | -08608 Down |
| Msn | 0858974 | 0515298 Up |
| Maea | 085933 | 0342855 Up |
| Cuedc1 | 085933 | -101998 Down |
| Dtd2 | 085933 | 0260078 Up |
| Shtn1 | 0859982 | 1487669 Up |
| Aspscr1 | 0860291 | 0308205 Up |
| Chmp3 | 0860472 | -031962 Down |
| 2310039H0 | 0860472 | 0374018 Up |
| Irf2bp2 | 0860891 | 0829963 Up |
| Ncan | 0860956 | -017049 Down |
| Chic2 | 0862507 | 0742797 Up |
| Cnot4 | 0862869 | 0396336 Up |
| Casd1 | 0863134 | 0743053 Up |
| Nfrkb | 0863134 | 0184292 Up |
| Cactin | 0863134 | 0172555 Up |
| Nfx1 | 0863515 | 1186957 Up |
| Cd2ap | 0863515 | 0418097 Up |
| Ncbp1 | 0864858 | -025255 Down |
| Frmd4b | 0864858 | -026837 Down |
| Borcs8 | 0864858 | 0686815 Up |
| Trappc6a | 086497 | -03517 Down |
| Nap1l4 | 0865045 | -018278 Down |
| Maz | 0866002 | 029604 Up |
| Zcchc18 | 0866002 | -030287 Down |
| Prpf40b | 0866534 | 0744418 Up |
| Fam109a | 0866534 | -035821 Down |

| Gadd45gip | 086658 | 0816978 Up |
| --- | --- | --- |
| Hbb-bs | 0866662 | -049174 Down |
| Sp3 | 0866761 | 0239656 Up |
| Nsdhl | 0866761 | 042358 Up |
| Coro1c | 0867687 | -061387 Down |
| Eif4enif1 | 0867687 | -015151 Down |
| Frat2 | 0867687 | -020073 Down |
| Fktn | 086779 | 0300433 Up |
| Rdh11 | 086779 | 0289669 Up |
| Exoc2 | 086779 | 0346769 Up |
| Cdh10 | 086779 | -050581 Down |
| Efr3a | 086779 | -024634 Down |
| Josd1 | 086779 | 0867805 Up |
| RP23-291B | 086779 | 0892943 Up |
| Cacybp | 0868175 | 0329516 Up |
| Hepacam | 0868499 | -024052 Down |
| Tcerg1 | 0868499 | -027135 Down |
| Sltm | 0869045 | -060558 Down |
| Dazap2 | 0869107 | 0319727 Up |
| Otud1 | 0869479 | 039675 Up |
| Sp3os | 0869568 | -036889 Down |
| 2810403A0 | 0869568 | 0643443 Up |
| Nwd1 | 0869772 | -036938 Down |
| Kcnk1 | 0869772 | 0534736 Up |
| Kcnmb4 | 0869772 | -03729 Down |
| 2510009E0 | 0869772 | -124988 Down |
| Upf2 | 0871543 | -030515 Down |
| Itpa | 0871543 | -071174 Down |
| Camk1 | 0871543 | 0641167 Up |
| Mtif2 | 0871543 | -032909 Down |
| Bex4 | 0871543 | 0363808 Up |
| Iars2 | 0871844 | 0583123 Up |
| Trim32 | 0871844 | 0212235 Up |
| Eed | 0871844 | -113218 Down |
| 2300009A0 | 0871844 | 1952117 Up |
| Ppp2r5d | 0871844 | -019286 Down |
| Tra2a | 0872439 | 0245375 Up |
| Ube2h | 0873365 | 0274641 Up |
| Mrpl4 | 0873365 | 162244 Up |
| Ube2d3 | 0873497 | 0204774 Up |
| Tpx2 | 0874006 | -041951 Down |
| Wasf1 | 0874006 | 0388918 Up |
| Pmepa1 | 0874103 | -072947 Down |
| Cbl | 0874103 | -017593 Down |
| Cox17 | 0874103 | -084748 Down |
| Galnt1 | 0874103 | -065408 Down |
| Erp29 | 087423 | 0256765 Up |
| Vhl | 0874302 | 0193393 Up |
| Tubg1 | 0874302 | 0193541 Up |
| 1500009C0 | 0874302 | 0232479 Up |

| Cbs | 0874302 | 018778 Up |
| --- | --- | --- |
| Atp5c1 | 0874488 | 0168012 Up |
| Rpl39 | 0874574 | 0359141 Up |
| Mid1ip1 | 08756 | 0205207 Up |
| Myo10 | 0876262 | 0282826 Up |
| Psmg2 | 0876376 | -018164 Down |
| Gltscr2 | 0876635 | 0744002 Up |
| Supt16 | 0878191 | -030168 Down |
| Ublcp1 | 0878261 | -057891 Down |
| N6amt2 | 0878261 | -020826 Down |
| Lrrc42 | 0878261 | 0811018 Up |
| Syap1 | 0878261 | -04272 Down |
| Hps4 | 0878555 | -070374 Down |
| 4931406P1 | 0878555 | 0335355 Up |
| Rapgef6 | 0878555 | -048711 Down |
| Myh9 | 0878555 | 1032577 Up |
| Gpm6b | 0878756 | -016719 Down |
| Cnot3 | 0879978 | 0316328 Up |
| Fbxo2 | 0880103 | -04681 Down |
| Ppp1r18 | 0880103 | -063176 Down |
| Evi5l | 0880103 | 0252644 Up |
| Homer3 | 0880103 | 1101027 Up |
| Cdk19 | 0880103 | 0869749 Up |
| Slc30a5 | 0880103 | 0197047 Up |
| Msi1 | 0880295 | 0157448 Up |
| Vps13d | 0881361 | -023162 Down |
| Adipor1 | 0881546 | -031498 Down |
| Csk | 0881546 | -08114 Down |
| Tmem205 | 0881546 | 0537666 Up |
| Tmem63b | 0881546 | -065926 Down |
| Phf3 | 0881995 | 0241099 Up |
| Ndn | 0882465 | 0492666 Up |
| Rab28 | 088298 | 0276129 Up |
| Slc4a4 | 088298 | 0239922 Up |
| Vps45 | 0883134 | 0191623 Up |
| Tekt1 | 0883134 | 0159844 Up |
| Farsb | 0883872 | 0309918 Up |
| Ddx54 | 0883872 | -033916 Down |
| Cmas | 0883872 | -031213 Down |
| Cpeb4 | 0883872 | 0388757 Up |
| Taf6l | 0883872 | 0415266 Up |
| Ipo8 | 0884192 | 0276701 Up |
| Cbr4 | 0884192 | 0342431 Up |
| Alkbh8 | 0884192 | 2065457 Up |
| Per1 | 0884192 | -101382 Down |
| Sfrp2 | 0884194 | 0645982 Up |
| Igsf3 | 0884194 | -046329 Down |
| Kti12 | 0884194 | 1112112 Up |
| Amt | 0884194 | 0695358 Up |
| Nme6 | 0884194 | 0507154 Up |

| Gopc | 0884194 | 0816949 Up |
| --- | --- | --- |
| Zbtb7a | 0884194 | 0830455 Up |
| Scn8a | 0884194 | 0687046 Up |
| Adprh | 0884293 | -017776 Down |
| Eif3j1 | 0884898 | 0253588 Up |
| Arglu1 | 0885088 | 0214566 Up |
| Capza2 | 0885373 | -019283 Down |
| Atp5g3 | 0885486 | 0339949 Up |
| Flrt2 | 0886131 | 0372826 Up |
| Sepp1 | 08866 | 0573356 Up |
| Atp6v0d1 | 0887618 | 0269148 Up |
| Rrp15 | 088775 | -061951 Down |
| Pgrmc2 | 088775 | -030195 Down |
| Bsdc1 | 088775 | -021467 Down |
| Dhrs7b | 088775 | 053055 Up |
| Kif3c | 088775 | 0321841 Up |
| Fam98a | 088775 | -123756 Down |
| B4galt6 | 088775 | 1429799 Up |
| Rabgap1l | 0888243 | -035101 Down |
| Aen | 0888243 | -015393 Down |
| Nedd9 | 0888243 | -057569 Down |
| Gtf2e1 | 0888243 | -019445 Down |
| Amn1 | 088855 | 1260898 Up |
| Usp16 | 0888726 | 0777263 Up |
| Phyhipl | 0889438 | -018094 Down |
| Nme1 | 0890226 | -049944 Down |
| Trim24 | 0891699 | -028896 Down |
| Ntrk2 | 0891699 | -047771 Down |
| 1110038F1 | 0891699 | 0879788 Up |
| Ap2a2 | 0891819 | -032254 Down |
| Eif2s3x | 0891819 | 0408949 Up |
| Bhlhb9 | 0891819 | 0254192 Up |
| Rab6a | 0892189 | 0371178 Up |
| Wnk1 | 0892746 | -049996 Down |
| Ash1l | 0893721 | -057578 Down |
| Rbm6 | 0893823 | 0227339 Up |
| Pspc1 | 0893823 | -016276 Down |
| Ttc14 | 0894146 | -023452 Down |
| Snrnp70 | 0894532 | -036482 Down |
| Txndc12 | 0894626 | 1466199 Up |
| Elovl5 | 0894626 | 0165758 Up |
| Fhl1 | 0894626 | 043041 Up |
| Vma21 | 0894626 | 1420616 Up |
| Phf5a | 0895484 | 0356632 Up |
| Ddx21 | 0895904 | 0462582 Up |
| Cep164 | 0896101 | -03974 Down |
| Ube2r2 | 0896173 | 0497715 Up |
| Ube2b | 0896511 | 0517505 Up |
| Mrpl52 | 0896511 | 0626004 Up |
| Babam1 | 0896854 | -093774 Down |

| Akap8 | 0896854 | 0895736 Up |
| --- | --- | --- |
| Hspa5 | 0896955 | 033947 Up |
| 2410004B1 | 0897074 | 043057 Up |
| Gm2a | 0897074 | 0942537 Up |
| H13 | 0897074 | -080724 Down |
| Tox | 0898304 | 0421643 Up |
| Scmh1 | 0898626 | -033768 Down |
| Ppp1r12a | 0899153 | -033372 Down |
| Ddx1 | 0899153 | 0829645 Up |
| Lmbrd1 | 0899866 | 0331971 Up |
| Kif2a | 0899995 | 0637447 Up |
| Tnfaip1 | 0900035 | 0854235 Up |
| Acp2 | 0900285 | -015827 Down |
| Keap1 | 0900285 | 0694012 Up |
| Mmp16 | 0900643 | 0172419 Up |
| Prc1 | 0900643 | -089964 Down |
| Ascc1 | 0900643 | 0676641 Up |
| Fam13c | 0900643 | 0331544 Up |
| Ubtd2 | 0900643 | 0495612 Up |
| Lrrn2 | 0901912 | 0942509 Up |
| Rnf114 | 0901912 | -18911 Down |
| Ppm1l | 0901912 | 0980311 Up |
| Prr14 | 0901912 | 0308525 Up |
| Ilvbl | 0901912 | 0470075 Up |
| Hacl1 | 0901912 | 0613206 Up |
| Camk2n2 | 0901912 | -053307 Down |
| Tmem138 | 0902074 | -03433 Down |
| Rhoc | 090233 | -038736 Down |
| Arf1 | 090236 | 0466959 Up |
| Ppp1r37 | 0903484 | 0910644 Up |
| Bmp2k | 0903701 | 0966135 Up |
| Cby1 | 0904106 | -015038 Down |
| Tdrkh | 0904481 | 1086482 Up |
| Lrrc59 | 0904481 | 020095 Up |
| Phax | 0904603 | 0306407 Up |
| Nav1 | 0905057 | -01871 Down |
| Rpn2 | 0905057 | 0628748 Up |
| Mt1 | 0905471 | 0470793 Up |
| 2-Sep | 0906193 | 0203407 Up |
| Nfix | 0906505 | 0208839 Up |
| Cat | 090651 | 1231385 Up |
| Krcc1 | 090651 | -017984 Down |
| Ppdpf | 0906787 | 0321874 Up |
| Minos1 | 0907473 | 0321073 Up |
| Pex2 | 0907735 | 0165358 Up |
| Dctn1 | 0907735 | -02321 Down |
| Prmt2 | 0907735 | 0326953 Up |
| Rpain | 0907735 | 0285877 Up |
| Acot13 | 0907735 | 0193848 Up |
| Rbm42 | 0907749 | -053422 Down |

| Sall1 | 0908184 | 0157675 Up |
| --- | --- | --- |
| Ubl5 | 0908415 | 0232274 Up |
| Hspa8 | 0908604 | -016778 Down |
| Zfp36 | 0908762 | 0454281 Up |
| Zfp560 | 0908762 | 0282614 Up |
| Acyp2 | 0908762 | 0511967 Up |
| Map2k4 | 0908762 | 0268266 Up |
| Xpo7 | 0908762 | 0294665 Up |
| Dynlt3 | 0908762 | 0282534 Up |
| Wash1 | 0908762 | 0255625 Up |
| Sec14l1 | 0909881 | 0198645 Up |
| Actn1 | 0909881 | 0201766 Up |
| Mrpl50 | 0910544 | 0215496 Up |
| Ldb1 | 0910544 | -107972 Down |
| Rbm34 | 0910876 | -027182 Down |
| Bcan | 0912769 | 0231498 Up |
| Anp32b | 0912827 | 0463951 Up |
| B230219D | 0913205 | 0441611 Up |
| Polr3h | 0913205 | 0273547 Up |
| Tax1bp1 | 0913553 | -017315 Down |
| Anapc16 | 0913874 | 021962 Up |
| Faap100 | 0914156 | -106493 Down |
| Gpr137b | 0914156 | -030662 Down |
| Snhg12 | 0914201 | 2064019 Up |
| Galnt7 | 0914201 | 0427096 Up |
| Ubxn2a | 0914201 | 0200981 Up |
| Skil | 0914471 | 0256668 Up |
| Klf2 | 0914471 | -05386 Down |
| Dtx4 | 0914471 | 0412466 Up |
| Setd1b | 091534 | 0249889 Up |
| Fam49b | 091557 | 0440701 Up |
| Rnf167 | 0916008 | 0392412 Up |
| Arhgef40 | 0916008 | 0199742 Up |
| Kif3a | 091648 | 0226776 Up |
| Phkg2 | 0917137 | -055585 Down |
| Prpsap2 | 0917137 | -018678 Down |
| Haus1 | 0917137 | -052879 Down |
| Dock11 | 0917137 | 0272418 Up |
| Itch | 0917167 | -06694 Down |
| Tpd52l2 | 0917167 | 0233438 Up |
| Lamtor3 | 0917167 | -083533 Down |
| Stag1 | 0917242 | 0459542 Up |
| Sash1 | 09174 | 18713 Up |
| Cyb5a | 0917874 | -018086 Down |
| Cr1l | 0918276 | 0552313 Up |
| Acaca | 0918276 | 0404666 Up |
| Ubl4a | 0918276 | 0371946 Up |
| Dennd1a | 0918665 | -037445 Down |
| Ankrd11 | 0919296 | 0358975 Up |
| Usmg5 | 091962 | 0186845 Up |

| Slc35f5 | 0919679 | 053133 Up |
| --- | --- | --- |
| Rgs2 | 0919679 | -084598 Down |
| Fam110a | 0919679 | -058562 Down |
| Zfp932 | 0919679 | -04003 Down |
| Med24 | 0919679 | 0349528 Up |
| Slc41a1 | 0919691 | 0749668 Up |
| Trp53bp2 | 0919691 | 1265006 Up |
| Polr3c | 0919691 | 0251195 Up |
| Parg | 0919691 | -020286 Down |
| Tbc1d4 | 0919691 | 020046 Up |
| Cpt1a | 0919691 | 0664116 Up |
| Calm3 | 0919699 | 0351229 Up |
| Vcp | 0919766 | 0259026 Up |
| Ociad1 | 0919817 | -045566 Down |
| Scamp2 | 0919817 | 0598927 Up |
| Nt5c | 0919817 | -03425 Down |
| Rpa3 | 0920004 | 025084 Up |
| Ptpn12 | 0920869 | 0365461 Up |
| Gbas | 0920869 | 0328992 Up |
| Pkn2 | 092183 | 0160786 Up |
| Gsk3a | 092183 | 1240742 Up |
| Ebpl | 092183 | 1135485 Up |
| Atp13a2 | 0921934 | 0483676 Up |
| Diablo | 0922022 | -016194 Down |
| Dtl | 0923917 | -051052 Down |
| Exoc1 | 0923917 | 0435583 Up |
| Ddhd2 | 0923917 | 0428815 Up |
| Clk3 | 0923917 | -017318 Down |
| Agpat5 | 092426 | -038795 Down |
| Psmd11 | 0924413 | -03592 Down |
| Dars | 092494 | 0595854 Up |
| Panx1 | 0925359 | -077935 Down |
| Mbip | 0925359 | -041215 Down |
| Ppp1r1a | 0925565 | -021123 Down |
| Polr2a | 0926667 | -031196 Down |
| Apex1 | 0926667 | 1138264 Up |
| Zc2hc1a | 0927263 | 0937466 Up |
| Zcchc8 | 0927263 | -018486 Down |
| Tmem219 | 0927263 | 0522799 Up |
| Nucks1 | 0927596 | 0525134 Up |
| Snf8 | 0928004 | 0571789 Up |
| Zfand5 | 0928377 | -021175 Down |
| Rnpep | 0928684 | 0735115 Up |
| Mfap1b | 0928684 | 0365298 Up |
| Ncor2 | 0928684 | 0377889 Up |
| Rnf126 | 0928684 | -082744 Down |
| Stxbp4 | 0928684 | 0771968 Up |
| Lsm8 | 0929574 | 0409622 Up |
| Aplp2 | 0929574 | -022821 Down |
| Wdr34 | 0929719 | -048166 Down |

| Amer2 | 0929719 | -040271 Down |
| --- | --- | --- |
| Gm10600 | 0929719 | -031199 Down |
| Gna12 | 0929967 | 0333107 Up |
| Rwdd4a | 0929967 | 054163 Up |
| Rab10 | 0930794 | -019691 Down |
| Tank | 0931129 | 1009154 Up |
| Pigu | 0931313 | 0373266 Up |
| Axl | 0931313 | 0593197 Up |
| Sdhaf2 | 0931313 | 0682783 Up |
| Zfp703 | 0932185 | -044275 Down |
| Tsfm | 0932185 | 1254132 Up |
| Zmym5 | 0932185 | 0542718 Up |
| Rhob | 0932279 | -044665 Down |
| Sec61a1 | 0933096 | -021671 Down |
| Acadsb | 0933096 | 0633903 Up |
| Clpx | 0933146 | -100108 Down |
| Eif4g1 | 0933207 | -015688 Down |
| Ubqln4 | 0933666 | -040936 Down |
| Aptx | 0933666 | 050942 Up |
| Pygo1 | 0933666 | -03008 Down |
| Sfswap | 0934024 | 0198832 Up |
| Ppm1b | 0934024 | -046719 Down |
| Tubb3 | 0934645 | -07387 Down |
| Bcl10 | 0935562 | 0855704 Up |
| Rab35 | 0935562 | 0385172 Up |
| Mettl5 | 0935714 | 0574721 Up |
| Dmap1 | 0935714 | 1094597 Up |
| Rybp | 0935714 | 0437463 Up |
| Armc8 | 0935714 | 0502587 Up |
| Caskin2 | 0935714 | 0168902 Up |
| Fem1c | 0935714 | 080853 Up |
| Rbfox2 | 0936236 | -027371 Down |
| Cdc42se1 | 0936854 | 0375695 Up |
| Tmbim1 | 0937486 | -078603 Down |
| Ackr3 | 0937486 | -017507 Down |
| Tfb2m | 0937486 | -026242 Down |
| Gtdc1 | 0937486 | 0414151 Up |
| Nsmaf | 0937486 | 0801553 Up |
| Alpl | 0937486 | 0584609 Up |
| Rpap2 | 0937486 | -089157 Down |
| Deaf1 | 0937486 | -038085 Down |
| Hivep1 | 0937486 | -051803 Down |
| Slain1 | 0937486 | 0349538 Up |
| Ankrd54 | 0937486 | 0775438 Up |
| Ano6 | 0937486 | -030072 Down |
| Snx15 | 0937486 | -048524 Down |
| Tle4 | 0937486 | -028443 Down |
| Rftn2 | 0938001 | 0274901 Up |
| Zyg11b | 0938001 | 1120379 Up |
| Abhd8 | 0938001 | 1262375 Up |

| Ext1 | 0938001 | 0227956 Up |
| --- | --- | --- |
| Elavl3 | 0938863 | -02923 Down |
| Azin1 | 0938863 | 0239455 Up |
| 0610007P1 | 0938863 | 036737 Up |
| Mfsd10 | 0939101 | -017306 Down |
| Med17 | 0939101 | 0528698 Up |
| Psme3 | 0939101 | -01613 Down |
| Rap1a | 0940835 | -032389 Down |
| Bmpr2 | 0941072 | 0246399 Up |
| Sympk | 0941781 | 0670226 Up |
| Zfp260 | 0941781 | 1353678 Up |
| Gnpat | 0941781 | 1474163 Up |
| Tm7sf3 | 0941781 | -023462 Down |
| Yeats2 | 0941781 | -028689 Down |
| Abhd10 | 0941781 | -028127 Down |
| Coro1b | 0941781 | -061116 Down |
| Banf1 | 0941964 | 0262719 Up |
| Adh5 | 0942205 | -025167 Down |
| Cct3 | 0942394 | -018982 Down |
| Gng5 | 0942715 | -023473 Down |
| Abhd2 | 094292 | 0291922 Up |
| Tshz1 | 094292 | 036364 Up |
| Chuk | 0943948 | 1825144 Up |
| Hmbox1 | 0943948 | -025575 Down |
| Rpap3 | 0943948 | -040437 Down |
| Slc29a3 | 0943984 | -046894 Down |
| Akap7 | 0945309 | 0194079 Up |
| Lpar4 | 0945309 | -025678 Down |
| Chn1 | 0945724 | 1162258 Up |
| Ppp1r35 | 0946494 | 0347389 Up |
| Ppp1r10 | 0947198 | 0802762 Up |
| 4930402H2 | 0947198 | 0238336 Up |
| Rit1 | 0947198 | -063974 Down |
| Npas3 | 0947198 | 0388332 Up |
| Srpk1 | 0947198 | -024392 Down |
| Tmem258 | 0947629 | -015151 Down |
| Cntnap2 | 094794 | 018934 Up |
| Acadvl | 094794 | 1039805 Up |
| Exosc1 | 094794 | 2309492 Up |
| A230046K0 | 094794 | 0182477 Up |
| Fam107b | 0948457 | 0543511 Up |
| Gtf2h3 | 0950225 | -049416 Down |
| Ahi1 | 095072 | 0244759 Up |
| Myo18a | 095072 | 0944902 Up |
| Mrpl13 | 095072 | 0461671 Up |
| Ddx18 | 0951397 | -055527 Down |
| Uri1 | 095176 | 0435339 Up |
| Pex11b | 0951763 | 0413982 Up |
| Fam53b | 0951763 | 055836 Up |
| 0610009L1 | 0951763 | 0164463 Up |

| Naa30 | 0951763 | 0451725 Up |
| --- | --- | --- |
| Lpp | 0951763 | 0628965 Up |
| Poglut1 | 0951763 | 0365084 Up |
| Uxt | 0951763 | 0364014 Up |
| Ahsa1 | 0951966 | 0187557 Up |
| Rab5a | 0952216 | -03772 Down |
| Sdhd | 0952652 | 0841988 Up |
| Sparc | 0953172 | -028853 Down |
| Cfdp1 | 0953556 | -026233 Down |
| 1110008F1 | 0953556 | -051239 Down |
| Coprs | 0954306 | 0539863 Up |
| Ttc8 | 0954306 | 0408155 Up |
| Cnpy3 | 0954306 | 0415981 Up |
| Fra10ac1 | 0954306 | -036056 Down |
| Nmi | 0955325 | -109119 Down |
| Ncoa5 | 0955325 | 1864334 Up |
| Foxo1 | 0955325 | 0161749 Up |
| Foxp2 | 0955325 | 0420428 Up |
| Nav2 | 0955325 | -019992 Down |
| Chid1 | 0955325 | -049641 Down |
| Enkd1 | 0955325 | -023762 Down |
| Nmral1 | 0955325 | 0414626 Up |
| Vps52 | 0955325 | -03635 Down |
| Vmac | 0955325 | 1578276 Up |
| Ypel5 | 0955325 | 0783658 Up |
| Smim20 | 0955368 | -031574 Down |
| 2610002M | 0956319 | -099136 Down |
| Prom1 | 0957174 | 0449609 Up |
| Larp1 | 0957174 | -063339 Down |
| Tacc1 | 0957466 | 0175636 Up |
| Cdc42se2 | 0957466 | 0284661 Up |
| Rnaseh2a | 0957945 | 0303363 Up |
| Mob4 | 0959256 | 0661047 Up |
| Romo1 | 0959256 | 0197192 Up |
| Sypl | 0959279 | -015189 Down |
| Ctnnb1 | 0959446 | 0420043 Up |
| Gtf3a | 0959858 | -059853 Down |
| Rtn1 | 095986 | -015366 Down |
| Cdk9 | 0960386 | 0198521 Up |
| Emsy | 0960868 | 0253917 Up |
| Cacna2d1 | 096092 | -03154 Down |
| Zpr1 | 096092 | -026077 Down |
| Tpm1 | 096131 | 0237055 Up |
| Arcn1 | 096131 | 0161043 Up |
| Uqcc3 | 096131 | 0694086 Up |
| Ddrgk1 | 0962087 | 0356735 Up |
| Zfp871 | 0962087 | 0210125 Up |
| Tmem208 | 0962428 | 0785159 Up |
| Nelfe | 0962428 | 0342349 Up |
| Tns3 | 0962742 | 0300823 Up |

| Insm1 | 0962801 | 0625058 Up |
| --- | --- | --- |
| Plekhf2 | 0962801 | 0387019 Up |
| Ppp2r4 | 0962801 | 1610236 Up |
| Fam60a | 0962801 | 1081751 Up |
| Hectd1 | 096303 | 0211781 Up |
| Sssca1 | 096303 | -1088 Down |
| Fam171b | 0963538 | -015218 Down |
| Camta1 | 0963761 | 0209476 Up |
| Ctsb | 0963761 | -069955 Down |
| Taf7 | 0963965 | 0555401 Up |
| Ergic3 | 0964152 | 0539294 Up |
| Ppp1r2 | 096448 | 0592457 Up |
| 6330403K0 | 0964623 | 0739797 Up |
| Tspan3 | 0965235 | -018486 Down |
| Exosc3 | 0965532 | -022304 Down |
| Fam192a | 0965532 | 0164543 Up |
| Ppan | 0965625 | 0441745 Up |
| Tdrd7 | 0966804 | -085011 Down |
| Paxip1 | 0966804 | -150783 Down |
| Vamp8 | 0966804 | -097914 Down |
| Cdk12 | 0967386 | -017583 Down |
| Mcm3 | 0967831 | 0499617 Up |
| Stx2 | 0967831 | 0535731 Up |
| Tpst1 | 0967831 | 0313055 Up |
| Agk | 0967831 | 05438 Up |
| Zfp579 | 0967831 | 023721 Up |
| Prkx | 0967831 | 0689387 Up |
| Isca2 | 0968092 | 0343555 Up |
| Tnpo2 | 0968732 | 2092342 Up |
| Smg5 | 0969528 | 0376982 Up |
| Srm | 0969528 | 0230103 Up |
| Bcl2l12 | 0969528 | 0342896 Up |
| Chmp2b | 0969528 | 1807243 Up |
| Pdpk1 | 0969528 | 0285975 Up |
| Med21 | 0969611 | -035386 Down |
| Gnai3 | 0970267 | -100727 Down |
| Papola | 0970722 | -017252 Down |
| Spns1 | 0971442 | 0152315 Up |
| Irs2 | 0971442 | 0254304 Up |
| Ifrd1 | 0971442 | 0546676 Up |
| AI314180 | 0972423 | 1618054 Up |
| Anapc2 | 0972423 | -020819 Down |
| Nmt2 | 0972846 | 0312026 Up |
| Tlk1 | 0972846 | 0432943 Up |
| Atxn1 | 0972846 | 0570666 Up |
| Bre | 0972846 | 0648541 Up |
| Gtf3c3 | 0973186 | 0873763 Up |
| Chp1 | 0973186 | 1755232 Up |
| Fndc3b | 0973186 | 0450196 Up |
| Ankrd50 | 0973186 | 016088 Up |

| Yars | 0973186 | 1815534 Up |
| --- | --- | --- |
| Repin1 | 0973186 | 1048565 Up |
| Ccp110 | 0973186 | 0285621 Up |
| Stard3 | 0973186 | -018483 Down |
| Dubr | 0973186 | 0224786 Up |
| Me2 | 0973186 | -14215 Down |
| BC023829 | 0973186 | 0290133 Up |
| Suv420h2 | 0973186 | -026986 Down |
| Prnp | 0973584 | -041143 Down |
| Galk1 | 0974201 | -021019 Down |
| Cdk2ap2 | 0974201 | -065661 Down |
| Mcrs1 | 0974719 | 0300437 Up |
| Ywhag | 0974719 | -021465 Down |
| Slc6a1 | 0974719 | -031047 Down |
| S100a1 | 0975156 | 0313899 Up |
| Thra | 0975156 | 0672136 Up |
| Smim7 | 0975348 | 1052182 Up |
| Glrx2 | 0976497 | -017216 Down |
| Vps29 | 0976497 | -022576 Down |
| Zscan21 | 0976546 | -047667 Down |
| Cops6 | 0976576 | 0337292 Up |
| Psmb6 | 097704 | 0362542 Up |
| Iqce | 0977675 | 0474682 Up |
| Msrb1 | 0977675 | 035669 Up |
| Fahd1 | 0977675 | 0650731 Up |
| Pdzd8 | 0978579 | -060259 Down |
| Stam2 | 0979315 | 0658384 Up |
| Dnpep | 0980852 | 1111813 Up |
| Nckap5 | 0980852 | 0222258 Up |
| Osgep | 098151 | 0610967 Up |
| Plgrkt | 0981764 | 0773823 Up |
| Prpf40a | 0982431 | 0214126 Up |
| Tma16 | 0982808 | 0891229 Up |
| Apeh | 0982808 | 0714734 Up |
| Ints9 | 0982808 | 1164901 Up |
| Tmem176a | 0983399 | -048789 Down |
| Paip2b | 0983399 | -05703 Down |
| Zbtb21 | 0983399 | -148 Down |
| Srf | 0983399 | -044394 Down |
| Lancl2 | 0983912 | -090309 Down |
| Mvd | 0983912 | 0493871 Up |
| Man2c1 | 0983912 | 0924029 Up |
| Mlx | 0983912 | -030073 Down |
| Gnl1 | 0983912 | 0629834 Up |
| Snap23 | 0984761 | -115181 Down |
| Adipor2 | 0984761 | -066813 Down |
| Pym1 | 0984761 | 050878 Up |
| Zmiz2 | 0984761 | 0567906 Up |
| Il11ra1 | 0985433 | 0833773 Up |
| Psmd5 | 0985433 | -080442 Down |

| Ormdl3 | 0985433 | -056229 Down |
| --- | --- | --- |
| Pop7 | 0985719 | 0347163 Up |
| M6pr | 0985719 | 0237558 Up |
| Ubfd1 | 0985719 | 1480926 Up |
| Ap4s1 | 0985719 | 0379351 Up |
| Rragd | 0985976 | 0220085 Up |
| Rab31 | 0985976 | 0633359 Up |
| Ammecr1l | 0985976 | 0947034 Up |
| Fam167a | 0985976 | 0196897 Up |
| Dnajb14 | 0986421 | -081605 Down |
| Mapk8ip3 | 0986421 | 0506358 Up |
| Vamp4 | 0986614 | -018406 Down |
| Pogz | 098679 | -048707 Down |
| Eci1 | 098679 | 0161989 Up |
| Slc39a6 | 0986951 | 0217564 Up |
| 2210013O | 0986951 | 1314753 Up |
| Ccdc115 | 0987099 | 035888 Up |
| Mapk6 | 0987099 | -062698 Down |
| Dnlz | 0987358 | 0313849 Up |
| Acsl3 | 0987472 | 0420501 Up |
| Fam213a | 0987472 | 0410236 Up |
| Mex3a | 0987761 | 0398217 Up |
| Atad1 | 0987761 | -031467 Down |
| Mtpn | 0987917 | 0389433 Up |
| Npdc1 | 0987985 | 0354298 Up |
| Dnajc3 | 0987985 | -083127 Down |
| Emc10 | 0988322 | 0206493 Up |
| Smc3 | 0988322 | 0273688 Up |
| Srsf11 | 0988409 | 0218276 Up |
| Atp6v0e | 0988431 | 0286504 Up |
| Aldoa | 0988451 | 0257572 Up |
| Apoe | 0988452 | -032187 Down |
| Ssr2 | 0988452 | -017499 Down |
| Ywhaz | 0988519 | 0469643 Up |
| Ncoa3 | 0991061 | 0202659 Up |
| Ndufaf6 | 0991061 | 0171431 Up |
| Atp11a | 0991061 | 0791323 Up |
| Gsr | 0991061 | -057291 Down |
| Stoml1 | 0991061 | 0403303 Up |
| Pde4d | 0991061 | 0211205 Up |
| Scaf8 | 0991061 | 0235302 Up |
| Rrp1b | 0991061 | 0175628 Up |
| Mkl2 | 0991061 | -047637 Down |
| Cntln | 0992181 | -053967 Down |
| Trpc1 | 0992181 | -029994 Down |
| Nsun2 | 0992558 | 0437638 Up |
| Phkb | 0993104 | -068956 Down |
| 0610037L1 | 0993475 | -015169 Down |
| Nrd1 | 0993617 | 0596899 Up |
| Slmo2 | 0993736 | -035379 Down |

| Polr2e | 0993992 | 0312572 Up |
| --- | --- | --- |
| Psmb4 | 0994242 | -019268 Down |
| Fam133b | 1 | -058335 Down |
| Mest | 1 | 0547374 Up |
| Trappc1 | 1 | 0477395 Up |
| Tmem167 | 1 | -041629 Down |
| Cd9 | 1 | -037313 Down |
| Fis1 | 1 | 0244248 Up |
| Slc1a3 | 1 | -015168 Down |
| Rbp1 | 1 | 0563628 Up |
| Snrpc | 1 | -049941 Down |
| Pdcd10 | 1 | 0535163 Up |
| Gipc1 | 1 | -025624 Down |
| Emc2 | 1 | 0299176 Up |
| Taf1d | 1 | -015751 Down |
| Nubp2 | 1 | 0181863 Up |
| Atad2b | 1 | 0750931 Up |
| Atxn2l | 1 | -016398 Down |
| Sox5 | 1 | -017976 Down |
| Cib1 | 1 | -081867 Down |
| Smad7 | 1 | -049616 Down |
| Dnajb11 | 1 | 0330701 Up |
| Naa10 | 1 | 0215509 Up |
| Zfp280d | 1 | 0336507 Up |
| Ssbp2 | 1 | 0333595 Up |
| Gpcpd1 | 1 | -035195 Down |
| Lzic | 1 | 0281911 Up |
| Eef2kmt | 1 | 0939663 Up |
| Ift52 | 1 | 054734 Up |
| Alg8 | 1 | 0359334 Up |
| Usp32 | 1 | 0165889 Up |
| Tsr3 | 1 | 0576406 Up |
| Stk19 | 1 | 0190086 Up |
| Map3k4 | 1 | -01737 Down |
| Pcgf5 | 1 | -04973 Down |
| Cep290 | 1 | 1062565 Up |
| Kcnj10 | 1 | -015718 Down |
| Elp5 | 1 | -018975 Down |
| Dcaf7 | 1 | -028831 Down |
| Rfc1 | 1 | -033443 Down |
| Bcap31 | 1 | -053066 Down |
| Commd9 | 1 | -026464 Down |
| Cyth3 | 1 | 016575 Up |
| 8-Sep | 1 | 0429228 Up |
| Pgam5 | 1 | 0181042 Up |
| Arhgdib | 1 | 0298152 Up |
| Arhgef17 | 1 | -062203 Down |
| Cmtm3 | 1 | 0244808 Up |
| Pld2 | 1 | 0359376 Up |
| Abcd4 | 1 | 0282619 Up |

| Cplx2 | 1 | 0292512 Up |
| --- | --- | --- |
| Ppp1r16a | 1 | 0211317 Up |
| Wdr53 | 1 | 0641255 Up |
| Gpatch11 | 1 | 0265211 Up |
| Tfe3 | 1 | 0371631 Up |

Supplemental table 4: Microglia CKO vs WT genes expression in P2 p_val	avg_log2FCsig

Xist	266E-73	1324849 significant Up

Tsix	224E-52	1062675 significant Up mt-Co3	558E-31	2590203 significant Up Uty	156E-30	-917592 significant Down Fkbp5	943E-26	2627455 significant Up mt-Co1	114E-25	2122163 significant Up mt-Atp6	394E-25	2237024 significant Up mt-Co2	261E-24	2725413 significant Up mt-Nd4	545E-21	2857004 significant Up mt-Cytb	972E-21	251437 significant Up mt-Nd1	104E-20	2529072 significant Up mt-Nd2	299E-20	2602825 significant Up Ddx3y	795E-15	-775077 significant Down Eif2s3y	117E-13	-739761 significant Down mt-Nd3	777E-13	3738257 significant Up mt-Nd5	101E-12	2646456 significant Up Kdm5d	254E-10	-680937 significant Down Ms4a4a	607E-10 1639963 significant Up mt-Nd4l 411E-08 2240892 significant Up Gm47283 728E-08 1402015 significant Up Ccnd3	886E-08 1347058 significant Up Mrc1	102E-06 0994557 significant Up F13a1	163E-06 0964016 significant Up Cd163	281E-06 1194359 significant Up Rbm47	303E-06 0741953 significant Up Rps29	334E-06 0702489 significant Up Ms4a6c	408E-06 1282002 significant Up Man2a1 651E-06 1188875 significant Up Lyve1	105E-05 1110177 significant Up Klf4	152E-05 1026234 significant Up Abca9	254E-05 0747464 significant Up Rbpj	303E-05 0687117 significant Up Tsc22d3 321E-05 1566892 significant Up Hif3a	328E-05 2508016 significant Up Vav3	480E-05 0969602 significant Up Gm30489 916E-05 150657 significant Up Gab3	0000107 0623382 significant Up

E330020D1 0000117	0933969 significant Up

Chka	0000132	1238022 significant Up

Anapc10	0000142	-200268 significant Down

Cnpy2	0000175	1914851 significant Up

Ms4a6b	0000243	0780515 significant Up

Rnf169	0000262	0749846 significant Up

AC149090.	0000297	-065474 significant Down

Dab2	00003	0475259 Up

Zbtb37	0000335	169638 significant Up

Tmsb10	0000393	1237846 significant Up

Lhfpl2	0000423	-131694 significant Down

| Nrp1 | 0000434 | 0488262 Up |
| --- | --- | --- |
| Arhgef3 | 0000462 | 0607966 significant Up |
| Dhx9 | 0000467 | 0633899 significant Up |
| Anxa3 | 0000496 | -099235 significant Down |
| Ppp2r5a | 0000505 | 0992545 significant Up |
| Egfr | 0000512 | 1354195 significant Up |
| Ptprc | 0000514 | -073553 significant Down |
| Osbpl9 | 0000541 | 0742217 significant Up |
| Gng2 | 0000582 | 0581885 significant Up |
| Il6st | 0000622 | -122026 significant Down |
| Sall1 | 0000641 | -155373 significant Down |
| Maf | 0000655 | 0411842 Up |
| Pf4 | 0000689 | 0627246 significant Up |
| Etv6 | 0000689 | -046048 Down |
| Col4a3bp | 0000698 | 0535515 Up |
| Ctnnd1 | 0000701 | 0893305 significant Up |
| Nrip1 | 0000759 | -091873 significant Down |
| Mctp1 | 0000861 | 0747402 significant Up |
| Bin2 | 0000872 | -194679 significant Down |
| Smurf2 | 0000873 | 0589819 significant Up |
| Sh3kbp1 | 0000906 | 0573176 Up |
| Gtf2a2 | 0000948 | 119809 significant Up |
| Tbl1xr1 | 0000966 | -075416 significant Down |
| Ncf1 | 0001 | 0696691 significant Up |
| Frmd4b | 0001062 | 0572536 Up |
| Ndufb8 | 0001167 | 0820219 significant Up |
| Kazn | 0001173 | -105084 significant Down |
| Ms4a7 | 0001178 | 0605977 significant Up |
| Colec12 | 0001334 | 0813592 significant Up |
| Clcn5 | 0001393 | 0744649 significant Up |
| Zfp951 | 0001447 | -144456 significant Down |
| Tlr4 | 0001452 | 0664526 significant Up |
| Sgce | 0001482 | -084878 significant Down |
| Wwp1 | 0001544 | 0512242 Up |
| Ftx | 0001564 | 1241792 significant Up |
| Camk2d | 0001622 | -06399 significant Down |
| C1qc | 0001644 | 0368822 Up |
| Wdr7 | 0001692 | -08942 significant Down |
| Ctsl | 0001731 | 0575865 Up |
| Snx2 | 0001806 | 0504809 Up |
| Sbf2 | 0001815 | 0485286 Up |
| Apbb1ip | 0001823 | -060542 significant Down |
| Uba52 | 0001945 | 1086666 significant Up |
| Lmln | 0002116 | 0858808 significant Up |
| Serinc3 | 000231 | 0518251 Up |
| Rnase4 | 0002314 | 0939652 significant Up |
| Aoah | 0002405 | 0813641 significant Up |
| Alms1 | 0002456 | 105476 significant Up |
| Tlr7 | 0002482 | 0718387 significant Up |
| Slc49a4 | 0002497 | 1169059 significant Up |

| Lpcat3 | 0002514 | -112637 significant Down |
| --- | --- | --- |
| Psd3 | 0002533 | 0443215 Up |
| Marcks | 0002607 | -053587 Down |
| Lcorl | 0002735 | 0439621 Up |
| Chd7 | 0002766 | -067646 significant Down |
| Nop10 | 0002794 | 0818661 significant Up |
| F630028O1 | 0002974 | 0469811 Up |
| Pgs1 | 0002998 | -206257 significant Down |
| Phlpp2 | 0003055 | 1502222 significant Up |
| Hgsnat | 000306 | -207781 significant Down |
| Rps21 | 0003062 | 0526794 Up |
| Pcca | 0003239 | 0594469 significant Up |
| Txnip | 0003243 | 0432937 Up |
| Osbpl11 | 000329 | 0555661 Up |
| Txnrd1 | 0003427 | -163735 significant Down |
| Ctnna1 | 0003633 | 081697 significant Up |
| Uqcrb | 0003698 | 1156905 significant Up |
| Rpl41 | 0003859 | 0505802 Up |
| Tpt1 | 0004109 | 0428377 Up |
| Lifr | 0004291 | 0668145 significant Up |
| Tgfa | 0004334 | -206892 significant Down |
| Acat1 | 0004382 | 0705981 significant Up |
| Map2k4 | 0004496 | -067556 significant Down |
| Rps17 | 0004547 | 0338624 Up |
| Stard8 | 0004765 | 0590215 significant Up |
| Gtf2a1 | 00048 | -123125 significant Down |
| Fcgrt | 0004865 | 0415876 Up |
| Pafah1b2 | 0005014 | 0888847 significant Up |
| Plxdc2 | 0005094 | -067023 significant Down |
| B4galt1 | 0005157 | 0667973 significant Up |
| Rhoh | 000524 | -149059 significant Down |
| Mat2a | 0005272 | -090943 significant Down |
| Eid1 | 0005327 | 100303 significant Up |
| Ddx31 | 0005338 | -106104 significant Down |
| Rab3gap1 | 0005339 | 0594907 significant Up |
| Msr1 | 0005465 | 0978831 significant Up |
| Pmpcb | 0005498 | 1876511 significant Up |
| Zfhx2 | 0005603 | -152973 significant Down |
| Itga6 | 0005722 | -079432 significant Down |
| Ap2a2 | 0005742 | 0556422 Up |
| Sema4d | 0005979 | -092113 significant Down |
| Enox2 | 0006032 | 0799664 significant Up |
| Snx5 | 0006032 | 0314224 Up |
| Parl | 0006039 | 0994744 significant Up |
| Adap2 | 0006296 | -07843 significant Down |
| Pdxdc1 | 0006426 | -07558 significant Down |
| Gm7072 | 0006496 | -080583 significant Down |
| Arsb | 0006695 | -063725 significant Down |
| Mif | 0006788 | 0802607 significant Up |
| Zhx3 | 0006979 | 0857968 significant Up |

| Ms4a6d | 0007263 | 0649359 significant Up |
| --- | --- | --- |
| Scamp2 | 0007279 | 0428402 Up |
| Rnf180 | 0007341 | -078344 significant Down |
| Ly96 | 0007405 | 136901 significant Up |
| Cd86 | 0007564 | 0482416 Up |
| Myo5a | 0007711 | 0527356 Up |
| Ehmt2 | 0007802 | 0980625 significant Up |
| Ccr1 | 0007872 | 1069339 significant Up |
| Hmgb1 | 0007946 | 0412775 Up |
| St6gal1 | 000802 | -057623 Down |
| Tmcc3 | 0008029 | -077317 significant Down |
| Cops4 | 0008054 | 1255875 significant Up |
| Rpl15 | 0008077 | 0331547 Up |
| Lair1 | 0008214 | -087167 significant Down |
| Birc3 | 0008269 | 1267356 significant Up |
| Nfxl1 | 000828 | 0526102 Up |
| Sec24c | 0008367 | -09093 significant Down |
| Sparc | 0008423 | -094953 significant Down |
| Ostf1 | 0008427 | 0417449 Up |
| Uchl1 | 0008987 | 0919752 significant Up |
| Gm26917 | 0009027 | -05567 Down |
| Dpm2 | 0009159 | -123778 significant Down |
| Casp8 | 0009219 | -076916 significant Down |
| Dock10 | 0009505 | -040869 Down |
| Mrtfb | 0009575 | 0688875 significant Up |
| Rasa4 | 0009599 | -105208 significant Down |
| Jmjd1c | 0009731 | -027065 Down |
| Rcc2 | 0009837 | 0678207 significant Up |
| Dennd5a | 0009893 | -058348 significant Down |
| Neurod6 | 0009893 | 1494084 significant Up |
| Mgat1 | 0010106 | 0570463 Up |
| Deptor | 0010196 | 058044 significant Up |
| Ncan | 0010446 | 0971082 significant Up |
| Xpot | 0010617 | -08078 significant Down |
| Lrp1 | 0010679 | -026345 Down |
| Tut7 | 0010796 | 0558689 Up |
| Ang | 0010953 | 0725616 significant Up |
| Cd200r1 | 0011158 | 1153807 significant Up |
| Eif2s3x | 0011625 | 0936412 significant Up |
| Phip | 0011663 | -0541 Down |
| Mxd4 | 0011928 | 1181423 significant Up |
| Atp6v0a2 | 0011984 | -100463 significant Down |
| Sh2b3 | 0012193 | 1204426 significant Up |
| BC004004 | 001229 | -094867 significant Down |
| Fuca1 | 0012292 | 0616126 significant Up |
| Hexa | 0012351 | 024775 Up |
| Rpl23a | 0012592 | 0312283 Up |
| Rps11 | 0012688 | 0411645 Up |
| Atp2b1 | 0012863 | 0198259 Up |
| 1110019D1 | 0012942 | 1784209 significant Up |

| Bcl10 | 0013026 | 1177623 significant Up |
| --- | --- | --- |
| Ndufb3 | 0013062 | 078021 significant Up |
| Ap1b1 | 0013069 | 04347 Up |
| Gm19951 | 0013164 | 0522655 Up |
| Commd7 | 0013245 | -123257 significant Down |
| Wdfy2 | 0013254 | 0591291 significant Up |
| Pikfyve | 0013294 | 0560083 Up |
| Trim14 | 0013486 | -09535 significant Down |
| Sncaip | 0013503 | -099509 significant Down |
| Lcp2 | 0013781 | -08745 significant Down |
| Msn | 0013781 | -066189 significant Down |
| Akap6 | 0013815 | -08655 significant Down |
| Igsf6 | 0013901 | 1120927 significant Up |
| Ier3ip1 | 0013901 | 0849641 significant Up |
| Spats2 | 0014109 | -088785 significant Down |
| Prkag2 | 0014258 | 0663734 significant Up |
| Mapk1 | 0014367 | 0670848 significant Up |
| Smg9 | 0014421 | -148914 significant Down |
| Fkbp15 | 001503 | 0426902 Up |
| Fcer1g | 0015113 | 0512095 Up |
| Uqcr11 | 0015135 | 0563583 Up |
| Hook3 | 0015186 | -070088 significant Down |
| Hadhb | 0015273 | -084188 significant Down |
| Arhgap24 | 0015405 | -105075 significant Down |
| Ptbp3 | 0015419 | 0353742 Up |
| Zfc3h1 | 0015456 | 0707356 significant Up |
| Fmnl3 | 0015465 | -136525 significant Down |
| Dido1 | 0015469 | -062943 significant Down |
| Bclaf3 | 0015494 | 090346 significant Up |
| Uri1 | 0015693 | -064654 significant Down |
| Zfp638 | 0015889 | 0394594 Up |
| Apobec1 | 0015954 | -079937 significant Down |
| Tsc22d4 | 0016035 | -038621 Down |
| Med12l | 0016045 | -070379 significant Down |
| Rplp2 | 0016075 | 036676 Up |
| Irf2bpl | 0016322 | -095919 significant Down |
| C87436 | 0016563 | -142175 significant Down |
| Rpl38 | 0016617 | 0402092 Up |
| Rps15a | 0016617 | 0307715 Up |
| Rpl35a | 001685 | 0557777 Up |
| Rcbtb2 | 0016904 | -094405 significant Down |
| Pot1b | 0017187 | 1427984 significant Up |
| Anks3 | 0017239 | -03357 Down |
| Cnep1r1 | 0017598 | 1055692 significant Up |
| Arhgef7 | 0017622 | -077672 significant Down |
| Arrdc3 | 0017663 | 1078136 significant Up |
| Cox7c | 0017672 | 0393279 Up |
| Zfand6 | 0017727 | 025007 Up |
| Riok1 | 0017922 | -124719 significant Down |
| Irf8 | 0017931 | -062883 significant Down |

| Ociad1 | 0017944 | 0828705 significant Up |
| --- | --- | --- |
| Map4k4 | 0018086 | -056268 Down |
| Btg1 | 0018147 | -055446 Down |
| Stk17b | 0018332 | 0800095 significant Up |
| Zfp318 | 0018377 | -056024 Down |
| Zc3h6 | 0018377 | 0728973 significant Up |
| Dnm1l | 0018424 | -056035 Down |
| Cspp1 | 0018637 | -079443 significant Down |
| Per1 | 0018656 | 0845165 significant Up |
| Gdi2 | 0018755 | -030803 Down |
| Etv1 | 0018907 | 0481939 Up |
| Accs | 0018979 | 0944443 significant Up |
| Map1lc3a | 0019048 | 0845148 significant Up |
| Cfh | 001919 | 0355137 Up |
| Hist1h2bc | 0019358 | -113277 significant Down |
| Galc | 0019684 | 0550925 Up |
| Tbxas1 | 0019802 | -051919 Down |
| Brip1os | 0019865 | -062915 significant Down |
| Atp5mpl | 0019932 | 0385626 Up |
| Rab5c | 0019966 | 0875381 significant Up |
| Clpp | 0020065 | -148355 significant Down |
| Sdk1 | 0020171 | -082782 significant Down |
| Hexb | 0020263 | -041551 Down |
| Eif1 | 0020285 | 0648852 significant Up |
| Gas5 | 0020439 | -095154 significant Down |
| Lamp1 | 0020664 | 0272755 Up |
| Nras | 0020765 | -111441 significant Down |
| Mylip | 0021005 | -078461 significant Down |
| Lpcat2 | 0021007 | -064981 significant Down |
| Gmps | 0021098 | 0773098 significant Up |
| Slc16a6 | 0021139 | -083531 significant Down |
| Mgll | 0021189 | -120612 significant Down |
| Plekhg5 | 0021238 | 0566268 Up |
| Gtpbp2 | 0021281 | -05361 Down |
| Tmem170b | 0021288 | -084438 significant Down |
| Atp5md | 0021712 | 067499 significant Up |
| Pdzd8 | 002172 | -113516 significant Down |
| Clec10a | 0022362 | 0358887 Up |
| Tln2 | 0022364 | 0676523 significant Up |
| Specc1 | 0022407 | -044931 Down |
| Mt1 | 0022465 | 063523 significant Up |
| Birc2 | 0022882 | 1503878 significant Up |
| Iqsec1 | 0023301 | -094001 significant Down |
| Uqcrq | 0023527 | 0511315 Up |
| Psenen | 0023647 | 1500282 significant Up |
| Gm26542 | 0023683 | -081135 significant Down |
| Cstb | 0024088 | 1497498 significant Up |
| Ptprk | 0024154 | 0430207 Up |
| Glyr1 | 0024235 | -061367 significant Down |
| Wapl | 0024621 | -047204 Down |

| Chst11 | 0024965 | -199364 significant Down |
| --- | --- | --- |
| Alkbh3 | 0024969 | 0971631 significant Up |
| Irf2bp2 | 0025047 | 0528976 Up |
| Zdhhc3 | 0025136 | -149005 significant Down |
| Rsrc1 | 0025324 | -040436 Down |
| Heatr6 | 0025585 | 0715361 significant Up |
| Slc25a37 | 0025957 | 0639746 significant Up |
| Bex2 | 0026005 | 0935998 significant Up |
| Abcc3 | 002602 | -057777 Down |
| Syt4 | 00261 | 0833876 significant Up |
| Arhgap35 | 0026101 | -078848 significant Down |
| Osgep | 0026149 | -117996 significant Down |
| Slc9a8 | 0026209 | 0752556 significant Up |
| Gm2629 | 0026454 | -075642 significant Down |
| Lgals1 | 0026653 | 0796256 significant Up |
| Ppp6r3 | 0026865 | -037769 Down |
| Ezh2 | 0026885 | -05448 Down |
| Ube2d2a | 0027242 | -060134 significant Down |
| Copg2 | 0027386 | 0809635 significant Up |
| Suclg2 | 002781 | -078151 significant Down |
| Itsn1 | 0027829 | 0401815 Up |
| Gm16286 | 0027875 | 1075191 significant Up |
| Mob4 | 0027952 | 0970375 significant Up |
| Polk | 0027996 | 0937451 significant Up |
| Tpd52 | 0028054 | -070527 significant Down |
| Csde1 | 0028297 | -049324 Down |
| Smim19 | 0028309 | 0914156 significant Up |
| Tlk1 | 0028378 | 0453867 Up |
| Kif21b | 0028755 | -070468 significant Down |
| Soga1 | 0029328 | -085912 significant Down |
| Vwa8 | 0029649 | 0503895 Up |
| Zc3h12b | 002998 | -108986 significant Down |
| Chmp3 | 0030395 | 0711407 significant Up |
| Smurf1 | 0030656 | 0806202 significant Up |
| Ap3b1 | 003068 | -040734 Down |
| Ndufa6 | 0030776 | 0939143 significant Up |
| Dnajc24 | 0030843 | -112353 significant Down |
| Tigd2 | 0031003 | 0880346 significant Up |
| Kcnk6 | 0031073 | 0725899 significant Up |
| Heatr5a | 0031493 | 0354488 Up |
| Cd24a | 0031714 | 0840172 significant Up |
| Zfp40 | 003191 | 0963504 significant Up |
| Ubn1 | 0031976 | 0778227 significant Up |
| Pros1 | 0032709 | -048412 Down |
| 7-Sep | 0032884 | 0341054 Up |
| Naga | 0032945 | 0475579 Up |
| Fxyd6 | 0033211 | 0950125 significant Up |
| Gmip | 0033245 | -045718 Down |
| Tox3 | 0033291 | -152524 significant Down |
| Rpl34 | 0033384 | 0473224 Up |

| Cox5b | 0033409 | 0665313 significant Up |
| --- | --- | --- |
| Tent2 | 0033639 | 0376827 Up |
| Ppp4r3b | 0034206 | 0345329 Up |
| Eif3a | 0034228 | -056456 Down |
| Slc25a12 | 003466 | -119431 significant Down |
| Fli1 | 0034676 | -032273 Down |
| Slc9a9 | 0034929 | 0333917 Up |
| Sipa1l1 | 0035089 | -074895 significant Down |
| Xpo6 | 003513 | -046309 Down |
| Gtf2h2 | 0035159 | -030474 Down |
| Tbc1d8 | 0035397 | -101058 significant Down |
| Pdcd5 | 0035795 | 1204047 significant Up |
| Acox3 | 0035797 | -080403 significant Down |
| Pcgf3 | 0035971 | -058094 significant Down |
| Eif5b | 0036065 | -07728 significant Down |
| Adk | 0036107 | -084533 significant Down |
| Ptpn1 | 0036386 | -073693 significant Down |
| Hip1 | 0036589 | 0646261 significant Up |
| Ctso | 0036939 | 0817511 significant Up |
| Mthfd1l | 0037266 | -087112 significant Down |
| Cuta | 0037903 | 1234607 significant Up |
| Csf3r | 0037936 | -087377 significant Down |
| Cul4a | 0038149 | 0529599 Up |
| Kcnq1ot1 | 0038174 | -048642 Down |
| Stard3nl | 0038238 | 0783779 significant Up |
| Snx24 | 0038304 | -03617 Down |
| Hdac9 | 003832 | 039312 Up |
| Nin | 0038325 | -066422 significant Down |
| Agap1 | 0038605 | -07065 significant Down |
| Pten | 003862 | 0454293 Up |
| Wars2 | 0038742 | 0493535 Up |
| Prex1 | 0038842 | -076453 significant Down |
| Rpl19 | 0039098 | 0303864 Up |
| Rpl22 | 0039124 | 0298202 Up |
| Rimklb | 0039352 | -06932 significant Down |
| Slc4a7 | 0039464 | -052039 Down |
| Pea15a | 0039807 | 1112831 significant Up |
| Nfatc2 | 0039859 | 0719077 significant Up |
| Rbbp8 | 0039932 | -083828 significant Down |
| Stat3 | 0040507 | 0387393 Up |
| Calm1 | 0040651 | 0339338 Up |
| Snrpg | 004077 | 0741354 significant Up |
| Pi4ka | 0040799 | -074499 significant Down |
| Sorl1 | 0040972 | -095048 significant Down |
| Slc40a1 | 0041021 | 0277925 Up |
| Ncoa5 | 0041045 | 0819955 significant Up |
| Sephs1 | 0041056 | 0946264 significant Up |
| Ankfy1 | 0041264 | 058314 significant Up |
| Gcnt1 | 0041637 | 0314343 Up |
| Mlxip | 0041862 | 0784691 significant Up |

| Klf7 | 0041924 | 046639 Up |
| --- | --- | --- |
| Wdfy3 | 0042037 | 0231423 Up |
| Tanc2 | 0042141 | -01795 Down |
| Ehd1 | 0042277 | 0155296 Up |
| Erbb4 | 0042467 | -031351 Down |
| Zfp398 | 0042691 | 0701945 significant Up |
| Ubtd2 | 0042716 | 1118969 significant Up |
| Cept1 | 0042841 | 0222909 Up |
| Cwc22 | 0042844 | 1253524 significant Up |
| Npc2 | 0042861 | 0252014 Up |
| Gramd1b | 0043589 | -049169 Down |
| Npnt | 0043958 | -12133 significant Down |
| Efcab14 | 0043973 | 0696541 significant Up |
| Fcna | 0044286 | 0399879 Up |
| Pnpla7 | 0044308 | 024342 Up |
| Stag1 | 0044361 | -023689 Down |
| Clec4a2 | 0044489 | 061631 significant Up |
| Eef1b2 | 0044935 | 0470071 Up |
| Ube2l3 | 0044959 | -074997 significant Down |
| Rpl36al | 0045088 | 0376424 Up |
| Ldb2 | 00451 | -128193 significant Down |
| Stxbp3 | 0045167 | 089632 significant Up |
| Large1 | 0045298 | -03765 Down |
| Mosmo | 0045333 | -081407 significant Down |
| AI480526 | 0045351 | -108915 significant Down |
| Pvt1 | 0045401 | -068682 significant Down |
| Mrpl23 | 0045405 | 0594295 significant Up |
| Grm7 | 0045495 | 0316193 Up |
| Eif2ak2 | 0045498 | -077822 significant Down |
| Pbrm1 | 0045532 | 0305006 Up |
| Usp29 | 0045537 | -127947 significant Down |
| Cdc40 | 0045611 | 0275863 Up |
| Ints6 | 0045621 | 0764113 significant Up |
| E2f3 | 0046006 | -05873 significant Down |
| Mrpl28 | 0046291 | 048535 Up |
| Mon2 | 0046847 | 0518387 Up |
| Dcaf13 | 0047131 | 0484055 Up |
| Tmem256 | 0047367 | 0756544 significant Up |
| Ccl12 | 0047597 | 0422619 Up |
| Frmd4a | 0047641 | -031007 Down |
| Atp5j2 | 0047704 | 0440804 Up |
| 0610040J0 | 0047833 | -058317 significant Down |
| Nfya | 0047909 | -0702 significant Down |
| Snrnp40 | 0048046 | 0494642 Up |
| Taf1b | 0048216 | -087902 significant Down |
| Med17 | 0048243 | 0573818 Up |
| Rnf19b | 0048279 | 0388646 Up |
| C1qb | 0048387 | 0229285 Up |
| Aars | 0048591 | -101855 significant Down |
| Pex13 | 0048659 | 0767114 significant Up |

| Ccnl2 | 0048708 | -055137 Down |
| --- | --- | --- |
| Kif16b | 0048805 | 0387974 Up |
| Morrbid | 0048816 | -064928 significant Down |
| Ranbp3 | 004887 | 0654484 significant Up |
| Pex5 | 0048903 | 0815936 significant Up |
| Tdp1 | 0048912 | -120605 significant Down |
| Sel1l | 0048974 | 0383103 Up |
| Snrnp48 | 0049208 | 0441167 Up |
| Tpk1 | 004931 | 1221621 significant Up |
| Max | 0049407 | -061751 significant Down |
| Eif4g1 | 0049415 | 0556646 Up |
| Ywhab | 0049425 | 0540577 Up |
| Tspan7 | 0049435 | -100875 significant Down |
| Sun1 | 0049744 | 0706855 significant Up |
| Tspan13 | 004977 | 0486776 Up |
| Rpl24 | 0050166 | 0328322 Up |
| Ythdf3 | 00503 | 0375772 Up |
| Tpi1 | 0050345 | -117494 Down |
| Kdm2b | 0051112 | -06672 Down |
| Srpk1 | 0051181 | -089061 Down |
| Rab18 | 0051294 | -079609 Down |
| Cdk5rap2 | 0051404 | 0850357 Up |
| Lrrtm4 | 0051463 | -058572 Down |
| Rpl36 | 0051488 | 0378301 Up |
| Ccnl1 | 0051784 | 0440877 Up |
| Plekha1 | 0051838 | 0240487 Up |
| Trit1 | 005203 | 0614542 Up |
| Rfc1 | 0052117 | 0736611 Up |
| Vps13c | 0052399 | -035987 Down |
| Mgat5b | 0052593 | -10635 Down |
| Serpine2 | 0052735 | -031069 Down |
| Hmox1 | 0052735 | 0565743 Up |
| Bsg | 0052881 | 038673 Up |
| Spock1 | 0053007 | -06466 Down |
| Galnt1 | 0053146 | 0473394 Up |
| Ap2s1 | 0053311 | 0413174 Up |
| 4933406I1 | 0053647 | -045415 Down |
| Pgrmc1 | 0053675 | 1006015 Up |
| Ldlrad4 | 0053845 | -047265 Down |
| Lamp2 | 0053985 | 0320956 Up |
| Setdb1 | 0054009 | 0564681 Up |
| Fkbp1a | 005416 | 0834219 Up |
| Cast | 0054265 | 0623887 Up |
| Gucy1b1 | 0054314 | 0281181 Up |
| Elk3 | 005433 | -05038 Down |
| Paip1 | 0054439 | 0949026 Up |
| Ndufa1 | 0054593 | 0551118 Up |
| Ankrd10 | 0054889 | -080111 Down |
| Wbp2 | 0054897 | -064899 Down |
| Rffl | 0055075 | -071237 Down |

| Secisbp2 | 0055102 | -068069 Down |
| --- | --- | --- |
| Rplp1 | 0055186 | 0283444 Up |
| Nrep | 0055242 | 0829476 Up |
| Epb41l2 | 0055336 | -037621 Down |
| Dym | 0055515 | -082748 Down |
| Rabgap1 | 0055572 | -036791 Down |
| Dync1h1 | 0055587 | -049595 Down |
| Cyb5a | 005563 | 0727397 Up |
| Rapgef1 | 0055671 | -069476 Down |
| Wdr43 | 0055877 | -056271 Down |
| Psmd1 | 0055972 | -06389 Down |
| Dtd1 | 0056194 | 0846778 Up |
| 1700086O | 0056194 | 1206638 Up |
| Herc4 | 0056473 | 0418899 Up |
| Ntrk3 | 0056589 | -041388 Down |
| Mphosph8 | 0056677 | -099008 Down |
| Jpx | 005684 | 0547101 Up |
| Picalm | 0056962 | 0296603 Up |
| Nsa2 | 0057064 | 0492677 Up |
| Nr6a1os | 0057345 | -077006 Down |
| Pantr1 | 0057436 | 0718028 Up |
| Rpl4 | 005774 | -079257 Down |
| Mtmr6 | 0057759 | 0338098 Up |
| Tmem234 | 0057959 | 0475572 Up |
| Aco1 | 0058028 | -094611 Down |
| Rps25 | 0058288 | 0609551 Up |
| Cybb | 0058374 | -072987 Down |
| Cnot7 | 0058577 | -04484 Down |
| Peli2 | 0058718 | -058831 Down |
| Ier5 | 0058889 | -120779 Down |
| Rps28 | 0058957 | 0430874 Up |
| Ifi207 | 0059084 | 0370418 Up |
| Pabpn1 | 0059174 | -035543 Down |
| Ubtf | 0059243 | 0508094 Up |
| Slc16a2 | 0059318 | -068795 Down |
| Ncor2 | 0059349 | -07372 Down |
| Nr3c1 | 0059513 | -057396 Down |
| Kdm6a | 0059529 | 0782532 Up |
| Ifitm2 | 0060188 | 0743957 Up |
| Cdc23 | 0060393 | -09455 Down |
| Faap100 | 0060468 | -055365 Down |
| Rfwd3 | 0060481 | -076701 Down |
| Mphosph9 | 0060714 | -110645 Down |
| Tyrobp | 0060801 | 0437066 Up |
| Gnai1 | 006085 | -069747 Down |
| Tbc1d9 | 006148 | -024465 Down |
| Eea1 | 006181 | 0324396 Up |
| Maea | 0061872 | 0719981 Up |
| Nt5c2 | 0062321 | 046716 Up |
| Lgr4 | 0062959 | -120062 Down |

| Csk | 0063175 | 070201 Up |
| --- | --- | --- |
| Prkcb | 0063477 | -052339 Down |
| Ago2 | 0063759 | -041437 Down |
| Agmo | 0064012 | 023106 Up |
| Fam118a | 0064432 | 1568857 Up |
| Sdc4 | 0064494 | 0903805 Up |
| Stab1 | 006491 | 0257382 Up |
| Bckdhb | 0065253 | -116317 Down |
| Cox6b1 | 0065623 | 0297793 Up |
| Usp36 | 0065847 | 084416 Up |
| Nrxn1 | 0066016 | -020857 Down |
| Ghitm | 0066061 | 0559227 Up |
| Pkp4 | 0066404 | -01913 Down |
| Gga2 | 0066821 | -132359 Down |
| Usp3 | 0067789 | -026155 Down |
| Ints2 | 0067867 | -093677 Down |
| Cdc73 | 0067924 | -050045 Down |
| Cdon | 0068422 | -075579 Down |
| Plcg2 | 0068517 | -02994 Down |
| Abcd2 | 0068596 | -062057 Down |
| Rc3h2 | 0068698 | -06085 Down |
| Klf12 | 0068704 | -054788 Down |
| Prdm10 | 0068819 | -058177 Down |
| Rbms1 | 0068903 | 0439864 Up |
| Hat1 | 0069236 | -074025 Down |
| Fnta | 0069268 | 1457951 Up |
| Upf2 | 0069538 | -04797 Down |
| Cyth4 | 0069581 | -051239 Down |
| Arnt | 0069648 | 0294031 Up |
| Crebbp | 006979 | -03784 Down |
| Ikbkb | 0069856 | -058855 Down |
| Vapa | 007014 | 0385953 Up |
| Ugcg | 0070565 | 0582426 Up |
| Grn | 007069 | 0205188 Up |
| Copb1 | 0070703 | 0615322 Up |
| Dnmt3a | 007074 | -051182 Down |
| Prmt3 | 0070856 | -071992 Down |
| Zfp608 | 007099 | -04858 Down |
| Ints10 | 0071121 | -07905 Down |
| Stt3b | 0071552 | -045754 Down |
| Cox6c | 0071756 | 0725885 Up |
| Tomm7 | 0072073 | 0626458 Up |
| Prpf38b | 0072242 | 0334906 Up |
| Nagk | 0072316 | 0359794 Up |
| Colgalt1 | 0072679 | 0239172 Up |
| Tnks2 | 0072686 | -036943 Down |
| Mtss1 | 0072699 | 0413238 Up |
| G3bp2 | 0073173 | -050555 Down |
| Rars | 0073341 | -105336 Down |
| Thrap3 | 0073348 | -05514 Down |

| Srp14 | 0073487 | 0503463 Up |
| --- | --- | --- |
| Rer1 | 0073611 | 0849845 Up |
| Chd9 | 0074009 | -039111 Down |
| Tafa5 | 007448 | -060792 Down |
| Zfp182 | 0074548 | 0449039 Up |
| Fgfr2 | 0074638 | 0292789 Up |
| Pde7b | 0075151 | 0470294 Up |
| Rpl23 | 0075344 | 03375 Up |
| Ppp1cb | 0075576 | 0569691 Up |
| Pcna | 007577 | 078561 Up |
| Plekhm3 | 007578 | -064674 Down |
| Mdfic | 0075907 | 0399226 Up |
| Ybx3 | 0075975 | -067991 Down |
| Tbc1d14 | 0076336 | 0303655 Up |
| Cpsf3 | 0076421 | 0400582 Up |
| Dusp6 | 0076771 | 0507308 Up |
| Cacnb2 | 00771 | 0205546 Up |
| Agl | 007713 | -081114 Down |
| Arhgap17 | 0077142 | -028446 Down |
| Evl | 0077258 | -035225 Down |
| Cyld | 0077502 | -080544 Down |
| Adam9 | 0077748 | 0378005 Up |
| Slco2b1 | 0077794 | 0164142 Up |
| Zfp329 | 0077797 | 1208514 Up |
| Tsc1 | 007794 | 0692836 Up |
| Cadm1 | 0078317 | -067158 Down |
| Mboat2 | 0078516 | -100477 Down |
| 1700012D1 | 0078551 | 0582537 Up |
| Map3k20 | 0078731 | -070878 Down |
| R3hdm2 | 0078759 | -027975 Down |
| Grb2 | 0079462 | -03826 Down |
| Sik3 | 0079664 | -050968 Down |
| Zbtb34 | 0079743 | 0486723 Up |
| Sp3 | 0079803 | 033881 Up |
| Dock11 | 0079899 | -031476 Down |
| Phf12 | 0080158 | -066744 Down |
| Leng8 | 0080334 | 0650594 Up |
| Stk10 | 0080393 | -056074 Down |
| Atp5g1 | 0080461 | 0176815 Up |
| Zfp407 | 0080787 | 0328761 Up |
| Ago3 | 0080915 | 0224329 Up |
| Atad1 | 008102 | -071858 Down |
| Atg10 | 0081363 | 018743 Up |
| Rpl27a | 0081511 | 0403503 Up |
| Tgfbi | 008175 | 0590908 Up |
| Clmp | 0081804 | 099675 Up |
| Ryr2 | 0081932 | 1188184 Up |
| Diaph1 | 0082116 | -057621 Down |
| Kcnk13 | 0082338 | -05134 Down |
| Pde3b | 0082622 | -063384 Down |

| Nebl | 0082679 | -069491 Down |
| --- | --- | --- |
| Ndufb7 | 0082807 | 0351317 Up |
| Ash1l | 0082914 | 0321832 Up |
| Zmym6 | 0082977 | -076284 Down |
| Klf6 | 0083042 | 0276985 Up |
| Tcf12 | 0083111 | -023366 Down |
| Rhobtb1 | 0083348 | 0266144 Up |
| Dna2 | 0083402 | -097434 Down |
| Palld | 008343 | 0559785 Up |
| Chpt1 | 0083574 | -060275 Down |
| Adrb2 | 0083614 | 0440941 Up |
| Daam1 | 008382 | -071734 Down |
| Apbb2 | 0084064 | -042967 Down |
| Dgkb | 0084091 | -029255 Down |
| Hps3 | 0084382 | 0441156 Up |
| Gm28379 | 0084532 | 0724538 Up |
| Mid1 | 0084744 | 0397924 Up |
| Plcl1 | 0084984 | -053535 Down |
| Reep3 | 0085233 | -033456 Down |
| Nr3c2 | 0085367 | -04487 Down |
| Carnmt1 | 0085367 | 0813219 Up |
| Orai1 | 0085425 | -049396 Down |
| Gas2l3 | 0085449 | 0612334 Up |
| Lin7c | 0085599 | -066147 Down |
| Ptpn11 | 0085849 | -063341 Down |
| Tmsb4x | 0085907 | 0237637 Up |
| Map3k14 | 0085943 | -075055 Down |
| Gnpat | 0085986 | -055074 Down |
| Prkar1a | 008617 | -065522 Down |
| Vps37b | 0086352 | 0762865 Up |
| Pou3f3 | 0086396 | -05775 Down |
| Socs6 | 0086655 | 0201383 Up |
| Itgav | 0086761 | -069572 Down |
| Mcu | 0087004 | -057844 Down |
| Usp37 | 0087246 | -040195 Down |
| Heatr3 | 0087328 | -051539 Down |
| Kpna4 | 0087338 | -045588 Down |
| Hpf1 | 0087552 | -075014 Down |
| Ubp1 | 0087899 | 0424396 Up |
| Actr2 | 0088282 | -036695 Down |
| Asph | 0088462 | -065003 Down |
| Tmem164 | 0088771 | -034044 Down |
| Pcf11 | 0088858 | -035109 Down |
| Wasl | 0088874 | 0192431 Up |
| Plxna4 | 0089141 | -034206 Down |
| Dhx15 | 0089182 | -039667 Down |
| Herc3 | 0089424 | -047611 Down |
| Hivep3 | 0089603 | 0458595 Up |
| Uso1 | 0089645 | -086165 Down |
| Cd9 | 0089954 | -047782 Down |

| Clec4a1 | 0089976 | 0641359 Up |
| --- | --- | --- |
| Lacc1 | 0090189 | -050784 Down |
| Cmss1 | 00904 | 0179796 Up |
| Cope | 0090512 | 0566716 Up |
| Clint1 | 0090539 | -054481 Down |
| Ppil4 | 0090785 | -107106 Down |
| Chd2 | 0090931 | 0269928 Up |
| Gpcpd1 | 009122 | -063235 Down |
| Abcc1 | 0091436 | 0376397 Up |
| Fam102b | 0091457 | -083004 Down |
| Smarcc1 | 0091513 | -042461 Down |
| Rnf216 | 0091798 | -037634 Down |
| Uba2 | 0091918 | -041229 Down |
| Mmd | 0092202 | 0675317 Up |
| Traf6 | 0092486 | -054065 Down |
| Nid2 | 0092657 | 0725595 Up |
| Sptlc1 | 0092657 | -111474 Down |
| Clasp2 | 009279 | -045491 Down |
| Dscaml1 | 0092826 | -096816 Down |
| Kdm7a | 0092964 | 0272485 Up |
| Tbck | 0093243 | -041258 Down |
| Slc45a4 | 0093389 | 0474046 Up |
| Actr10 | 0093747 | 0503436 Up |
| Emc8 | 0093855 | -054318 Down |
| Mdga2 | 0094044 | -026978 Down |
| Trappc6b | 0094066 | -048508 Down |
| Spire1 | 0094342 | 0213865 Up |
| Tfec | 0094499 | 0573764 Up |
| Pdcd2l | 0094688 | -047689 Down |
| Kcnk2 | 0094837 | -094331 Down |
| Tmem199 | 0094934 | 0511864 Up |
| Top2b | 0095769 | -050503 Down |
| Madd | 0095786 | -058947 Down |
| Rtn1 | 0096083 | 0397482 Up |
| Psmb3 | 009609 | -053675 Down |
| Ppwd1 | 0096314 | -033311 Down |
| Npl | 0096422 | -052252 Down |
| Cbx3 | 0096425 | -056189 Down |
| Fzd3 | 0096468 | -098712 Down |
| Noc2l | 0096878 | -070474 Down |
| Gns | 009688 | 0361541 Up |
| Capza1 | 0097045 | 0368459 Up |
| Ppp4r2 | 009706 | -045921 Down |
| Map3k1 | 009723 | -028193 Down |
| Uvrag | 0097243 | 0299825 Up |
| Sumo2 | 0097406 | 0432413 Up |
| Cbx1 | 0097451 | 0593868 Up |
| Igf1r | 0097554 | -028337 Down |
| Selenot | 0097647 | 02849 Up |
| Proser1 | 009776 | -119746 Down |

| Rps9 | 0097974 | 0227419 Up |
| --- | --- | --- |
| Atl3 | 0098028 | 0955263 Up |
| Gnaq | 0098067 | 0165293 Up |
| Jmy | 0098221 | -041116 Down |
| Cmklr1 | 0098723 | 0789146 Up |
| Vti1b | 0098895 | -090544 Down |
| Rasgrp3 | 0099247 | -040705 Down |
| Trpm3 | 0099249 | 037343 Up |
| Zc3h3 | 0099451 | -065876 Down |
| Prpf39 | 0099559 | -022979 Down |
| Marf1 | 0099568 | -062278 Down |
| Elp1 | 0099959 | -038854 Down |
| Rps3a1 | 0100398 | 0245126 Up |
| Trpc4ap | 010045 | 0353832 Up |
| Tcf25 | 010079 | -051492 Down |
| Gm20732 | 0101022 | 0817483 Up |
| Mgst3 | 0101224 | 1142562 Up |
| Chmp4b | 0101288 | 025308 Up |
| Naa40 | 0101489 | -05845 Down |
| Adcy9 | 0101593 | 040772 Up |
| Ubtd1 | 0101747 | -066945 Down |
| Fcgr1 | 0101941 | 0833824 Up |
| Agfg1 | 0102239 | 0645988 Up |
| 2700049A0 | 0102463 | -060852 Down |
| Hint1 | 0102506 | 0384712 Up |
| Eif5a | 0102616 | 0642002 Up |
| Nectin3 | 0102661 | 0927413 Up |
| Zfp131 | 0102665 | -051195 Down |
| Chd3 | 0102849 | -038351 Down |
| Ankrd13c | 0102892 | -055914 Down |
| Gm4258 | 0102964 | 0215817 Up |
| Tubb2b | 0103293 | 048298 Up |
| Dnm1 | 0103319 | 0571943 Up |
| Sat1 | 0103392 | -030427 Down |
| Gsk3b | 0103397 | -026769 Down |
| Tdrd7 | 0103406 | 0663625 Up |
| Magi1 | 0103465 | -051665 Down |
| Zeb2os | 0103847 | -050092 Down |
| Ezh1 | 0103935 | -076764 Down |
| Eef1d | 0104102 | 0745041 Up |
| Dhx36 | 0104273 | -02794 Down |
| Cep95 | 0104278 | -0644 Down |
| Tdrd3 | 0104352 | -071636 Down |
| Pcmt1 | 0104403 | 0495308 Up |
| Kdm3b | 0104417 | -032166 Down |
| Strn | 0104453 | 0567857 Up |
| Ssbp1 | 0104499 | -047369 Down |
| Nubpl | 0104608 | 0557217 Up |
| Ncoa1 | 0104729 | 0502814 Up |
| Gnptab | 0104826 | -06748 Down |

| Atg4c | 0104896 | -087238 Down |
| --- | --- | --- |
| Snx3 | 0105224 | 0471637 Up |
| Elavl4 | 0105447 | -017524 Down |
| Rpl3 | 0105657 | -047007 Down |
| Dlat | 0105723 | -05586 Down |
| Zrsr2 | 010594 | -066158 Down |
| Oga | 0105944 | 024334 Up |
| Zdhhc21 | 0106121 | -040589 Down |
| Mapkapk2 | 0106125 | -045473 Down |
| Dctn1 | 0106178 | -054675 Down |
| Nap1l4 | 0106642 | 0594677 Up |
| Ppia | 0106689 | 0316782 Up |
| Clec4a3 | 0106793 | 0333835 Up |
| Foxp1 | 01068 | 0217687 Up |
| S100pbp | 0107075 | 0745676 Up |
| Fcgr2b | 0107203 | 032817 Up |
| Nufip2 | 010775 | 0256451 Up |
| Ep300 | 0107831 | 0286401 Up |
| Ctbp1 | 0107987 | 0723521 Up |
| Tle3 | 0107987 | 054361 Up |
| Mdn1 | 010837 | -082228 Down |
| Elob | 0108388 | 074211 Up |
| Tsen34 | 0108426 | 1056491 Up |
| C530008M | 0108468 | -074132 Down |
| Plk2 | 0108485 | 0367364 Up |
| Rhot1 | 0108655 | -02945 Down |
| Rasal2 | 0108831 | 0155433 Up |
| Ppig | 0108961 | 0422097 Up |
| Selenok | 010924 | -084967 Down |
| Trappc12 | 0109344 | 0257187 Up |
| Ergic3 | 010953 | 0469894 Up |
| Cdr1os | 010979 | -072252 Down |
| Tomm40 | 0110027 | -075634 Down |
| Ncor1 | 0110279 | -023 Down |
| Maml3 | 0110618 | -017639 Down |
| Arhgap20 | 0110838 | -088377 Down |
| Rps23 | 0110894 | 0354579 Up |
| Hnrnpu | 011108 | 0259544 Up |
| Tmem135 | 0111176 | -042612 Down |
| Gtf3c1 | 0111542 | -079965 Down |
| Hdlbp | 0111571 | -035255 Down |
| Pde10a | 0111607 | -068897 Down |
| Gcfc2 | 0111771 | 0758324 Up |
| Ltn1 | 0111845 | -064037 Down |
| Ulk4 | 0111959 | -084564 Down |
| Ints7 | 0112052 | -070163 Down |
| Rbl2 | 0112058 | -103971 Down |
| Pon2 | 0112192 | 0374307 Up |
| Cpne1 | 0112426 | 0526862 Up |
| Myo9a | 0112582 | -022791 Down |

| Lrmda | 0112659 | -030335 Down |
| --- | --- | --- |
| Rock1 | 0112886 | 0341601 Up |
| Ino80d | 0113152 | 0576836 Up |
| Scand1 | 0113316 | 0534349 Up |
| Usp45 | 0113331 | 0903702 Up |
| Rpl26 | 0113482 | 0250151 Up |
| Stxbp5l | 011383 | -086652 Down |
| Rsbn1 | 0114162 | 041229 Up |
| Bmpr2 | 0114251 | 040643 Up |
| Ist1 | 0114365 | -065959 Down |
| Smim14 | 0114454 | -063951 Down |
| Ibtk | 0114491 | -067022 Down |
| Azi2 | 0114521 | 0601501 Up |
| Rnf150 | 0114533 | 0358755 Up |
| Ndufv2 | 0114606 | 0628385 Up |
| Mterf3 | 0114609 | -07801 Down |
| Carm1 | 0114628 | -138215 Down |
| Gcn1 | 011497 | -030189 Down |
| Exoc5 | 0114975 | 0385584 Up |
| Tnrc6c | 0115205 | -026677 Down |
| Dnajc7 | 0115332 | 0156152 Up |
| Eif3c | 011564 | -06474 Down |
| Rchy1 | 0115855 | 0398842 Up |
| Flcn | 0116124 | -045685 Down |
| Cacna1d | 0116716 | -046693 Down |
| Lrfn5 | 0116781 | -044787 Down |
| Eda | 011709 | -074812 Down |
| Gas6 | 0117468 | 024256 Up |
| Akap8l | 0117633 | 0353184 Up |
| 1600010M | 0117675 | -065515 Down |
| Map3k2 | 0118253 | 0327192 Up |
| Ube2a | 011848 | 0448548 Up |
| Fam133b | 0118498 | -061351 Down |
| Qk | 0118744 | -020441 Down |
| Prkca | 0119734 | 0327043 Up |
| Dnajb14 | 0119953 | 0574091 Up |
| Nrg1 | 0119981 | -031068 Down |
| Gbp7 | 012009 | 0489023 Up |
| Vwa5a | 0120135 | -078857 Down |
| Fbxw8 | 0120277 | -076195 Down |
| Hmg20a | 0120456 | -045995 Down |
| Wdr26 | 0120629 | 0231173 Up |
| Abcd3 | 012104 | 0599539 Up |
| Sla | 0121076 | 0644933 Up |
| Cep85l | 0121131 | -055809 Down |
| Skil | 0121166 | -04913 Down |
| Eefsec | 0121551 | -063611 Down |
| Lclat1 | 0121577 | -078286 Down |
| Cd63 | 012184 | 0455833 Up |
| Atp5d | 0122121 | 0499039 Up |

| Ctsa | 0122718 | 0281289 Up |
| --- | --- | --- |
| Cog4 | 012296 | -057791 Down |
| Glud1 | 0122987 | -043831 Down |
| Plcb4 | 0123 | 0819958 Up |
| Ppfia1 | 0123002 | -028834 Down |
| Ints9 | 0123287 | 0400538 Up |
| Galnt11 | 0123337 | -058587 Down |
| Fgf13 | 0123772 | -062819 Down |
| Tcf4 | 0123934 | -017685 Down |
| Zfyve9 | 0123988 | -049023 Down |
| Dock2 | 012399 | -030608 Down |
| Vta1 | 0124104 | -046695 Down |
| Hmgcs1 | 012444 | 0783565 Up |
| Fchsd2 | 0124514 | 0256689 Up |
| Gpr155 | 0124519 | 0995488 Up |
| Cd47 | 0124554 | 0318219 Up |
| Zfr | 0124662 | -023681 Down |
| Ncapd3 | 0124723 | 0182997 Up |
| Rnf115 | 0124846 | -064658 Down |
| Eef2k | 0125116 | 07436 Up |
| Gspt1 | 012527 | -059076 Down |
| Ranbp1 | 0125531 | 0898875 Up |
| Ndufa2 | 0125701 | 0424321 Up |
| Vdac1 | 0125925 | -060264 Down |
| Ppp1r9a | 0126059 | 0195455 Up |
| Apc2 | 0126335 | 0895102 Up |
| Acss1 | 0126484 | -06328 Down |
| Nadk | 0126529 | -048218 Down |
| Ifi204 | 0126915 | 0378279 Up |
| Brinp3 | 012698 | -085698 Down |
| Ninl | 0127113 | -067934 Down |
| Tnrc6a | 0127711 | -018086 Down |
| 6720427I0 | 0127734 | 0399971 Up |
| Usp31 | 0127875 | 0830213 Up |
| H3f3b | 012833 | -021644 Down |
| Aig1 | 0128423 | -034896 Down |
| Rab8b | 0128478 | 0352216 Up |
| Psma3 | 0128887 | 0327443 Up |
| Nfic | 0128964 | -054617 Down |
| Tmem39a | 0129025 | 0578957 Up |
| Slc35a3 | 0129028 | -066071 Down |
| Nedd1 | 0129106 | 0525681 Up |
| Fopnl | 0129106 | 0658567 Up |
| Zfp942 | 0129235 | 0529731 Up |
| Itm2c | 0129311 | 0259357 Up |
| Ndufb4 | 0129445 | 0381789 Up |
| Nipsnap2 | 0129534 | 0798203 Up |
| Ppp1r21 | 0129844 | 0589489 Up |
| Usp33 | 0129989 | -068224 Down |
| Egr1 | 013026 | -039055 Down |

| Trpc4 | 0130287 | -061359 Down |
| --- | --- | --- |
| Bicra | 0130334 | -031794 Down |
| Ski | 01304 | -03862 Down |
| Eftud2 | 0130517 | -015453 Down |
| Atp2c1 | 0131122 | -051091 Down |
| Kdm5c | 0131198 | 0749517 Up |
| N4bp1 | 0131263 | 0304555 Up |
| Dyrk1a | 0131978 | -022374 Down |
| Mark2 | 0132078 | -03816 Down |
| Sdhc | 0132127 | -072564 Down |
| Dcaf6 | 0132917 | 0670789 Up |
| Cdk5r1 | 0132938 | 0609576 Up |
| Ube2e1 | 0133104 | 051117 Up |
| Snap25 | 0133353 | -050307 Down |
| Hist1h1c | 0133408 | -0573 Down |
| Wasf1 | 0133416 | -035679 Down |
| Usf2 | 0133495 | -021212 Down |
| Nnat | 0133832 | 0302527 Up |
| Pias1 | 0133947 | 0245405 Up |
| Lrrfip1 | 0133969 | -052924 Down |
| Unk | 0134349 | 0565754 Up |
| Smap1 | 0134399 | 0613484 Up |
| Rian | 0134714 | -070065 Down |
| Cds2 | 0134743 | 0472408 Up |
| Chd4 | 0134941 | -040632 Down |
| Zc3hav1 | 0135009 | -063785 Down |
| Shtn1 | 0135033 | -07805 Down |
| Cd300a | 0135454 | -041731 Down |
| Dnttip1 | 0135461 | -077362 Down |
| Ptprn2 | 0135656 | -080483 Down |
| Tent5a | 0135696 | 0431893 Up |
| Vps45 | 0135889 | -055159 Down |
| Dzip3 | 0135904 | 0365025 Up |
| Lncpint | 0135933 | -055056 Down |
| Mgat5 | 0136215 | -034305 Down |
| Spata6 | 0136487 | 0785831 Up |
| Gm42047 | 0136936 | 0289805 Up |
| Atpif1 | 0137373 | 0441794 Up |
| Ric1 | 0137425 | -036128 Down |
| Eif4g3 | 0138007 | -037777 Down |
| Zeb1 | 0138101 | -046679 Down |
| Eya3 | 0138349 | -045711 Down |
| Luzp1 | 0138515 | 0816196 Up |
| Mfsd11 | 0138533 | 0372335 Up |
| Ssh1 | 0138774 | -080642 Down |
| Kcnab1 | 0139125 | -090258 Down |
| Ercc6 | 01392 | -070175 Down |
| Snx13 | 0139722 | 0217885 Up |
| Tma7 | 0140054 | 0638285 Up |
| Papola | 0140078 | -039138 Down |

| Pi4kb | 0140358 | -046596 Down |
| --- | --- | --- |
| Atp11b | 0140377 | 035415 Up |
| Spg11 | 0140494 | -023147 Down |
| Tmem106b | 0140663 | 05429 Up |
| Blnk | 0140977 | -024347 Down |
| Txlna | 0141722 | -091837 Down |
| Rab5b | 0141941 | 0503848 Up |
| Drg1 | 0142085 | -053198 Down |
| Adcy7 | 0142265 | 035011 Up |
| Tceal9 | 0142532 | 0355543 Up |
| Pan3 | 0142649 | -025125 Down |
| Fez1 | 0142843 | -070701 Down |
| Kpna1 | 0142924 | 0485998 Up |
| Cask | 0142982 | 0302001 Up |
| Dipk1a | 0143075 | 0507693 Up |
| Rab7 | 01437 | 0369603 Up |
| Zfp1 | 0143899 | 0562451 Up |
| Rab12 | 0144363 | 034602 Up |
| Man2b1 | 0144792 | 0171922 Up |
| Cuedc2 | 0144794 | 0479626 Up |
| Mta3 | 0145111 | 0467151 Up |
| Ptprz1 | 0145427 | -042698 Down |
| Xpr1 | 0145675 | 0350331 Up |
| Gab1 | 014576 | 0301938 Up |
| Gstcd | 0145808 | -081611 Down |
| Tmbim6 | 014618 | 0229492 Up |
| Myo9b | 0146326 | -045488 Down |
| Tmem181a | 0146345 | -047255 Down |
| Gm10790 | 0146404 | -063632 Down |
| 9-Sep | 0146445 | -028922 Down |
| Rnf11 | 0146487 | -064864 Down |
| Slc38a9 | 0146732 | -049479 Down |
| Celf4 | 0146776 | -052057 Down |
| Nf2 | 0146806 | 0457942 Up |
| Dner | 0146845 | -071288 Down |
| Hmg20b | 0147121 | -091604 Down |
| Zfx | 0147333 | 0465851 Up |
| Smarcc2 | 0147511 | -051663 Down |
| Dock4 | 0147842 | -020951 Down |
| Sub1 | 0148029 | 0326183 Up |
| Npat | 0148236 | -060351 Down |
| Kdm6b | 0148513 | -048688 Down |
| Enox1 | 0148527 | -046308 Down |
| AU020206 | 0148893 | -052752 Down |
| Camk4 | 014928 | -031431 Down |
| Cebpz | 0149564 | -081229 Down |
| Paxbp1 | 014959 | -040071 Down |
| Nfe2l2 | 0149972 | -061966 Down |
| Itpkb | 0150203 | -021822 Down |
| Ythdf1 | 0150459 | 091277 Up |

| Arid1a | 015066 | 0273059 Up |
| --- | --- | --- |
| Aqr | 0150777 | -041355 Down |
| Gpbp1l1 | 0150777 | 0703421 Up |
| Piezo1 | 015097 | -033054 Down |
| Igfbp4 | 0151138 | 0406834 Up |
| Filip1l | 0151398 | 0273391 Up |
| Ufd1 | 0151583 | 0259421 Up |
| Ogdh | 0151758 | -058856 Down |
| Riok2 | 0151823 | -093496 Down |
| Cttnbp2nl | 0152239 | -019049 Down |
| Otulinl | 0152557 | -060492 Down |
| Zfp62 | 0152919 | -054922 Down |
| Gm5086 | 0152955 | -045273 Down |
| Marcksl1 | 0153151 | 0864953 Up |
| Nop56 | 0153272 | -116206 Down |
| Lats1 | 0153748 | -045953 Down |
| Enc1 | 0153778 | 029397 Up |
| Mir100hg | 0154058 | -029383 Down |
| Sbno1 | 0154371 | -078583 Down |
| Sirt7 | 0154492 | -093512 Down |
| Zfp386 | 0154798 | -063918 Down |
| Katnbl1 | 0155019 | -04049 Down |
| Parp8 | 0155026 | -05109 Down |
| Psmd14 | 0155245 | 0771989 Up |
| Pygb | 0155668 | -053678 Down |
| Usp6nl | 0156282 | -037663 Down |
| Slc6a6 | 0156425 | -022878 Down |
| Nedd8 | 015666 | 0398909 Up |
| Cpd | 0157176 | -038926 Down |
| Ctdp1 | 0157555 | 065442 Up |
| Kat6b | 015784 | -040108 Down |
| Psmd4 | 0157849 | 0373555 Up |
| Tm2d1 | 0157892 | 0283172 Up |
| Gpd2 | 0158087 | -076008 Down |
| Cx3cr1 | 0158107 | -022759 Down |
| Pard3b | 01583 | 0339189 Up |
| Cul5 | 0158748 | -054333 Down |
| H2afj | 0159083 | -063854 Down |
| Erp29 | 0159212 | -036252 Down |
| Nckap5 | 0159725 | -036281 Down |
| Trip11 | 0159824 | -030933 Down |
| Ggnbp2 | 0159856 | -033894 Down |
| Klf13 | 01604 | 0706968 Up |
| Rp2 | 0161211 | 0336971 Up |
| Hnrnpa0 | 0161611 | -03365 Down |
| Wnk1 | 0161677 | 0270611 Up |
| Tbc1d32 | 0161738 | -032606 Down |
| Samd8 | 0161865 | -037394 Down |
| Syn3 | 0162076 | -042357 Down |
| Dapk1 | 0162077 | -031025 Down |

| Clock | 0162111 | -040652 Down |
| --- | --- | --- |
| Adss | 0162225 | 040539 Up |
| Aftph | 0162615 | 026156 Up |
| Rpl11 | 0162757 | 0194727 Up |
| Ergic1 | 0162832 | -068244 Down |
| Hnrnph2 | 0162958 | 062762 Up |
| Adarb2 | 016316 | -087053 Down |
| Luc7l2 | 016325 | 0175956 Up |
| Sipa1l2 | 0163575 | -06846 Down |
| Ctnnbl1 | 0164007 | -072306 Down |
| Rsf1 | 0164205 | -022917 Down |
| Pgls | 0164339 | 0292069 Up |
| Arl6ip5 | 0164522 | 0550747 Up |
| Cd180 | 0164625 | -026211 Down |
| Sdhb | 0165273 | 0192235 Up |
| Pik3c2b | 0165752 | -060367 Down |
| Vps29 | 0165855 | 033531 Up |
| Cblb | 0166033 | 0469831 Up |
| Dmxl2 | 0166232 | 057233 Up |
| Scai | 0166254 | 0718172 Up |
| Nsf | 0166281 | 0554847 Up |
| Elf1 | 0166344 | -023223 Down |
| Rnf187 | 0166594 | 074291 Up |
| Trrap | 0166824 | -065645 Down |
| Gnai2 | 0166903 | -032552 Down |
| Adam19 | 0167129 | 0633756 Up |
| Tnks | 0167201 | 0382838 Up |
| Naa20 | 016722 | -037289 Down |
| Dnmt1 | 0167276 | -05056 Down |
| Emc10 | 0167672 | 0564172 Up |
| Oip5os1 | 0167939 | 0567768 Up |
| Cpne3 | 0167978 | -060459 Down |
| Abl1 | 0168318 | -068518 Down |
| Dock8 | 0169174 | -043839 Down |
| Afdn | 0169203 | -046421 Down |
| Fmn1 | 0169333 | -039463 Down |
| Adam17 | 0169382 | -061953 Down |
| Rgl1 | 0169406 | -037339 Down |
| Dennd5b | 0169528 | 0658689 Up |
| Gapdh | 0169602 | 033437 Up |
| Nedd4l | 0169971 | -043955 Down |
| Cdc42se2 | 0170031 | 040676 Up |
| Msl2 | 0170215 | -047958 Down |
| Tnik | 0170241 | 0173194 Up |
| Wdr33 | 0170285 | 0170379 Up |
| Kpnb1 | 017054 | 0164949 Up |
| Tnrc18 | 0170747 | -036275 Down |
| Grid1 | 0171022 | -06591 Down |
| Dnajc1 | 0171108 | -019229 Down |
| Atp6v1h | 0171123 | 0274995 Up |

| Tjp1 | 0171279 | -072123 Down |
| --- | --- | --- |
| Senp6 | 0171312 | 0238245 Up |
| Ddi2 | 0171341 | -02457 Down |
| Sec24a | 0171355 | 0441728 Up |
| Acvr1 | 0171524 | -053464 Down |
| Pou2f2 | 0171534 | -040486 Down |
| Rbm41 | 0171911 | -102523 Down |
| Tbc1d1 | 0172077 | -063723 Down |
| Cep164 | 01726 | -062938 Down |
| Il1rapl1 | 0172625 | -024689 Down |
| Dusp1 | 0172878 | 0823282 Up |
| Atp6ap2 | 0172966 | 047547 Up |
| Apobec3 | 0173311 | -025282 Down |
| Tln1 | 017338 | -046657 Down |
| Mapre2 | 0174015 | 0428975 Up |
| Pde1b | 0174162 | -064912 Down |
| Ttyh3 | 017418 | -051233 Down |
| Nudt21 | 0174244 | 0830615 Up |
| Ehd4 | 0174276 | 0292028 Up |
| Cabin1 | 0174358 | -039516 Down |
| Ttc7 | 0174523 | 0462822 Up |
| 2410006H1 | 0174774 | 0260932 Up |
| Slc12a2 | 0174872 | -071405 Down |
| Mia2 | 0175024 | 0197456 Up |
| Mllt11 | 0175149 | 0474017 Up |
| Zup1 | 0175186 | 0356843 Up |
| Rps27a | 0175258 | 0153528 Up |
| Brf1 | 0175346 | -02877 Down |
| Add2 | 0175538 | -057702 Down |
| Ola1 | 0175639 | 0328747 Up |
| Mob1a | 0175734 | -061595 Down |
| Ip6k2 | 0175906 | 0546617 Up |
| Zfp292 | 0176117 | -039794 Down |
| Arid3b | 0176359 | -037302 Down |
| Tgfb1 | 0176366 | -071317 Down |
| Ptpn9 | 0176832 | 0655645 Up |
| Slit2 | 0177244 | -05885 Down |
| Map3k8 | 017775 | 0411303 Up |
| Myh9 | 017802 | -019852 Down |
| Abcd4 | 017891 | -051758 Down |
| Ndufb1-ps | 0178971 | 0534862 Up |
| Erc1 | 017904 | -0488 Down |
| Nsun6 | 0179159 | -052423 Down |
| B3glct | 0179458 | 0644201 Up |
| Abraxas2 | 0179726 | 074252 Up |
| Rps24 | 0179755 | 0305039 Up |
| Smim4 | 0179884 | 0615501 Up |
| Pcsk7 | 0180105 | -060713 Down |
| Tmem87b | 0180208 | 0333255 Up |
| Caap1 | 0180282 | -056266 Down |

| Zfp655 | 0180343 | -044592 Down |
| --- | --- | --- |
| Wasf2 | 0180362 | 0332565 Up |
| Mtus1 | 0180446 | -045738 Down |
| Hmgn2 | 0180915 | 0221554 Up |
| Atp1b3 | 0181061 | -046808 Down |
| Atp13a2 | 0181265 | -075009 Down |
| Bbs9 | 0181662 | -015534 Down |
| Ccdc12 | 0181895 | -055707 Down |
| Tmem50a | 0182015 | 0359697 Up |
| Hs3st5 | 0182129 | -0382 Down |
| Spcs3 | 018231 | 0274379 Up |
| Ccdc59 | 0182414 | -033005 Down |
| Add1 | 018246 | -039103 Down |
| Evi5l | 0182505 | -056596 Down |
| Gm4951 | 0182536 | 0338573 Up |
| Ncoa7 | 0182562 | 0553744 Up |
| Syngr1 | 0182906 | -051007 Down |
| Pnpt1 | 018304 | -066856 Down |
| Auts2 | 0183157 | -023738 Down |
| Hk2 | 0183774 | -03852 Down |
| Phf8 | 0184027 | -093903 Down |
| Itprid2 | 0184255 | -043358 Down |
| Cdh8 | 018464 | -062871 Down |
| Spats2l | 0184642 | -070444 Down |
| Tenm4 | 0184881 | -072724 Down |
| Itgb5 | 0185263 | -041692 Down |
| Tax1bp1 | 0185295 | -048043 Down |
| Eps8 | 0185339 | 0349808 Up |
| Thoc2 | 0185396 | 0355074 Up |
| Zfp516 | 0185454 | 0359891 Up |
| Zfp598 | 0185828 | -09874 Down |
| Dclk2 | 0185863 | -055521 Down |
| Smarca5 | 0186484 | -038349 Down |
| Stmn1 | 0186536 | 0354094 Up |
| Glul | 0186628 | 0282949 Up |
| Atp9a | 0186789 | 0910778 Up |
| Sh3pxd2a | 018688 | -038337 Down |
| Glb1 | 0187766 | 0545172 Up |
| Samm50 | 0187881 | 0211631 Up |
| Rab22a | 0188054 | -052551 Down |
| Cnksr2 | 0188306 | -038624 Down |
| Nr2c2 | 0188464 | -025336 Down |
| Grin2a | 0188496 | -073817 Down |
| Txndc11 | 0188521 | 0471407 Up |
| Ubash3b | 0188583 | -028093 Down |
| Cln3 | 018862 | -028143 Down |
| Atp5k | 0188746 | 0522866 Up |
| 2810403D2 | 0188755 | 0525255 Up |
| Rhoq | 018889 | 069257 Up |
| Suz12 | 0189076 | -045717 Down |

| Chchd2 | 0189438 | 0358456 Up |
| --- | --- | --- |
| Gigyf2 | 0190115 | 0259294 Up |
| Ppt1 | 0190246 | -043198 Down |
| Ttc13 | 0190671 | 0723197 Up |
| Atp2a2 | 0190968 | 0162903 Up |
| Zfp846 | 0191138 | -029907 Down |
| Caln1 | 0191153 | -034065 Down |
| Smad7 | 0191218 | -080698 Down |
| 2900097C1 | 019173 | -076823 Down |
| Ago4 | 0192281 | 0543451 Up |
| Wdr75 | 0192526 | 0361873 Up |
| Tnfrsf21 | 0192822 | -052807 Down |
| Stim1 | 0193019 | -026859 Down |
| Lpp | 0193388 | 01656 Up |
| Sobp | 019339 | -046376 Down |
| Atp6v0e | 0193794 | 0544979 Up |
| Nsg2 | 0193969 | -036956 Down |
| Hbs1l | 0194143 | 0250911 Up |
| Daglb | 0194261 | 0485371 Up |
| Tead1 | 0194458 | -021165 Down |
| Dennd2a | 0194975 | -055046 Down |
| Eml5 | 0195197 | -063731 Down |
| Nkain2 | 0195346 | -016727 Down |
| Nsun2 | 0196123 | -111859 Down |
| Slc25a46 | 0196198 | 0920578 Up |
| Capzb | 0196306 | -035762 Down |
| Spi1 | 0196365 | -031268 Down |
| Atxn7 | 0196527 | 0280875 Up |
| Rusc2 | 0197043 | 0489538 Up |
| Gga3 | 0197851 | -069528 Down |
| Polr2a | 0197879 | -056875 Down |
| Maco1 | 0197981 | -023105 Down |
| Med13 | 0198299 | 0345068 Up |
| Nr2f1 | 0198555 | -11745 Down |
| Eif3m | 0199015 | -086498 Down |
| Ap2b1 | 0199677 | 0315516 Up |
| Prpf40a | 0200248 | -020276 Down |
| Msh3 | 0200479 | -028341 Down |
| Glg1 | 0201075 | 0256622 Up |
| Zfp950 | 0201184 | 0379846 Up |
| Ssh2 | 0201318 | 0252945 Up |
| Cndp2 | 0201323 | 0174257 Up |
| P3h2 | 020189 | 0811436 Up |
| Slf2 | 0201987 | 0153642 Up |
| Nck1 | 0202064 | 0438404 Up |
| Smc3 | 0202249 | -038292 Down |
| Wac | 0202406 | 0249226 Up |
| A230057D | 0202587 | -044136 Down |
| Zbtb7a | 020259 | -065317 Down |
| Xpo1 | 0202707 | -037484 Down |

| Inpp5f | 0202851 | 0219111 Up |
| --- | --- | --- |
| Tmem106a | 0202851 | 0314071 Up |
| B3galnt1 | 0202905 | 0313741 Up |
| Bnip1 | 0203116 | 0289169 Up |
| Slx4ip | 020419 | 0323011 Up |
| Cnot3 | 0204397 | 04704 Up |
| Efhd2 | 0204397 | -074468 Down |
| Heatr5b | 0204449 | 0622866 Up |
| Svil | 0204861 | 0341208 Up |
| Fxr1 | 0205121 | -021779 Down |
| Atp13a3 | 0205702 | -022306 Down |
| Rbm5 | 0205886 | -028286 Down |
| Rundc3b | 0206287 | 1107821 Up |
| Immp2l | 0206347 | 0244757 Up |
| Nudt3 | 0206514 | -037013 Down |
| Cerk | 0206968 | -037782 Down |
| Cep83 | 0206994 | -048352 Down |
| Cep112 | 0207091 | -044943 Down |
| Dnmbp | 0207826 | 0469933 Up |
| Slc43a2 | 0207931 | 030977 Up |
| Zdhhc14 | 0208533 | -031106 Down |
| Ank | 020865 | -04377 Down |
| Epha7 | 0208653 | 0451616 Up |
| Srsf3 | 0208678 | -016879 Down |
| Nxf1 | 0209103 | -049193 Down |
| Ankmy2 | 0209421 | 025501 Up |
| Zdhhc20 | 0209688 | 029112 Up |
| Specc1l | 0209936 | 0376873 Up |
| Psma2 | 0209964 | -054527 Down |
| Zfyve16 | 0209989 | 0574729 Up |
| Lars2 | 0210265 | 0169889 Up |
| Mtpap | 0210288 | -070678 Down |
| Ppm1a | 0211073 | -041209 Down |
| Mcts1 | 0211734 | -048027 Down |
| Gm35188 | 0211816 | -073822 Down |
| Pdcd11 | 0211816 | -059434 Down |
| Lamtor3 | 0211817 | -070828 Down |
| Atp1a3 | 0212072 | -074856 Down |
| Hmgcl | 0212116 | 0507481 Up |
| Cdyl2 | 0212533 | -064581 Down |
| Fkbp8 | 0213508 | -040317 Down |
| Ythdc1 | 0213552 | 0285681 Up |
| Camk2g | 0213663 | 0327596 Up |
| Ndufa12 | 0213791 | 0162508 Up |
| Cpeb3 | 0214179 | 0304698 Up |
| Ogfrl1 | 0214505 | 0676141 Up |
| Ttc9c | 0214506 | 0486755 Up |
| Ifi30 | 0214621 | -060779 Down |
| Scfd2 | 0215293 | -067785 Down |
| 2510009E0 | 0215501 | 0422961 Up |

| Sh3bgrl3 | 0215627 | 0212186 Up |
| --- | --- | --- |
| Slc9a7 | 0215766 | -053569 Down |
| Gpx1 | 0215829 | 023369 Up |
| Tgs1 | 021587 | 04772 Up |
| Senp2 | 0216136 | -044148 Down |
| Rps26 | 0216241 | 0208664 Up |
| Grk2 | 0216344 | -04621 Down |
| Rgs7bp | 0216446 | 0218079 Up |
| Afg3l1 | 0216801 | 0542988 Up |
| Ccni | 0216823 | 0565657 Up |
| Eif2ak3 | 0216921 | 0984552 Up |
| Ppm1g | 0217214 | 0211512 Up |
| Rcor3 | 021731 | 0601403 Up |
| Hacd4 | 0217474 | 0268165 Up |
| Ube2o | 0217858 | -080799 Down |
| Rnf144b | 0217881 | -060608 Down |
| Sf3a1 | 0218174 | -07164 Down |
| Fam204a | 0218886 | -053806 Down |
| Caprin1 | 0219267 | -029598 Down |
| Rere | 0219363 | -019977 Down |
| Ahnak | 0219583 | 0184219 Up |
| Ccdc58 | 0219705 | 0349142 Up |
| Usp4 | 0219764 | 0291011 Up |
| Syne1 | 0219795 | -053382 Down |
| Bicral | 0220246 | -072986 Down |
| Ambra1 | 0220438 | -029316 Down |
| Usp50 | 0220757 | 0362643 Up |
| Mrpl41 | 022076 | -059988 Down |
| Mbtd1 | 0220867 | -021023 Down |
| Atf7 | 0221157 | -033734 Down |
| Slc22a23 | 0221288 | 0399704 Up |
| Dlgap2 | 0222143 | -024602 Down |
| Arrb2 | 0222412 | -054942 Down |
| Btg2 | 0222658 | 1170544 Up |
| Naa25 | 0222686 | -063214 Down |
| Hipk3 | 0222921 | -075574 Down |
| Rmnd5a | 0223055 | 0231389 Up |
| Trak1 | 0223088 | -030426 Down |
| Myo1f | 0223143 | -044553 Down |
| Dnajc10 | 0223232 | 0532407 Up |
| Actr1b | 0223317 | -037207 Down |
| Arap1 | 0223497 | -068342 Down |
| Zfp532 | 0223536 | -045136 Down |
| Sdf2 | 022378 | 0336841 Up |
| Neat1 | 0223846 | 0221231 Up |
| Coro1a | 0224213 | -06648 Down |
| Cnnm2 | 0224402 | -072 Down |
| Lrp6 | 0224797 | 0151383 Up |
| Rpl37 | 0225172 | 0167113 Up |
| Set | 0225234 | 0428503 Up |

| Zfp821 | 0225399 | -105662 Down |
| --- | --- | --- |
| Rspry1 | 0225773 | 0619743 Up |
| D930016D | 0225897 | 0446245 Up |
| Actr1a | 0226122 | 0700352 Up |
| Zfp810 | 0226367 | 0427559 Up |
| Arhgap15 | 0226819 | 0279393 Up |
| Gars | 0227067 | -086361 Down |
| Dgcr8 | 022754 | 0377235 Up |
| Prmt2 | 0227781 | -095142 Down |
| Comt | 0228633 | 0156784 Up |
| Vps26c | 0228814 | 0634092 Up |
| Ahctf1 | 0229117 | 0212494 Up |
| Spag9 | 022936 | 0272782 Up |
| Slc35c2 | 0229439 | -036845 Down |
| Ppp1cc | 022944 | 0663726 Up |
| Gm49797 | 0229453 | -061356 Down |
| Anks1b | 0230028 | -020004 Down |
| Ccnt1 | 0230114 | 0322963 Up |
| Blmh | 023028 | -0363 Down |
| Agbl4 | 02307 | -016125 Down |
| Nab1 | 0230953 | -044855 Down |
| Blvra | 0231244 | 0467956 Up |
| Ptn | 0231319 | -015346 Down |
| Tmem243 | 0231577 | 0299094 Up |
| Stxbp5 | 0231612 | 0284525 Up |
| Strada | 0231745 | -081372 Down |
| AW554918 | 0231875 | -028373 Down |
| Phf3 | 0232392 | 0203747 Up |
| Rcsd1 | 0232436 | 0306013 Up |
| 10-Sep | 023306 | -063661 Down |
| Trpm7 | 023353 | -018959 Down |
| Psmd13 | 0233643 | -030693 Down |
| Zfp322a | 0233682 | -106484 Down |
| Atp5a1 | 0234413 | 0218884 Up |
| Sptbn2 | 0234753 | 0236208 Up |
| Mical3 | 0235042 | -068382 Down |
| Als2 | 0235372 | -078679 Down |
| Ppm1b | 0235567 | 0317161 Up |
| Smg5 | 0235609 | -033865 Down |
| Rufy2 | 0235908 | 0472775 Up |
| Kcnq2 | 0236073 | -01892 Down |
| Zfp160 | 0236086 | 0277308 Up |
| Atp7a | 023631 | 0205528 Up |
| Naa30 | 0236318 | 0737347 Up |
| Pip4p1 | 0236465 | -039943 Down |
| Cnot4 | 023655 | -025111 Down |
| Tmlhe | 0236797 | 0542491 Up |
| Trim44 | 0236941 | -03652 Down |
| Gabrb2 | 0237446 | -032674 Down |
| Ddx5 | 0237678 | 0170687 Up |

| Idh1 | 0237693 | 044178 Up |
| --- | --- | --- |
| Smchd1 | 0238072 | -029321 Down |
| 7-Mar | 0238097 | 0383386 Up |
| Mtmr12 | 0238208 | 0637575 Up |
| Pds5a | 023828 | -018013 Down |
| Pmp22 | 023864 | 0475961 Up |
| Gapvd1 | 0238663 | -038286 Down |
| Cd37 | 0238787 | -038983 Down |
| Sqle | 0239001 | 0338573 Up |
| Kif3b | 0239518 | -030346 Down |
| Mgat4b | 0239547 | -037058 Down |
| Golm1 | 0239812 | -038214 Down |
| Klhl2 | 0239812 | -058588 Down |
| Zfp622 | 0239966 | 0281887 Up |
| Slf1 | 0240098 | -029885 Down |
| Rpl29 | 0240469 | -026665 Down |
| Cyren | 0240739 | 0490271 Up |
| Zcchc14 | 0240829 | 0610361 Up |
| Arf4 | 0241136 | 0281891 Up |
| Tmco1 | 02412 | -061705 Down |
| Fcrls | 0241341 | -023847 Down |
| Zfp148 | 0241735 | -027083 Down |
| Rps20 | 0241754 | 0211931 Up |
| Foxo3 | 0242019 | -037968 Down |
| Camk2b | 0242062 | -079493 Down |
| Rap1a | 0242078 | -027861 Down |
| Ecd | 0242145 | -025534 Down |
| Hdac2 | 024217 | -057692 Down |
| Gpr45 | 0242603 | -043969 Down |
| Tep1 | 0243211 | -021498 Down |
| Cebpg | 0243683 | 0408743 Up |
| Ddhd2 | 0244133 | -039805 Down |
| Ostm1 | 0244184 | 0706079 Up |
| E230029C0 | 0244898 | -073706 Down |
| Tab2 | 0244961 | 0464188 Up |
| Mcl1 | 024526 | 0254506 Up |
| Hspa4 | 0245843 | -045998 Down |
| Dhx8 | 0246184 | -031029 Down |
| Cep295 | 0246241 | -034753 Down |
| Rcor1 | 0246468 | -048928 Down |
| Dnaja2 | 0246505 | -101728 Down |
| Rps18 | 0246646 | 0165485 Up |
| Slc38a10 | 0247307 | -038491 Down |
| Zfp207 | 0247331 | -022162 Down |
| Zcchc7 | 0247452 | -025325 Down |
| Glmn | 0247524 | -028171 Down |
| Man1a2 | 0247907 | -023406 Down |
| Focad | 0248125 | -033597 Down |
| Cadps | 0248242 | -037093 Down |
| Pafah1b1 | 0248259 | -020328 Down |

| Egfem1 | 0248372 | 0466416 Up |
| --- | --- | --- |
| Vps18 | 0248572 | -049536 Down |
| D10Wsu10 | 0248644 | -038484 Down |
| Ctxn1 | 0248802 | 0543938 Up |
| Atpaf1 | 024881 | 0205084 Up |
| Gm3764 | 0249118 | -067842 Down |
| Hspd1 | 0249172 | 0403874 Up |
| Mpp5 | 0249364 | -038392 Down |
| Orc5 | 0249414 | 0537665 Up |
| Dad1 | 0249564 | -071291 Down |
| Aagab | 0249969 | -073248 Down |
| Pacs2 | 0250521 | 0549457 Up |
| Phf20 | 0250571 | 0263026 Up |
| Mrps9 | 0250742 | 0441656 Up |
| Wdfy1 | 025101 | -027849 Down |
| Asb7 | 0251143 | -052875 Down |
| Ubxn7 | 025123 | -062128 Down |
| Bcl9 | 025134 | -062478 Down |
| Zfp943 | 0251447 | 0555484 Up |
| Crlf3 | 025147 | 0277064 Up |
| Ptgs1 | 0252245 | -03747 Down |
| Map2k5 | 025234 | -038933 Down |
| Gpatch2l | 0252351 | 0256651 Up |
| Ndufaf2 | 0253179 | -019379 Down |
| Rab3c | 0253322 | -044789 Down |
| Rps3 | 0253585 | 0179202 Up |
| H3f3a | 0253585 | 0350291 Up |
| Srrm4 | 0253673 | -025064 Down |
| Pycard | 0253709 | -092868 Down |
| Edem3 | 0253863 | 0597099 Up |
| Akap7 | 0253975 | -06391 Down |
| Kirrel3 | 0254488 | -072348 Down |
| Nudcd3 | 0254605 | -020984 Down |
| Sox2ot | 0254976 | -042472 Down |
| Arhgef6 | 0255138 | 062695 Up |
| B830012L1 | 025536 | -01596 Down |
| Avl9 | 0255624 | 02568 Up |
| Nup205 | 0255672 | -058118 Down |
| Lrrfip2 | 0255735 | 0185329 Up |
| Clpx | 0256175 | 0154782 Up |
| Cox8a | 025619 | 0266214 Up |
| Sec31a | 0256331 | 0371507 Up |
| Dnaja1 | 0256658 | 0307169 Up |
| Sema6a | 0256909 | -057226 Down |
| Nucb1 | 0256948 | 0738903 Up |
| Srrm2 | 0257212 | -018936 Down |
| Arhgap19 | 0257482 | 0175385 Up |
| Rpgr | 0257847 | 0290547 Up |
| Gap43 | 0257872 | 0313091 Up |
| Camkmt | 0258665 | -044412 Down |

| Yars | 0258872 | -042822 Down |
| --- | --- | --- |
| Siglech | 0259058 | -038496 Down |
| Rbm28 | 0259285 | 053736 Up |
| 4932438A1 | 025931 | 0227074 Up |
| Tm9sf2 | 0259752 | -038522 Down |
| Tmem168 | 0259764 | 0421852 Up |
| 3110056K0 | 026018 | -039037 Down |
| Cep170 | 0261501 | 0161822 Up |
| Cdh4 | 0261762 | -06028 Down |
| Xrn1 | 0262055 | -03668 Down |
| Fubp1 | 0262085 | -028346 Down |
| Grip1 | 0262087 | -030113 Down |
| Slco3a1 | 026281 | -040552 Down |
| Fam168b | 0262946 | -046993 Down |
| Cryl1 | 0263846 | 0253537 Up |
| Phtf2 | 0264066 | 0534318 Up |
| Bclaf1 | 0264076 | 0164208 Up |
| 2210408I2 | 0265032 | -040885 Down |
| Ctsb | 0265323 | 0210393 Up |
| Rab40c | 0265387 | 0443092 Up |
| Sdha | 0265443 | 0300064 Up |
| Capza2 | 0265473 | -035976 Down |
| Mecp2 | 0265675 | 0327139 Up |
| Rp9 | 0266002 | -038088 Down |
| Lpgat1 | 0266255 | -041859 Down |
| Map7d1 | 0266464 | -032464 Down |
| Tmem260 | 026654 | 0402736 Up |
| Dcaf7 | 0266553 | -091808 Down |
| Lrp12 | 0266636 | -056197 Down |
| Rps6ka3 | 0266831 | -015597 Down |
| Cog5 | 0267032 | -021985 Down |
| Rala | 0267416 | 0275602 Up |
| Rac1 | 0267489 | -048681 Down |
| Canx | 0267628 | 0279314 Up |
| Itsn2 | 0267715 | 0294369 Up |
| Vav2 | 0267845 | -021784 Down |
| Cpt1a | 0267943 | -06444 Down |
| Ggps1 | 0267953 | 0255526 Up |
| Slc39a10 | 0268391 | -065356 Down |
| Peli1 | 0268395 | -020616 Down |
| Cnot2 | 0268424 | -032859 Down |
| Arhgef1 | 0268446 | 040147 Up |
| Nup155 | 0268497 | 0161456 Up |
| Dusp11 | 0268553 | 0438248 Up |
| Hmgxb3 | 0268584 | -064909 Down |
| Cfap20 | 026873 | 0224645 Up |
| Cul3 | 0268783 | -042025 Down |
| Ash2l | 0268794 | -043765 Down |
| Ndufa13 | 0268868 | 0483119 Up |
| Xkr4 | 0269056 | -021698 Down |

| Nme1 | 0269638 | 0499253 Up |
| --- | --- | --- |
| B3gntl1 | 0269738 | -049081 Down |
| Pfdn5 | 027006 | 025098 Up |
| Dvl1 | 0270172 | -064438 Down |
| Ulbp1 | 027022 | 0157131 Up |
| Psmb7 | 0270314 | 0261561 Up |
| Metap2 | 0270578 | -01704 Down |
| Emp3 | 0270809 | 0184084 Up |
| Unc93b1 | 0270864 | 0227498 Up |
| Wdr44 | 0270964 | -03027 Down |
| Nsmaf | 0271073 | -018094 Down |
| Rpl32 | 0271206 | 0286425 Up |
| Dock1 | 0271699 | 0276111 Up |
| Kctd12 | 027213 | -023938 Down |
| Fuca2 | 0272174 | -073023 Down |
| Adgre1 | 0272176 | -025189 Down |
| Zfp827 | 0272389 | -055745 Down |
| Bcl11a | 0272629 | -018954 Down |
| Pak1 | 0272824 | 0863829 Up |
| Tuba1a | 0273387 | 0351046 Up |
| Tiparp | 0273685 | 042574 Up |
| Snhg20 | 0274272 | -039048 Down |
| Ufsp2 | 0275015 | 0287748 Up |
| Arhgef10l | 0275133 | 0550844 Up |
| Adam33 | 0275213 | 0168507 Up |
| Metap1d | 0275253 | -049359 Down |
| Basp1 | 0275818 | -033004 Down |
| Mettl9 | 0276239 | 0414673 Up |
| Lrrcc1 | 0276422 | 057745 Up |
| Arglu1 | 0276536 | -021234 Down |
| Rpl7l1 | 0276801 | 0229404 Up |
| Fbxl3 | 0277046 | 0759712 Up |
| Syt14 | 0277519 | -061974 Down |
| Npepps | 0278092 | -038407 Down |
| Dot1l | 0278298 | -062804 Down |
| Atad2 | 0278512 | 018108 Up |
| Ephb1 | 0278812 | -04715 Down |
| Pde4dip | 0279003 | -027706 Down |
| Rab3il1 | 0279322 | -022712 Down |
| Hars | 0279963 | -033241 Down |
| Tmem14c | 0280076 | 0562396 Up |
| Pcyox1 | 0280404 | -043815 Down |
| Mapk9 | 028104 | -080178 Down |
| Zfp280c | 0281188 | 0275605 Up |
| Zfand5 | 0281269 | 0220062 Up |
| Txndc9 | 0281432 | 0409738 Up |
| Pdzd4 | 0281481 | -064931 Down |
| Ly6h | 0281664 | 0276996 Up |
| Ocrl | 0282001 | 0280332 Up |
| Polr1a | 0282005 | -033875 Down |

| Zfp236 | 0282265 | 0304345 Up |
| --- | --- | --- |
| Kcnq3 | 0282523 | -027029 Down |
| Hnrnpc | 0282537 | -037504 Down |
| Ggta1 | 0282629 | 0250308 Up |
| Stim2 | 0282827 | -05319 Down |
| Irf5 | 0283812 | 0259567 Up |
| Cmpk1 | 0283847 | -039948 Down |
| Csrnp3 | 0284045 | -045708 Down |
| Rnf103 | 0284326 | 0339746 Up |
| Ssr2 | 0284334 | 0286427 Up |
| Capn15 | 0284702 | -045203 Down |
| Mdm4 | 0285664 | -022128 Down |
| Fdft1 | 0285752 | 0735322 Up |
| Efl1 | 0285916 | -017993 Down |
| Usp22 | 0285995 | 0332202 Up |
| Cacna2d3 | 028639 | -032619 Down |
| Csmd3 | 0286425 | -030781 Down |
| Ccdc93 | 0286536 | -033502 Down |
| Hist1h1e | 0286883 | -022792 Down |
| Trim9 | 0286899 | -10305 Down |
| Sec11a | 0286966 | -022609 Down |
| Cdc37l1 | 028775 | -025092 Down |
| Strbp | 0288427 | -031431 Down |
| Gm9801 | 0288641 | 0586334 Up |
| Arhgap10 | 0288829 | 0545441 Up |
| Rnpepl1 | 0289452 | 0317271 Up |
| Chn2 | 028956 | -046944 Down |
| Kcnt2 | 0289694 | -016419 Down |
| Cse1l | 0290757 | -019672 Down |
| Rprd1a | 0290966 | 0377346 Up |
| Dnase2a | 0291212 | -043459 Down |
| Rpl36a | 0291351 | 0182056 Up |
| Stk24 | 0291411 | -052831 Down |
| Rmnd1 | 0291431 | -046358 Down |
| Cep162 | 029146 | -103737 Down |
| Ufl1 | 0291629 | 0466555 Up |
| Apba1 | 0291658 | -032674 Down |
| Ino80dos | 0291841 | 08871 Up |
| Acbd6 | 0291889 | -053984 Down |
| Yme1l1 | 0292257 | -030351 Down |
| Elovl6 | 0292301 | 0313755 Up |
| Ik | 0292515 | -017988 Down |
| Parn | 0292634 | -058958 Down |
| Ptprg | 0292906 | 032228 Up |
| Cep250 | 0293724 | -052221 Down |
| Scfd1 | 0293804 | -027716 Down |
| Hipk1 | 0293963 | 059288 Up |
| Tbl1x | 0294004 | 0220579 Up |
| Pgam1 | 0294321 | -065549 Down |
| Rpl35 | 0294513 | 0329697 Up |

| Glmp | 029469 | -027959 Down |
| --- | --- | --- |
| Xbp1 | 0294787 | 0263484 Up |
| Fbrsl1 | 029491 | -057905 Down |
| Tbcel | 0295152 | -053413 Down |
| Pwwp2a | 0295234 | -058214 Down |
| Mtx2 | 0295448 | 0622528 Up |
| Mapk3 | 0295658 | 0345911 Up |
| Ttc7b | 0296087 | -026784 Down |
| Foxp2 | 0296386 | 0509144 Up |
| Wdr36 | 0296476 | -043741 Down |
| Cpne8 | 0296525 | -064789 Down |
| Trim41 | 0296861 | 0410852 Up |
| Pik3cg | 0296874 | 0556725 Up |
| Nek7 | 0297141 | -030439 Down |
| Snx14 | 0297158 | -037814 Down |
| Gtf2i | 0297285 | -054288 Down |
| Bach1 | 0297393 | -04702 Down |
| Brd4 | 0297534 | -023297 Down |
| Pmepa1 | 029783 | -041895 Down |
| Slc16a7 | 0298419 | 030572 Up |
| Exoc1 | 0298898 | -068468 Down |
| Hecw1 | 0298919 | -029924 Down |
| Myo16 | 0298985 | -041999 Down |
| C130073E2 | 0299223 | -140166 Down |
| Dok6 | 0299509 | -047836 Down |
| Mndal | 030028 | 0371231 Up |
| Ivns1abp | 0300629 | -016422 Down |
| Hnrnpdl | 0301197 | -028856 Down |
| Rbbp6 | 0301366 | 0210005 Up |
| Fbxo33 | 0301492 | -057207 Down |
| Dph6 | 0301585 | -022905 Down |
| Grin2b | 0301848 | -023943 Down |
| Lrch1 | 0302064 | -022403 Down |
| Pdcd10 | 0302208 | 0349138 Up |
| Prkch | 0302259 | -028871 Down |
| Tubb2a | 0302324 | 0269959 Up |
| Phf21a | 0302442 | -02342 Down |
| Rc3h1 | 03026 | 0157022 Up |
| Chn1 | 0302689 | -048445 Down |
| Mtpn | 0302757 | -04872 Down |
| Ndfip1 | 0303414 | 0152029 Up |
| Uchl5 | 0303615 | -063325 Down |
| Szrd1 | 0304053 | -03508 Down |
| Pcmtd1 | 0304351 | 0223663 Up |
| Dtx3 | 0304886 | -036549 Down |
| Glis3 | 0305148 | 0191422 Up |
| Arfgap1 | 0305248 | -02732 Down |
| Fam222b | 0305557 | -015269 Down |
| Atp11a | 0305703 | 0306789 Up |
| Ddost | 0306091 | -058569 Down |

| Dnajc8 | 030623 | 0445308 Up |
| --- | --- | --- |
| Ube2e2 | 0306273 | 0193921 Up |
| Lipa | 0306431 | 026892 Up |
| Btk | 0306555 | -034788 Down |
| Atp5j | 0307192 | 0396316 Up |
| Hprt | 0307258 | 0419508 Up |
| Fip1l1 | 0307385 | -020572 Down |
| D5Ertd579 | 0307561 | -048029 Down |
| Acvr2a | 0307666 | 0332902 Up |
| Srgap3 | 0307694 | -040971 Down |
| Cops3 | 0308465 | 0278056 Up |
| Mef2d | 0308875 | -044444 Down |
| Ppp4c | 0309331 | 0432045 Up |
| Casp3 | 0309399 | 0450621 Up |
| Foxn3 | 0309681 | -027291 Down |
| Cdh12 | 0310492 | 0893553 Up |
| Fam126b | 0310969 | 0376699 Up |
| Tgfbr1 | 0311158 | -020756 Down |
| Scn8a | 0311203 | -031723 Down |
| Sgms1 | 0311268 | 0543576 Up |
| Map3k7 | 0311508 | -042735 Down |
| Agap3 | 0311783 | 0207386 Up |
| Dpysl3 | 0311961 | 0316102 Up |
| Sclt1 | 0311987 | 0249843 Up |
| Unc13b | 0311988 | -041026 Down |
| Dtnbp1 | 0312898 | -036921 Down |
| Gna13 | 0313687 | -017607 Down |
| Atg2b | 0314048 | -039695 Down |
| Nalcn | 0314149 | -052317 Down |
| Abce1 | 0314215 | -047945 Down |
| Scarb2 | 031439 | -0403 Down |
| Ube2h | 0314657 | 0480754 Up |
| Atp8a1 | 031488 | -022833 Down |
| Cacna1a | 03154 | -032422 Down |
| Ldha | 031543 | 0206439 Up |
| Zbtb18 | 0315454 | -041501 Down |
| H2-K1 | 031566 | -016372 Down |
| Bms1 | 0315697 | 0206565 Up |
| Rab11b | 0315757 | 040611 Up |
| Ttc28 | 0316113 | 015689 Up |
| Clstn1 | 0316376 | 0687202 Up |
| Smc4 | 0316789 | -074291 Down |
| Mcm3ap | 0316881 | 037034 Up |
| Aak1 | 0316952 | -035456 Down |
| P4ha1 | 0317588 | -037455 Down |
| Notch1 | 0318352 | 0184787 Up |
| Nup188 | 0318967 | 0523609 Up |
| Btbd7 | 0319356 | -024457 Down |
| Arhgap5 | 0319455 | -040879 Down |
| Mblac2 | 0319523 | -056737 Down |

| Cul9 | 0319999 | -072237 Down |
| --- | --- | --- |
| Tfe3 | 032197 | 0176633 Up |
| Rab14 | 0322059 | -01751 Down |
| D430041D | 0322121 | -070598 Down |
| Nop58 | 0322468 | -06978 Down |
| Atp1a1 | 0322633 | 0441708 Up |
| Fnbp4 | 0322735 | -023406 Down |
| Tbce | 0322943 | -046774 Down |
| Asic2 | 0323089 | -056069 Down |
| Lingo2 | 0323568 | -026456 Down |
| Ints3 | 0323754 | 0183353 Up |
| Nfx1 | 0324098 | -023562 Down |
| Tet3 | 03246 | -030269 Down |
| Gm1673 | 0325112 | 0292795 Up |
| Tbc1d19 | 0325199 | 0655076 Up |
| Csnk1g1 | 0325257 | 0211332 Up |
| Trim30a | 0325895 | 0223035 Up |
| Rpl7 | 0326295 | -045433 Down |
| Parp1 | 0326408 | 0228078 Up |
| Nono | 0326719 | 0363935 Up |
| Rsrc2 | 032673 | -038604 Down |
| Cnot6l | 0326748 | 0406815 Up |
| Myh10 | 0326769 | 0233594 Up |
| Ptpre | 0326791 | -055125 Down |
| Gdpd1 | 0326791 | 0432059 Up |
| Brd9 | 032703 | -034027 Down |
| Prpsap2 | 0327458 | -03815 Down |
| Lgals8 | 0328173 | -071551 Down |
| Map2k2 | 0328347 | 0178874 Up |
| Prkcd | 0329439 | -036351 Down |
| Dgkd | 0329547 | -019253 Down |
| Btf3l4 | 033074 | 0262454 Up |
| Hltf | 0331158 | -015388 Down |
| Rab28 | 0331195 | 027018 Up |
| Rhoa | 0331593 | -045196 Down |
| Kdm4b | 0331779 | -050632 Down |
| Hnrnpm | 0331801 | -026864 Down |
| Cdk6 | 0331829 | -035068 Down |
| Vps41 | 0332522 | -043057 Down |
| Gng12 | 0333301 | -028874 Down |
| Gabbr1 | 0333632 | -044289 Down |
| Pbx1 | 0334389 | -030868 Down |
| Usp40 | 0334765 | 0250813 Up |
| Rab21 | 0336035 | -055478 Down |
| Rap2a | 0337274 | -040342 Down |
| Pfkfb3 | 0337746 | -047953 Down |
| Irak1 | 0337898 | -033812 Down |
| Prmt1 | 0338269 | 0299412 Up |
| Tmeff1 | 0338297 | -018068 Down |
| Kif3c | 0339627 | -034496 Down |

| Mex3c | 0339627 | -036126 Down |
| --- | --- | --- |
| Vcpip1 | 0339761 | 0336405 Up |
| Cyth1 | 0339936 | 0270892 Up |
| Matr3 | 0339988 | 0522655 Up |
| 4632427E1 | 0340046 | -03912 Down |
| Grik2 | 0340055 | -046507 Down |
| Xpo4 | 0340068 | 0157089 Up |
| Abcg3 | 0340263 | -027043 Down |
| Tmem44 | 0340287 | -07486 Down |
| St7 | 0340372 | -040848 Down |
| Macrod2 | 034042 | 0335603 Up |
| Nbr1 | 0340602 | 0228291 Up |
| Aatf | 0340632 | -028356 Down |
| Tesk2 | 0340986 | 0423022 Up |
| Rnasel | 0341073 | 0220837 Up |
| Rae1 | 0341115 | -073223 Down |
| Man1c1 | 0341845 | -039347 Down |
| Pabpc1 | 0341891 | 0345916 Up |
| Dazap1 | 0342045 | 0276332 Up |
| Swap70 | 0343116 | 0298492 Up |
| Trappc4 | 0343131 | -021962 Down |
| Atp6v0a1 | 0343638 | -043851 Down |
| Dhdds | 0343837 | -068845 Down |
| Snapc3 | 0344103 | -029643 Down |
| Clec16a | 0344214 | 0201203 Up |
| Rnf13 | 034489 | 0337589 Up |
| Rpn1 | 0345149 | 0339583 Up |
| B2m | 0345385 | 0259687 Up |
| Sppl3 | 0345711 | -037904 Down |
| Ube2q2 | 0347089 | 0333795 Up |
| 2610020C0 | 0347191 | -023845 Down |
| Pum2 | 0347696 | -02397 Down |
| Tfrc | 0347787 | -101907 Down |
| Morc3 | 0348555 | -047313 Down |
| Cct6a | 034858 | -047164 Down |
| Amph | 0348783 | -033942 Down |
| Camsap2 | 0349364 | -021735 Down |
| Micu2 | 0349481 | 0308949 Up |
| Fam160b1 | 0349558 | 0513687 Up |
| Memo1 | 0349593 | 017281 Up |
| Arl5b | 0350135 | -049262 Down |
| Rbbp7 | 0350148 | -05283 Down |
| Prickle1 | 0350473 | -052283 Down |
| Grm5 | 0350534 | -031954 Down |
| Galnt17 | 0350734 | 0158311 Up |
| Hsd17b4 | 0350945 | -034723 Down |
| Cd99l2 | 0350964 | 0323935 Up |
| Ranbp2 | 0350973 | 0195262 Up |
| Dennd2c | 0351154 | -053998 Down |
| Fyb | 0351346 | -019352 Down |

| Rnf141 | 0351823 | 0594064 Up |
| --- | --- | --- |
| Tmeff2 | 0351912 | -03022 Down |
| Abhd17b | 0351991 | -034074 Down |
| Msra | 0352363 | -025404 Down |
| Nisch | 0353144 | 021287 Up |
| Hist1h4d | 0353602 | -057271 Down |
| Rab6b | 035368 | -054325 Down |
| Slc25a5 | 0353757 | -019874 Down |
| Slc35b1 | 0353864 | 0870765 Up |
| Slc46a3 | 0354542 | -040548 Down |
| Lamtor1 | 0354825 | 0173388 Up |
| Dop1b | 0354948 | -026771 Down |
| Elmo1 | 035512 | -01604 Down |
| Spcs1 | 0355273 | 0158002 Up |
| Ppp2r5e | 035541 | -03432 Down |
| Slc30a5 | 0355531 | -029888 Down |
| Zfp36l2 | 0355715 | 0172867 Up |
| Mgat4c | 0355946 | 0497841 Up |
| Tmem189 | 0356396 | -037901 Down |
| Ncstn | 0356413 | 0437191 Up |
| Rnf165 | 0356511 | -047246 Down |
| Plekhm2 | 0356688 | -037466 Down |
| Smad4 | 0356738 | -03797 Down |
| Mzt1 | 0356895 | -07982 Down |
| Asxl2 | 0357098 | -02097 Down |
| Stard7 | 0357685 | 0617899 Up |
| Hspa8 | 0357691 | -017983 Down |
| Zbtb41 | 0357771 | 0630654 Up |
| Sec11c | 0358049 | 0280812 Up |
| Ntrk2 | 0358176 | -02739 Down |
| Dhx32 | 035831 | 0286146 Up |
| Lnpep | 0358796 | 0247541 Up |
| Myef2 | 0358816 | 0306852 Up |
| Dpy19l1 | 0359484 | -038216 Down |
| Iqcb1 | 0359566 | -045177 Down |
| Rcbtb1 | 0359579 | 0299124 Up |
| Scamp1 | 0359648 | -067184 Down |
| Wipi2 | 0359726 | 0609207 Up |
| Pcnp | 0360242 | 0337658 Up |
| Id2 | 036044 | -015567 Down |
| Sys1 | 036049 | 0208129 Up |
| 1810026B0 | 0360786 | -021685 Down |
| Gabrb3 | 036117 | -039298 Down |
| Dock9 | 0361403 | 0175574 Up |
| Ubr4 | 0361637 | 029355 Up |
| Tspan5 | 0361675 | 0305219 Up |
| Mitf | 0361801 | -057014 Down |
| Rassf1 | 0361891 | -046815 Down |
| Qser1 | 0361956 | 0500393 Up |
| Uba6 | 0362339 | 0394922 Up |

| Csad | 0362519 | 0194069 Up |
| --- | --- | --- |
| Exosc10 | 0362643 | -069725 Down |
| Spata5 | 0362653 | -031914 Down |
| Tmem223 | 0362755 | -046389 Down |
| Clip2 | 0363115 | 0268131 Up |
| Atl1 | 0363174 | -039172 Down |
| Cherp | 0363281 | 0555947 Up |
| Cic | 0363963 | -063843 Down |
| Snx9 | 0364029 | -053477 Down |
| Arhgap32 | 0364111 | -030011 Down |
| Fgfr1 | 03643 | 0183721 Up |
| Rb1cc1 | 0364447 | 0185523 Up |
| Ranbp10 | 0364662 | 0632566 Up |
| Cdc5l | 0364755 | -056646 Down |
| Sorbs1 | 0364957 | -045359 Down |
| Ppp1r10 | 0365055 | 0478077 Up |
| Nol4l | 0365086 | -023748 Down |
| Tcirg1 | 0365087 | -037441 Down |
| Klhdc2 | 0365157 | 0682923 Up |
| Chrm3 | 0365208 | -047991 Down |
| Pgd | 036539 | 0467053 Up |
| Derl1 | 0365458 | 0581449 Up |
| Arl3 | 0366284 | -023624 Down |
| Krcc1 | 0366882 | 0205483 Up |
| Micos10 | 0367364 | -077177 Down |
| Tapt1 | 0367428 | -051119 Down |
| Nck2 | 0367709 | 0322228 Up |
| Lcp1 | 0368278 | 040164 Up |
| Schip1 | 0368633 | -110123 Down |
| Tusc3 | 0368675 | -034817 Down |
| Rapgef5 | 0368807 | -027549 Down |
| Pigu | 0368928 | -066873 Down |
| Fam117b | 0369327 | 024347 Up |
| Dpp10 | 0369428 | -026352 Down |
| Hmgn1 | 0369953 | -096173 Down |
| Anks1 | 0370299 | 0151041 Up |
| Pofut1 | 0370535 | -06424 Down |
| Qrich1 | 0370585 | -040415 Down |
| Tfg | 037073 | 0239009 Up |
| Vcan | 037077 | -050815 Down |
| Ep400 | 0371025 | -036497 Down |
| P4hb | 037112 | -045953 Down |
| Bbip1 | 037112 | -047623 Down |
| Rorb | 0371425 | 0213548 Up |
| Ckap5 | 0371993 | -029604 Down |
| Rfx3 | 0372498 | 0150784 Up |
| Tasor2 | 0372828 | -039098 Down |
| Apba2 | 0373417 | -050521 Down |
| Saraf | 0373525 | -052126 Down |
| Csf2ra | 037354 | -027667 Down |

| Sugp2 | 0373747 | -048177 Down |
| --- | --- | --- |
| Dhx57 | 0373773 | 0244086 Up |
| Wdr70 | 0374022 | -034026 Down |
| Sacm1l | 0374051 | 0156991 Up |
| Coro7 | 0374298 | 0162343 Up |
| Ppp2r5c | 0374617 | -018113 Down |
| Ccdc85a | 0374872 | -066598 Down |
| Clcn7 | 0374966 | -029128 Down |
| Dpy19l4 | 0375057 | -036792 Down |
| Nlk | 0375729 | -024344 Down |
| Ppp4r1 | 0376027 | -022838 Down |
| Atraid | 0376075 | -03961 Down |
| Knop1 | 0376345 | 0308764 Up |
| Nmnat2 | 0376415 | 0206826 Up |
| Timm9 | 03765 | 0488865 Up |
| Tk2 | 0377251 | 0206578 Up |
| Nampt | 0377502 | 0503392 Up |
| Cit | 0377734 | 0164482 Up |
| Ilf3 | 0377988 | -025731 Down |
| Fgd2 | 0378196 | -03761 Down |
| Kat7 | 0378724 | -019265 Down |
| Washc2 | 0378842 | 0312088 Up |
| Tafa1 | 0378986 | -075679 Down |
| Fam193b | 0379265 | 0813729 Up |
| Tmem108 | 0379282 | -020954 Down |
| Cct5 | 0379659 | -028092 Down |
| Tmem63b | 0379801 | -032186 Down |
| Hspa9 | 0379816 | -040174 Down |
| Scaper | 0380079 | -038098 Down |
| Cep120 | 0380087 | -045139 Down |
| Gucy1a2 | 0380784 | -024396 Down |
| Snhg3 | 0381017 | 0511669 Up |
| Parp11 | 0381017 | 0399726 Up |
| Rbm3 | 0381246 | 026252 Up |
| Pspc1 | 0381449 | -018268 Down |
| Mat2b | 038161 | 0282931 Up |
| Lrrk1 | 0381955 | -035868 Down |
| Gm1976 | 0382246 | -048654 Down |
| Fbxo7 | 0382935 | -020942 Down |
| Erh | 0383132 | -057881 Down |
| Preb | 0383237 | -060342 Down |
| Snx4 | 0383796 | 0883385 Up |
| Phf20l1 | 0384044 | 0263397 Up |
| Ndufs3 | 0384551 | 0155752 Up |
| Vps35 | 0384646 | 0170161 Up |
| Atad2b | 0384714 | -046325 Down |
| Ddx18 | 0385438 | 0435095 Up |
| Pld4 | 0386851 | -022404 Down |
| Lgals9 | 0387074 | 0269005 Up |
| Plaa | 0387175 | -048983 Down |

| Klf2 | 0387524 | 0306503 Up |
| --- | --- | --- |
| Atp6v1e1 | 0388429 | -038245 Down |
| Gm26749 | 0388678 | 0172246 Up |
| Pcbp2 | 0388736 | -037845 Down |
| Anp32b | 0388994 | 031972 Up |
| Armh3 | 0389278 | -020618 Down |
| Ppp2ca | 0389586 | 0170457 Up |
| Fabp5 | 0389629 | 0282904 Up |
| Gm49678 | 0389923 | 025901 Up |
| Ptpn4 | 0389956 | -01716 Down |
| Gpr158 | 0390445 | -022637 Down |
| Ptpa | 0390699 | -079531 Down |
| Dlc1 | 039088 | -039018 Down |
| Anp32a | 0391049 | 0382425 Up |
| Nfyc | 0391066 | -042365 Down |
| Cnot1 | 0392067 | -025274 Down |
| Elf2 | 0392325 | -033832 Down |
| Rab11fip2 | 0392939 | -018128 Down |
| Dctn4 | 0393039 | -031884 Down |
| Ntpcr | 0393048 | 0207033 Up |
| Smg1 | 0393171 | -026153 Down |
| Cdipt | 0393474 | 0403727 Up |
| Srsf11 | 0394042 | -023452 Down |
| Eif4ebp2 | 039444 | 044048 Up |
| Clk2 | 0394602 | 0506991 Up |
| Guf1 | 0394758 | -023818 Down |
| Trappc11 | 0394758 | 030084 Up |
| Taf1d | 0394767 | -035709 Down |
| Uba1 | 0395128 | 0443545 Up |
| Phka1 | 0395131 | 0164625 Up |
| Tecpr1 | 0396293 | -063325 Down |
| Magi3 | 03965 | -018932 Down |
| Prdx5 | 039686 | -044215 Down |
| Ckb | 0397697 | -025899 Down |
| Fam120c | 0397988 | -044732 Down |
| Setbp1 | 0398801 | -021408 Down |
| Carmil1 | 0399197 | -055856 Down |
| Dock7 | 0399229 | -015099 Down |
| Zfp652 | 0399811 | -019137 Down |
| Smarcad1 | 0399935 | -034909 Down |
| Pcdh7 | 0399935 | -034635 Down |
| Mpv17l | 0400117 | 0553022 Up |
| Ubqln1 | 0400907 | 0452695 Up |
| Sucla2 | 0401169 | 041544 Up |
| Chfr | 0401313 | 0233316 Up |
| Ammecr1l | 0401965 | 0269015 Up |
| Elp4 | 0401973 | 0402214 Up |
| Esyt2 | 040199 | -022934 Down |
| Cd164 | 0402058 | 0701988 Up |
| Pitpna | 0402547 | 0356392 Up |

| Ilf2 | 0402745 | -087384 Down |
| --- | --- | --- |
| Cdk7 | 040285 | -029536 Down |
| Slc23a2 | 0402926 | -028861 Down |
| Atxn10 | 0402926 | -025887 Down |
| Hcn1 | 0403128 | -037473 Down |
| Tbc1d24 | 0403167 | 0251096 Up |
| Entpd1 | 0403374 | -035016 Down |
| Tbcb | 0403436 | 0584702 Up |
| Arhgap25 | 0403658 | -031501 Down |
| Top1 | 0403837 | -033005 Down |
| Zfp592 | 0404085 | 034706 Up |
| Gm49336 | 040425 | -018945 Down |
| Nhlrc3 | 0404532 | 022748 Up |
| Trip4 | 0404532 | -039302 Down |
| Ick | 0404676 | 0532838 Up |
| Nfib | 0405321 | -053367 Down |
| Glcci1 | 0405334 | 0163462 Up |
| Cand1 | 0405688 | 0277145 Up |
| Mllt10 | 0405767 | 0184693 Up |
| Trio | 0405862 | -015148 Down |
| Farsb | 040638 | -045612 Down |
| Timm23 | 0406443 | -074264 Down |
| Med23 | 0406501 | -025515 Down |
| Dcun1d1 | 0406713 | -036841 Down |
| Hk3 | 0407074 | -031871 Down |
| R3hdm4 | 0407318 | 0568348 Up |
| Atad5 | 0407318 | 0306974 Up |
| Arhgap39 | 0407725 | -024562 Down |
| Arid2 | 0407865 | -031956 Down |
| Acp2 | 0408088 | -055909 Down |
| Brk1 | 040838 | -035352 Down |
| Braf | 0408419 | -034462 Down |
| Stk38 | 0409268 | -033224 Down |
| Smad1 | 0409465 | -035371 Down |
| Zfand3 | 0409896 | -025509 Down |
| Kcnq5 | 0410026 | -020089 Down |
| Edf1 | 0410053 | 0316435 Up |
| Ndst1 | 0410525 | 0564613 Up |
| Galnt10 | 0410571 | 0202284 Up |
| Zfp90 | 0411011 | 025618 Up |
| Srsf10 | 0411377 | 0287943 Up |
| Gpr34 | 041143 | -019856 Down |
| Scamp5 | 0411454 | 0154065 Up |
| Slc38a2 | 0412169 | -018715 Down |
| Sh3bgrl | 0412647 | -01675 Down |
| Osbpl1a | 0412756 | -03582 Down |
| Cpeb4 | 0412996 | -021459 Down |
| Pdk1 | 0413164 | 021972 Up |
| Epg5 | 0413299 | -027869 Down |
| Rnf214 | 0413422 | 0181476 Up |

| Hps5 | 0413523 | 0422532 Up |
| --- | --- | --- |
| Polr3b | 0413689 | 019176 Up |
| Rin2 | 0414321 | 0161288 Up |
| Retreg1 | 0414594 | -052343 Down |
| Crebrf | 0414642 | 0216751 Up |
| Atrn | 041495 | 0320444 Up |
| Hnrnpd | 0415157 | -0273 Down |
| Tpr | 0415167 | -020606 Down |
| Ndufb11 | 0415168 | 0506302 Up |
| Ralgapb | 041569 | -017388 Down |
| Trappc9 | 0415783 | 018893 Up |
| Sh3rf3 | 0415804 | -031235 Down |
| Fam168a | 0415983 | 0248065 Up |
| Pptc7 | 0415993 | 0291553 Up |
| Sap130 | 0416232 | 0364464 Up |
| St8sia2 | 0416286 | -018365 Down |
| Kcnd3 | 04164 | -045323 Down |
| Cspg5 | 0416724 | 0278105 Up |
| Grik1 | 0417225 | 0838471 Up |
| Pik3cd | 0417726 | -036233 Down |
| Ssr4 | 0417788 | -036078 Down |
| Sorbs2 | 0417907 | -01901 Down |
| Bin1 | 0418237 | -040137 Down |
| Lemd3 | 0418443 | -028544 Down |
| Casc4 | 0418698 | -019725 Down |
| Mrpl20 | 0418908 | 0608777 Up |
| Mknk1 | 0419206 | -046436 Down |
| Ptk2 | 0419291 | 0213323 Up |
| Fmnl2 | 0419482 | -032438 Down |
| Stk39 | 0419616 | 0164464 Up |
| Tmem33 | 0419782 | 032114 Up |
| Miat | 0420154 | -056035 Down |
| Dync1li1 | 0420197 | 0162351 Up |
| Nvl | 0420641 | -038759 Down |
| Luc7l | 0420719 | -020179 Down |
| Hnrnph3 | 0420982 | 0196857 Up |
| Eprs | 0421279 | -034052 Down |
| Zdhhc17 | 0421643 | 0159337 Up |
| Fra10ac1 | 0422778 | -017468 Down |
| Zmiz1 | 0423064 | -015165 Down |
| Gabra2 | 0423098 | 0805488 Up |
| Stx8 | 0424234 | 0203254 Up |
| Osbp | 0424299 | -058323 Down |
| Tmem165 | 0424592 | 0357773 Up |
| Rb1 | 0424659 | -017839 Down |
| Pdpk1 | 0424776 | 0260401 Up |
| Arrb1 | 0425483 | 05703 Up |
| Flii | 0425535 | -05608 Down |
| Plppr4 | 0425634 | -03035 Down |
| Ust | 0425885 | -033116 Down |

| Mark3 | 0425931 | 0171069 Up |
| --- | --- | --- |
| Abhd12 | 0426062 | -049767 Down |
| Ube2d3 | 0427376 | -030265 Down |
| Ripk2 | 042853 | -059591 Down |
| Oxct1 | 0428558 | -019436 Down |
| Uhrf1bp1l | 0428723 | -056662 Down |
| Mnat1 | 042936 | -015312 Down |
| Lrrc8d | 0429477 | -023769 Down |
| Hcfc2 | 0429683 | 0425409 Up |
| Depdc5 | 0430096 | 0269781 Up |
| Slmap | 0430127 | -031532 Down |
| Ppip5k2 | 0430145 | 018774 Up |
| Zfp282 | 0430584 | -049115 Down |
| Cops8 | 0430625 | 0314005 Up |
| Kansl3 | 04308 | 0575427 Up |
| Tubb3 | 0430912 | 042609 Up |
| Ptpro | 043102 | 0189114 Up |
| Wtap | 0431056 | 0446652 Up |
| Psmc3 | 04323 | 0210114 Up |
| Adnp | 043264 | -044712 Down |
| Ap3s2 | 0432775 | 0783649 Up |
| Pgap1 | 0432954 | 0157157 Up |
| Eif3f | 0432971 | 0442052 Up |
| Eml4 | 043301 | -015337 Down |
| Nfam1 | 0433632 | 0511731 Up |
| Ddx24 | 0434027 | -031313 Down |
| Banp | 0434067 | -031852 Down |
| Myl6 | 0435559 | 0176528 Up |
| Arhgef12 | 0435791 | 029298 Up |
| Ascc3 | 0435901 | -026508 Down |
| Rabggtb | 0435963 | -07189 Down |
| Vac14 | 043616 | -044789 Down |
| Tbc1d5 | 0436946 | 015727 Up |
| Lrpap1 | 0436977 | 045809 Up |
| St6galnac3 | 0437112 | -03929 Down |
| Ttc19 | 0437217 | -034853 Down |
| Zfp704 | 0437237 | -020404 Down |
| Dgkz | 043777 | -021515 Down |
| Rpl31 | 0438152 | 019891 Up |
| Pdss2 | 0438904 | 0272354 Up |
| L1cam | 043922 | -073861 Down |
| Tle4 | 0439362 | -016839 Down |
| Plin2 | 0439457 | -0387 Down |
| Jarid2 | 0439617 | -015568 Down |
| Cggbp1 | 0440078 | -084618 Down |
| Nfkbiz | 0440388 | -021015 Down |
| Trmt13 | 0440418 | -052435 Down |
| Tom1l2 | 0440471 | 0418176 Up |
| Cox5a | 0441287 | 0308568 Up |
| Nup214 | 044137 | 0533062 Up |

| Klhl6 | 0441546 | -030078 Down |
| --- | --- | --- |
| Nckap1 | 0442019 | -036644 Down |
| Rangap1 | 0442276 | -03222 Down |
| Trim2 | 0442445 | -049104 Down |
| Pclo | 0442496 | -020677 Down |
| Rnasek | 0442689 | 0340242 Up |
| Ddx21 | 0442689 | -067584 Down |
| Tmtc3 | 0443679 | 0506395 Up |
| Fbxo11 | 0443722 | -026051 Down |
| Cul1 | 0444065 | -024352 Down |
| Dnajc19 | 0444096 | 0215426 Up |
| Xylt1 | 0444096 | -053466 Down |
| Zfp81 | 0444601 | 0449151 Up |
| Csf1r | 0444795 | -01551 Down |
| Asxl1 | 0445886 | -026899 Down |
| Prpf8 | 0446193 | -04424 Down |
| Abcg1 | 0446238 | -091215 Down |
| Vsir | 044624 | -055487 Down |
| Smc5 | 0446315 | 0256966 Up |
| 4930444A1 | 044651 | 0332206 Up |
| Dpm1 | 044696 | -042045 Down |
| Itga4 | 0447265 | 022937 Up |
| Rbm27 | 0447333 | -018492 Down |
| Zbtb38 | 0447734 | -031846 Down |
| Cyth3 | 0447781 | -026907 Down |
| Atf2 | 0448156 | -031527 Down |
| Wdfy4 | 04486 | -05209 Down |
| Snta1 | 0448608 | -035339 Down |
| Dtx4 | 0448923 | -024102 Down |
| Rnf213 | 0449004 | -02709 Down |
| Hnrnpa3 | 044916 | -02255 Down |
| Pou6f1 | 0449435 | -024412 Down |
| Selenof | 0449526 | 0227473 Up |
| Rufy1 | 044967 | -039966 Down |
| Med15 | 0450013 | -021798 Down |
| Cct3 | 045031 | -031982 Down |
| Jpt1 | 045032 | 0279657 Up |
| Spp1 | 0450477 | 0551131 Up |
| Psmd11 | 0450792 | 0182779 Up |
| Bzw2 | 0450815 | -029473 Down |
| Ndfip2 | 0451151 | 0185696 Up |
| Tent4b | 045152 | -016432 Down |
| Rbfox1 | 0451729 | 01535 Up |
| Sertad2 | 0452102 | 0172662 Up |
| Kmt2c | 0452274 | -015316 Down |
| Zfp26 | 045231 | -047666 Down |
| Ptprm | 0452377 | 0714145 Up |
| Tbk1 | 0452455 | 0249411 Up |
| Gpr183 | 0453014 | -039381 Down |
| 5330438D1 | 0453916 | -035535 Down |

| Pyroxd2 | 0454356 | -023541 Down |
| --- | --- | --- |
| Rgmb | 0454692 | -045408 Down |
| Por | 0454918 | -024404 Down |
| Naa35 | 0455016 | -027437 Down |
| Srbd1 | 0455066 | -077676 Down |
| Thada | 0455335 | -028196 Down |
| Slc39a11 | 0455687 | -026492 Down |
| Trafd1 | 0455833 | -016502 Down |
| Fbxl2 | 0456084 | -033511 Down |
| Smarce1 | 045716 | 0283534 Up |
| Gpr137c | 0457191 | -02632 Down |
| Slc25a40 | 0457837 | -030687 Down |
| Tenm2 | 0457894 | 0245435 Up |
| Gatb | 0458111 | 04735 Up |
| Mcmbp | 0458142 | -047654 Down |
| Ndufs1 | 0458587 | -025196 Down |
| Acin1 | 0458762 | -034065 Down |
| Ndufb9 | 0460361 | 0190343 Up |
| Ubac2 | 0460434 | -016974 Down |
| Arid4b | 0461669 | -019228 Down |
| Synrg | 0461944 | -019397 Down |
| Enah | 0462671 | -027962 Down |
| Grcc10 | 0462758 | 044563 Up |
| Rps16 | 0462918 | 0197306 Up |
| Epn2 | 0464371 | -020385 Down |
| Arel1 | 0464598 | 0463202 Up |
| Jak2 | 0464693 | -02303 Down |
| Kcnb2 | 0466278 | -037865 Down |
| Tm9sf4 | 0466803 | -026107 Down |
| Gtf2ird1 | 0466803 | 0237905 Up |
| Traf3ip3 | 0466938 | 0319667 Up |
| Gtf2h5 | 0466938 | 0396824 Up |
| Acsl1 | 0466983 | -033309 Down |
| Stx12 | 0467315 | -02388 Down |
| Ptpn12 | 0467477 | 0163977 Up |
| Hira | 0467912 | -037121 Down |
| Trir | 046804 | 0274087 Up |
| Pfn1 | 0468051 | 0236893 Up |
| Fto | 0468104 | -030318 Down |
| Fsd1l | 0468485 | -034634 Down |
| Clic1 | 0468717 | -037326 Down |
| Gkap1 | 046878 | 0671567 Up |
| Clvs1 | 0469267 | 0976754 Up |
| Rims1 | 046935 | -01871 Down |
| Fyco1 | 0470232 | -027385 Down |
| Zswim5 | 0470468 | -046952 Down |
| Rbmxl1 | 0470491 | 038003 Up |
| Plgrkt | 0470797 | -026417 Down |
| Tcerg1 | 0471696 | -021019 Down |
| Gmcl1 | 0471819 | -016704 Down |

| Ints8 | 0471831 | -019677 Down |
| --- | --- | --- |
| Ccdc186 | 0472323 | -067731 Down |
| Tom1 | 047328 | -025093 Down |
| Trim33 | 0473868 | -019778 Down |
| Chtop | 0473911 | 0274797 Up |
| Mast2 | 0474747 | 0189049 Up |
| Fbxw11 | 0474922 | -025141 Down |
| Eps15l1 | 0475435 | -031506 Down |
| Naca | 0475632 | 0379083 Up |
| Hbp1 | 0475708 | 042843 Up |
| Chl1 | 0475936 | 0160928 Up |
| Cntn4 | 0477344 | 0261078 Up |
| Rragc | 0477593 | -026863 Down |
| Sf3b2 | 0477689 | -036159 Down |
| Dgki | 0477985 | -0245 Down |
| Trim8 | 0478859 | 0447181 Up |
| Ralyl | 0479086 | 0346769 Up |
| Lysmd4 | 0479433 | 03259 Up |
| Dclk1 | 0479794 | -022097 Down |
| Megf9 | 0480611 | 0154309 Up |
| Rasa2 | 0481166 | 0346461 Up |
| Ube2b | 0481298 | -019858 Down |
| Arl8a | 0481642 | 0311024 Up |
| Arl4c | 0481669 | -02897 Down |
| Chchd6 | 0482219 | 0728308 Up |
| Pxk | 0482681 | 0444285 Up |
| Ctsd | 0482781 | 016563 Up |
| Hipk2 | 0483285 | -035167 Down |
| Ftl1 | 0483301 | 0160625 Up |
| Rab11a | 0483568 | 0441797 Up |
| Ranbp9 | 0483957 | -023207 Down |
| Abi2 | 0484437 | 0156542 Up |
| Abca3 | 0484643 | -058793 Down |
| Appl1 | 0485058 | 0466925 Up |
| Edem1 | 0485166 | -042099 Down |
| Prkd3 | 0485222 | -021926 Down |
| Bcap29 | 048528 | 0537944 Up |
| Snap23 | 0485835 | -03327 Down |
| U2af2 | 048631 | 0246952 Up |
| Atrx | 0486818 | -021111 Down |
| Ptprj | 0486965 | -018336 Down |
| Fcgr3 | 0487098 | 0205834 Up |
| Pex14 | 0487188 | 0298846 Up |
| Lancl1 | 0487301 | -02357 Down |
| Akap9 | 0487524 | -027315 Down |
| Mindy3 | 0488077 | 0249606 Up |
| Dag1 | 0488137 | -018881 Down |
| Rassf3 | 0489016 | -075034 Down |
| Ctnnbip1 | 0489053 | -028921 Down |
| Git2 | 0489245 | 0161643 Up |

| Taf15 | 0489657 | -026295 Down |
| --- | --- | --- |
| Diaph3 | 0490039 | -016079 Down |
| Nedd9 | 0490406 | 0414246 Up |
| Tex10 | 0490639 | -019652 Down |
| Aldh2 | 0490938 | 0300792 Up |
| Med1 | 0491722 | 0186488 Up |
| Arih2 | 0491727 | -038893 Down |
| Chordc1 | 049214 | 0385891 Up |
| Dgcr2 | 0492214 | 0189076 Up |
| Gm37494 | 0492292 | 0347207 Up |
| Bri3 | 0492482 | -052226 Down |
| Tmem68 | 0492887 | -018696 Down |
| Ube2s | 0493202 | 0490498 Up |
| A830082K1 | 0493495 | 0416248 Up |
| Srcap | 0494151 | -021696 Down |
| Lmo4 | 04949 | 0571069 Up |
| Pura | 0495133 | -046979 Down |
| Arhgdia | 0495725 | 0289807 Up |
| Ilrun | 0495918 | -029903 Down |
| Scyl2 | 0495968 | 0381708 Up |
| Tubgcp5 | 0496005 | -022804 Down |
| Eif4b | 0496482 | -030354 Down |
| Cyba | 0496636 | 0152722 Up |
| Klhl7 | 0496726 | 0246033 Up |
| Csmd1 | 0496821 | -045928 Down |
| Cfl1 | 0497015 | 0167579 Up |
| Map3k5 | 0497076 | 0191989 Up |
| Kcnc2 | 0497166 | 0496474 Up |
| Nfia | 0497298 | -018813 Down |
| Cyfip2 | 0497344 | -048467 Down |
| Msh2 | 0497355 | -030553 Down |
| Tmem241 | 0497545 | 0245403 Up |
| Sfxn1 | 0497587 | -023842 Down |
| Usf3 | 0498667 | 0311239 Up |
| Smpd3 | 0498806 | 0574514 Up |
| Kif5c | 0498849 | 0194058 Up |
| Hdgfl3 | 0499285 | -033915 Down |
| Wdr45b | 0499569 | -017681 Down |
| Map4k3 | 0500543 | -025517 Down |
| Brd2 | 0501405 | -017219 Down |
| Platr25 | 0501706 | -033121 Down |
| Tsc22d1 | 0502046 | -029309 Down |
| Zmym2 | 0502262 | -028492 Down |
| C9orf72 | 050261 | -080473 Down |
| Npc1 | 0502692 | -047905 Down |
| Uhmk1 | 0502739 | 057236 Up |
| Naip5 | 0502824 | 0467105 Up |
| Ptprt | 0502827 | -028601 Down |
| Ndufaf7 | 0503213 | -038094 Down |
| Rabgap1l | 0503283 | 0400516 Up |

| Thra | 0503594 | 0263678 Up |
| --- | --- | --- |
| Malt1 | 0504721 | -019213 Down |
| Fam89b | 050491 | 0174358 Up |
| Map2k1 | 0505033 | 0214225 Up |
| Taf1 | 0505441 | 0386152 Up |
| Map3k3 | 0505584 | -023105 Down |
| Hmgxb4 | 0506214 | 0443478 Up |
| Rev1 | 0506665 | -028194 Down |
| Alkbh1 | 0507343 | -015955 Down |
| Tlr1 | 050774 | 0217214 Up |
| Ndufs7 | 050827 | 0208376 Up |
| Inpp5k | 0508315 | 0294364 Up |
| Agpat3 | 0508524 | 0249859 Up |
| Tnr | 0509188 | -04553 Down |
| Mrpl52 | 0509221 | -047919 Down |
| Nrd1 | 0509359 | -02864 Down |
| Atox1 | 0509595 | -018078 Down |
| Nploc4 | 0510588 | -043722 Down |
| Nf1 | 0510815 | -021075 Down |
| Rlim | 0512474 | -034711 Down |
| Grik5 | 051264 | 0380863 Up |
| Esf1 | 0513414 | -041724 Down |
| Reep5 | 0514639 | 037668 Up |
| Lyst | 0514821 | -026007 Down |
| Sav1 | 0515726 | -033408 Down |
| Dctn2 | 0515804 | -032125 Down |
| Arl8b | 0517006 | -018668 Down |
| Mki67 | 0517052 | 0484038 Up |
| Dhps | 0517166 | 0219838 Up |
| Cotl1 | 0517594 | -045196 Down |
| Zfp451 | 051825 | -035381 Down |
| Hps4 | 0519601 | 0322572 Up |
| Api5 | 0519799 | -043718 Down |
| Mrpl1 | 0520003 | 085197 Up |
| Frrs1 | 0520675 | -031958 Down |
| Ppil2 | 0520885 | -035944 Down |
| Pdcd4 | 0521124 | 0432483 Up |
| Spidr | 0521587 | -019096 Down |
| Ube2i | 0521947 | -037455 Down |
| Dcaf8 | 0522227 | 0159519 Up |
| Zranb2 | 0522993 | -031445 Down |
| Manf | 0523047 | -030003 Down |
| Ppp1r13b | 0523143 | -016243 Down |
| Cntn5 | 0523371 | -034823 Down |
| Fbxo42 | 0524032 | 0406176 Up |
| Ipo5 | 0524128 | -049099 Down |
| Ddx60 | 0524335 | -029018 Down |
| Slc25a36 | 052455 | -042312 Down |
| Ptk2b | 0525208 | -030633 Down |
| Babam2 | 0525572 | -024306 Down |

| Vps13a | 0525671 | 0223468 Up |
| --- | --- | --- |
| Nek9 | 0525902 | 0307986 Up |
| Prrc2a | 0525963 | 0303893 Up |
| Golgb1 | 0526084 | -017459 Down |
| AW549877 | 0526217 | -066483 Down |
| Gatm | 0526465 | -038789 Down |
| Rab43 | 0526917 | -018501 Down |
| Pign | 0527248 | -02685 Down |
| Ralbp1 | 0527311 | 0391258 Up |
| Fosb | 0527454 | 0360283 Up |
| Vps53 | 0527454 | -05478 Down |
| Cenpc1 | 0527522 | 0204353 Up |
| Pcdh15 | 0529372 | 0475893 Up |
| Ncl | 0530032 | -029296 Down |
| Gm16599 | 0530458 | -030514 Down |
| Anapc16 | 0531822 | 0350526 Up |
| Evi5 | 0531903 | 0237392 Up |
| Pcdh11x | 0532225 | -059272 Down |
| Khdrbs1 | 0532254 | 0210295 Up |
| Hspa14 | 0532352 | 0356492 Up |
| Numa1 | 0532459 | -021457 Down |
| Arpc1a | 0532549 | -031358 Down |
| Kat2b | 0532647 | -023621 Down |
| Chuk | 0532929 | 0209212 Up |
| Satb1 | 0533037 | 0632908 Up |
| Adam22 | 053461 | 0290258 Up |
| Lrp5 | 0534793 | -030641 Down |
| Poc1b | 0535374 | -026047 Down |
| Gtf2f2 | 0535383 | 0396118 Up |
| Ahi1 | 0535721 | -049759 Down |
| Rnf111 | 0536046 | -016395 Down |
| Srpr | 0536369 | -061638 Down |
| Flnb | 0537329 | -032148 Down |
| Lrrc58 | 0537479 | -04149 Down |
| Upf3b | 0538241 | -053662 Down |
| 8-Mar | 053829 | 0329185 Up |
| Msi2 | 0539049 | -023952 Down |
| Akirin1 | 0539199 | -041613 Down |
| Axin1 | 0539344 | 0288533 Up |
| Inpp5a | 0539556 | -026793 Down |
| Ncaph2 | 053957 | -056691 Down |
| Rgl2 | 0539791 | -034076 Down |
| Rictor | 0540265 | -044813 Down |
| Pkd1 | 0540512 | 0369569 Up |
| Eya4 | 0540663 | 0190592 Up |
| Fbxo34 | 0540876 | -015855 Down |
| Csnk2a2 | 0541307 | -032653 Down |
| Metap1 | 0541582 | -025348 Down |
| Cstf2 | 0541729 | -062693 Down |
| Dpyd | 0541792 | 0219242 Up |

| Atp6v1d | 0542713 | -041412 Down |
| --- | --- | --- |
| Srsf6 | 0543006 | -025175 Down |
| Wrn | 0543084 | -029473 Down |
| Klf3 | 0543679 | 0189293 Up |
| Calm3 | 0544202 | -023092 Down |
| Cacna1b | 0544635 | -031229 Down |
| Fancl | 0544819 | -03369 Down |
| Hsf2 | 0544987 | 0370479 Up |
| Dip2a | 054574 | 0279916 Up |
| Rabep1 | 0545869 | 0398167 Up |
| A830018L1 | 0546056 | -052222 Down |
| Samd4 | 0546289 | -01548 Down |
| Mthfd2l | 0546365 | -034988 Down |
| Klc1 | 0547379 | 0181946 Up |
| Nceh1 | 0547988 | -026719 Down |
| Yy1 | 054801 | -051544 Down |
| Ucp2 | 054817 | 0399815 Up |
| Ap1g1 | 0549354 | -021448 Down |
| Sdcbp | 0551891 | 0235657 Up |
| Ttc21b | 0552813 | -080239 Down |
| Pou2f1 | 0552981 | -015089 Down |
| Ubl5 | 0553138 | 02427 Up |
| Ric8b | 055315 | -037615 Down |
| Tiam2 | 0553455 | 0376556 Up |
| P2rx7 | 0553822 | -01734 Down |
| Gria2 | 055414 | -01937 Down |
| Lmbrd1 | 0555397 | 0182585 Up |
| Prune2 | 055632 | -015862 Down |
| Nrxn2 | 0556524 | -029668 Down |
| Hspa5 | 0557742 | -02364 Down |
| Sdccag8 | 0557928 | -025475 Down |
| Taf3 | 0557987 | -015133 Down |
| Zwint | 0558846 | 0325863 Up |
| Lrrc4 | 0559124 | -069267 Down |
| Fbxw2 | 0559319 | 0267293 Up |
| Ncoa3 | 0560106 | -033692 Down |
| Mfn1 | 0560837 | -016655 Down |
| BC002059 | 0561127 | -058359 Down |
| Pitpnb | 056159 | 0381244 Up |
| Vamp4 | 0563583 | 0207686 Up |
| Clip1 | 0564096 | -034315 Down |
| Mfsd1 | 0564133 | -032168 Down |
| Ccdc57 | 056426 | -053598 Down |
| Helq | 0564379 | -063271 Down |
| Vps33a | 0565091 | -046465 Down |
| 3110039M | 0565307 | -029951 Down |
| Zfp385b | 0566381 | 1223981 Up |
| Ndufa10 | 0566496 | 019553 Up |
| Kmt5b | 0567421 | 0241614 Up |
| Nup88 | 0567653 | 0324999 Up |

| Pde4b | 0568381 | -044767 Down |
| --- | --- | --- |
| Zfp68 | 0568597 | 0351788 Up |
| Abr | 0568682 | -02608 Down |
| Slc38a7 | 0568697 | -024353 Down |
| Gpc5 | 0568801 | 0577868 Up |
| Fbxl4 | 0569303 | 0303488 Up |
| Anapc5 | 056988 | -031742 Down |
| Camta1 | 0569993 | -016958 Down |
| Epc1 | 0573105 | -020491 Down |
| 4931406P1 | 057433 | 0376696 Up |
| Fnip2 | 0574606 | -050947 Down |
| Pip5k1a | 0574924 | -017286 Down |
| Arhgef11 | 057505 | 0186051 Up |
| Elmod1 | 0575914 | -054539 Down |
| Eif4h | 0576459 | -029911 Down |
| Mettl15 | 0577468 | -03633 Down |
| Hspe1 | 0577584 | 0235625 Up |
| Ccl25 | 057771 | 0294686 Up |
| Gak | 0577894 | 0221538 Up |
| Arpp19 | 0578191 | 0228682 Up |
| Bcl2l1 | 0578823 | -034319 Down |
| 4732471J0 | 0578893 | -021005 Down |
| Amfr | 0579061 | 0162358 Up |
| Tcaf1 | 058 | -025624 Down |
| Plekho1 | 0580361 | -025543 Down |
| Adam23 | 0580406 | 0217814 Up |
| Sox5 | 0580422 | -016746 Down |
| Rgs2 | 05806 | -019229 Down |
| Fam110b | 0580616 | -048512 Down |
| Sox6 | 0580788 | -036871 Down |
| Ran | 0580915 | 0392795 Up |
| Akap8 | 0581745 | -045583 Down |
| Pcmtd2 | 0582228 | 0315462 Up |
| Dhx40 | 0582612 | 0296315 Up |
| Rps4x | 0582789 | 0230415 Up |
| Commd3 | 0582872 | 035361 Up |
| Vamp8 | 0583671 | -021509 Down |
| Abhd18 | 0584045 | -035946 Down |
| Ypel5 | 0584096 | -027398 Down |
| Miga1 | 0584297 | -032877 Down |
| Tmx3 | 0584495 | -047154 Down |
| Ube3a | 058497 | 0204697 Up |
| Rbfox3 | 0585091 | -04471 Down |
| Aimp1 | 0585201 | -02101 Down |
| Dbi | 0585253 | -019014 Down |
| Usp24 | 0586138 | 0192491 Up |
| Golga5 | 0586355 | 0160116 Up |
| Pik3ca | 0586375 | 0151742 Up |
| Bank1 | 0586431 | -020965 Down |
| Cep350 | 0586604 | 0168102 Up |

| Foxo1 | 0586889 | -030735 Down |
| --- | --- | --- |
| Lars | 0587387 | -024446 Down |
| Adgrb3 | 058799 | -021332 Down |
| Yipf5 | 0588508 | 02046 Up |
| Nos1ap | 0588962 | -033003 Down |
| Sirpa | 0589296 | -025061 Down |
| Sfi1 | 0589576 | -025005 Down |
| Dcaf10 | 0589919 | 0602999 Up |
| Becn1 | 0590133 | -057751 Down |
| Rnpep | 0590146 | -019613 Down |
| Zfp654 | 0591107 | 0597752 Up |
| Rab39 | 0591683 | -022963 Down |
| Ppp1r2 | 0591748 | 0449378 Up |
| Spg7 | 0592512 | -052633 Down |
| Shank2 | 0592832 | -032796 Down |
| Rbx1 | 0593282 | 0280004 Up |
| Snw1 | 0593677 | 0189395 Up |
| Shisa9 | 0593865 | -053977 Down |
| Il16 | 0594653 | -032845 Down |
| B230219D | 0594781 | -027827 Down |
| Mpp6 | 0595269 | 0165783 Up |
| Lrif1 | 059569 | -033677 Down |
| Pcyt1a | 0596301 | -048293 Down |
| Psmb2 | 0596778 | 0181321 Up |
| Akr1a1 | 0597603 | -031665 Down |
| Ppcdc | 0597734 | -044297 Down |
| Csnk1g3 | 0597864 | -045565 Down |
| Nova1 | 0598833 | -020848 Down |
| Ywhaq | 0598927 | -022442 Down |
| Psma5 | 0599091 | -01572 Down |
| Dynll1 | 0599394 | 023635 Up |
| Glt8d1 | 0600057 | 0204806 Up |
| 1500004A1 | 0600096 | -017877 Down |
| Rasa3 | 0600096 | 028637 Up |
| Pias2 | 0600609 | -015719 Down |
| Smc6 | 0600656 | 0287957 Up |
| Odf2l | 0600768 | -031623 Down |
| Fbxw4 | 0601251 | -029005 Down |
| Usp46 | 060148 | 0218651 Up |
| U2af1 | 0602138 | -036791 Down |
| Nelfa | 0602314 | 0247664 Up |
| Chsy3 | 0602395 | 0262758 Up |
| Cct7 | 0603468 | -055325 Down |
| Mbtps1 | 060393 | 0193529 Up |
| Camsap1 | 0604472 | -022231 Down |
| Asah1 | 0604908 | 0154527 Up |
| Vdac2 | 0605119 | -03286 Down |
| Zc3h18 | 0605807 | -075235 Down |
| Kdm2a | 0606042 | 0166189 Up |
| Wdr3 | 0606624 | 0224275 Up |

| Psme4 | 0607314 | -021748 Down |
| --- | --- | --- |
| Ubc | 0607622 | -02215 Down |
| Cd300c2 | 0607903 | 016907 Up |
| Brd7 | 0608059 | -023626 Down |
| Abcb1b | 0608303 | 0213721 Up |
| Rprd2 | 0608556 | -034772 Down |
| Crim1 | 0609099 | 027039 Up |
| Rbm6 | 0609406 | -016963 Down |
| Kctd3 | 0610338 | -06961 Down |
| Tbca | 0610582 | -016063 Down |
| Pgm2l1 | 0611283 | 0355294 Up |
| Gtf3c2 | 0612475 | -027078 Down |
| Ap3m1 | 0612518 | -02204 Down |
| Zcchc8 | 0612874 | -031663 Down |
| Trmt1 | 0612924 | -069817 Down |
| Cct8 | 0614931 | -022619 Down |
| Maz | 0615326 | 0308004 Up |
| Acsl4 | 0615367 | -049334 Down |
| Il4ra | 061585 | 0222072 Up |
| Eipr1 | 0616357 | -03139 Down |
| Esco1 | 061676 | 0194915 Up |
| Slc20a2 | 0617448 | -027544 Down |
| Trim35 | 0617631 | -02121 Down |
| Uchl3 | 0618231 | -018346 Down |
| Alcam | 0618369 | 0233843 Up |
| Tiam1 | 0618775 | -039952 Down |
| Nek6 | 0619077 | 026099 Up |
| Phactr2 | 0619317 | 0243965 Up |
| Myt1l | 0619483 | -018803 Down |
| Abcf1 | 0619788 | 0283553 Up |
| Slc38a1 | 0620803 | -020776 Down |
| Rab11fip3 | 0621197 | -016769 Down |
| Pmm2 | 062146 | -02799 Down |
| Nfrkb | 0621469 | -036325 Down |
| Gpr107 | 0621496 | -02081 Down |
| Tecpr2 | 0621513 | -028347 Down |
| Setd7 | 0621621 | 0401376 Up |
| Rpl27 | 0622246 | 0206236 Up |
| Reln | 0622579 | 0319959 Up |
| Cox6a1 | 0622579 | 0184192 Up |
| Zfp266 | 0623827 | 0346089 Up |
| Dbnl | 0623845 | -022879 Down |
| Togaram1 | 0624254 | -016729 Down |
| Rasgef1b | 0624296 | -019525 Down |
| Krit1 | 0624532 | -019958 Down |
| Sh3pxd2b | 062541 | -015032 Down |
| Nme7 | 0625443 | 0359984 Up |
| Lsm7 | 0625632 | -057174 Down |
| Smarca4 | 0626552 | 0407036 Up |
| Snx25 | 0627264 | -036376 Down |

| Cbx5 | 0627516 | -015044 Down |
| --- | --- | --- |
| H2afv | 0628728 | -015435 Down |
| Syt11 | 0629104 | -017328 Down |
| Rell1 | 0629679 | 0170965 Up |
| Naa16 | 0629827 | -030326 Down |
| Mertk | 0629848 | 0196296 Up |
| Atg16l2 | 0630214 | -039477 Down |
| A330076H | 063052 | -033902 Down |
| 2700062C0 | 06306 | 0189574 Up |
| Nrf1 | 0630814 | -03597 Down |
| Nemf | 0631271 | 0312071 Up |
| Lonp2 | 0631673 | -029319 Down |
| Rtraf | 0631878 | 0403566 Up |
| Astn1 | 0632014 | -029487 Down |
| Slc44a1 | 0633303 | 0302507 Up |
| Cnrip1 | 0633442 | -036857 Down |
| Cnot10 | 0633459 | -017914 Down |
| Ube2d1 | 0634978 | -049679 Down |
| Etf1 | 0634987 | -029894 Down |
| Rxylt1 | 0635818 | 0238171 Up |
| Jkamp | 0636845 | 0696947 Up |
| 3-Sep | 0636949 | 0293773 Up |
| Numb | 0637136 | 0240822 Up |
| Notch2 | 0637241 | -03683 Down |
| Wdr1 | 0638285 | -032957 Down |
| Hdac4 | 0639828 | 0163479 Up |
| Pigk | 0639945 | -040821 Down |
| Cdip1 | 0640344 | 0308908 Up |
| Eif1b | 064075 | 0267417 Up |
| Odr4 | 0641071 | 0231461 Up |
| Unc5d | 0641238 | -048536 Down |
| Blvrb | 0641382 | 0236748 Up |
| Kansl1l | 0642231 | 0150923 Up |
| Epc2 | 064236 | -027128 Down |
| Siah1a | 0642618 | -026969 Down |
| Celf1 | 0642672 | 0164005 Up |
| Rad21 | 064341 | -021345 Down |
| Fgfr1op2 | 0644512 | -035159 Down |
| Slc12a9 | 0644742 | -017758 Down |
| Mgrn1 | 0645019 | -022613 Down |
| Eif4e | 0645492 | -029082 Down |
| Kdm3a | 0646149 | -022498 Down |
| Jazf1 | 0646355 | 024296 Up |
| Emc2 | 0646638 | -016319 Down |
| Psmb1 | 0647062 | -047396 Down |
| Ndufa4 | 0647256 | 0209372 Up |
| Phactr3 | 0647397 | -01538 Down |
| Rel | 0648491 | -032476 Down |
| Hnrnpr | 0648543 | -025709 Down |
| Prkdc | 065009 | 0175636 Up |

| Usp10 | 0650309 | -04657 Down |
| --- | --- | --- |
| Actr3 | 0650762 | -019203 Down |
| Zfp800 | 0650982 | -024869 Down |
| Washc4 | 0651853 | -025599 Down |
| Rims2 | 0652471 | -017637 Down |
| Supt16 | 0652682 | -027983 Down |
| Stard9 | 0652717 | -018872 Down |
| Gfpt1 | 0653094 | -019435 Down |
| Tor1aip1 | 0653257 | 0201587 Up |
| Cdk19 | 0654058 | 0376151 Up |
| Uggt2 | 0654107 | -021698 Down |
| Gria3 | 0654739 | -034273 Down |
| Timm13 | 0654807 | 0240899 Up |
| Mff | 0654888 | 0222699 Up |
| Lilrb4a | 0655107 | -065569 Down |
| Tial1 | 065512 | -019375 Down |
| Tomm34 | 0657247 | 0391178 Up |
| BC052040 | 0658729 | 0430784 Up |
| Rapgef2 | 0658944 | 015024 Up |
| Nipa2 | 0659193 | 0157308 Up |
| Ube2g1 | 0659202 | -054664 Down |
| C5ar1 | 0659681 | 0316335 Up |
| Gpr137b | 0659926 | -052297 Down |
| Mpeg1 | 0660495 | -021155 Down |
| Scpep1 | 0660827 | 0751119 Up |
| Rnf157 | 0661031 | -017557 Down |
| Robo1 | 0661265 | -017693 Down |
| Pcnx | 0661784 | -03143 Down |
| Galntl6 | 0662133 | -018175 Down |
| Srsf1 | 06625 | -049852 Down |
| Gnpda1 | 0663435 | -015014 Down |
| Foxk2 | 0663671 | -04362 Down |
| Med14 | 0665262 | -03839 Down |
| Kpna3 | 0665526 | -01657 Down |
| Gm30382 | 0665614 | 0341527 Up |
| Fggy | 0665791 | 0625482 Up |
| Cep68 | 0666267 | -034751 Down |
| Orc2 | 0666573 | 02456 Up |
| Wbp11 | 0667052 | -032602 Down |
| Uxs1 | 0667204 | 0417489 Up |
| Maged1 | 0668318 | -017315 Down |
| Napg | 0668425 | 0344084 Up |
| Fubp3 | 0668809 | 0476229 Up |
| Tmed9 | 0669344 | -030962 Down |
| Slc25a28 | 0669792 | -052268 Down |
| Fat3 | 0670079 | -015509 Down |
| Stk4 | 067034 | -034036 Down |
| Ipo11 | 0670769 | -016266 Down |
| Dhx30 | 0671027 | 0308532 Up |
| Vav1 | 0672239 | -018604 Down |

| Trerf1 | 0672646 | -025104 Down |
| --- | --- | --- |
| Atp2b2 | 0673914 | -019209 Down |
| Gsdmd | 0674079 | -063596 Down |
| Nol11 | 0674832 | -018317 Down |
| Npas3 | 0675186 | -017458 Down |
| Gm26871 | 0675186 | -030218 Down |
| St3gal3 | 0675695 | -01753 Down |
| Smap2 | 0676776 | 0210168 Up |
| Iqgap1 | 0677165 | -052795 Down |
| Cd81 | 0678943 | -017978 Down |
| Supt5 | 067984 | 0207762 Up |
| Ylpm1 | 0680601 | 0202524 Up |
| Zfpm2 | 068175 | -053438 Down |
| Slc4a10 | 0681954 | -042647 Down |
| Dnm3 | 0681967 | 0288861 Up |
| Snd1 | 0681997 | -029342 Down |
| Ankrd28 | 0682635 | -024617 Down |
| Rpl18a | 0683348 | -01694 Down |
| Gabbr2 | 0683578 | -034703 Down |
| Snx1 | 068415 | -024905 Down |
| Aff1 | 0684173 | 0172857 Up |
| Cdk17 | 0684707 | -019122 Down |
| Ammecr1 | 0685163 | -022844 Down |
| Fbxo3 | 0686046 | -022129 Down |
| Elmsan1 | 0686348 | 0171326 Up |
| Scn2a | 068636 | 0342778 Up |
| Phf6 | 0686741 | 0362115 Up |
| Sergef | 0687077 | -023097 Down |
| Epha5 | 0687199 | -017439 Down |
| Capn7 | 0687909 | -019245 Down |
| Stmn3 | 0688818 | -020708 Down |
| BC005561 | 0689141 | -020793 Down |
| Acadsb | 0689166 | 0206107 Up |
| Ifnar2 | 0689271 | -027492 Down |
| Srsf7 | 0689313 | 0172773 Up |
| Mical2 | 0689353 | -034741 Down |
| Trim26 | 0690741 | 0316285 Up |
| Cntnap5a | 0690787 | -036508 Down |
| Stau1 | 069139 | -032125 Down |
| Zfp426 | 0691669 | 0330954 Up |
| Tardbp | 069177 | -020295 Down |
| Cited2 | 0692087 | 0194327 Up |
| Camk1 | 0692169 | -016355 Down |
| Cops5 | 0693984 | -028552 Down |
| Nup54 | 0693987 | 0347154 Up |
| Sgip1 | 069409 | -036627 Down |
| Kifap3 | 0694129 | -024314 Down |
| Snx10 | 0694181 | 0206305 Up |
| Syk | 0695234 | -026812 Down |
| Amn1 | 0696423 | 0325791 Up |

| Tcn2 | 0697 | 0224018 Up |
| --- | --- | --- |
| Zfyve1 | 0697748 | -018083 Down |
| Rapgef4 | 0697829 | 0258751 Up |
| Mrpl33 | 0699795 | 0237516 Up |
| Stambpl1 | 0700275 | -026956 Down |
| Otud7b | 0700783 | 023886 Up |
| Vdac3 | 0700844 | -030208 Down |
| Cyb5b | 0700844 | -033196 Down |
| Bsn | 0700959 | 0273849 Up |
| Nsd2 | 0701868 | 0242268 Up |
| Trp53bp1 | 0702703 | -031219 Down |
| Actn1 | 0703068 | -030377 Down |
| Stat2 | 0703342 | -020167 Down |
| Pfdn1 | 0703505 | 0419507 Up |
| Pcyt2 | 0704455 | 0281555 Up |
| Pi4k2a | 0704466 | 0253583 Up |
| Gnb2 | 0704758 | -015194 Down |
| Pbx3 | 0705227 | 0255494 Up |
| Ybx1 | 0705506 | -022236 Down |
| Tshz1 | 0705621 | 0728362 Up |
| Arhgap30 | 0706278 | -025818 Down |
| Map4k5 | 070638 | 0316584 Up |
| Arhgap45 | 0706484 | -026444 Down |
| Soat1 | 0706947 | 0271386 Up |
| Ppp6r2 | 0707627 | -049549 Down |
| Emsy | 0708671 | -039018 Down |
| Prr14l | 0709886 | -0256 Down |
| Hadha | 071104 | -031284 Down |
| Slc33a1 | 0711817 | 0363307 Up |
| Ptdss1 | 0711953 | 0433614 Up |
| Bfar | 0712374 | -032439 Down |
| P2rx4 | 071243 | 0261846 Up |
| Cryzl1 | 0712883 | -022963 Down |
| Pcdh9 | 0712938 | 0157387 Up |
| Rabac1 | 0713677 | -033288 Down |
| Nrp2 | 0713716 | 0152177 Up |
| Zfp991 | 0714294 | -019168 Down |
| Sox4 | 0714341 | 0237685 Up |
| Znfx1 | 0714764 | -048013 Down |
| Las1l | 0715177 | 044955 Up |
| Dop1a | 0715402 | -020978 Down |
| Slk | 0715402 | -030843 Down |
| Kif26b | 0715854 | 0212298 Up |
| Ehbp1 | 0716089 | -025036 Down |
| Tpcn1 | 0716445 | -030128 Down |
| Tomm20 | 071665 | 0367085 Up |
| Mprip | 0716739 | -016915 Down |
| Tmem183a | 0717178 | -031172 Down |
| Gpr160 | 0717744 | 0162412 Up |
| Zkscan1 | 0717806 | 0163532 Up |

| Topbp1 | 0718312 | -01675 Down |
| --- | --- | --- |
| Shank1 | 071881 | -077295 Down |
| Pkm | 071909 | 0195292 Up |
| Morf4l2 | 0719703 | 0158319 Up |
| Sik2 | 0719921 | -036679 Down |
| Gigyf1 | 0720011 | -03818 Down |
| Ppp3r1 | 0720808 | -019474 Down |
| 9930111J2 | 0721387 | 0273875 Up |
| Dync1li2 | 0722467 | 0673897 Up |
| Plxnc1 | 0723023 | -022732 Down |
| Lhfpl3 | 0723598 | -045682 Down |
| Tbc1d12 | 0724632 | -017578 Down |
| Ypel3 | 0725071 | -020339 Down |
| Sox11 | 0725248 | -030082 Down |
| Cnr1 | 0725557 | 0289501 Up |
| Auh | 0725557 | -041779 Down |
| Lman1 | 0725633 | 0401947 Up |
| Nav2 | 0726319 | -017615 Down |
| Chd1 | 0727259 | -017141 Down |
| Nkain3 | 0728228 | -032193 Down |
| Stt3a | 0728406 | -040231 Down |
| Nbas | 0728431 | 0151683 Up |
| Slc27a1 | 0728581 | 0167136 Up |
| Mark1 | 0728604 | -02073 Down |
| Wdr91 | 0729103 | -029205 Down |
| Agpat5 | 072925 | -044468 Down |
| Erlec1 | 0731274 | 0305489 Up |
| Ddx10 | 0731427 | -025889 Down |
| Ncbp3 | 0731456 | -018316 Down |
| Nol4 | 0731895 | -02362 Down |
| Agrn | 0731958 | 0310188 Up |
| Mtmr2 | 0732021 | -01853 Down |
| Map4 | 0732113 | -021641 Down |
| Acaca | 0732518 | -026372 Down |
| Plppr1 | 0732565 | -016036 Down |
| Dcp1a | 0732638 | -03553 Down |
| Ttc5 | 0732638 | -039345 Down |
| Sec63 | 073296 | -028369 Down |
| Rbms3 | 0733141 | 0298964 Up |
| Ube2e3 | 0733211 | 0219541 Up |
| Vim | 0733369 | -038158 Down |
| Arhgap4 | 0734165 | -042512 Down |
| Il10ra | 0734543 | 031525 Up |
| 5430405H0 | 0734662 | 0312748 Up |
| Map1b | 0734964 | 0177811 Up |
| Nlrp1b | 0735004 | -025591 Down |
| Slc44a2 | 0735051 | 0206451 Up |
| Shoc2 | 0735379 | -025962 Down |
| Ncoa6 | 0735663 | 0268427 Up |
| Mthfs | 0735742 | -023136 Down |

| Ppp4r3a | 0736646 | -035123 Down |
| --- | --- | --- |
| Gabpb1 | 0736822 | 0171062 Up |
| Ndufab1 | 0736922 | 0180336 Up |
| Nars | 073694 | -01942 Down |
| Frs2 | 0738221 | -028951 Down |
| Ap1ar | 0738233 | -015398 Down |
| Fut8 | 0738274 | 0222232 Up |
| Uqcc1 | 0739179 | 0286833 Up |
| Ercc6l2 | 0739412 | -018724 Down |
| Rnf41 | 0739742 | 0182231 Up |
| Ctcf | 0739947 | -026566 Down |
| Washc5 | 0741109 | 02849 Up |
| Dab1 | 0741528 | -017749 Down |
| Immt | 0741636 | -028374 Down |
| Wipf2 | 0742239 | -028008 Down |
| Dock3 | 0742729 | -024739 Down |
| Ipo8 | 0742888 | 0242891 Up |
| Armc9 | 0743167 | -029851 Down |
| Sgcz | 0743246 | -017995 Down |
| Pnn | 0743501 | 0169362 Up |
| Rassf2 | 0743528 | 0486289 Up |
| Fyttd1 | 0743817 | 0226305 Up |
| Nav1 | 0743887 | -016704 Down |
| Cdc123 | 0744714 | 0191323 Up |
| Mfsd14a | 0745523 | 0155897 Up |
| Tmem208 | 0745605 | 0189267 Up |
| B4galt5 | 0746881 | -042524 Down |
| Zbtb11 | 0747319 | -02297 Down |
| Ppp6c | 07474 | 0304244 Up |
| Clec2d | 0748059 | -031986 Down |
| Kmt2a | 0748096 | -01888 Down |
| Arpp21 | 0748726 | -018452 Down |
| Dstn | 0750056 | -045227 Down |
| Arid3a | 0750206 | -048696 Down |
| Tasp1 | 0750275 | -02624 Down |
| Rab2a | 0750284 | 0172258 Up |
| Taldo1 | 0750508 | -051911 Down |
| Uevld | 075119 | -018924 Down |
| Tango2 | 0751418 | -019345 Down |
| Selenow | 0751422 | -03427 Down |
| Lyl1 | 0751636 | -064428 Down |
| Gria4 | 0753537 | -016953 Down |
| Dbn1 | 0753874 | 0265941 Up |
| Fam126a | 0754385 | -024282 Down |
| Sumf1 | 075468 | 0212596 Up |
| Elf4 | 0755363 | -026761 Down |
| Fermt3 | 0756012 | -017729 Down |
| Uckl1 | 0756393 | 0528094 Up |
| Itpr1 | 0756447 | -03346 Down |
| Clta | 0757317 | -021451 Down |

| Foxn2 | 0757581 | 0363203 Up |
| --- | --- | --- |
| Firre | 0758537 | -029106 Down |
| Klhl9 | 0759316 | -041935 Down |
| Ssbp2 | 0760032 | 0161918 Up |
| Cct2 | 0760164 | -021441 Down |
| Naxd | 0761249 | 0514138 Up |
| Dscam | 0761397 | 0290346 Up |
| Mms22l | 0761662 | -027504 Down |
| Herpud2 | 0762211 | -03972 Down |
| Atxn7l1 | 0762247 | -01553 Down |
| Sh3d19 | 0762423 | 0341862 Up |
| Hnrnpul1 | 0762823 | 0161082 Up |
| Fut9 | 0763414 | 0157381 Up |
| 1700017B0 | 0763581 | -024111 Down |
| Arid1b | 076452 | -0234 Down |
| Rprd1b | 0765063 | -033251 Down |
| Agk | 076509 | -027778 Down |
| Appbp2 | 0765832 | 0299226 Up |
| 2610008E1 | 0766054 | -053535 Down |
| Wdr11 | 0766553 | -027237 Down |
| Ctr9 | 076713 | -033349 Down |
| Gtf2ird2 | 0767955 | 0310617 Up |
| Eml6 | 0768769 | 0172065 Up |
| Ccdc34 | 0770288 | -036747 Down |
| Slc30a9 | 0770369 | -020994 Down |
| Unc5c | 0770716 | -043617 Down |
| Zkscan3 | 0771691 | 0268517 Up |
| Mysm1 | 0771743 | 0211401 Up |
| Rnf19a | 0772047 | 0198051 Up |
| Slc10a7 | 0772418 | 0215194 Up |
| Snrnp200 | 0773181 | -022247 Down |
| N4bp2 | 0773853 | 0424387 Up |
| Akap11 | 0774076 | -03472 Down |
| Clcn4 | 0774248 | -016756 Down |
| Lrpprc | 0775122 | -038739 Down |
| Psma7 | 0775407 | -025548 Down |
| Igfbpl1 | 0775413 | -042769 Down |
| Cdkl3 | 0775587 | -019286 Down |
| Vps37a | 0775641 | -024638 Down |
| Fstl5 | 0775641 | -035109 Down |
| Alg13 | 0775983 | -030414 Down |
| Tmem126a | 077601 | 0492258 Up |
| Wdr47 | 0777147 | -024453 Down |
| Stx18 | 0779679 | -024809 Down |
| Hs3st4 | 0780314 | -015943 Down |
| Kmt2d | 0780561 | -018873 Down |
| Brd3 | 078086 | -021601 Down |
| Rnf8 | 0781532 | 0563427 Up |
| Arpc1b | 0781878 | -018588 Down |
| Ubxn4 | 078333 | -015785 Down |

| Nln | 0784109 | -016546 Down |
| --- | --- | --- |
| Akt3 | 0784464 | -021728 Down |
| Stx5a | 078462 | -026751 Down |
| Polr3c | 0785667 | 0151295 Up |
| Mfsd14b | 0786743 | -015744 Down |
| 2810013P0 | 0787284 | -039379 Down |
| 2610507B1 | 0788228 | -02209 Down |
| Meis2 | 0788619 | -032706 Down |
| Slc22a17 | 0789322 | 0486936 Up |
| 1700025G | 0790198 | 0319926 Up |
| Smg7 | 0790419 | 0268206 Up |
| Atp5f1 | 0791031 | -023248 Down |
| Arhgap12 | 0791077 | -026887 Down |
| Il27 | 0791961 | 0322683 Up |
| Ern1 | 0792066 | -045054 Down |
| Wdr20 | 0792434 | 029355 Up |
| C3ar1 | 0793285 | -02941 Down |
| Syt1 | 0793946 | 0211164 Up |
| Acer3 | 0794708 | 0182005 Up |
| Rgs6 | 079483 | -039973 Down |
| Mir9-3hg | 0795098 | -023102 Down |
| Nrg3 | 0795134 | -025959 Down |
| Cbfa2t2 | 0795741 | -030948 Down |
| Wwc2 | 0795799 | -028135 Down |
| Ascc2 | 0797012 | 0157074 Up |
| Ralgps2 | 0799593 | -039485 Down |
| D630045J1 | 0799747 | -031735 Down |
| Slc35b3 | 0801424 | -020244 Down |
| Ehmt1 | 0801613 | -02008 Down |
| Fam118b | 0802767 | 0188942 Up |
| Vti1a | 0804601 | 0156755 Up |
| Cflar | 0806239 | -026385 Down |
| Alas1 | 0806734 | -033003 Down |
| Commd1 | 0808159 | 0202785 Up |
| Mbnl2 | 080822 | -049573 Down |
| Ddx1 | 0808448 | 0282687 Up |
| Ccnc | 0809052 | -056479 Down |
| Snrpe | 0809067 | -032077 Down |
| Dicer1 | 0809924 | 038363 Up |
| Ica1 | 0810305 | 0173135 Up |
| Klf10 | 0810607 | -026345 Down |
| Psma6 | 0810698 | -018553 Down |
| Lrrtm3 | 0811737 | -02914 Down |
| Med27 | 0812737 | -0304 Down |
| Ccser2 | 0814701 | -04011 Down |
| Smarca2 | 0815725 | 0271191 Up |
| Ssr1 | 0815768 | 0176508 Up |
| Gps2 | 0816104 | -031196 Down |
| Lmnb1 | 0818872 | -029855 Down |
| Sp4 | 0819568 | 0170378 Up |

| Parp14 | 0819644 | -027074 Down |
| --- | --- | --- |
| Reps2 | 0820053 | 0156869 Up |
| Usp14 | 0821544 | -017539 Down |
| Tmem179b | 0821879 | -023948 Down |
| Klhl24 | 0822498 | -028112 Down |
| Crot | 0822685 | 0278152 Up |
| Ss18 | 0822981 | -037424 Down |
| Gbe1 | 0823073 | 0184354 Up |
| Ddx3x | 082356 | -016602 Down |
| Crybg3 | 0823803 | 0224264 Up |
| Cntn3 | 0824034 | -102024 Down |
| Ttll4 | 0824238 | -034977 Down |
| Hck | 0824684 | -045692 Down |
| Hpcal1 | 0825168 | 0158645 Up |
| Puf60 | 0825415 | -016385 Down |
| Kat6a | 0826066 | -015444 Down |
| Rap2b | 0826498 | -026043 Down |
| Tsc22d2 | 0826963 | -018803 Down |
| Atp5h | 0827189 | -048482 Down |
| Dars | 0827731 | -025465 Down |
| Ndufs5 | 0828372 | 0402794 Up |
| Mtf2 | 0828492 | -034136 Down |
| Prickle2 | 0828735 | -018321 Down |
| Fam155a | 0829156 | 0280818 Up |
| Smarcb1 | 0829582 | 0253442 Up |
| Arl6ip1 | 0829872 | -03292 Down |
| M6pr | 0830816 | -053465 Down |
| Spin1 | 0831037 | -030714 Down |
| Galnt13 | 0832214 | 0582092 Up |
| Scoc | 083304 | -034752 Down |
| Stub1 | 0834052 | 0195088 Up |
| Fgfr1op | 0834052 | -052987 Down |
| Tmem144 | 083436 | -047956 Down |
| Pank2 | 0835039 | 0387921 Up |
| St13 | 0835943 | -017265 Down |
| Negr1 | 0836022 | -034224 Down |
| Mvb12b | 0836034 | -01866 Down |
| Supt6 | 0836184 | -018702 Down |
| Far1 | 0837627 | -023998 Down |
| Tkt | 0837771 | -047088 Down |
| Golga1 | 0838183 | 0367973 Up |
| Gm20528 | 0838652 | -027284 Down |
| Phyhd1 | 0839062 | -07048 Down |
| Zc3h7a | 0839177 | -0159 Down |
| Trak2 | 0839466 | 0419725 Up |
| Hibadh | 0839748 | -019214 Down |
| Kcnk10 | 084064 | -031136 Down |
| Pfkfb4 | 0841219 | 0196535 Up |
| Ubr2 | 0842094 | -027648 Down |
| Mxi1 | 0842997 | -035022 Down |

| Zcchc4 | 0843566 | 0315548 Up |
| --- | --- | --- |
| Snhg11 | 0843726 | 0371666 Up |
| H13 | 0843858 | -02051 Down |
| Sh3gl2 | 0844417 | 0155095 Up |
| Efna5 | 0844536 | -019537 Down |
| Wdr48 | 0844777 | 0351952 Up |
| Hsbp1 | 0845507 | -02291 Down |
| Elavl2 | 0845922 | -023714 Down |
| Arpc2 | 0845961 | -019349 Down |
| Pkd2 | 0846053 | 0341586 Up |
| Aplp1 | 0846053 | 0294487 Up |
| Sgk1 | 0846183 | 0242482 Up |
| Igf2bp3 | 0846591 | -023226 Down |
| Grina | 0847761 | -015068 Down |
| Mtmr1 | 0847836 | -025467 Down |
| Tomm70a | 0847995 | -032697 Down |
| Gripap1 | 0848875 | -019232 Down |
| Uqcrc2 | 0849046 | -023303 Down |
| Tenm1 | 0849629 | 0218505 Up |
| Ecpas | 0851526 | -018497 Down |
| Tef | 0851856 | 0248863 Up |
| Slc25a3 | 0851912 | -016512 Down |
| Rnf10 | 0852728 | -019949 Down |
| Spen | 0853495 | -021265 Down |
| Sema6d | 0853715 | -020575 Down |
| Creg1 | 0853799 | -019535 Down |
| Ap3s1 | 0854909 | -018509 Down |
| Rab6a | 0856404 | -01718 Down |
| Fabp7 | 0857513 | -019695 Down |
| Nmd3 | 0858193 | 0318672 Up |
| Sipa1l3 | 0858193 | 0416797 Up |
| Neo1 | 0859354 | -018709 Down |
| Tulp4 | 0860539 | -018929 Down |
| Dtna | 0860978 | -045284 Down |
| H1f0 | 0860979 | 0168802 Up |
| Csmd2 | 0861273 | -025646 Down |
| Sec23a | 0861356 | 0383079 Up |
| Fbxw7 | 0861795 | 0198655 Up |
| Prrc1 | 0861981 | -0178 Down |
| Adcy1 | 0862989 | 0518858 Up |
| Cacnb4 | 0863966 | 0285669 Up |
| Erp44 | 0864718 | -024497 Down |
| Pisd | 0866276 | -046434 Down |
| Desi2 | 0866676 | 0173921 Up |
| Samhd1 | 0867753 | 0197229 Up |
| Oxa1l | 0868148 | -019336 Down |
| Rnf121 | 0868871 | -022489 Down |
| Rsu1 | 0871255 | 0164333 Up |
| Kat14 | 0872044 | 0269952 Up |
| Mios | 0872424 | -026986 Down |

| Sptan1 | 0874173 | -015446 Down |
| --- | --- | --- |
| Gabrg2 | 0875495 | -01861 Down |
| Cplane1 | 0875755 | -031328 Down |
| Rif1 | 0876464 | -020294 Down |
| Rnf130 | 0877733 | -015208 Down |
| Elp2 | 0878832 | 0168201 Up |
| Spast | 0878867 | -018567 Down |
| Frmd5 | 0879448 | 0255252 Up |
| Galnt7 | 087956 | -022761 Down |
| Slc30a7 | 0879757 | 0454035 Up |
| Iars2 | 0880872 | -028268 Down |
| Aopep | 0881706 | -015282 Down |
| Prdx1 | 0881755 | -025888 Down |
| Ptpn2 | 0881924 | -04945 Down |
| Nrg2 | 0883138 | 053414 Up |
| Clstn2 | 0883248 | 05843 Up |
| Gm17106 | 0883717 | 0291407 Up |
| Bcas2 | 0884148 | -05517 Down |
| Exoc6 | 0884198 | -026745 Down |
| Otud5 | 0884821 | -015418 Down |
| Vps26a | 0885252 | 0160103 Up |
| Susd1 | 088583 | -017378 Down |
| Cdc16 | 0885975 | -068418 Down |
| Dennd4c | 0886674 | -020424 Down |
| Nek1 | 0886952 | 0208755 Up |
| Malsu1 | 0887098 | -044238 Down |
| Ube4b | 0887633 | 016938 Up |
| Zc3h7b | 0887748 | 0624769 Up |
| Sf3b6 | 0888372 | -020877 Down |
| Parp6 | 0889067 | -015219 Down |
| Sh3bp2 | 0889392 | -033799 Down |
| Thap3 | 0889999 | -015745 Down |
| Myo18a | 0890093 | -045765 Down |
| Gm13710 | 0890107 | -016977 Down |
| Rexo1 | 0890226 | -015143 Down |
| Selenos | 0890773 | 0304413 Up |
| Ahcyl1 | 0890968 | 0402074 Up |
| Adgrl3 | 0891013 | -015304 Down |
| Scn3a | 0891287 | -019996 Down |
| Adap2os | 0891302 | -03095 Down |
| Shprh | 0891433 | 0258859 Up |
| Gpm6b | 0892216 | 0195458 Up |
| Disc1 | 0892304 | -025128 Down |
| Mapre1 | 0892397 | -030437 Down |
| Zfp277 | 0892895 | -023989 Down |
| Tmem87a | 0893389 | 0182322 Up |
| Otud7a | 0893499 | -021913 Down |
| Ablim1 | 0893505 | -026658 Down |
| Adam10 | 0893866 | -017188 Down |
| Zc3h15 | 0893897 | -018901 Down |

| Yaf2 | 0894217 | -043692 Down |
| --- | --- | --- |
| Gm40841 | 0894569 | -026827 Down |
| Mcph1 | 0895055 | -027309 Down |
| Uqcrc1 | 0895392 | -018523 Down |
| Tbc1d9b | 0896045 | -015248 Down |
| Synj2bp | 0896258 | 0335119 Up |
| 11-Sep | 0896389 | -036128 Down |
| Mpp7 | 0896611 | 0217343 Up |
| Cdh10 | 08996 | 0211042 Up |
| Fam219a | 0899666 | -026832 Down |
| Rpl12 | 0899846 | -017918 Down |
| Pphln1 | 0900209 | -055756 Down |
| Pstpip2 | 0901332 | -033088 Down |
| Aebp2 | 0901872 | 043908 Up |
| Smad5 | 0902428 | -032457 Down |
| Gatad2b | 0902515 | -028556 Down |
| Setd1b | 0903373 | -028311 Down |
| Cenpv | 0906752 | -042667 Down |
| Epha3 | 0908848 | 0225978 Up |
| Cttnbp2 | 0909515 | -020119 Down |
| Mapk8ip3 | 0909798 | 0208683 Up |
| Trim37 | 0910848 | -016913 Down |
| Timm44 | 0911904 | 0260416 Up |
| Cab39l | 0912309 | -034165 Down |
| Dtnb | 091428 | -018163 Down |
| Dennd1b | 0915843 | -023424 Down |
| Cirbp | 0916813 | -052764 Down |
| Rnf2 | 0916822 | 0264709 Up |
| Gclc | 09176 | 0812849 Up |
| Gabrb1 | 0919379 | 0341594 Up |
| Asap2 | 0920018 | -022557 Down |
| Ndufa7 | 0920429 | 0278356 Up |
| 0610012G | 0920727 | -043619 Down |
| Micu3 | 0922006 | 017273 Up |
| Unc79 | 0922538 | 036059 Up |
| Akt1 | 092315 | -034575 Down |
| Atp6v1b2 | 0924786 | -020491 Down |
| Cr1l | 0926317 | 0161537 Up |
| Pik3r6 | 0926536 | -021697 Down |
| Pts | 0926627 | 0191535 Up |
| Gm37240 | 092711 | 0189655 Up |
| Edil3 | 0930108 | 0299646 Up |
| Srp72 | 0930421 | -023028 Down |
| Fam135b | 0930636 | -018774 Down |
| Fbh1 | 0931784 | 02742 Up |
| Idh3b | 0932504 | 0183291 Up |
| Tug1 | 093351 | -020125 Down |
| Dtymk | 0933921 | 0329655 Up |
| Zcchc2 | 0934411 | -018645 Down |
| Ykt6 | 0934535 | -059748 Down |

| Wdpcp | 0935195 | 0176305 Up |
| --- | --- | --- |
| Mfhas1 | 0935972 | -018966 Down |
| Tmem131l | 0936663 | -015272 Down |
| Casd1 | 0936746 | -019623 Down |
| Pcsk2 | 0936825 | -027171 Down |
| Sulf2 | 0937072 | -069451 Down |
| Naa50 | 0937531 | -031559 Down |
| Fam107b | 0937557 | 046703 Up |
| Sf1 | 0937672 | -015584 Down |
| Rad51b | 0937967 | 0186392 Up |
| Primpol | 0938009 | -038512 Down |
| Gm20754 | 0938265 | -020413 Down |
| Map2 | 0938319 | -019128 Down |
| Atxn2l | 094041 | 0241468 Up |
| Tmed10 | 0940821 | 020095 Up |
| Tsg101 | 0940942 | -016454 Down |
| Mgmt | 0941639 | -047172 Down |
| Rbl1 | 0942148 | -053535 Down |
| Strip1 | 0942174 | -026576 Down |
| Pcnt | 0942769 | -032375 Down |
| Homer1 | 0942792 | 0238178 Up |
| Nxph1 | 0942948 | 0445374 Up |
| Tmem59 | 0943831 | 0265409 Up |
| Rad54l2 | 0944257 | 021011 Up |
| Flrt2 | 0944278 | -024289 Down |
| Pola1 | 0944547 | 0452194 Up |
| Ddx42 | 0944588 | -020462 Down |
| Kdm5b | 0944665 | -028339 Down |
| Cst3 | 0945012 | -01591 Down |
| Snhg12 | 0946936 | -049712 Down |
| Itgam | 0946936 | -020786 Down |
| Crtc3 | 0947183 | -034487 Down |
| Swt1 | 094801 | -035671 Down |
| Hdac8 | 0948772 | -037747 Down |
| Lmbr1 | 0949431 | -03931 Down |
| Ttc39b | 0950019 | 0241698 Up |
| Hpgd | 0950153 | 057703 Up |
| Lrig2 | 0950397 | -015076 Down |
| Ripk1 | 0951438 | -023709 Down |
| Wdr12 | 0952826 | -02195 Down |
| Cep192 | 0954878 | -015292 Down |
| Epha6 | 095524 | -016331 Down |
| Eif3e | 0955423 | -036722 Down |
| Zbtb44 | 0956494 | 0211456 Up |
| Eif2a | 0956745 | -019861 Down |
| Map1lc3b | 0957416 | -033773 Down |
| Luzp2 | 0957637 | -057171 Down |
| Pik3c2a | 0957957 | -016343 Down |
| Sh2b2 | 0959777 | -025729 Down |
| Grm3 | 0960657 | -016503 Down |

| Podxl2 | 0961073 | 029687 Up |
| --- | --- | --- |
| Sbno2 | 0961481 | 0274734 Up |
| Jund | 0962389 | -029475 Down |
| Magi2 | 0963948 | -019148 Down |
| Fmn2 | 0964039 | -043139 Down |
| Dcp1b | 0964093 | -044483 Down |
| Soga3 | 0964589 | 0225425 Up |
| Ttll5 | 0965776 | -046961 Down |
| Ndufs2 | 0965803 | -04805 Down |
| Pla2g15 | 0965884 | -023414 Down |
| L3mbtl3 | 0966654 | -032072 Down |
| Ssb | 09684 | -023091 Down |
| Gmeb1 | 096869 | 0190581 Up |
| Arhgdib | 0968956 | -034426 Down |
| Suco | 0970319 | 0174868 Up |
| Entr1 | 0973061 | -02763 Down |
| Cntn6 | 0973401 | 0530398 Up |
| Cntln | 0973496 | -026508 Down |
| Cntnap2 | 0973705 | 031337 Up |
| Cbfa2t3 | 097382 | -032435 Down |
| Rps27 | 0973912 | -019914 Down |
| Dcaf17 | 0974975 | -027797 Down |
| Tgfbrap1 | 0975711 | 0347445 Up |
| Gprasp1 | 0975819 | 0302959 Up |
| Arfgef3 | 0975923 | -029643 Down |
| Iars | 0976146 | -037848 Down |
| Neto2 | 0976638 | 0327819 Up |
| Parg | 0977353 | -015514 Down |
| Znrf3 | 0977835 | -016536 Down |
| Akirin2 | 0978151 | -030918 Down |
| Cntnap4 | 0978536 | -052797 Down |
| Opcml | 0980341 | 0707737 Up |
| Sqstm1 | 0981394 | -016703 Down |
| Arid5b | 0981668 | -02127 Down |
| Cops9 | 0982071 | 0280506 Up |
| Klhl5 | 0982428 | -022245 Down |
| Cemip2 | 0984287 | 028027 Up |
| Mtch2 | 0984737 | 0420672 Up |
| Syncrip | 0985859 | 0157156 Up |
| Pnpla8 | 0986651 | -022872 Down |
| Pkn1 | 0986989 | 0241276 Up |
| Pde7a | 0987256 | 0270072 Up |
| Ergic2 | 0988256 | -019456 Down |
| Vkorc1l1 | 0989199 | -039472 Down |
| B3galt1 | 0989415 | 0156038 Up |
| Sec22a | 0989651 | -02919 Down |
| Sem1 | 0989663 | -031264 Down |
| Psma1 | 0989741 | -021372 Down |
| Bnip2 | 0990951 | 0228709 Up |
| Cdk9 | 0990991 | -053892 Down |

| Sf3b3 | 0991678 | -025723 Down |
| --- | --- | --- |
| Zfhx3 | 0992063 | -015107 Down |
| Hmox2 | 0992947 | -017459 Down |
| Cisd2 | 0993208 | -015886 Down |
| Pard3 | 0994003 | 0293063 Up |
| Apaf1 | 099486 | 0221433 Up |
| Elavl3 | 099596 | -017352 Down |
| Sec61g | 0996238 | -015668 Down |
| Sft2d1 | 0996533 | -024437 Down |
| Robo2 | 099699 | 0192279 Up |
| Pkig | 0997898 | -035967 Down |
| Uba5 | 0998174 | -025645 Down |
| Csf2rb | 0999369 | -019177 Down |
| Acadl | 0999378 | -035936 Down |
| Stmn4 | 0999403 | -017272 Down |

Supplemental table 5: Annotation table of cell type and related marker genes

**Cell clusters	marker genes**

**Single cell transcriptomic**

Neural stem cells(NSC)	Ascl1, Top2a, MKI67, Hmgb2, Nes

Astrocyte	Pdgfrb, Aldoc

Ependymal	Foxj1

Microglia	Ctss

Oligodendrocyte	Oligo2, Pdgfra

Neuroblasts	Top2a, Hmgb2

Cortical upper-layer excitatory neurons	Satb2, Ldb2

MGE-derived inhibitory interneurons	Gad1, Gad2, Lhx6

GABAergic neuroblasts	Gad1,Gad2, Id4, Npy

Thalamus neurons	Nrg3, Erbb4, Dcx, Sp9

iMSNs	Drd2, Bcl11b, Sp9

Excitatory neurons	Slc17a5, Camk2a

Excitatory neuron progenitor cells	Neurod2, Slc17a6

PV+ inhibitory interneurons	Gad1, Gad2, Lhfpl3

Immature inhibitory neuron	Npy, Stmn2, Tubb3

Cajal-Retzius (CR) cells	Ebf2, Reln Diencephalic projection neuron-like cluster	Slc17a6, Rora, Lef1 Endothelial	Cldn5, Vtn

VLMC(Vascular and leptomeningeal cells)	Serpinh1, Lgals1, Bst2

**Spatial transcriptomic**

**Cell clusters	marker genes**

Neural stem progenitor cells(NSPC)	Sox2, Mki67, Eomes

Deeper-layer neurons	Sox5, Tbr1

Lateral septum	Tmem130,Fxyd6

MGE-derived interneurons	Sst, Npy, Lhx6

Olfactory tubercle	Penk, Gng7

Striatum	Foxp1, Pcp4

Upper-layer neurons	Cux1,Cux2

Intermediate zone	Sox5, Dcx

Vascular and leptomeningeal cells	Vtn, Lgals1, Col1a2

Red blood cells	Hbb-bs, Hbb-bt

Choroid plexus	Ttr, Col9a3

Microglia	Ctss, C1qc, Apoe

|  | A | B | C | D | E | F | G | H | I | J |
| --- | --- | --- | --- | --- | --- | --- | --- | --- | --- | --- |
| 1 | Supplemental table 6: Sequencing data information | | | | | | | | | |
| 2 | ID | Experiment title | Point | Plateform | Library construction | Sequencing type | Run data file type | Open data |  |  |
| 3 | 1 | E17.5 CKO | 3380 | Illumina NovaSeq 6000 | 10x Genomics | scRNA-seq | 10x files | <https://ngdc.cncb.ac.cn/omix/preview/PVzxrgn8> | | |
| 4 | 2 | E17.5 WT | 3276 | Illumina NovaSeq 6000 | 10x Genomics | scRNA-seq | 10x files | <https://ngdc.cncb.ac.cn/omix/preview/PVzxrgn8> | | |
| 5 | 3 | P2 CKO | 9386 | Illumina NovaSeq 6000 | 10x Genomics | scMultiome-seq | 10x files | <https://ngdc.cncb.ac.cn/omix/preview/PVzxrgn8> | | |
| 6 | 4 | P2 WT | 8211 | Illumina NovaSeq 6000 | 10x Genomics | scMultiome-seq | 10x files | <https://ngdc.cncb.ac.cn/omix/preview/PVzxrgn8> | | |
| 7 | 5 | P2 CKO | 101853 | MGISEQ-2000RS | STOmics | Stereo-seq | h5ad | <https://ngdc.cncb.ac.cn/omix/preview/PVzxrgn8> | | |
| 8 | 6 | P2 WT | 126579 | MGISEQ-2000RS | STOmics | Stereo-seq | h5ad | <https://ngdc.cncb.ac.cn/omix/preview/PVzxrgn8> | | |
